# Methanogens and Methanotrophs Show Nutrient-Dependent Community Assemblage Patterns Across Tropical Peatlands of the Pastaza-Marañón Basin, Peruvian Amazonia

**DOI:** 10.3389/fmicb.2020.00746

**Published:** 2020-04-24

**Authors:** Damien Robert Finn, Michal Ziv-El, Joost van Haren, Jin Gyoon Park, Jhon del Aguila-Pasquel, Jose David Urquiza–Muñoz, Hinsby Cadillo-Quiroz

**Affiliations:** ^1^School of Life Sciences, Arizona State University, Tempe, AZ, United States; ^2^Swette Center for Environmental Biotechnology, Biodesign Institute, Arizona State University, Tempe, AZ, United States; ^3^Biosphere 2, University of Arizona, Tucson, AZ, United States; ^4^Center for Personalized Diagnostics, Biodesign Institute, Arizona State University, Tempe, AZ, United States; ^5^Instituto de Investigaciones de la Amazonia Peruana, Iquitos, Peru; ^6^Laboratorio de Suelos del Centro de Investigaciones de Recursos Naturales de la Amazonia Peruana, and Facultad de Ciencias Forestales, Universidad de la Amazonia Peruana, Iquitos, Peru; ^7^Center for Fundamental and Applied Microbiomics, Biodesign Institute, Arizona State University, Tempe, AZ, United States

**Keywords:** greenhouse gases, methane, peatlands, amazon, methanogens, methanotrophs, community assemblage

## Abstract

Tropical peatlands are globally important carbon reservoirs that play a crucial role in fluxes of atmospheric greenhouse gases. Amazon peatlands are expected to be large source of atmospheric methane (CH_4_) emissions, however little is understood about the rates of CH_4_ flux or the microorganisms that mediate it in these environments. Here we studied a mineral nutrient gradient across peatlands in the Pastaza-Marañón Basin, the largest tropical peatland in South America, to describe CH_4_ fluxes and environmental factors that regulate species assemblages of methanogenic and methanotrophic microorganisms. Peatlands were grouped as minerotrophic, mixed and ombrotrophic categories by their general water source leading to different mineral nutrient content (rich, mixed and poor) quantified by trace elements abundance. Microbial communities clustered dependent on nutrient content (ANOSIM *p* < 0.001). Higher CH_4_ flux was associated with minerotrophic communities compared to the other categories. The most dominant methanogens and methanotrophs were represented by *Methanobacteriaceae*, and *Methylocystaceae*, respectively. Weighted network analysis demonstrated tight clustering of most methanogen families with minerotrophic-associated microbial families. Populations of *Methylocystaceae* were present across all peatlands. Null model testing for species assemblage patterns and species rank distributions confirmed non-random aggregations of *Methylococcacae* methanotroph and methanogen families (*p* < 0.05). We conclude that in studied amazon peatlands increasing mineral nutrient content provides favorable habitats for *Methanobacteriaceae*, while *Methylocystaceae* populations seem to broadly distribute independent of nutrient content.

## Introduction

Peatlands are ecosystems where plant primary productivity exceeds organic matter decomposition, resulting in the accumulation of partially decomposed soil organic matter, acting as globally important organic carbon (OC) reserves (Page et al., 2011; Sjogersten et al., 2014; Page and Baird, 2016). While northern, low-temperature peatlands have been extensively characterized in terms of their microbial communities (Andersen et al., 2013; Serkebaeva et al., 2013; Zhou et al., 2017), little is understood about tropical peatlands, particularly for those in the Amazon basin. Amazon peatlands have been heavily understudied due to the lack of reports of their existence prior to 2008 (Lahteenoja et al., 2009), however over 80 550 km^2^ containing 7.07 Gt OC have been reported so far, representing 18% of the surface area and 8.3% of the OC content of global tropical peatlands (Rieley and Page, 2016). Due to the high OC content, Amazon peatlands are expected to produce significant amounts of greenhouse gases (GHG) such as carbon dioxide (CO_2_), methane (CH_4_) and nitrous oxide (N_2_O). In fact, recent early reports on GHGs fluxes from a few Amazon peatlands show significant yet highly variable levels of CH_4_ and N_2_O across sites, across seasons in a site, or across gradients within in a site (Teh et al., 2017; Winton et al., 2017). Regionally, it has been coarsely estimated that the Amazon Basin can emit 31.6 – 41.1 Tg CH_4_ year^–1^, or approximately 7% of global CH_4_ emissions (Wilson et al., 2016; Teh et al., 2017; Winton et al., 2017) but such an estimate is based on too few data points. To our knowledge there is a limited availability of GHG flux reports from Amazon peatlands and no study has addressed the composition and potential functional patterns of soil microbial communities and their association with CH_4_ flux in Amazon peatlands.

To better understand the role of microbes participating in CH_4_ flux it is important to evaluate the environmental controls (i.e., pH, nutrient content, others) as well as microbe-microbe interactions affecting the activity of CH_4_ producers or consumers. Methane production is performed by methanogenic *Archaea* (methanogens) as the final stage of the organic matter decomposition cascade, where a broad range of heterotrophic organisms metabolize complex organic molecules releasing simpler methanogenic substrates (acetate, formate, methanol and CO_2_) (Garcia et al., 2000). Consequently, methanogens are metabolically dependent on active heterotrophs in peatlands. Methanogens are metabolically and physiologically diverse, and are primarily grouped taxonomically in the Orders Methanopyrales, Methanococcales, Methanobacteriales, Methanomicrobiales, and Methanosarcinales (Garcia et al., 2000; Thauer et al., 2008). Conversely, CH_4_ can also act as a carbon and energy source for specific microbial species known as methanotrophs. These predominantly include aerobic Gamma- and Alpha-proteobacteria of the *Methylococcaceae* and *Methylocystaceae* families, respectively (Hanson and Hanson, 1996), aerobic Verrucomicrobia (Dunfield et al., 2007), *Archaea* that anaerobically oxidize CH_4_ via coupling with reduction of alternative terminal electron acceptors [like sulfate (Orphan et al., 2002; Orphan et al., 2009) nitrate (Haroon et al., 2013), Fe or Mn (Ettwig et al., 2016)], and NC10 bacteria that is capable to produce its own oxygen from nitrite and oxidize CH_4_ in anaerobic environments (Ettwig et al., 2010).

Studies of the ecological diversity of methanogens in tropical peatlands globally are few and in some cases with contrasting results. In Peat Swamp Forest in Thailand, hydrogenotrophic members of the Class Methanomicrobia (Kanokratana et al., 2011) were found dominant, while an equivalent peatland in Malaysia putative methylotrophic Methanomassiliicoccales methanogens were identified as the dominant (Too et al., 2018). The first study is in agreement with well-characterized northern peatlands, in which several studies demonstrated that the Order Methanomicrobiales and, to lesser extent, hydrogenotrophic members of the Methanobacteriales and acetoclastic Methanosarcinales are important for CH_4_ production (Juottonen et al., 2005; Cadillo-Quiroz et al., 2006; Steinberg and Regan, 2008; Lin et al., 2012). However, the result of the second study indicates that there is significant methanogen’s (and plausible methanotrophs) variation that is yet to be accounted in tropical peatlands. To our knowledge, the diversity of methanotrophs in Amazon peatlands has not been documented. Contrasting reports have been observed in other tropical regions where a tropical peatland in Indonesia showed a dominance of *Methylomonas* sp. from the *Methylococcaceae* (Arai et al., 2014), while a Malaysian swamp forest peatland showed *Methylocystaceae* (Too et al., 2018) as the dominant. In northern peatlands, the dominant methanotrophic bacteria tend to befree living or *Sphagnum*-moss symbionts of the family *Methylocystaceae* (Chen et al., 2008; Dedysh, 2009; Wieczorek et al., 2011; Kip et al., 2012).

Naturally existing nutrient gradients have historically been used by ecologists to identify environmental parameters that govern observable species assemblages (Tilman and Wedin, 1991; Keddy, 1992; McGill et al., 2006). Geochemical and nutrient gradients in northern and tropical peatlands are typically associated to the dominant water source described as follows: a) minerotrophic -groundwater fed fens or swamps are relatively nutrient-rich; b) ombrotrophic -rain fed bogs are nutrient-poor; and c) mixed water source poor fens or swamps have intermediate nutrient levels (Juottonen et al., 2005; Cadillo-Quiroz et al., 2006; Martini et al., 2007; Lahteenoja and Page, 2011). The biodiversity of methanogenic species has previously been shown to change across peatland geochemical gradients (Juottonen et al., 2005; Cadillo-Quiroz et al., 2006; Lin et al., 2012), but attempts to describe a set of community-assembly associations for tropical peatland methanogens has yet to be considered.

Here we describe GHG flux variation and the first accounts of prokaryotic community compositions across a tropical peatland nutrient gradient in the Pastaza-Marañón Basin, in the western Amazonia, Peru. The Pastaza-Marañón Basin holds the most extensive tropical peatland distribution in South America (Lahteenoja et al., 2012; Draper et al., 2014). A rich diversity of ecosystem types, based on nutrient content, exists here due to mineral deposition from dynamic hydrological activity of river channels originating in the Andes, or rain-fed, or mix water source-fed peat formation (Lahteenoja et al., 2012). The soils included in this study thus originated from peatlands showing minerotrophic (Charo, San Roque, and Buena Vista), mixed (Nueva York and Quistococha) and ombrotrophic (Miraflores and San Jorge) conditions. Sites were assigned to categories as in previous reports (Lahteenoja et al., 2009; Teh et al., 2017) based on soil analysis and site characteristics, plus an additional assessment based on soil mineral content of sodium, magnesium, phosphorous, sulfate, potassium, calcium, manganese, iron, nickel, copper, and zinc (Table 1) as quantifiable geochemical proxies to the nutrient conditions provided by ground water-fed (minerotrophic) or rain-fed (ombrotrophic) or mixed water sources in peatlands (Whitfield et al., 2009).

**TABLE 1:** List of categories, location, atmospheric records, and soil properties of 7 peatlands from the Pastaza-Marañón Basin.

**Soil**	**San Roque**	**Buena Vista**	**Charo**	**Quistococha**	**Nueva York**	**Miraflores**	**San Jorge**
Soil category	Minerotrophic	Minerotrophic	Minerotrophic	Mixed	Mixed	Ombrotrophic	Ombrotrophic
Latitude	−3.5333	−4.2398	−4.2633	−3.8611	−4.3835	−4.4166	−4.0609
Longitude	−74.6168	−73.2028	−73.257	−73.3794	−74.2667	−74.0667	−73.1851
MAT (°C)	26	25.8	26	26.4	26.6	26.6	26.4
MAP (mm)	3037.7	2506.8	2506.8	2987.4	2882.7	2882.7	2506.8
Bulk Density (g cm^–3^)	0.11 ± 0	0.09 ± 0	0.11 ± 0	0.11 ± 0	0.09 ± 0	0.13 ± 0	0.1 ± 0
pH	5.9 ± 0.2^a^	5.6 ± 0.1^a^	5.9 ± 0.1^a^	3.7 ± 0.1^b^	3.2 ± 0^b^	2.9 ± 0.1^c^	2.5 ± 0^c^
Alpha Diversity (H’)	3.78 ± 0.23^a^	3.84 ± 0.04^a^	4.07 ± 0.14^a^	3.16 ± 0.03^b^	3.43 ± 0.13^b^	3.52 ± 0.2^c^	3.55 ± 0.1^c^
Evenness	0.57 ± 0.03^a^	0.58 ± 0.01^a^	0.61 ± 0.02^a^	0.5 ± 0^b^	0.55 ± 0.03^b^	0.57 ± 0.03^a^	0.58 ± 0.03^a^
Na (mg kg^–1^ dry soil)	150.6 ± 14.5^a^	273.8 ± 66^a^	1667.9 ± 151.4^a^	79.4 ± 24.7^b^	68.7 ± 44.4^b^	63.1 ± 28.9^b^	39 ± 7.4^b^
Mg (mg kg^–1^ dry soil)	525.5 ± 270^a^	729 ± 71.2^a^	2358.8 ± 1081^a^	374.6 ± 43.6^b^	221.3 ± 196^b^	196.1 ± 82.7^b^	276.3 ± 59.5^b^
P (mg kg^–1^ dry soil)	797.6 ± 105.7^a^	1113.1 ± 339.6^a^	1261.4 ± 89.9^a^	1048.2 ± 27.6^b^	613.5 ± 522.1^b^	850.9 ± 377.7^b^	676.5 ± 139.6^b^
S (mg kg^–1^ dry soil)	2675.8 ± 663.5^a^	4746.7 ± 718.3^a^	2914.5 ± 350.1^a^	2786.7 ± 547.7^b^	2738.9 ± 1058.6^b^	1401.3 ± 404.2^b^	1863.3 ± 161.1^b^
K (mg kg^–1^ dry soil)	656.8 ± 61.9^a^	1763.3 ± 741.4^a^	6972.4 ± 885.5^a^	452.9 ± 18.4^b^	441.2 ± 384.4^b^	469.4 ± 137.4^b^	372.6 ± 110.9^b^
Ca (mg kg^–1^ dry soil)	8094 ± 1245.3^a^	6516.1 ± 244.1^a^	10508.2 ± 928.8^a^	4875.5 ± 1303.1^b^	241.6 ± 122.7^b^	257.8 ± 214.9^b^	952 ± 269.2^b^
Mn (mg kg^–1^ dry soil)	247.4 ± 33.2^a^	70.3 ± 27.7^a^	411.2 ± 73.8^a^	49.2 ± 12.3^b^	26.5 ± 19.9^b^	17.5 ± 10.7^b^	35.5 ± 7.1^b^
Fe (g kg^–1^ dry soil)	8.3 ± 0.9^a^	7.1 ± 2.8^a^	356 ± 90^a^	3.3 ± 3.8^b^	3.3 ± 0.7^b^	2.1 ± 1.3^b^	1.3 ± 0.1^b^
Ni (mg kg^–1^ dry soil)	4.8 ± 0.5^a^	7.4 ± 2.4^a^	16.7 ± 3.6^a^	2.3 ± 0.1^b^	1.6 ± 1.3^b^	2 ± 0.7^b^	1.9 ± 0.3^b^
Cu (mg kg^–1^ dry soil)	7.3 ± 0.7^a^	9.8 ± 3.3^a^	39.4 ± 3.6^a^	3.2 ± 0.3^b^	2.2 ± 1.4^b^	4.6 ± 1.2^b^	6 ± 0.9^b^
Zn (mg kg^–1^ dry soil)	13.4 ± 1.3^a^	29 ± 11^a^	51.7 ± 4.2^a^	10.6 ± 3.6^b^	15.9 ± 3^b^	21.2 ± 5.5^c^	20.6 ± 5.5^c^

*Soil values are averages and standard deviations representing six independent measurements per site. MAT = Mean Annual Temperature, MAP = Mean Annual Precipitation. Physicochemical properties were evaluated for statistically significant differences among categories with a Least Significant Difference *post hoc* test where different letters (a, b, c) indicate significant differences across 3 categories (minerotrophic, mixed and ombrotrophic) at the *p* = 0.05 level.*

We hypothesized the following: 1) nutrient concentration variation, measured as elemental composition via inductively coupled plasma-mass spectrometry (ICP-MS), among peatlands with contrasting geochemistry influenced by their source of water shapes the overall microbial community composition; and 2) such variations in nutrient concentration can be associated with CH_4_ fluxes as well as explain discrete (non-random) assemblages of methanogen and methanotroph families in the study sites.

## Materials and Methods

### Site Description and Field Sampling

Seven study sites were selected in the Pastaza-Marañon basin, in the Peruvian Western Amazon region (Table 1). Petland information regarding vegetation, geochemistry and peat depths have been described previously for Quistococha (Roucoux et al., 2013), San Jorge and Buena Vista (Kelly et al., 2013), San Roque, Miraflores, and Charo (Lahteenoja et al., 2009) and Nueva York (Lahteenoja and Page, 2011). Annual climatological data was obtained from the database described in Espinoza Villar et al. (2009). Samples were collected in December of 2011 and December of 2012 near the end of the “dry” season or a transitional period to rainy conditions. On-site measurements included pH, air and soil temperature using a HI 99121N meter (Hanna Instruments, RI, United States), and fixed soil volume collection (400 cm^3^) for bulk density analysis (Chambers et al., 2010). A 100-m long transect near the center of the peatland with north to south direction was done using center location and soil characterization from previous studies (Lahteenoja et al., 2009; Lahteenoja et al., 2012) to better represent peat geochemical conditions. In transect two pair of independent points (∼10 m apart in east to west direction) were placed every 50 m (6 sampling points per site with variable distances from 20 to ∼160 m). Soil samples were collected from the center of sampling points shortly after flux measurements were completed and when relevant at two depths (0–15 and 15–30 cm) (12 samples per site). For each of the six sampling points in the transect, a 15 cm deep and 5 cm diameter mini soil core was collected in sterile bags and mixed, then subsamples were taken for corresponding analysis: triplicated 0.5 gr were aseptically placed in sterile tubes and mixed with 1 ml of MoBio Lifeguard Soil stabilization solution (MoBio, United States), ∼50 g of soil was sealed in a bag for physical or chemical analysis. All samples were stored in refrigerated conditions within 24–48 h of collection during field expedition, and subsequently frozen and stored at −20C (for chemical) or −80C (for DNA) until appropriate analysis. GHG fluxes were measured at each of the six soil sampling points at the soil surface. All flux measurements were conducted in a time window of ±3 h from noon (9 am– 3 pm) using metal static vented soil chambers (Keller et al., 2005) enclosing a 0.045 m^2^ area, where collars with airproof rubber seals in their base were affix to ground with 30 cm stakes at least 30 min before measurements. After the chamber reached pressure equilibration, air samples were taken with plastic syringes at 10, 20, 30, and 40 min and transferred into nitrogen-flushed, pre-evacuated 6 mL glass vials sealed with butyl stoppers (Bellco, NJ, United States).

### Soil and Gas Chemistry

A Quadrupole ICP-MS (Thermo Electron X series with CCT, Fisher Scientific, United States) was used to analyze soil elemental composition in the Metals, Environmental and Terrestrial Analytical Laboratory, part of the Chemical and Environmental Characterization Core Facilities at Arizona State University. Between 1.5 – 2.5 g of soil was dried at 80°C for 72 h, digested in hydrofluoric, nitric and hydrochloric acid (12 and 3 h steps), followed by microwave heat digestion (30 min) and final evaporation. ICP-MS analysis of diluted samples in nitric acid along with standards for all elements evaluated done as described elsewhere (Potts, 1992). GHG were analyzed on a modified SRI Greenhouse Gas GC (SRI Instruments, CA, United States) with dual valco valves for separate gas streams for a Flame Ionization Detector (FID) for CO_2_ and CH_4_ and an Electron Conductivity Detector (ECD) for N_2_O. The flux rates were calculated from a linear change in trace gas concentration over time with reference to the internal volume of the chamber and the soil area covered (Hutchinson and Livingston, 1993).

### Molecular Analyses

MoBio Lifeguard soil stabilization solution was removed by centrifugation and supernatant removal. Then, genomic DNA was extracted from 0.5 g of soil (wet weight) using a MoBio UltraClean Soil DNA isolation kit (MoBio, CA, United States), with the addition of aluminum ammonium sulfate (5 mM) prior to bead beating. Extracted DNA was quantified with a Qubit fluorometer (Invitrogen, MA, United States) assessed via gel electrophoresis. PCR amplifications were carried out using the 515F (5′-GTGYCAGCMGCCGCGGTA) (Baker et al., 2003) and 909R (5′-CCCCGYCAATTCMTTTRAGT) (Wang and Qian, 2009; Tamaki et al., 2011) primers that cover the V4 and V5 regions of the 16S rRNA gene of *Bacteria* and *Archaea*. Unique barcodes of 6 – 10 base pairs were attached to the 5′ end of primers. The PCR was carried out with 1.2 U μL^–1^ high-fidelity Hotstart Q5 Taq polymerase (New England Biosciences, MA, United States). Thermocycler parameters were: 98°C for 30 s, followed by 25 cycles of 98°C for 10 s, 63°C for 15 s, and 72°C for 15 s, and a final extension at 72°C for 2 min. Each sample was amplified in triplicate and pooled to reduce sequencing bias. PCR products were cleaned via Agencourt AMPure XP beads (Beckman Coulter, CA, United States), and quantified via Bioanalyzer (Agilent Technologies, CA, United States). The Illumina TruSeq DNA Sample Prep kit was used to prepare libraries and sequenced on the Illumina MiSeq v2 platform (Illumina, CA, United States). Paired-end sequences were quality filtered as described elsewhere (Bokulich et al., 2013). Paired-end reads were demultiplexed with an in house script. Downstream analyses for OTU annotation was performed with the Quantitative Insights into Microbial Ecology Two (Qiime2) pipeline (Caporaso et al., 2010). Chimeric sequences were removed with Uchime (Edgar et al., 2011). Closed-reference operational taxonomic unit (OTU) picking at 97% identity was used in relation to the Silva 128 database (Quast et al., 2013). Four 16S rRNA gene analyses performed on Northern peatlands from Sphagnum-dominated bog at Grand Rapids, Minnesota, United States (SRA IDs SRR6514003, SRR6514014, SRR6514002 and SRR6514033), were included in Qiime2 analyses for coarse comparative purposes against Amazon peatlands. Sequences of this study were deposited under SRA Bioproject ID PRJNA501909.

### Statistical Analyses

All statistical analyses were performed in R v 3.4.1 (R Development Core Team, 2013). Analysis of variance (ANOVA) was used to compare soil physicochemical variables followed by least-significant difference (LSD) testing (*p* < 0.05) across defined site categories (minerotrophic, mixed, ombrotrophic). Also, a Permutational multivariate analysis of variance (PERMANOVA) analysis was completed on soil physicochemical variables to test whether the tree site categories will better explained soil conditions than any two-way combination (R2, *p* < 0.001). ANOVA was also used to compare CO_2_ gas fluxes, and Kruskal Wallis for CH_4_ and N_2_O gas fluxes. Fluxes were visualized as a stacked barplot, with LSD (*p* < 0.05) using the defined categories plus a Northern peatland one. Alpha diversity (H′) and evenness, as well as ordination of microbial communities with non-metric multidimensional scaling (NMDS) and fitting of climatological variables, environmental variables and GHG fluxes was performed as described elsewhere (Oksanen et al., 2013). Chloroplast and mitochondrial OTUs were identified and removed from the family level matrix. Weighted network analysis was performed on OTU covariance at the family level, with edge weights filtered at the *p* = 0.05 level determined by Spearman correlation (Csardi and Nepusz, 2006). The Spinglass technique was used to calculate modularity of the network (Reichardt and Bornholdt, 2006). Networks for minerotrophic, mixed and ombrotrophic sites were generated as above, and the frequency of methanogen and methanotroph connections to other nodes were summed, with corresponding nodes reported at the phylum level. Kruskal-Wallis tests were performed for methanogen and methanotroph family abundance with site classification as a categorical variable. Rank Abundance calculations were performed as described elsewhere (Kindt and Coe, 2005). A log-normal model (Equation 1) and a geometric model (Equation 2) were fit to log family abundances over ranks with non-linear least squares, and model fits were compared via Akaike Information Criteria (AIC) (R Development Core Team, 2013). The log-normal model is as follows:

(1) $$S\,\left(R\right)=S_{o}\,e^{\left(-a^{2}\,R^{2}\right)}$$

Where S(R) is abundance of the R^th^ rank, *S*_*o*_ is the abundance of the first rank, *a* is an inverse measure of the distribution width, and *R* corresponds to the R^th^ rank (Ludwig and Reynolds, 1988). The geometric model is as follows:

(2) S(R)=S+Op(1-p)R

Where S(R) is abundance of the R^th^ rank, *S*_0_ is the abundance of the first rank, *p* is the proportional decrease in abundance and *R* corresponds to the R^th^ rank (Dolan et al., 2007). Finally, community assembly patterns of methanogen and methanotroph families were conducted using variance ratio (V-ratio) (Gotelli, 2000). Null model testing of 1000 random permutations of the family matrix was performed with fixed sums and equiprobable distribution across sites (Gotelli, 2000).

## Results

### Soil Properties and Gas Fluxes

**FIGURE 1:**
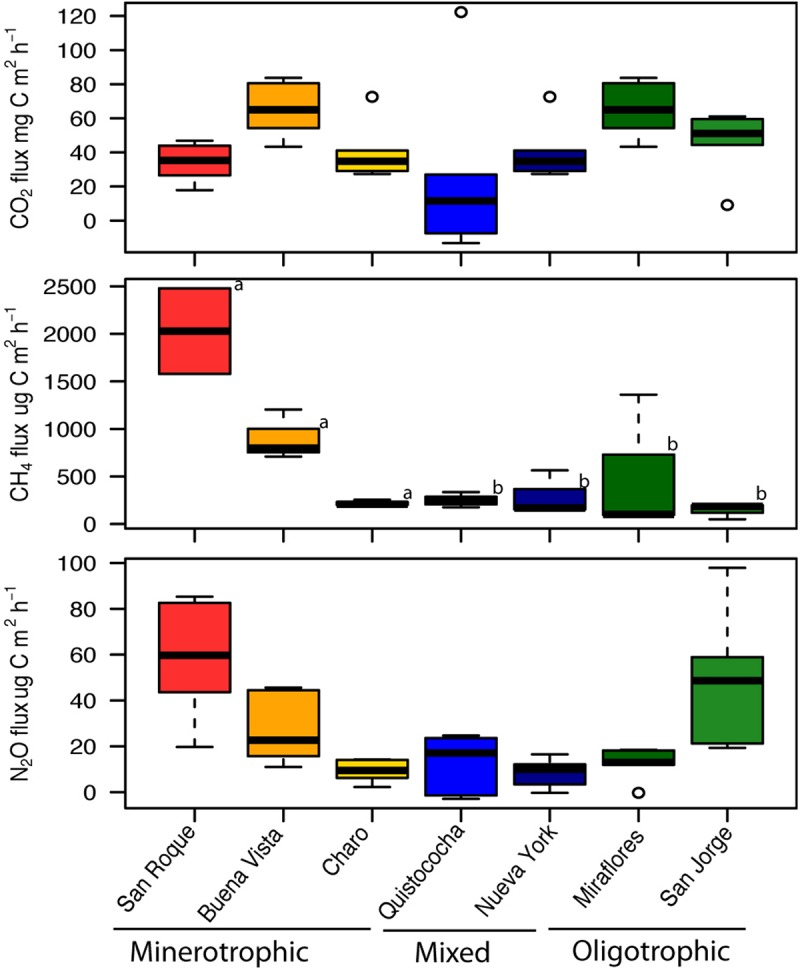
Greenhouse gas fluxes of CO_2_ (mg C m^2^ h^–1^), CH_4_ (μg C m^2^ h^–1^), and N_2_O (μg N m^2^ h^–1^) across the Pastaza-Marañón Basin. Box plots show the mean and quartiles of six independent measurements. All flux measurements were evaluated for statistically significant differences among categories with a Least Significant Difference *post hoc* test where different letters (a, b) indicate significant differences across site categories (minerotrophic, mixed and ombrotrophic) at the *p* = 0.05 level.

In soil properties measured across study sites (Table 1), a general trend was observed across the geochemical and nutrient gradient (minerotrophic to ombrotrophic) of decreasing pH and soil elemental nutrient concentration, with the ombrotrophic sites demonstrating low pH (<3) (LSD, *p* < 0.05). No trends were noted for mean annual temperature (MAT), mean annual precipitation (MAP), H′ or evenness. The concentration of elemental soil nutrients consistently supported the separation of the minerotrophic soil category from mixed and ombrotrophic (LSD, *p* = 005); but not of the last two due to replicate variability despite lower means in ombrotrophic soils (Table 1). However, multivariate analysis of mineral nutrient, pH and bulk soil measurements (PERMANOVA, *p* < 0.001), showed the separation of mixed and ombrotrophic categories (in addition to minerotrophic), has the highest R2 fit (33%) than any pairwise (up to 26%) and thus better represent the variation across sites supporting our use of tree site categories for Amazon peatland as in this study. For gas flux comparisons (Figure 1), carbon dioxide fluxes differed among all peatlands (ANOVA, *p* = 0.04), but these differences were independent of the elemental nutrient gradient (ANOVA, *p* = 0.09). Similarly, N_2_O flux had high variation among sites but the recorded levels (∼0.5–100 ug N m^–2^ h^–1^) were highly specific to individual peatlands (Kruskal-Wallis, *p* = < 0.001), independent of the nutrient gradient (Kruskal-Wallis, *p* = 0.05). Conversely, CH_4_ flux showed a dependence on the elemental nutrient gradient (Kruskal-Wallis, *p* = 0.03) and independent of individual peatlands (Kruskal-Wallis, *p* = 0.1). San Roque and Buena Vista (minerotrophic), which had an order of magnitude greater CH_4_ flux than the other sites, drove this. Charo, also a minerotrophic site, had an unusually low CH_4_ flux compared to the San Roque and Buena Vista sites. CH_4_ flux remained relatively low for all mixed and ombrotrophic sites.

### Relationships Between Communities Across the Nutrient Gradient

**FIGURE 2:**
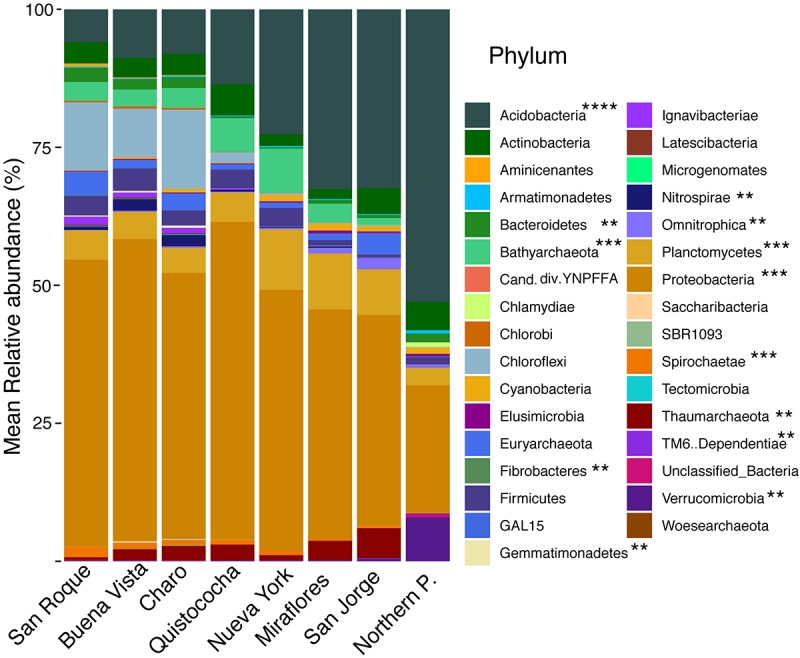
Stacked barplot of relative abundances (%) of *Bacterial* and *Archaeal* phyla across the Pastaza-Marañón Basin. Significant differences between phyla in the minerotrophic, mixed, ombrotrophic and Northern peatland groups are shown as: (**) differences between two groups; (***) differences between three groups; and (****) all groups differed. Phyla above 0.005% are shown. Relative abundances are the mean of six independent measurements for Amazon soils, and four for the Northern peatland soil.

**FIGURE 3:**
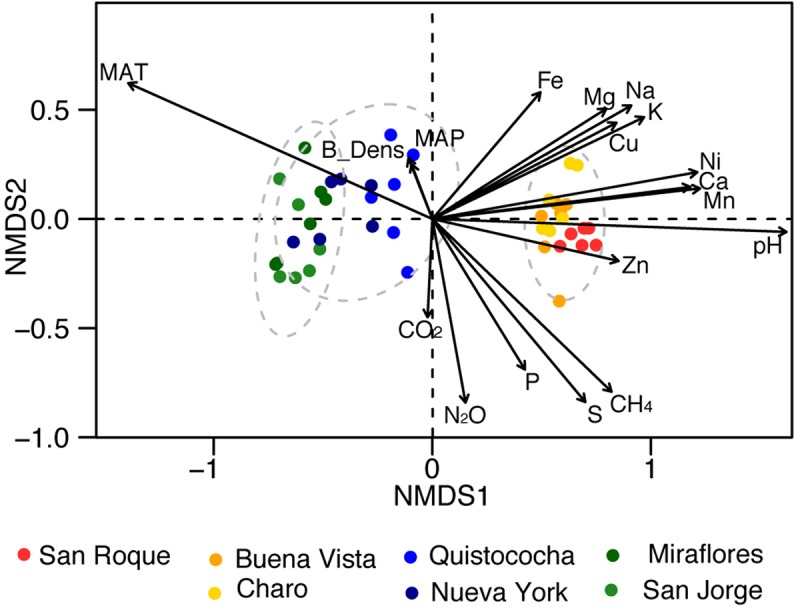
Non-metric multidimensional scaling (NMDS) of microbial community composition of the Pastaza-Marañón Basin peat soils (nodes), and climatological [MAT = mean annual temperature (°C) and MAP = mean annual precipitation (mm)], soil geochemical properties (B_Dens = bulk density, pH, Na, Mg, P, S, K, Ca, Mn, Fe, Ni, Cu, and Zn in units as per Table 1) and gas fluxes (CO_2_, CH_4_, and N_2_O in units as per Figure 1) as loadings. The dotted circles represent 95% confidence intervals of minerotrophic, mixed, and ombrotrophic communities.

Rarefaction of OTUs at the 97% sequence homology and sequencing depth of 50 000 sequences per sample show sufficient sampling was achieved (Supplementary Figure 1) and from the 32 *Bacterial* and *Archaeal* phyla with a relative abundance of greater than 0.005% across the soils over a third (mark with asterisks in Figure 2) differed significantly in their frequency across site’s categories (LSD, *p* < 0.05). Proteobacteria were the dominant phylum fraction across all samples, and were significantly more abundant in minerotrophic and mixed (52 ± 3% and 52 ± 4%, respectively) than ombrotrophic soils (40 ± 5%). The abundance of Acidobacteria differed significantly between the three categories, decreasing from ombrotrophic (32 ± 5%) to minerotrophic (8 ± 2%) as pH increased. Bathyarchaeota abundance was significantly higher in the mixed peatland category (7 ± 1%) than either minerotrophic or ombrotrophic (3 ± 1% and 2 ± 1%, respectively). The low abundance phyla Bacteroidetes, Chloroflexi, Chlorobi, Gemmatimonadaetes, Nitrospirae, and Spirochaetae were significantly higher in minerotrophic peats. Meanwhile Planctomycetes, Thaumarchaeota, Elusimicrobia, and Omnitrophica were significantly higher in the ombrotrophic. Data from a *Sphagnum*-dominated Northern Peatland soil was included for a broad comparison between Northern and tropical Amazon communities. The primary significant differences in the included northern peatland microbial community to Amazon peatlands were higher abundances of Acidobacteria (52 ± 2%), Verrucomicrobia (8 ± 0%) and TM6 (0.2 ± 0%), and lower abundances of Proteobacteria (23 ± 0%) (Figure 2). Ordination of Amazon peatlands’ microbial communities at the 97% OTU similarity level demonstrated clustering of communities based on geochemical/nutritional categories used in this study (Figure 3), with statistically significant differences (ANOSIM *p* < 0.001). Environmental variables showed pH and elemental nutrient concentration separated all microbial communities along the first NMDS axis. Of the GHG, only CH_4_ flux showed an association with minerotrophic communities.

### Methanogen and Methanotroph Relationships Across the Nutrient Gradient

The values of relative abundances of methanogen and methanotroph families (Table 2) show that type II *Methylocystaceae* represented the most abundant methanotrophs in all peats and were not affected by nutrient content (*p* > 0.05) whereas Type I *Methylococcaceae* were only detectable in the minerotrophic peats. No Verrucomicrobial or anaerobic NC10 methanotrophs were identified at the 97% cut off level among reads. All methanogen families, with the exception of unclassified Methanomicrobia and Methanosarcinales, had highest abundances in the minerotrophic soils. Members of *Methanobacteriaceae* were the most dominant family in the minerotrophic soils and were almost no detectable in the ombrotrophic soils (*p* < 0.001). *Methanoregulaceae*, *Methanosaetaceae*, *Methanosarcinaceae*, *Methanomicrobiaceae* and *Methanocellaceae* all decreased with decreasing nutrient content (*p* < 0.005). Members of GOM Arc I (previously termed ANME-2d but not shown to be methanotrophic) were highly abundant in Charo but otherwise not affected by geochemical and nutrient conditions. Only members of the unclassified Methanomicrobia preferred oligotrophic and ombrotrophic peats to rich minerotrophic peats, however these organisms were in very low relative abundance (0.0006%). The OTUs assigned as unclassified Methanomicrobia, Methanomicrobiales or Methanosarcinales are based on environmental, uncultured 16S rRNA genes in the Silva 128 database that lack a close relative to a characterized methanogen family.

**TABLE 2:** Relative abundances of methanogen (1) and methanotroph (2) families across the Pastaza-Marañón Basin nutrient gradient.

		***Methanobacteriaceae*^1^**	***Methanocellaceae*^1^**	***Methanomicrobiaceae*^1^**	***Methanoregulaceae*^1^**	***Methanospirillaceae*^1^**
San Roque	Minerotrophic	21.3 ± 4^a^	1.16 ± 0.3^a^	1.5 ± 0.9^a^	1.9 ± 0.8^a^	0.02 ± 0.02^a^
Buena Vista		5.1 ± 2^a^	1.11 ± 0.1^a^	2.1 ± 0.1^a^	0.3 ± 0.1^a^	0.02 ± 0.03^a^
Charo		5.5 ± 0.7^a^	0.3 ± 0.1^a^	0.6 ± 0.2^a^	1.1 ± 1^a^	0^a^
Quistococha	Mixed	0.3 ± 0.1^b^	0.4 ± 0.2^b^	0.06 ± 0^b^	0.08 ± 0.07^b^	0^b^
Nueva York		0.09 ± 0^b^	0.4 ± 0.3^b^	0.14 ± 0.1^b^	0.24 ± 0.1^b^	0^b^
Miraflores	Ombrotrophic	0^b^	0.04 ± 0^c^	0.02 ± 0.03^b^	0.02 ± 0.03^b^	0^b^
San Jorge		0.02 ± 0^b^	0.23 ± 0^c^	0^b^	0.2 ± 0.2^b^	0^b^
P value		0.0002***	0.004**	0.0002***	0.003**	0.048*

		**Unclassified Methanomicrobiales^1^**	**GOM Arc I^1^**	***Methanosaetaceae*^1^**	***Methanosarcinaceae*^1^**	**Unclassified Methanosarcinales^1^**

San Roque	Minerotrophic	6.1 ± 5.6^a^	0^a^	10.2 ± 3.1^a^	2.3 ± 1^a^	2.8 ± 1.3^a^
Buena Vista		6.6 ± 5.8^a^	0.03 ± 0.05^a^	2 ± 0.2^a^	2.4 ± 1.1^a^	1.3 ± 0.5^a^
Charo		2.9 ± 5^a^	17.6 ± 28.5^a^	3.2 ± 1.1^a^	0.5 ± 0.2^a^	1.4 ± 0.3^a^
Quistococha	Mixed	0^b^	0^a^	0.08 ± 0^b^	0.2 ± 0.03^b^	2.7 ± 1.9^b^
Nueva York		0^b^	0.02 ± 0.03^a^	0.11 ± 0^b^	0.6 ± 0.3^b^	4 ± 1.4^b^
Miraflores	Ombrotrophic	0^b^	0^a^	0^b^	0.02 ± 0.02^b^	0.4 ± 0.2^c^
San Jorge		0^b^	0^a^	0.05 ± 0.04^b^	0.05 ± 0.04^b^	0.8 ± 1^c^
P value		0.02*	0.44	0.0003***	0.0003***	0.005**

		**Unclassified Methanomicrobia^1^**	***Methylocystaceae*^2^**	***Methylococcaceae*^2^**		

San Roque	Minerotrophic	0^a^	1.2 ± 0.3^a^	0.08 ± 0.03^a^		
Buena Vista		0^a^	0.7 ± 0.3^a^	0.3 ± 0.05^a^		
Charo		0^a^	0.7 ± 0.3^a^	1.7 ± 0.06^a^		
Quistococha	Mixed	8.3 ± 9.2^b^	0.3 ± 0.1^a^	0^b^		
Nueva York		5.9 ± 2.7^b^	0.1 ± 0.08^a^	0^b^		
Miraflores	Ombrotrophic	1.1 ± 1.8^c^	0.4 ± 0.3^a^	0^b^		
San Jorge		5 ± 3.3^c^	0.5 ± 0.1^a^	0^b^		
P value		0.001**	0.16	0.0002***		

*All values are expressed as relative abundance (%) at 10^–3^, with the exception of Unclassifed Methanomicrobiales and Methanomicrobia, which are expressed as relative abundance (%) at 10^–6^. *P* values are results of Kruskal-Wallis tests of abundances across peatland categories. Averages and standard deviations represent six independent measurements.*

**FIGURE 4:**
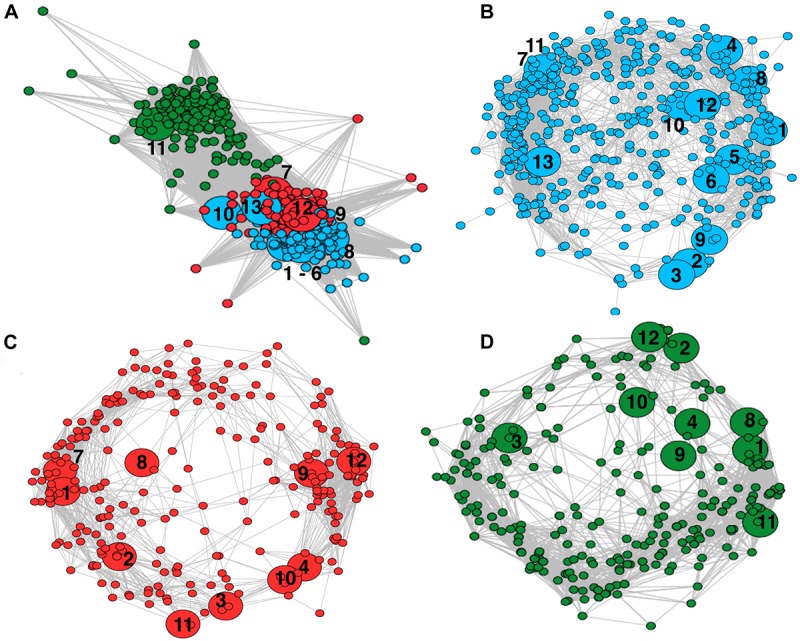
Weighted network analyses of OTU covariance across Pastaza-Marañón Basin peat soils. Nodes represent individual OTUs grouped at the family level. Edges represent significantly covarying OTUs (*p* < 0.05). Methanogen and methanotroph nodes have been enlarged relative to other nodes, and are labeled at the family level. The numbering scheme is as follows: *Methanobacteriaceae* (1); *Methanocellaceae* (2); *Methanomicrobiaceae* (3); *Methanoregulaceae* (4); *Methanospirillaceae* (5); Uncultured Methanomicrobiales (6); GOM Arc I (7); *Methanosaetaceae* (8); *Methanosarcinaceae* (9); Uncultured Methanosarcinales (10); Uncultured Methanomicrobia (11); *Methylocystaceae* (12); *Methylococcaceae* (13). **(A)** network of all peatland communities. Nodes are colored by modularity calculated with the Spinglass technique, Module One (blue), Module Two (red), and Module Three (green). Most methanogen and *Methylococcaceae* aggregated in Module One, with *Methylocystaceae* in Two and Uncultured Methanomicrobia in Three. **(B)** A network of the minerotrophic sites (San Roque, Buena Vista and Charo). **(C)** A network of the mixed sites (Quistococha and Nueva York). **(D)** A network of the ombrotrophic sites (Miraflores and San Jorge).

**FIGURE 5:**
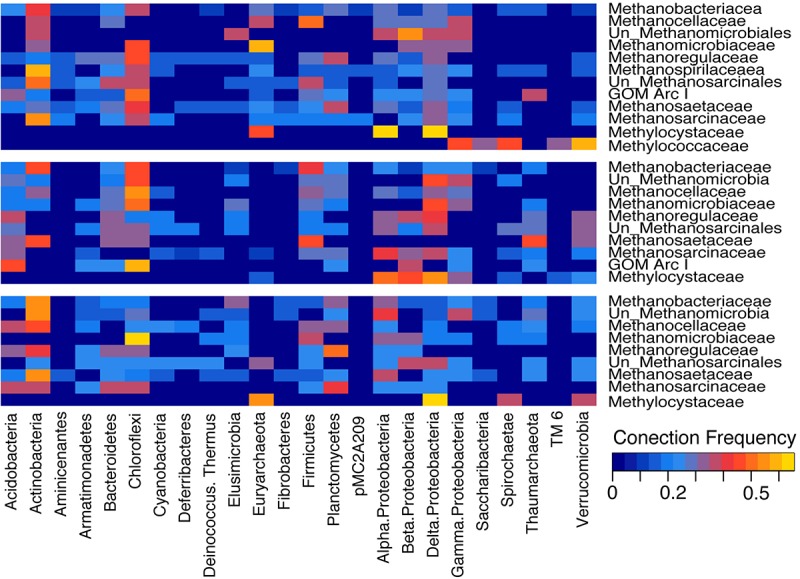
Heatmap of connection frequencies between methanogen and methanotroph nodes to significantly covarying nodes (*p* < 0.05) from minerotrophic (blue), mixed (red), and ombrotrophic (green) networks. Covarying nodes on the y axis are reported at the Phylum level. Scaled frequencies range from zero (dark blue) to 0.7 (yellow). Units are Hellinger transformed discrete counts of node connections.

Covariance network analysis of microbial families across peats showed three separate modules (Figure 4A). The abundant methanogens in the minerotrophic sites all clustered in Module One (blue). The Unclassified Methanomicrobia grouped with Module Three (green). Methanotrophic *Methylococcacae*, only identified in the minerotrophic soils, grouped with Module One. The more evenly dispersed *Methylocystaceae* grouped with Module Two (red), which was intermediate between Modules One and Three. The analysis of individual networks for minerotrophic, mixed and ombrotrophic communities (Figures 4B–D), showed that the total number of nodes (i.e., taxon families) decreased along the gradient (totals of 495, 327, and 301 in the minerotrophic, mixed and ombrotrophic networks, respectively). The variance to mean ratio (VMR) of node connectivity also decreased along the gradient as the number of highly connected, central nodes dispersed (VMRs of 0.81, 0.66, and 0.54 for minerotrophic, mixed and ombrotrophic networks, respectively). Finally, mean path length also decreased as nodes became more dispersed (lengths of 5.88, 2.95, and 2.81 for minerotrophic, mixed and ombrotrophic networks, respectively). A heatmap analysis of the connection frequency between methanogen/methanotroph nodes to significantly covarying nodes in individual networks (Figure 5), shows that methanogen nodes were most often connected to Actinobacteria, Chloroflexi, Firmicutes, Planctomycetes, Alpha- and Delta-Proteobacteria across all categories, and to Bacteroidetes, Thaumarchaeota and Verrucomicrobia and other Euryarchaeota to a lesser degree. Connections to Delta-Proteobacteria were primarily to sulfate reducers (*Desulfaculaceae*, *Desulfobacteraceae*, *Desulfurellaceae*, *Desulfovibrionaceae*) and the family *Syntrophorhabdaceae* (Supplementary Table 2). Connections to Alpha-Proteobacteria were between methanogens and methanotrophic *Methylocystaceae*, *Rhizobiaceae*, and *Sphingomonads* (Supplementary Table 2). *Methylocystaceae* methanotrophs were predominantly connected to varied Alpha-, Beta-, Delta- and Gamma-Proteobacteria. *Methylococcaceae* showed sparse connections to other Gamma-Proteobacteria, Saccharibacteria, Spirochaetae, Tectomicrobia, TM6, and Verrucomicrobia in the minerotrophic network (Figure 5).

**FIGURE 6:**
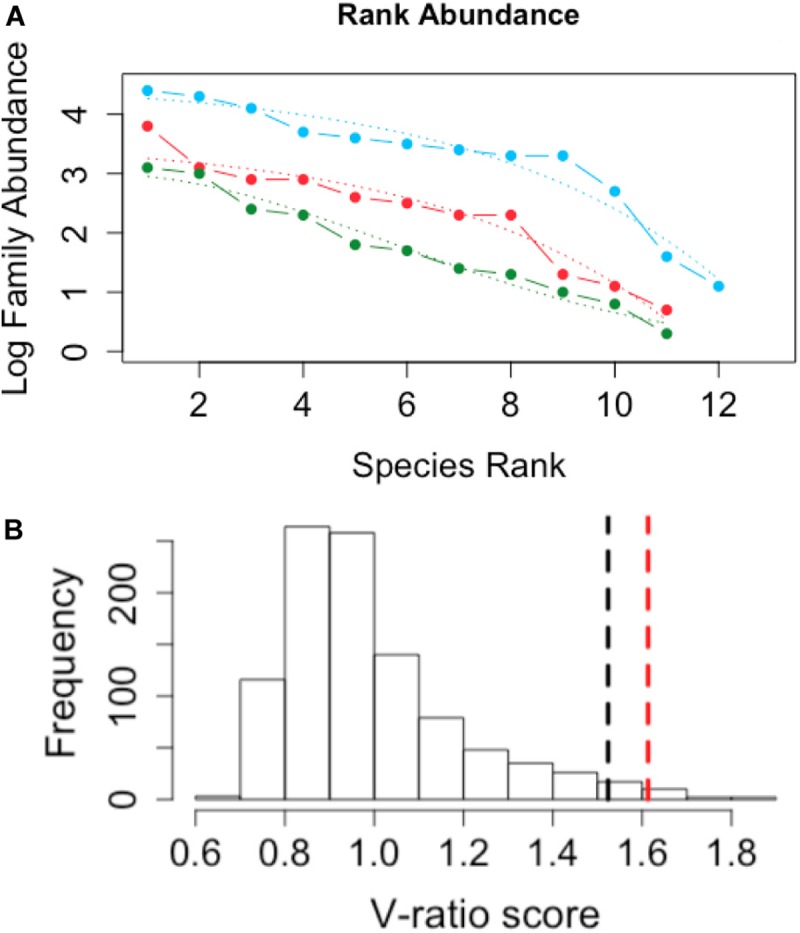
**(A)** Species Rank abundance for minerotrophic (blue), mixed (red), and ombrotrophic (green) methanogen and methanotroph families. Dotted lines indicate geometric (minerotrophic and mixed) and log-normal model (ombrotrophic) fits for communities from the three peatland categories. **(B)** Histogram of the distribution of null species co-occurrence, measured as the V-ratio, with a randomized null model. The black dotted line indicates the two-tailed upper 95% tail. The red dotted line indicates the observed V-ratio of methanogen and methanotroph families. The null model was simulated with 1000 replications.

Rank abundance curves of methanogen and methanotroph families (Figure 6A) and AIC model comparisons demonstrated geometric distributions (i.e., a poorly structured community) for minerotrophic (blue) (R^2^ = 0.95) and mixed (red) (R^2^ = 0.93) communities, whereas the ombrotrophic community had a log-normal distribution (green) (R^2^ = 0.97) indicative of a highly structured community. Finally, the V-ratio of co-occurrence of methanotroph and methanogen families was 1.6131, which was significantly greater than the upper 95% tail of 1000 permutations of the family matrix (mean of 0.99, standard deviation of 0.04, Figure 6B.).

## Discussion

### Variation in CH_4_ Flux Across the Nutrient Gradient

Methane fluxes from tropical wetlands can vary greatly, from atmospheric uptake to 30 mg CH_4_-C m^2^ h^–1^, indicative of the underlying complexities that contribute to CH_4_ flux (Bartlett and Harriss, 1993; Sjogersten et al., 2014; Zhu et al., 2015). Both flight-derived measurements above the Amazon Basin and ground-level field-scale measurements agree that regional differences arising from vegetation type, hydrology and soil pH are important contributors to CH_4_ flux (Wilson et al., 2016; Teh et al., 2017; Winton et al., 2017). Consistently greater fluxes occur from high-productivity vegetation and nutrient rich soils types that experience seasonal flooding (minerotrophic), compared to rainfall-fed (ombrotrophic) poor and low-productivity vegetation types, which emphasizes the complex interactions between seasonality, hydrology and biological processes in the Amazon Basin (Lahteenoja et al., 2009; Teh et al., 2017).

The measurements recorded here, of 0.3 to 2.5 mg CH_4_-C m^2^ h^–1^, are in similar range to measurements previously collected in diverse wetlands and flooded areas in the Amazon basin, 0.08 to 6 mg CH_4_-C m^2^ h^–1^ (Bartlett et al., 1988; Richey et al., 1988; Bartlett et al., 1990; Miller et al., 2007; Belger et al., 2011). However, CH_4_ values in this study are in a lower range than those observed in the only other multisite measurements completed in peatlands of Pastaza-Marañón Basin for their dry season measurements (1.8–31 mg CH_4_-C m^2^ h^–1^) (Teh et al., 2017). Nevertheless, the opposite was observed in N_2_O flux rates in comparison to that same study (0.5–100 vs. ∼0.03–0.87 ug N_2_O-N m^2^ h^–1^ respectively) (Teh et al., 2017). The observed differences between studies are likely explained by the different sampling design, inclusion or not of temporal component in either study, and methodological differences in gas collection indicating further studies, common methodologies and common benchmarks are still needed to better understand CH_4_ flux variation in the Amazon and develop more accurate regional estimates. Our study did not aim to record seasonal variation which can significantly change CH_4_ flux in the Pastaza-Marañón peatlands (Teh et al., 2017), but focused on differences across sites in the same season with flux sampling during similar times of day (±3 h around midday) to reduce potential effects of day fluctuations. We found a strong relationship between CH_4_ flux, nutrient content and pH (LDS test Table 1, and Kruskal-Wallis, *p* = 0.03) similar to general findings of other studies in both Northern and tropical peatlands (Bartlett and Harriss, 1993; Lai, 2009; Ye et al., 2012; Teh et al., 2017; Winton et al., 2017). The minerotrophic Charo site proved an exception to this, as CH_4_ fluxes from this site mirrored the mixed and ombrotrophic sites. High concentrations of Fe (356 ± 90 g kg^–1^ dry soil) and Mn (411 ± 73.8 mg kg^–1^ dry soil) were present at Charo. In fact, supplementary water chemistry assessments also showed that the concentration of sulfate was also high at Charo compared to the other peatland sites (16.4 ± 9.3 mg L^–1^ *versus* 0.1 – 1.67 mg L^–1^, Supplementary Table 1). As microbial Fe (III), Mn and sulfate reduction are more energetically favorable than methanogenesis (Dubey, 2005) the low CH_4_ flux observed at Charo is explained by the occurrence of microbial respiration based on alternative terminal electron acceptor for anaerobic respiration (like Fe, Mn, SO_4_) which are more energetically favorable and outcompete methanogens for several substrates (acetate, formate, etc.) (Bridgham et al., 2013). This was not observed in the other two minerotrophic sites and underscore the importance of evaluating the geochemistry of amazon peatlands as a control of methanogenesis.

### Composition of Microbial Phyla Across the Nutrient Gradient

In terms of overall community composition, the dominant Proteobacteria decreased from minerotrophic to ombrotrophic sites, counter mirrored by an increase in the fraction of Acidobacteria in the same gradient. A dominance of Proteobacteria in near-neutral, minerotrophic Northern (Zhou et al., 2017) and poor (pH 3.8) tropical peatlands has been reported in previous community surveys (Espenberg et al., 2018). Similarly, a more dramatic shift in the community favoring Acidobacteria has been found in acidic, nutrient-poor ombrotrophic Northern and tropical peatlands (Jackson et al., 2009; Lin et al., 2012; Lin et al., 2014), and is apparent in the Northern peatland included here for general comparison (pH ∼ 3.5). Bacteroidetes, Firmicutes, Actinobacteria and Chloroflexi represent secondarily dominant phyla in Northern and tropical peatlands, as observed here. Unique to this study was to note relatively high abundances of Bathyarchaeota (up to 8% in the mixed) and Thaumarchaeota (up to 5% in the ombrotrophic peats). As the Bathyarchaeota have only recently been reclassified and incorporated into 16S rRNA gene reference databases, their distribution in terrestrial environments is still not yet fully accounted, but appear to dominate *Archaeal* communities in some peat, freshwater wetland and mangrove sediments (Xiang et al., 2017). In the comparison to reads from a Northern peatland site incorporated here, Bathyarchaeota were detectable at a mean relative abundance of 0.006%, suggesting a preference for tropical peatlands. However, since Northern peatlands have been shown to hold significant variation in microbial diversity, a more comprehensive comparison against more sites is required to better assess Bathyarchaeota or other group’s preferences. Thaumarchaeota have recently become of great interest as certain isolates are involved in aerobic ammonia oxidation (Brochier-Armanet et al., 2012). These *Archaea* have also been shown to form macroscopic filaments attached to surfaces in tropical mangrove sediments, that support symbiotic growth of sulfur reducing Proteobacteria (Muller et al., 2010). The environmental role of Bathyarchaeota and Thaumarchaeota in these tropical peatlands remains to be determined.

### Methanogen and Methanotroph Family Assemblages Across the Gradient

Methane flux from peatlands is a combined effect of *in situ* production *versus* consumption due to the activity of methanogens and methanotrophs, respectively. Methanogenic activity is further constrained by the supply of methanogenic substrates from heterotrophic activity or competition for methanogenic substrate consumption when alternative terminal electron acceptors are available in the ecosystem (Garcia et al., 2000). These two factors make methanogens sensitive to nutrient conditions affecting heterotrophs or competitors, while aerobic methanotrophs can be effected by levels of CH_4_ or competition for O_2_ as a shared electron acceptor with other aerobic heterotrophs.

Here we use the relative abundance of a taxonomic unit as a proxy for an organism’s fitness under certain conditions. Relative abundance is a reflection of the capacity of an organism to out-compete others for control of a limiting resource (Pielou, 1975). Such competition gives rise to highly structured, non-uniform species assemblages whereby each species can be considered as occupying a distinct niche (Sugihara, 1980). We sought to investigate how the environment and potential microbial taxa interactions affect methanogenic and methanotrophic communities and whether changes in community assembly were associated with CH_4_ flux. While *de novo* genome assemblies suggest that members of the Bathyarchaeota may be capable of methanogenesis (Evans et al., 2015), other studies have demonstrated acetogenic (He et al., 2016) and heterotrophic (Maus et al., 2018; Yu et al., 2018) metabolism by this phyla, and so this taxonomic group was not included in ecological analyses as methanogens. Furthermore, while GOM Arc I were initially termed ANME-2d, this group is neither monophyletic with other ANME groups nor has it been shown to consume CH_4_ (Lloyd et al., 2006) and so GOM Arc I were considered as methanogens here.

Interestingly, optimal modularity of a network of all peatlands in study agreed with their *a priori* categorization into three categories. By separating this network based on water geochemistry/nutrient categories and constructing sub-networks, it was clear that connectivity within networks decreased as nodes generally became less central with decreasing mineral levels from minerotrophic to ombrotrophic. From these sub-networks it was possible to gauge the frequency of connections between methanogens/methanotrophs and other phyla. Methanogens were most commonly associated with anaerobic sulfate reducing Delta-Proteobacteria, methanotrophic Alpha-Proteobacterial *Methylocystaceae*, Actinobacteria, Firmicutes and Chloroflexi. Actinobacteria and Firmicutes include many primary and secondary fermenters (including known syntrophs) sustaining methanogens as shown in northern peatlands (Drake et al., 2009; Wust et al., 2009). The positive covariance between competitive methanogens and sulfate reducers was surprising. However, sulfate reducing Delta-Proteobacteria have been shown to be predominant members of peatland microflora previously (Morales et al., 2006; Lin et al., 2014; Zhou et al., 2017), while it has also been observed that certain hydrogenotrophic methanogens, such as *Methanobacteriaceae* and Methanomicrobia that dominated these systems, can coexist *in vitro* and generate CH_4_ in a stable manner while in co-culture with sulfate reducers (Chen et al., 2018). Indeed, as sulfate reducers can utilize propionate, butyrate, lactate and acetate as alternative electron donors to hydrogen, the potential for coexistence between sulfate reducers and methanogens has been noted for some time (Bryant et al., 1977; Muyzer and Stams, 2008). A relationship between methanogens and methanotrophic *Methylocystaceae* was not surprising, although the absence of an interaction with *Methylococcaceae* was. This may be a result of differences in methanotroph ecophysiology, explained in greater detail below. Actinobacteria play a vital role in the catabolism of plant-derived organic matter, important for the initial stages of decomposition (Goodfellow and Williams, 1983; Bentley et al., 2002; Berlemont and Martiny, 2013). The phylum Firmicutes includes many well-characterized heterotrophs responsible for producing partially fermented organic compounds suitable for methanogens, and have been noted to positively covary with methanogens when enriched in batch culture (Sierocinski et al., 2018). In fact, primary and secondary fermenters in Actinobacteria and Firmicutes have been shown sustaining methanogenic metabolism in northern peatland soils (Drake et al., 2009; Wust et al., 2009). Finally, while Chloroflexi are less understood, *in silico* analyses suggest that this phyla is also involved in the anaerobic degradation of plant-derived organic matter (Hug et al., 2013). Thus, these taxa in consort with methanogens may play an important role in the synergistic degradation of plant-derived organic matter and CH_4_ cycling in these peatlands. The methanotrophs primarily demonstrated associations with other Proteobacteria, particularly with the more dominant and evenly dispersed *Methylocystaceae*. This may imply some level of metabolic codependence between Proteobacterial taxa (Morris et al., 2012; Sachs and Hollowell, 2012), which has been demonstrated in co-cultures of methanotrophs and heterotrophic Proteobacteria (Iguchi et al., 2011; Ho et al., 2014; Jeong and Kim, 2018). Also, methanotrophs produce methanol as part of their metabolism and it has been shown they could accumulate it externally upon inhibition of their methanol dehydrogenase (Patel et al., 2016), and putatively could support methylotrophic Proteobacteria. Alternatively, this effect may simply be a product of strong covariance between highly dominant Proteobacteria.

The modularity visualized with the network were supported further as either geometric (minerotrophic and mixed) or log-normal (ombrotrophic) rank distributions (Figure 6A). A log-normal rank distribution is reflective of highly structured, hierarchical communities whereby taxonomic units are separated based on niche differentiation (Preston, 1962) whereas a geometric distribution is reflective of poorly even distributions whereby few taxa of the highest ranks are highly competitive and monopolize available resources at the expense of others (Ludwig and Reynolds, 1988). Also, the null hypothesis of an absence of structured assemblages was rejected based on the V-ratio of methanogen and methanotroph family distributions (*p* < 0.05, Figure 6B). A V-ratio significantly greater than one indicates aggregation of taxa assemblages (Gotelli, 2000), which was driven by aggregations of methanogen families and Type I *Methylococcaceae* methanotrophs in the minerotrophic systems. Taken together, all these evidences support a structured, niche-dependent ecology of methanogens and Type I *Methylococcaceae* in the Pastaza-Marañón Basin.

### Ecophysiology of Methanogen and Methanotroph Families

It has been shown previously that methanogen species richness is higher in minerotrophic *versus* ombrotrophic northern peatlands (Juottonen et al., 2005; Lin et al., 2012). There are likely multiple underlying reasons for this, and for the structured community assemblages in the Pastaza-Marañón Basin. Plant net primary productivity is highest in minerotrophic peatlands relative to others, and higher quantities of plant root exudates can provide potential methanogenic substrates to communities (Whiting and Chanton, 1993). The ombrotrophic peats of this study (Miraflores and San Jorge) are dominated by ‘pole forest’ species (Draper et al., 2014) which are expected to provide more phenolic-like compounds to the peat. Additions of Fe, Ni, Co, and Na have an immediate, positive effect on CH_4_ production in peatland soils, as these metals can be limiting cofactors for enzymes involved in microbial fermentation and/or methanogenesis (Basiliko and Yavitt, 2001). Finally, methanogenic activity is highly sensitive to pH and both hydrogenotrophic and acetoclastic methanogenesis decreases if the pH is less than 4.5 (Ye et al., 2012).

While the majority of methanogens favored the minerotrophic peats, the abundances of methanogen families differed. The most dominant families in the minerotrophic peatlands were *Methanobacteriaceae* and *Methanosataceae*. *Methanobacteriaceae* are hydrogenotrophic methanogens that typically dominate minerotrophic northern peats (Juottonen et al., 2005; Steinberg and Regan, 2008; Lin et al., 2012). Hydrogenotrophic methanogenesis is more energetically favorable than acetoclastic methanogenesis (−131 vs. −31 ΔG°’ kJ mol^–1^ CH_4_, respectively) (Garcia et al., 2000; Thauer et al., 2008). This could explain the dominance of the methanogen population by *Methanobacteriaceae* relative to acetoclastic *Methanosataceae*. Simultaneously, the greater abundance of acetoclastic *Methanosataceae* relative to other hydrogenotrophs could be as it occupies a separate, substrate-dependent niche to the most successful hydrogenotroph, *Methanobacteriaceae*.

The distributions of *Methanobacteriaceae* and *Methanosataceae* were primarily constrained to the minerotrophic peatlands. In the mixed and ombrotrophic environments, the dominant hydrogenotrophic and acetoclastic methanogens were Unclassified Methanomicrobia and Unclassified Methanosarcinales, respectively. The Class Methanomicrobia includes a wide range of Orders, such as Methanocellales, Methanomicrobiales and Methanosarcinales. That these OTUs could not be classified to the Family level suggests there are novel, uncultured methanogens in these mixed and ombrotrophic tropical peatlands. A number of traits in these groups may be favored in such environments. For example, Methanocellales may be uniquely equipped for nutrient-poor, low productivity environments, as they effectively utilize hydrogen under partial pressures reaching the thermodynamic limit of methanogenesis, and dinitrogen-fixation pathways have been identified in several genomes (Lyu and Lu, 2015). Methanosarcinales are metabolically diverse and demonstrate greater growth yield efficiency than hydrogenotrophic methanogens (7.2 vs. up to 3 g biomass mol^–1^ CH_4_, respectively) (Thauer et al., 2008).

Similarly to methanogens, methanotroph families demonstrated non-uniform distributions. *Methylocystaceae* dominance is consistent with observations in northern peatlands (Wieczorek et al., 2011;Andersen et al., 2013) and a forested swamp peatland in Malaysia (Too et al., 2018), and its abundance was unaffected by nutrient content or the relatively low CH_4_ fluxes from oligotrophic and ombrotrophic peats. Unlike *Methylococcacae*, *Methylocystaceae* can utilize carbon sources other than CH_4_ such as acetate, oxidize CH_4_ at relatively low oxygen concentrations, and can survive periods of anoxia via storing carbon intracellularly as poly beta-hydroxybutyrate (Vecherskaya et al., 2001; Semrau et al., 2011). As with Methanocellales, these traits allow *Methylocystaceae* to dominate nutrient-poor environments. Conversely, *Methylococcaceae* methanotrophs tend to occupy nutrient-rich environmental niches and are dependent on relatively higher concentrations of CH_4_ (Whittenbury et al., 1970). ANME or NC10 methanotrophs were not identified in this study at analyzed peat depths (0 – 30 cm), which could be explained by absence or low density at this water-saturated shallow depths. One study targeting deeper layers (80 – 135 cm) identified anaerobic methanotrophs in a northern peatland (Zhu et al., 2012), while others identified similar groups in shallow layers (top 15 cm) in wetlands (Segarra et al., 2015;Valenzuela et al., 2017). Since this study, did not target deeper layers to account for anaerobic methanotrophs then the role of nutrient concentration on below-surface anaerobic methanotroph communities, if any, is not addressed by the field sampling design used here.

### Relevance for Regional CH_4_ Fluxes in the Amazon Basin

As has been shown for northern peatlands, our results show a putative coupling between peat geochemical and nutrient content and CH_4_ flux (Whiting and Chanton, 1993). This has implications both for improvement of global CH_4_ biogeochemical models and can inform environmental regulations concerning peatlands. Although tropical peatlands are known to be fundamental players in global CH_4_ cycling, there is a concerning lack of empirical data to support GHG modeling (Sjogersten et al., 2014). Targeted measurements based on historical knowledge of nutrient status and hydrological patterns may assist in continent scale modeling of CH_4_ flux. Furthermore, anthropological disturbance of peatlands has consequences for ecosystem scale C and nutrient cycling (Andersen et al., 2013). As nutrient status could be key for regulating microbial communities involved in CH_4_ flux, changes to hydrological regimes and agricultural nutrient deposition has the potential to increase atmospheric CH_4_ emissions from tropical peatlands.

## Conclusion

In conclusion, nutrient concentration shapes microbial communities in the largest tropical peatland in South America, the Pastaza-Marañón Basin. Highly structured, niche-dependent distributions of methanogen and methanotroph families were found to exist, dependent on nutrient concentration. Methanogen communities, dominated by hydrogenotrophic *Methanobacteriaceae*, aggregate strongly with minerotrophic systems that display higher CH_4_ flux. This suggests that hydrogenotrophic methanogenesis is the primary source of CH_4_ in these minerotrophic systems. Methanotroph communities, dominated by *Methylocystaceae*, are dispersed across all systems regardless of CH_4_ flux, pH and nutrient content. The putative association between CH_4_ flux and peatland geochemical and nutrient content can inform modeling of the CH_4_ cycle in the tropics and suggest increased atmospheric CH_4_ emissions as a consequence of hydrology disturbance and agricultural nutrient deposition.

## Data Availability Statement

The 16S rRNA sequence datasets generated for this study can be found in the NCBI repository under the SRA Bioproject ID PRJNA501909 (https://www.ncbi.nlm.nih.gov/sra/SRX4961727).

## Author Contributions

HC-Q designed the study. HC-Q, DF, MZ-E, JP, JH, JA-P, and JU-M conducted the sample collection, gas, chemical, and molecular analyses. DF and MZ-E equally performed the data analyses, modeling and synthesis, and drafted the manuscript. All authors contributed to the final version of the manuscript.

## Conflict of Interest

The authors declare that the research was conducted in the absence of any commercial or financial relationships that could be construed as a potential conflict of interest.
