# Supplementary material for: Methanogens and Methanotrophs Show Nutrient-Dependent Community Assemblage Patterns Across Tropical Peatlands of the Pastaza-Marañón Basin, Peruvian Amazonia
Source: Front Microbiol. 2020 Apr 24;11:746. doi: 10.3389/fmicb.2020.00746 (PMC7193774; doi:10.3389/fmicb.2020.00746)
Supplement: Supplementary file 1 [file Data_Sheet_1.docx]

Supplementary Material

# Supplementary Figures and Tables


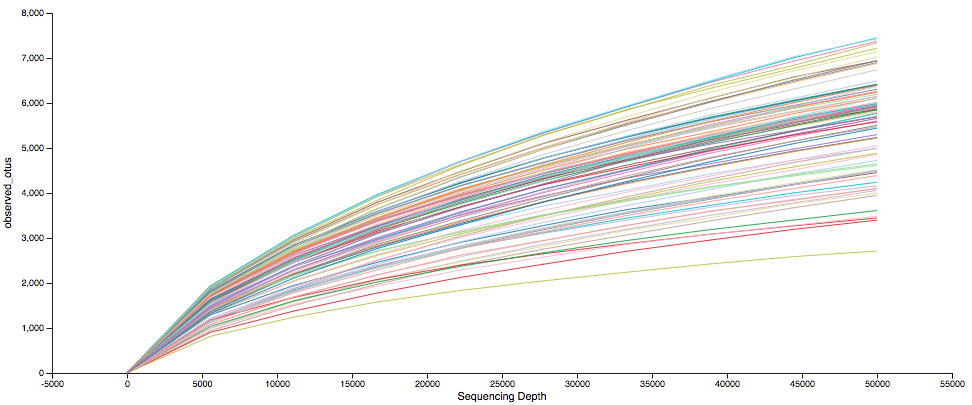


Supplementary Figure 1: Rarefaction analysis of sequences at 97% identity utilized in this study. All samples were rarefied using QIIME2 tools to show the distribution of putative OTU’s at different sampling intensity. All samples (colored lines) in general show a somewhat similar inflection point of their slope after 5000 reads per sample and that the majority of samples show a range of OTUs fluctuating from ~2000 to ~7000 OTU at around 50,000 reads per sample. Based on similarity of slope at near 50,000 reads we considered that a similar sampling effort was achieved across samples for comparisons.

Supplementary Table 1: Dissolved organic C (DOC), inorganic C (DIC) and anion/cation measurements for several of the tropical peatland sites analyzed in this study. Measurements are in mg L^-1^. Reported are the mean ± standard deviation of three measurements.

| Soil | DOC | DIC | Nitrite | Nitrate | Ammonium | Sulphate | Phosphate | Sodium |
| --- | --- | --- | --- | --- | --- | --- | --- | --- |
| San Roque | 21.4 ± 8.7 | 1 ± 0.2 | 0.14 ± 0.1 | 1.82 ± 1.8 | 0.22 ± 0.2 | 0.11 ± 0.1 | 0 | 2.65 ± 0.2 |
| Charo | 48 ± 5.7 | 3.6 ± 0.8 | 0.03 ± 0.04 | 1.2 ± 0.7 | 0.44 ± 0.2 | 16.4 ± 9.3 | 0 | 4.2 ± 0.8 |
| Buena Vista | NA | NA | NA | NA | NA | 0.67 ± 0.3 | NA | NA |
| Quistococha | 14.06 ± 12.9 | 0.44 ± 0.6 | BDT | 10.4 ± 15.8 | 0.32 ± 0.2 | 1.46 ± 0.2 | 0.16 ± 0.2 | 2.01 ± 0.2 |
| San Jorge | 36.2 ± 31.3 | 1.63 ± 0.7 | BDT | 0.71 ± 0.3 | 0.12 ± 0 | 0.11 ± 0.1 | 0.03 ± 0 | 0.31 ± 0 |
| Miraflores | 88.8 ± 9.7 | 0.74 ± 0.2 | BDT | 0.2 ± 0.1 | 0.15 ± 0.1 | 0.1 ± 0.1 | 0.2 ± 0.1 | 0.2 ± 0 |

DOC, DIC and anion/cation methodologies: Three water samples per site were collected from the upper 5 cm of the water table. Water was immediately filtered first through a 0.8 um pore diameter filter, and secondly by a 0.22 um pore diameter filter for ions, or a 0.45 um pore diameter filter for dissolved organic/inorganic carbon. Samples were stored in Nalgene bottles that contained hydrochloric acid. Samples were frozen within 24 hours of collection. Ion chromatography for anions and cations was performed with Dionex IonPac AS11 and IonPac AG11 guard columns (anions) and Dionex IonPac CS12A and IonPac SG11 guard columns (cations) as per the manufacturer’s instructions. Dissolved carbon measurements were performed with a TOC-VCSH analyzer equipped with combustion catalytic oxidation/non-dispersive infrared gas analyzer (Shimadzu Scientific Instruments, Columbia, MD) following method 5310B in Standard Methods (American Public Health Association, 2005. Standard Methods for the examination of water and wastewater. Washington DC: APHA, AWWA, WEF). BDT= below detection limit (~0.005 mg L^-1^ for Nitrite)

Supplementary Table 2: Edgelist of Families connected to methanogen and methanotroph nodes across networks.

| Network | Node A | Node B |
| --- | --- | --- |
| Minerotrophic | D_0__Archaea;D_1__Euryarchaeota;D_2__Methanomicrobia;D_3__Methanomicrobiales;D_4__Methanomicrobiaceae | D_0__Bacteria;D_1__CPR2;__;__;__ |
| Minerotrophic | D_0__Archaea;D_1__Euryarchaeota;D_2__Methanomicrobia;D_3__Methanomicrobiales;D_4__Methanospirillaceae | D_0__Archaea;D_1__Thaumarchaeota;__;__;__ |
| Minerotrophic | D_0__Archaea;D_1__Euryarchaeota;D_2__Methanobacteria;D_3__Methanobacteriales;D_4__Methanobacteriaceae | D_0__Bacteria;D_1__Proteobacteria;D_2__Deltaproteobacteria;D_3__Deltaproteobacteria Incertae Sedis;D_4__Syntrophorhabdaceae |
| Minerotrophic | D_0__Archaea;D_1__Euryarchaeota;D_2__Methanobacteria;D_3__Methanobacteriales;D_4__Methanobacteriaceae | D_0__Bacteria;D_1__Bacteroidetes;D_2__Bacteroidetes vadinHA17;__;__ |
| Minerotrophic | D_0__Archaea;D_1__Euryarchaeota;D_2__Methanomicrobia;D_3__Methanomicrobiales;D_4__Methanoregulaceae | D_0__Bacteria;D_1__Actinobacteria;D_2__Actinobacteria;D_3__Frankiales;D_4__Sporichthyaceae |
| Minerotrophic | D_0__Archaea;D_1__Euryarchaeota;D_2__Methanomicrobia;D_3__Methanomicrobiales;D_4__Methanoregulaceae | D_0__Bacteria;D_1__Deferribacteres;D_2__Deferribacteres Incertae Sedis;D_3__Unknown Order;D_4__Unknown Family |
| Minerotrophic | D_0__Archaea;D_1__Euryarchaeota;D_2__Methanomicrobia;D_3__Methanosarcinales;__ | D_0__Bacteria;D_1__Actinobacteria;D_2__Thermoleophilia;D_3__Solirubrobacterales;D_4__Elev-16S-1332 |
| Minerotrophic | D_0__Archaea;D_1__Euryarchaeota;D_2__Methanomicrobia;D_3__Methanosarcinales;D_4__GOM Arc I | D_0__Bacteria;D_1__Planctomycetes;D_2__Phycisphaerae;D_3__ODP1230B30.09;__ |
| Minerotrophic | D_0__Archaea;D_1__Euryarchaeota;D_2__Methanobacteria;D_3__Methanobacteriales;D_4__Methanobacteriaceae | D_0__Bacteria;D_1__Chloroflexi;D_2__Dehalococcoidia;D_3__vadinBA26;__ |
| Minerotrophic | D_0__Bacteria;D_1__Cyanobacteria;D_2__Melainabacteria;D_3__Vampirovibrionales;D_4__uncultured bacterium | D_0__Bacteria;D_1__Proteobacteria;D_2__Gammaproteobacteria;D_3__Methylococcales;D_4__Methylococcaceae |
| Minerotrophic | D_0__Archaea;D_1__Euryarchaeota;D_2__Methanomicrobia;D_3__Methanomicrobiales;__ | D_0__Bacteria;D_1__Proteobacteria;D_2__Betaproteobacteria;D_3__Rhodocyclales;D_4__Rhodocyclaceae |
| Minerotrophic | D_0__Bacteria;D_1__Planctomycetes;D_2__Phycisphaerae;D_3__CCM11a;D_4__uncultured bacterium | D_0__Bacteria;D_1__Proteobacteria;D_2__Gammaproteobacteria;D_3__Methylococcales;D_4__Methylococcaceae |
| Minerotrophic | D_0__Bacteria;D_1__Acidobacteria;D_2__Subgroup 13;__;__ | D_0__Bacteria;D_1__Proteobacteria;D_2__Gammaproteobacteria;D_3__Methylococcales;D_4__Methylococcaceae |
| Minerotrophic | D_0__Archaea;D_1__Euryarchaeota;D_2__Methanomicrobia;D_3__Methanomicrobiales;__ | D_0__Bacteria;D_1__Proteobacteria;D_2__Alphaproteobacteria;D_3__Caulobacterales;D_4__Caulobacteraceae |
| Minerotrophic | D_0__Archaea;D_1__Euryarchaeota;D_2__Methanomicrobia;D_3__Methanomicrobiales;D_4__Methanospirillaceae | D_0__Bacteria;D_1__Planctomycetes;D_2__Phycisphaerae;D_3__Pla1 lineage;__ |
| Minerotrophic | D_0__Archaea;D_1__Euryarchaeota;D_2__Methanomicrobia;D_3__Methanosarcinales;__ | D_0__Archaea;D_1__Euryarchaeota;D_2__Thermoplasmata;D_3__Thermoplasmatales;D_4__Marine Benthic Group D and DHVEG-1 |
| Minerotrophic | D_0__Bacteria;D_1__Planctomycetes;D_2__Phycisphaerae;D_3__mle1-8;__ | D_0__Bacteria;D_1__Proteobacteria;D_2__Alphaproteobacteria;D_3__Rhizobiales;D_4__Methylocystaceae |
| Minerotrophic | D_0__Archaea;D_1__Euryarchaeota;D_2__Methanomicrobia;D_3__Methanosarcinales;D_4__GOM Arc I | D_0__Bacteria;D_1__Chloroflexi;D_2__TK10;__;__ |
| Minerotrophic | D_0__Archaea;D_1__Euryarchaeota;D_2__Methanomicrobia;D_3__Methanomicrobiales;D_4__Methanomicrobiaceae | D_0__Bacteria;D_1__Chloroflexi;D_2__Dehalococcoidia;D_3__MSBL5;D_4__uncultured bacterium |
| Minerotrophic | D_0__Archaea;D_1__Euryarchaeota;D_2__Methanobacteria;D_3__Methanobacteriales;D_4__Methanobacteriaceae | D_0__Archaea;D_1__Euryarchaeota;D_2__Methanomicrobia;D_3__Methanomicrobiales;D_4__Methanospirillaceae |
| Minerotrophic | D_0__Bacteria;D_1__Proteobacteria;D_2__Alphaproteobacteria;D_3__Alphaproteobacteria Incertae Sedis;D_4__Unknown Family | D_0__Bacteria;D_1__Proteobacteria;D_2__Gammaproteobacteria;D_3__Methylococcales;D_4__Methylococcaceae |
| Minerotrophic | D_0__Bacteria;D_1__Actinobacteria;D_2__MB-A2-108;D_3__uncultured bacterium;D_4__uncultured bacterium | D_0__Bacteria;D_1__Proteobacteria;D_2__Gammaproteobacteria;D_3__Methylococcales;D_4__Methylococcaceae |
| Minerotrophic | D_0__Archaea;D_1__Euryarchaeota;D_2__Methanomicrobia;D_3__Methanomicrobiales;D_4__Methanospirillaceae | D_0__Bacteria;D_1__Proteobacteria;D_2__Gammaproteobacteria;D_3__Pseudomonadales;D_4__Moraxellaceae |
| Minerotrophic | D_0__Archaea;D_1__Euryarchaeota;D_2__Methanomicrobia;D_3__Methanosarcinales;D_4__Methanosarcinaceae | D_0__Bacteria;D_1__Chlamydiae;D_2__LD1-PA32;D_3__uncultured bacterium;D_4__uncultured bacterium |
| Minerotrophic | D_0__Archaea;D_1__Euryarchaeota;D_2__Methanomicrobia;D_3__Methanomicrobiales;D_4__Methanoregulaceae | D_0__Bacteria;D_1__Planctomycetes;D_2__Phycisphaerae;D_3__Phycisphaerales;__ |
| Minerotrophic | D_0__Bacteria;D_1__Proteobacteria;D_2__Alphaproteobacteria;D_3__Rhodospirillales;D_4__B79 | D_0__Bacteria;D_1__Proteobacteria;D_2__Gammaproteobacteria;D_3__Methylococcales;D_4__Methylococcaceae |
| Minerotrophic | D_0__Archaea;D_1__Euryarchaeota;D_2__Methanobacteria;D_3__Methanobacteriales;D_4__Methanobacteriaceae | D_0__Bacteria;D_1__WS2;__;__;__ |
| Minerotrophic | D_0__Bacteria;D_1__Acidobacteria;D_2__Subgroup 6;__;__ | D_0__Bacteria;D_1__Proteobacteria;D_2__Alphaproteobacteria;D_3__Rhizobiales;D_4__Methylocystaceae |
| Minerotrophic | D_0__Archaea;D_1__Euryarchaeota;D_2__Methanomicrobia;D_3__Methanomicrobiales;D_4__Methanoregulaceae | D_0__Bacteria;D_1__Cloacimonetes;__;__;__ |
| Minerotrophic | D_0__Archaea;D_1__Euryarchaeota;D_2__Methanomicrobia;D_3__Methanosarcinales;D_4__GOM Arc I | D_0__Bacteria;D_1__Nitrospirae;D_2__Nitrospira;D_3__Nitrospirales;D_4__Nitrospirales Incertae Sedis |
| Minerotrophic | D_0__Archaea;D_1__Euryarchaeota;D_2__Methanomicrobia;D_3__Methanosarcinales;D_4__Methanosaetaceae | D_0__Bacteria;D_1__Acidobacteria;D_2__Holophagae;D_3__TPD-58;D_4__uncultured bacterium |
| Minerotrophic | D_0__Bacteria;D_1__Chloroflexi;D_2__S085;D_3__uncultured bacterium;D_4__uncultured bacterium | D_0__Bacteria;D_1__Proteobacteria;D_2__Gammaproteobacteria;D_3__Methylococcales;D_4__Methylococcaceae |
| Minerotrophic | D_0__Bacteria;D_1__Proteobacteria;D_2__Alphaproteobacteria;D_3__Sphingomonadales;__ | D_0__Bacteria;D_1__Proteobacteria;D_2__Gammaproteobacteria;D_3__Methylococcales;D_4__Methylococcaceae |
| Minerotrophic | D_0__Archaea;D_1__Euryarchaeota;D_2__Methanomicrobia;D_3__Methanosarcinales;D_4__GOM Arc I | D_0__Bacteria;D_1__Chloroflexi;D_2__Ktedonobacteria;D_3__Ktedonobacterales;D_4__JG30a-KF-32 |
| Minerotrophic | D_0__Bacteria;D_1__Planctomycetes;D_2__Planctomycetacia;D_3__Planctomycetales;D_4__Planctomycetaceae | D_0__Bacteria;D_1__Proteobacteria;D_2__Alphaproteobacteria;D_3__Rhizobiales;D_4__Methylocystaceae |
| Minerotrophic | D_0__Archaea;D_1__Euryarchaeota;D_2__Methanobacteria;D_3__Methanobacteriales;D_4__Methanobacteriaceae | D_0__Bacteria;D_1__Chloroflexi;D_2__Dehalococcoidia;__;__ |
| Minerotrophic | D_0__Archaea;D_1__Euryarchaeota;D_2__Methanomicrobia;D_3__Methanosarcinales;__ | D_0__Bacteria;D_1__Bacteroidetes;D_2__Sphingobacteriia;D_3__Sphingobacteriales;D_4__env.OPS 17 |
| Minerotrophic | D_0__Archaea;D_1__Euryarchaeota;D_2__Methanobacteria;D_3__Methanobacteriales;D_4__Methanobacteriaceae | D_0__Bacteria;D_1__Chloroflexi;D_2__Dehalococcoidia;D_3__vadinBA26;D_4__uncultured bacterium |
| Minerotrophic | D_0__Archaea;D_1__Euryarchaeota;D_2__Methanomicrobia;D_3__Methanomicrobiales;D_4__Methanoregulaceae | D_0__Bacteria;D_1__Bacteroidetes;D_2__Bacteroidia;D_3__Bacteroidales;D_4__Prolixibacteraceae |
| Minerotrophic | D_0__Archaea;D_1__Euryarchaeota;D_2__Methanomicrobia;D_3__Methanosarcinales;__ | D_0__Bacteria;D_1__Chloroflexi;D_2__TK10;D_3__uncultured bacterium;D_4__uncultured bacterium |
| Minerotrophic | D_0__Archaea;D_1__Euryarchaeota;D_2__Methanomicrobia;D_3__Methanosarcinales;D_4__Methanosaetaceae | D_0__Bacteria;D_1__Chloroflexi;D_2__SJA-15;D_3__uncultured bacterium;D_4__uncultured bacterium |
| Minerotrophic | D_0__Bacteria;D_1__Proteobacteria;D_2__Gammaproteobacteria;D_3__Methylococcales;D_4__Methylococcaceae | D_0__Bacteria;D_1__TM6 (Dependentiae);D_2__uncultured bacterium;D_3__uncultured bacterium;D_4__uncultured bacterium |
| Minerotrophic | D_0__Archaea;D_1__Euryarchaeota;D_2__Methanomicrobia;D_3__Methanosarcinales;D_4__GOM Arc I | D_0__Bacteria;D_1__Candidatus Berkelbacteria;D_2__uncultured bacterium;D_3__uncultured bacterium;D_4__uncultured bacterium |
| Minerotrophic | D_0__Archaea;D_1__Euryarchaeota;D_2__Methanomicrobia;D_3__Methanomicrobiales;D_4__Methanospirillaceae | D_0__Archaea;D_1__Euryarchaeota;D_2__Thermoplasmata;D_3__Thermoplasmatales;D_4__Terrestrial Miscellaneous Gp(TMEG) |
| Minerotrophic | D_0__Archaea;D_1__Euryarchaeota;D_2__Methanomicrobia;D_3__Methanosarcinales;D_4__GOM Arc I | D_0__Bacteria;D_1__Actinobacteria;D_2__Actinobacteria;D_3__Corynebacteriales;D_4__Nocardiaceae |
| Minerotrophic | D_0__Archaea;D_1__Euryarchaeota;D_2__Methanomicrobia;D_3__Methanosarcinales;D_4__Methanosaetaceae | D_0__Bacteria;D_1__Firmicutes;D_2__Clostridia;D_3__Clostridiales;D_4__Christensenellaceae |
| Minerotrophic | D_0__Archaea;D_1__Thaumarchaeota;D_2__FHMa11 terrestrial group;__;__ | D_0__Bacteria;D_1__Proteobacteria;D_2__Gammaproteobacteria;D_3__Methylococcales;D_4__Methylococcaceae |
| Minerotrophic | D_0__Archaea;D_1__Euryarchaeota;D_2__Methanomicrobia;D_3__Methanosarcinales;D_4__GOM Arc I | D_0__Archaea;D_1__Thaumarchaeota;D_2__South African Gold Mine Gp 1(SAGMCG-1);D_3__uncultured archaeon;D_4__uncultured archaeon |
| Minerotrophic | D_0__Archaea;D_1__Euryarchaeota;D_2__Methanomicrobia;D_3__Methanosarcinales;D_4__Methanosaetaceae | D_0__Bacteria;D_1__WS1;D_2__uncultured bacterium;D_3__uncultured bacterium;D_4__uncultured bacterium |
| Minerotrophic | D_0__Archaea;D_1__Euryarchaeota;D_2__Methanomicrobia;D_3__Methanomicrobiales;D_4__Methanoregulaceae | D_0__Bacteria;D_1__Armatimonadetes;D_2__Chthonomonadetes;D_3__Chthonomonadales;D_4__uncultured bacterium |
| Minerotrophic | D_0__Bacteria;D_1__Chloroflexi;D_2__Ardenticatenia;D_3__uncultured;D_4__uncultured bacterium | D_0__Bacteria;D_1__Proteobacteria;D_2__Gammaproteobacteria;D_3__Methylococcales;D_4__Methylococcaceae |
| Minerotrophic | D_0__Archaea;D_1__Euryarchaeota;D_2__Methanomicrobia;D_3__Methanosarcinales;D_4__Methanosarcinaceae | D_0__Bacteria;D_1__Actinobacteria;D_2__OPB41;__;__ |
| Minerotrophic | D_0__Archaea;D_1__Euryarchaeota;D_2__Methanomicrobia;D_3__Methanosarcinales;D_4__Methanosaetaceae | D_0__Bacteria;D_1__Aminicenantes;D_2__uncultured bacterium;D_3__uncultured bacterium;D_4__uncultured bacterium |
| Minerotrophic | D_0__Archaea;D_1__Euryarchaeota;D_2__Methanomicrobia;D_3__Methanomicrobiales;D_4__Methanoregulaceae | D_0__Bacteria;D_1__Chloroflexi;D_2__Dehalococcoidia;D_3__Dehalococcoidales;D_4__Dehalococcoidaceae |
| Minerotrophic | D_0__Archaea;D_1__Euryarchaeota;D_2__Methanomicrobia;D_3__Methanomicrobiales;D_4__Methanoregulaceae | D_0__Bacteria;D_1__Armatimonadetes;D_2__Chthonomonadetes;D_3__Chthonomonadales;__ |
| Minerotrophic | D_0__Archaea;D_1__Euryarchaeota;D_2__Methanobacteria;D_3__Methanobacteriales;D_4__Methanobacteriaceae | D_0__Bacteria;D_1__Acidobacteria;D_2__Subgroup 17;D_3__uncultured bacterium;D_4__uncultured bacterium |
| Minerotrophic | D_0__Bacteria;D_1__Firmicutes;D_2__Bacilli;D_3__Bacillales;D_4__Staphylococcaceae | D_0__Bacteria;D_1__Proteobacteria;D_2__Gammaproteobacteria;D_3__Methylococcales;D_4__Methylococcaceae |
| Minerotrophic | D_0__Archaea;D_1__Euryarchaeota;D_2__Methanobacteria;D_3__Methanobacteriales;D_4__Methanobacteriaceae | D_0__Bacteria;D_1__AC1;D_2__uncultured bacterium;D_3__uncultured bacterium;D_4__uncultured bacterium |
| Minerotrophic | D_0__Archaea;D_1__Euryarchaeota;D_2__Methanomicrobia;D_3__Methanocellales;D_4__Methanocellaceae | D_0__Bacteria;D_1__Proteobacteria;D_2__Alphaproteobacteria;D_3__Rhizobiales;D_4__Rhizobiaceae |
| Minerotrophic | D_0__Archaea;D_1__Euryarchaeota;D_2__Methanomicrobia;D_3__Methanosarcinales;D_4__Methanosarcinaceae | D_0__Archaea;D_1__pMC2A209;D_2__uncultured archaeon;D_3__uncultured archaeon;D_4__uncultured archaeon |
| Minerotrophic | D_0__Archaea;D_1__Euryarchaeota;D_2__Methanomicrobia;D_3__Methanosarcinales;D_4__Methanosaetaceae | D_0__Bacteria;D_1__Planctomycetes;D_2__Phycisphaerae;D_3__MSBL9;__ |
| Minerotrophic | D_0__Bacteria;D_1__Proteobacteria;D_2__Alphaproteobacteria;D_3__Rhizobiales;D_4__Hyphomicrobiaceae | D_0__Bacteria;D_1__Proteobacteria;D_2__Alphaproteobacteria;D_3__Rhizobiales;D_4__Methylocystaceae |
| Minerotrophic | D_0__Archaea;D_1__Euryarchaeota;D_2__Methanomicrobia;D_3__Methanomicrobiales;D_4__Methanospirillaceae | D_0__Bacteria;D_1__Chloroflexi;D_2__Dehalococcoidia;D_3__MSBL5;D_4__uncultured bacterium |
| Minerotrophic | D_0__Archaea;D_1__Euryarchaeota;D_2__Methanomicrobia;D_3__Methanosarcinales;D_4__GOM Arc I | D_0__Bacteria;D_1__Omnitrophica;__;__;__ |
| Minerotrophic | D_0__Bacteria;D_1__Proteobacteria;D_2__Gammaproteobacteria;D_3__Methylococcales;D_4__Methylococcaceae | D_0__Bacteria;D_1__Verrucomicrobia;D_2__OPB35 soil group;D_3__uncultured bacterium;D_4__uncultured bacterium |
| Minerotrophic | D_0__Archaea;D_1__Euryarchaeota;D_2__Methanomicrobia;D_3__Methanocellales;D_4__Methanocellaceae | D_0__Bacteria;D_1__Firmicutes;D_2__Bacilli;D_3__Lactobacillales;D_4__Enterococcaceae |
| Minerotrophic | D_0__Archaea;D_1__Euryarchaeota;D_2__Methanomicrobia;D_3__Methanomicrobiales;D_4__Methanoregulaceae | D_0__Bacteria;D_1__Acidobacteria;D_2__Subgroup 25;__;__ |
| Minerotrophic | D_0__Bacteria;D_1__Proteobacteria;D_2__Alphaproteobacteria;D_3__Rhodospirillales;D_4__Acetobacteraceae | D_0__Bacteria;D_1__Proteobacteria;D_2__Gammaproteobacteria;D_3__Methylococcales;D_4__Methylococcaceae |
| Minerotrophic | D_0__Archaea;D_1__Euryarchaeota;D_2__Methanomicrobia;D_3__Methanosarcinales;D_4__Methanosarcinaceae | D_0__Bacteria;D_1__Actinobacteria;D_2__Actinobacteria;D_3__Micrococcales;D_4__Microbacteriaceae |
| Minerotrophic | D_0__Bacteria;D_1__Proteobacteria;D_2__Gammaproteobacteria;D_3__Methylococcales;D_4__Methylococcaceae | D_0__Bacteria;D_1__Proteobacteria;D_2__Gammaproteobacteria;D_3__Pseudomonadales;D_4__Pseudomonadaceae |
| Minerotrophic | D_0__Bacteria;D_1__Chloroflexi;D_2__Thermomicrobia;__;__ | D_0__Bacteria;D_1__Proteobacteria;D_2__Gammaproteobacteria;D_3__Methylococcales;D_4__Methylococcaceae |
| Minerotrophic | D_0__Archaea;D_1__Euryarchaeota;D_2__Methanomicrobia;D_3__Methanomicrobiales;D_4__Methanoregulaceae | D_0__Bacteria;D_1__Proteobacteria;D_2__Deltaproteobacteria;D_3__Myxococcales;D_4__Myxococcaceae |
| Minerotrophic | D_0__Archaea;D_1__Bathyarchaeota;Ambiguous_taxa;Ambiguous_taxa;Ambiguous_taxa | D_0__Archaea;D_1__Euryarchaeota;D_2__Methanomicrobia;D_3__Methanosarcinales;D_4__Methanosarcinaceae |
| Minerotrophic | D_0__Archaea;D_1__Euryarchaeota;D_2__Methanomicrobia;D_3__Methanosarcinales;D_4__GOM Arc I | D_0__Bacteria;D_1__Proteobacteria;D_2__Gammaproteobacteria;D_3__uncultured;D_4__uncultured gamma proteobacterium |
| Minerotrophic | D_0__Archaea;D_1__Euryarchaeota;D_2__Methanomicrobia;D_3__Methanomicrobiales;D_4__Methanoregulaceae | D_0__Archaea;D_1__Lokiarchaeota;D_2__uncultured archaeon;D_3__uncultured archaeon;D_4__uncultured archaeon |
| Minerotrophic | D_0__Bacteria;D_1__Planctomycetes;D_2__Phycisphaerae;D_3__MSBL9;__ | D_0__Bacteria;D_1__Proteobacteria;D_2__Alphaproteobacteria;D_3__Rhizobiales;D_4__Methylocystaceae |
| Minerotrophic | D_0__Archaea;D_1__Euryarchaeota;D_2__Methanomicrobia;D_3__Methanosarcinales;D_4__GOM Arc I | D_0__Bacteria;D_1__Acidobacteria;D_2__Subgroup 15;D_3__uncultured bacterium;D_4__uncultured bacterium |
| Minerotrophic | D_0__Archaea;D_1__Euryarchaeota;D_2__Methanomicrobia;D_3__Methanosarcinales;D_4__GOM Arc I | D_0__Bacteria;D_1__Chlamydiae;D_2__Chlamydiae;D_3__Chlamydiales;D_4__Simkaniaceae |
| Minerotrophic | D_0__Archaea;D_1__Euryarchaeota;D_2__Methanobacteria;D_3__Methanobacteriales;D_4__Methanobacteriaceae | D_0__Bacteria;D_1__Chloroflexi;D_2__Dehalococcoidia;D_3__GIF9;__ |
| Minerotrophic | D_0__Archaea;D_1__Euryarchaeota;D_2__Methanomicrobia;D_3__Methanocellales;D_4__Methanocellaceae | D_0__Archaea;D_1__Euryarchaeota;D_2__Methanomicrobia;D_3__Methanosarcinales;D_4__Methanosarcinaceae |
| Minerotrophic | D_0__Bacteria;D_1__Chloroflexi;D_2__Caldilineae;D_3__Caldilineales;D_4__Caldilineaceae | D_0__Bacteria;D_1__Proteobacteria;D_2__Gammaproteobacteria;D_3__Methylococcales;D_4__Methylococcaceae |
| Minerotrophic | D_0__Archaea;D_1__Candidate division YNPFFA;__;__;__ | D_0__Archaea;D_1__Euryarchaeota;D_2__Methanomicrobia;D_3__Methanosarcinales;D_4__Methanosarcinaceae |
| Minerotrophic | D_0__Bacteria;D_1__Chloroflexi;D_2__Thermomicrobia;D_3__JG30-KF-CM45;__ | D_0__Bacteria;D_1__Proteobacteria;D_2__Gammaproteobacteria;D_3__Methylococcales;D_4__Methylococcaceae |
| Minerotrophic | D_0__Archaea;D_1__Euryarchaeota;D_2__Methanomicrobia;D_3__Methanosarcinales;__ | D_0__Bacteria;D_1__Firmicutes;D_2__Clostridia;D_3__Halanaerobiales;D_4__ODP1230B8.23 |
| Minerotrophic | D_0__Archaea;D_1__Euryarchaeota;D_2__Methanomicrobia;D_3__Methanosarcinales;D_4__GOM Arc I | D_0__Bacteria;D_1__Proteobacteria;D_2__Deltaproteobacteria;D_3__Desulfobacterales;D_4__Desulfobacteraceae |
| Minerotrophic | D_0__Archaea;D_1__Euryarchaeota;D_2__Methanomicrobia;D_3__Methanosarcinales;D_4__Methanosaetaceae | D_0__Bacteria;D_1__Ignavibacteriae;D_2__Ignavibacteria;D_3__Ignavibacteriales;D_4__SR-FBR-L83 |
| Minerotrophic | D_0__Bacteria;D_1__Proteobacteria;D_2__Alphaproteobacteria;D_3__Rhizobiales;D_4__Methylocystaceae | D_0__Bacteria;D_1__Proteobacteria;D_2__Alphaproteobacteria;D_3__Rhizobiales;D_4__Xanthobacteraceae |
| Minerotrophic | D_0__Archaea;D_1__Euryarchaeota;D_2__Methanobacteria;D_3__Methanobacteriales;D_4__Methanobacteriaceae | D_0__Bacteria;D_1__Actinobacteria;D_2__Actinobacteria;D_3__Propionibacteriales;D_4__Nocardioidaceae |
| Minerotrophic | D_0__Archaea;D_1__Euryarchaeota;D_2__Methanomicrobia;D_3__Methanosarcinales;D_4__GOM Arc I | D_0__Bacteria;D_1__Proteobacteria;D_2__Betaproteobacteria;D_3__uncultured;D_4__uncultured bacterium |
| Minerotrophic | D_0__Archaea;D_1__Euryarchaeota;D_2__Methanomicrobia;D_3__Methanosarcinales;D_4__Methanosaetaceae | D_0__Bacteria;D_1__Ignavibacteriae;D_2__Ignavibacteria;D_3__Ignavibacteriales;__ |
| Minerotrophic | D_0__Archaea;D_1__Euryarchaeota;D_2__Methanomicrobia;D_3__Methanosarcinales;D_4__Methanosaetaceae | D_0__Archaea;D_1__Euryarchaeota;D_2__Thermoplasmata;D_3__Thermoplasmatales;D_4__Terrestrial Miscellaneous Gp(TMEG) |
| Minerotrophic | D_0__Bacteria;D_1__Chloroflexi;D_2__Chloroflexia;D_3__Chloroflexales;D_4__Roseiflexaceae | D_0__Bacteria;D_1__Proteobacteria;D_2__Gammaproteobacteria;D_3__Methylococcales;D_4__Methylococcaceae |
| Minerotrophic | D_0__Archaea;D_1__Euryarchaeota;D_2__Methanomicrobia;D_3__Methanosarcinales;D_4__GOM Arc I | D_0__Bacteria;D_1__Proteobacteria;D_2__Deltaproteobacteria;D_3__SAR324 clade(Marine group B);__ |
| Minerotrophic | D_0__Archaea;D_1__Euryarchaeota;D_2__Methanomicrobia;D_3__Methanosarcinales;D_4__GOM Arc I | D_0__Bacteria;D_1__Planctomycetes;D_2__Phycisphaerae;D_3__Tepidisphaerales;D_4__Tepidisphaeraceae |
| Minerotrophic | D_0__Archaea;D_1__Euryarchaeota;D_2__Methanomicrobia;D_3__Methanosarcinales;D_4__Methanosaetaceae | D_0__Bacteria;D_1__Proteobacteria;D_2__Gammaproteobacteria;D_3__Xanthomonadales;D_4__uncultured |
| Minerotrophic | D_0__Archaea;D_1__Euryarchaeota;D_2__Methanomicrobia;D_3__Methanomicrobiales;D_4__Methanoregulaceae | D_0__Bacteria;D_1__Proteobacteria;D_2__Deltaproteobacteria;D_3__Oligoflexales;D_4__0319-6G20 |
| Minerotrophic | D_0__Bacteria;D_1__Acidobacteria;D_2__Subgroup 18;__;__ | D_0__Bacteria;D_1__Proteobacteria;D_2__Gammaproteobacteria;D_3__Methylococcales;D_4__Methylococcaceae |
| Minerotrophic | D_0__Archaea;D_1__Euryarchaeota;D_2__Methanomicrobia;D_3__Methanosarcinales;D_4__GOM Arc I | D_0__Bacteria;D_1__Chloroflexi;D_2__Ktedonobacteria;D_3__Ktedonobacterales;D_4__1921-3 |
| Minerotrophic | D_0__Archaea;D_1__Euryarchaeota;D_2__Methanomicrobia;D_3__Methanomicrobiales;D_4__Methanospirillaceae | D_0__Bacteria;D_1__Actinobacteria;D_2__Coriobacteriia;D_3__Coriobacteriales;D_4__Coriobacteriaceae |
| Minerotrophic | D_0__Archaea;D_1__Euryarchaeota;D_2__Methanomicrobia;D_3__Methanosarcinales;D_4__Methanosaetaceae | D_0__Bacteria;D_1__Planctomycetes;D_2__Phycisphaerae;D_3__Pla1 lineage;D_4__uncultured bacterium |
| Minerotrophic | D_0__Archaea;D_1__Euryarchaeota;D_2__Methanomicrobia;D_3__Methanosarcinales;D_4__Methanosarcinaceae | D_0__Bacteria;D_1__Planctomycetes;D_2__BD7-11;D_3__uncultured bacterium;D_4__uncultured bacterium |
| Minerotrophic | D_0__Archaea;D_1__Euryarchaeota;D_2__Methanomicrobia;D_3__Methanosarcinales;D_4__Methanosarcinaceae | D_0__Bacteria;D_1__CPR2;__;__;__ |
| Minerotrophic | D_0__Bacteria;D_1__Proteobacteria;D_2__Betaproteobacteria;D_3__Hydrogenophilales;D_4__Hydrogenophilaceae | D_0__Bacteria;D_1__Proteobacteria;D_2__Gammaproteobacteria;D_3__Methylococcales;D_4__Methylococcaceae |
| Minerotrophic | D_0__Archaea;D_1__Euryarchaeota;D_2__Methanomicrobia;D_3__Methanosarcinales;D_4__Methanosaetaceae | D_0__Bacteria;D_1__Planctomycetes;D_2__Phycisphaerae;D_3__mle1-8;__ |
| Minerotrophic | D_0__Archaea;D_1__Euryarchaeota;D_2__Methanomicrobia;D_3__Methanocellales;D_4__Methanocellaceae | D_0__Archaea;D_1__Euryarchaeota;D_2__Methanomicrobia;D_3__Methanomicrobiales;D_4__Methanomicrobiaceae |
| Minerotrophic | D_0__Archaea;D_1__Euryarchaeota;D_2__Methanomicrobia;D_3__Methanomicrobiales;D_4__Methanospirillaceae | D_0__Bacteria;D_1__Actinobacteria;D_2__Actinobacteria;D_3__Frankiales;D_4__Frankiaceae |
| Minerotrophic | D_0__Archaea;D_1__Euryarchaeota;D_2__Methanomicrobia;D_3__Methanomicrobiales;D_4__Methanoregulaceae | D_0__Bacteria;D_1__Planctomycetes;D_2__Phycisphaerae;D_3__Pla1 lineage;D_4__uncultured bacterium |
| Minerotrophic | D_0__Bacteria;D_1__Acidobacteria;D_2__Subgroup 12;D_3__uncultured bacterium;D_4__uncultured bacterium | D_0__Bacteria;D_1__Proteobacteria;D_2__Gammaproteobacteria;D_3__Methylococcales;D_4__Methylococcaceae |
| Minerotrophic | D_0__Archaea;D_1__Euryarchaeota;D_2__Methanomicrobia;D_3__Methanomicrobiales;D_4__Methanospirillaceae | D_0__Bacteria;D_1__Actinobacteria;D_2__Thermoleophilia;D_3__Solirubrobacterales;D_4__0319-6M6 |
| Minerotrophic | D_0__Archaea;D_1__Euryarchaeota;D_2__Methanomicrobia;D_3__Methanomicrobiales;D_4__Methanoregulaceae | D_0__Bacteria;D_1__Proteobacteria;D_2__Alphaproteobacteria;D_3__Rhodospirillales;D_4__KCM-B-60 |
| Minerotrophic | D_0__Bacteria;D_1__Acidobacteria;D_2__Subgroup 13;D_3__uncultured bacterium;D_4__uncultured bacterium | D_0__Bacteria;D_1__Proteobacteria;D_2__Gammaproteobacteria;D_3__Methylococcales;D_4__Methylococcaceae |
| Minerotrophic | D_0__Archaea;D_1__Euryarchaeota;D_2__Methanobacteria;D_3__Methanobacteriales;D_4__Methanobacteriaceae | D_0__Bacteria;D_1__Chloroflexi;D_2__SJA-68;D_3__uncultured bacterium;D_4__uncultured bacterium |
| Minerotrophic | D_0__Bacteria;D_1__Planctomycetes;__;__;__ | D_0__Bacteria;D_1__Proteobacteria;D_2__Gammaproteobacteria;D_3__Methylococcales;D_4__Methylococcaceae |
| Minerotrophic | D_0__Archaea;D_1__Euryarchaeota;D_2__Methanomicrobia;D_3__Methanomicrobiales;D_4__Methanospirillaceae | D_0__Bacteria;D_1__Spirochaetae;D_2__Spirochaetes;D_3__Spirochaetales;D_4__Spirochaetaceae |
| Minerotrophic | D_0__Archaea;D_1__Euryarchaeota;D_2__Methanomicrobia;D_3__Methanomicrobiales;D_4__Methanoregulaceae | D_0__Bacteria;D_1__Aminicenantes;D_2__uncultured bacterium;D_3__uncultured bacterium;D_4__uncultured bacterium |
| Minerotrophic | D_0__Archaea;D_1__Euryarchaeota;D_2__Methanobacteria;D_3__Methanobacteriales;D_4__Methanobacteriaceae | D_0__Bacteria;D_1__Chloroflexi;D_2__Dehalococcoidia;D_3__Dehalococcoidales;D_4__uncultured |
| Minerotrophic | D_0__Bacteria;D_1__Ignavibacteriae;D_2__Ignavibacteria;D_3__Ignavibacteriales;__ | D_0__Bacteria;D_1__Proteobacteria;D_2__Alphaproteobacteria;D_3__Rhizobiales;D_4__Methylocystaceae |
| Minerotrophic | D_0__Archaea;D_1__Euryarchaeota;D_2__Methanobacteria;D_3__Methanobacteriales;D_4__Methanobacteriaceae | D_0__Bacteria;D_1__Spirochaetae;D_2__Spirochaetes;D_3__Spirochaetales;D_4__Spirochaetales Incertae Sedis |
| Minerotrophic | D_0__Archaea;D_1__Euryarchaeota;D_2__Methanomicrobia;D_3__Methanomicrobiales;D_4__Methanoregulaceae | D_0__Bacteria;D_1__Ignavibacteriae;D_2__Ignavibacteria;D_3__Ignavibacteriales;D_4__PHOS-HE36 |
| Minerotrophic | D_0__Bacteria;D_1__Acidobacteria;D_2__Subgroup 11;__;__ | D_0__Bacteria;D_1__Proteobacteria;D_2__Gammaproteobacteria;D_3__Methylococcales;D_4__Methylococcaceae |
| Minerotrophic | D_0__Bacteria;D_1__Chloroflexi;D_2__Ktedonobacteria;D_3__B12-WMSP1;D_4__uncultured bacterium | D_0__Bacteria;D_1__Proteobacteria;D_2__Gammaproteobacteria;D_3__Methylococcales;D_4__Methylococcaceae |
| Minerotrophic | D_0__Archaea;D_1__Euryarchaeota;D_2__Methanomicrobia;D_3__Methanomicrobiales;D_4__Methanoregulaceae | D_0__Bacteria;D_1__Proteobacteria;D_2__Betaproteobacteria;D_3__Burkholderiales;D_4__Oxalobacteraceae |
| Minerotrophic | D_0__Bacteria;D_1__Bacteroidetes;D_2__Sphingobacteriia;D_3__Sphingobacteriales;__ | D_0__Bacteria;D_1__Proteobacteria;D_2__Gammaproteobacteria;D_3__Methylococcales;D_4__Methylococcaceae |
| Minerotrophic | D_0__Archaea;D_1__Euryarchaeota;D_2__Methanomicrobia;D_3__Methanosarcinales;D_4__Methanosarcinaceae | D_0__Bacteria;D_1__Actinobacteria;D_2__Actinobacteria;D_3__Micromonosporales;D_4__Micromonosporaceae |
| Minerotrophic | D_0__Archaea;D_1__Euryarchaeota;D_2__Methanomicrobia;D_3__Methanomicrobiales;D_4__Methanoregulaceae | D_0__Bacteria;D_1__Firmicutes;D_2__Bacilli;D_3__Bacillales;D_4__Planococcaceae |
| Minerotrophic | D_0__Archaea;D_1__Euryarchaeota;D_2__Methanomicrobia;D_3__Methanomicrobiales;D_4__Methanospirillaceae | D_0__Bacteria;D_1__Latescibacteria;__;__;__ |
| Minerotrophic | D_0__Archaea;D_1__Euryarchaeota;D_2__Methanomicrobia;D_3__Methanosarcinales;D_4__Methanosarcinaceae | D_0__Bacteria;D_1__Chloroflexi;D_2__Dehalococcoidia;D_3__MSBL5;D_4__uncultured bacterium |
| Minerotrophic | D_0__Archaea;D_1__Euryarchaeota;D_2__Methanomicrobia;D_3__Methanosarcinales;D_4__GOM Arc I | D_0__Bacteria;D_1__Proteobacteria;D_2__Betaproteobacteria;__;__ |
| Minerotrophic | D_0__Archaea;D_1__Euryarchaeota;D_2__Methanobacteria;D_3__Methanobacteriales;D_4__Methanobacteriaceae | D_0__Bacteria;D_1__Proteobacteria;D_2__Alphaproteobacteria;D_3__Rhizobiales;D_4__A0839 |
| Minerotrophic | D_0__Archaea;D_1__Euryarchaeota;D_2__Methanomicrobia;D_3__Methanosarcinales;D_4__Methanosaetaceae | D_0__Bacteria;D_1__Proteobacteria;D_2__Betaproteobacteria;D_3__Burkholderiales;D_4__Oxalobacteraceae |
| Minerotrophic | D_0__Archaea;D_1__Euryarchaeota;D_2__Methanobacteria;D_3__Methanobacteriales;D_4__Methanobacteriaceae | D_0__Bacteria;D_1__Actinobacteria;D_2__Actinobacteria;D_3__Micrococcales;D_4__Microbacteriaceae |
| Minerotrophic | D_0__Archaea;D_1__Euryarchaeota;D_2__Methanomicrobia;D_3__Methanosarcinales;D_4__Methanosaetaceae | D_0__Bacteria;D_1__Bacteroidetes;D_2__Bacteroidetes vadinHA17;D_3__uncultured bacterium;D_4__uncultured bacterium |
| Minerotrophic | D_0__Archaea;D_1__Euryarchaeota;D_2__Methanobacteria;D_3__Methanobacteriales;D_4__Methanobacteriaceae | D_0__Archaea;D_1__Euryarchaeota;D_2__Thermoplasmata;D_3__Thermoplasmatales;D_4__Terrestrial Miscellaneous Gp(TMEG) |
| Minerotrophic | D_0__Archaea;D_1__Euryarchaeota;D_2__Methanomicrobia;D_3__Methanosarcinales;D_4__GOM Arc I | D_0__Bacteria;D_1__Proteobacteria;D_2__Gammaproteobacteria;D_3__Legionellales;D_4__Coxiellaceae |
| Minerotrophic | D_0__Archaea;D_1__Euryarchaeota;D_2__Methanomicrobia;D_3__Methanomicrobiales;D_4__Methanoregulaceae | D_0__Bacteria;D_1__Latescibacteria;D_2__Latescibacteria Incertae Sedis;D_3__Unknown Order;D_4__Unknown Family |
| Minerotrophic | D_0__Bacteria;D_1__Acidobacteria;D_2__Subgroup 5;__;__ | D_0__Bacteria;D_1__Proteobacteria;D_2__Gammaproteobacteria;D_3__Methylococcales;D_4__Methylococcaceae |
| Minerotrophic | D_0__Bacteria;D_1__Proteobacteria;D_2__Gammaproteobacteria;D_3__Methylococcales;D_4__Methylococcaceae | D_0__Bacteria;D_1__Verrucomicrobia;D_2__Opitutae;D_3__Opitutae vadinHA64;D_4__uncultured bacterium |
| Minerotrophic | D_0__Archaea;D_1__Euryarchaeota;D_2__Methanomicrobia;D_3__Methanosarcinales;D_4__Methanosaetaceae | D_0__Bacteria;D_1__Chloroflexi;D_2__Dehalococcoidia;__;__ |
| Minerotrophic | D_0__Bacteria;D_1__Chloroflexi;D_2__Ktedonobacteria;D_3__Ktedonobacterales;__ | D_0__Bacteria;D_1__Proteobacteria;D_2__Gammaproteobacteria;D_3__Methylococcales;D_4__Methylococcaceae |
| Minerotrophic | D_0__Bacteria;D_1__Actinobacteria;D_2__OPB41;D_3__uncultured bacterium;D_4__uncultured bacterium | D_0__Bacteria;D_1__Proteobacteria;D_2__Alphaproteobacteria;D_3__Rhizobiales;D_4__Methylocystaceae |
| Minerotrophic | D_0__Archaea;D_1__Euryarchaeota;D_2__Methanomicrobia;D_3__Methanosarcinales;D_4__Methanosaetaceae | D_0__Bacteria;D_1__Armatimonadetes;D_2__Chthonomonadetes;D_3__Chthonomonadales;__ |
| Minerotrophic | D_0__Archaea;D_1__Euryarchaeota;D_2__Methanobacteria;D_3__Methanobacteriales;D_4__Methanobacteriaceae | D_0__Bacteria;D_1__Ignavibacteriae;D_2__Ignavibacteria;D_3__Ignavibacteriales;D_4__PHOS-HE36 |
| Minerotrophic | D_0__Archaea;D_1__Euryarchaeota;D_2__Methanomicrobia;D_3__Methanomicrobiales;D_4__Methanoregulaceae | D_0__Bacteria;D_1__Proteobacteria;D_2__Deltaproteobacteria;D_3__Desulfarculales;D_4__Desulfarculaceae |
| Minerotrophic | D_0__Archaea;D_1__Euryarchaeota;D_2__Methanomicrobia;D_3__Methanomicrobiales;D_4__Methanoregulaceae | D_0__Bacteria;D_1__Planctomycetes;D_2__Phycisphaerae;D_3__Phycisphaerales;D_4__08D2Z94 hypersaline microbial mat group |
| Minerotrophic | D_0__Archaea;D_1__Euryarchaeota;D_2__Methanobacteria;D_3__Methanobacteriales;D_4__Methanobacteriaceae | D_0__Archaea;D_1__Euryarchaeota;D_2__Thermoplasmata;D_3__Thermoplasmatales;D_4__Marine Benthic Group D and DHVEG-1 |
| Minerotrophic | D_0__Archaea;D_1__Euryarchaeota;D_2__Methanomicrobia;D_3__Methanomicrobiales;__ | D_0__Bacteria;D_1__Proteobacteria;D_2__Deltaproteobacteria;D_3__Myxococcales;D_4__Archangiaceae |
| Minerotrophic | D_0__Archaea;D_1__Euryarchaeota;D_2__Methanomicrobia;D_3__Methanomicrobiales;D_4__Methanoregulaceae | D_0__Bacteria;D_1__Chloroflexi;D_2__Dehalococcoidia;D_3__vadinBA26;__ |
| Minerotrophic | D_0__Archaea;D_1__Euryarchaeota;D_2__Methanomicrobia;D_3__Methanosarcinales;D_4__GOM Arc I | D_0__Bacteria;D_1__Firmicutes;D_2__Clostridia;D_3__Clostridiales;D_4__Heliobacteriaceae |
| Minerotrophic | D_0__Archaea;D_1__Euryarchaeota;D_2__Methanomicrobia;D_3__Methanosarcinales;D_4__Methanosaetaceae | D_0__Bacteria;D_1__Bacteroidetes;D_2__Bacteroidia;D_3__Bacteroidales;D_4__Prolixibacteraceae |
| Minerotrophic | D_0__Archaea;D_1__Euryarchaeota;D_2__Methanomicrobia;D_3__Methanosarcinales;D_4__Methanosarcinaceae | D_0__Bacteria;D_1__AC1;D_2__uncultured bacterium;D_3__uncultured bacterium;D_4__uncultured bacterium |
| Minerotrophic | D_0__Archaea;D_1__Euryarchaeota;D_2__Methanomicrobia;D_3__Methanosarcinales;D_4__Methanosaetaceae | D_0__Bacteria;D_1__Latescibacteria;__;__;__ |
| Minerotrophic | D_0__Bacteria;D_1__Chloroflexi;D_2__Dehalococcoidia;D_3__GIF3;__ | D_0__Bacteria;D_1__Proteobacteria;D_2__Alphaproteobacteria;D_3__Rhizobiales;D_4__Methylocystaceae |
| Minerotrophic | D_0__Archaea;D_1__Euryarchaeota;D_2__Methanomicrobia;D_3__Methanosarcinales;D_4__GOM Arc I | D_0__Bacteria;D_1__Acidobacteria;D_2__Subgroup 13;__;__ |
| Minerotrophic | D_0__Archaea;D_1__Euryarchaeota;D_2__Methanomicrobia;D_3__Methanosarcinales;D_4__Methanosarcinaceae | D_0__Bacteria;D_1__Proteobacteria;D_2__Gammaproteobacteria;D_3__Xanthomonadales;D_4__Xanthomonadaceae |
| Minerotrophic | D_0__Archaea;D_1__Euryarchaeota;D_2__Methanomicrobia;D_3__Methanomicrobiales;D_4__Methanoregulaceae | D_0__Bacteria;D_1__Armatimonadetes;D_2__uncultured;__;__ |
| Minerotrophic | D_0__Archaea;D_1__Euryarchaeota;D_2__Methanomicrobia;D_3__Methanosarcinales;D_4__Methanosarcinaceae | D_0__Bacteria;D_1__Actinobacteria;D_2__Acidimicrobiia;D_3__Acidimicrobiales;D_4__uncultured |
| Minerotrophic | D_0__Bacteria;D_1__Chloroflexi;D_2__JG30-KF-CM66;D_3__uncultured bacterium;D_4__uncultured bacterium | D_0__Bacteria;D_1__Proteobacteria;D_2__Gammaproteobacteria;D_3__Methylococcales;D_4__Methylococcaceae |
| Minerotrophic | D_0__Archaea;D_1__Euryarchaeota;D_2__Methanobacteria;D_3__Methanobacteriales;D_4__Methanobacteriaceae | D_0__Archaea;D_1__Euryarchaeota;D_2__Methanomicrobia;D_3__Methanosarcinales;D_4__Methanosaetaceae |
| Minerotrophic | D_0__Archaea;D_1__Euryarchaeota;D_2__Methanomicrobia;D_3__Methanomicrobiales;D_4__Methanospirillaceae | D_0__Bacteria;D_1__Bacteroidetes;D_2__Sphingobacteriia;D_3__Sphingobacteriales;D_4__PHOS-HE51 |
| Minerotrophic | D_0__Archaea;D_1__Euryarchaeota;D_2__Methanomicrobia;D_3__Methanomicrobiales;D_4__Methanoregulaceae | D_0__Bacteria;D_1__Actinobacteria;D_2__OPB41;D_3__uncultured bacterium;D_4__uncultured bacterium |
| Minerotrophic | D_0__Archaea;D_1__Euryarchaeota;D_2__Methanomicrobia;D_3__Methanomicrobiales;D_4__Methanoregulaceae | D_0__Bacteria;D_1__Chloroflexi;D_2__KD4-96;D_3__uncultured bacterium;D_4__uncultured bacterium |
| Minerotrophic | D_0__Archaea;D_1__Euryarchaeota;D_2__Methanomicrobia;D_3__Methanosarcinales;D_4__GOM Arc I | D_0__Bacteria;D_1__Acidobacteria;D_2__Subgroup 2;D_3__uncultured bacterium;D_4__uncultured bacterium |
| Minerotrophic | D_0__Archaea;D_1__Euryarchaeota;D_2__Methanomicrobia;D_3__Methanomicrobiales;D_4__Methanoregulaceae | D_0__Bacteria;D_1__Bacteroidetes;D_2__Bacteroidetes vadinHA17;__;__ |
| Minerotrophic | D_0__Archaea;D_1__Euryarchaeota;D_2__Methanomicrobia;D_3__Methanosarcinales;D_4__Methanosarcinaceae | D_0__Bacteria;D_1__Firmicutes;D_2__Bacilli;D_3__Bacillales;D_4__Family XII |
| Minerotrophic | D_0__Bacteria;D_1__Proteobacteria;D_2__Deltaproteobacteria;D_3__Sva0485;__ | D_0__Bacteria;D_1__Proteobacteria;D_2__Gammaproteobacteria;D_3__Methylococcales;D_4__Methylococcaceae |
| Minerotrophic | D_0__Archaea;D_1__Euryarchaeota;D_2__Methanobacteria;D_3__Methanobacteriales;D_4__Methanobacteriaceae | D_0__Bacteria;D_1__Chlamydiae;D_2__LD1-PA32;D_3__uncultured bacterium;D_4__uncultured bacterium |
| Minerotrophic | D_0__Archaea;D_1__Euryarchaeota;D_2__Methanomicrobia;D_3__Methanomicrobiales;D_4__Methanoregulaceae | D_0__Bacteria;D_1__Proteobacteria;D_2__Deltaproteobacteria;D_3__Deltaproteobacteria Incertae Sedis;D_4__Syntrophorhabdaceae |
| Minerotrophic | D_0__Archaea;D_1__Euryarchaeota;D_2__Methanomicrobia;D_3__Methanosarcinales;__ | D_0__Bacteria;D_1__Bacteroidetes;__;__;__ |
| Minerotrophic | D_0__Archaea;D_1__Euryarchaeota;D_2__Methanomicrobia;D_3__Methanosarcinales;__ | D_0__Bacteria;D_1__Actinobacteria;D_2__Thermoleophilia;D_3__Solirubrobacterales;D_4__0319-6M6 |
| Minerotrophic | D_0__Archaea;D_1__Euryarchaeota;D_2__Methanomicrobia;D_3__Methanosarcinales;D_4__Methanosaetaceae | D_0__Bacteria;D_1__Planctomycetes;D_2__Phycisphaerae;D_3__Phycisphaerales;__ |
| Minerotrophic | D_0__Archaea;D_1__Bathyarchaeota;Ambiguous_taxa;Ambiguous_taxa;Ambiguous_taxa | D_0__Archaea;D_1__Euryarchaeota;D_2__Methanomicrobia;D_3__Methanomicrobiales;D_4__Methanomicrobiaceae |
| Minerotrophic | D_0__Bacteria;D_1__Proteobacteria;D_2__Alphaproteobacteria;D_3__Rhodospirillales;D_4__DA111 | D_0__Bacteria;D_1__Proteobacteria;D_2__Gammaproteobacteria;D_3__Methylococcales;D_4__Methylococcaceae |
| Minerotrophic | D_0__Archaea;D_1__Candidate division YNPFFA;__;__;__ | D_0__Archaea;D_1__Euryarchaeota;D_2__Methanomicrobia;D_3__Methanomicrobiales;D_4__Methanomicrobiaceae |
| Minerotrophic | D_0__Archaea;D_1__Euryarchaeota;D_2__Methanomicrobia;D_3__Methanomicrobiales;D_4__Methanoregulaceae | D_0__Bacteria;D_1__Chloroflexi;D_2__Dehalococcoidia;__;__ |
| Minerotrophic | D_0__Archaea;D_1__Euryarchaeota;D_2__Methanomicrobia;D_3__Methanomicrobiales;D_4__Methanoregulaceae | D_0__Bacteria;D_1__Ignavibacteriae;D_2__Ignavibacteria;D_3__Ignavibacteriales;__ |
| Minerotrophic | D_0__Archaea;D_1__Euryarchaeota;D_2__Methanomicrobia;D_3__Methanosarcinales;D_4__GOM Arc I | D_0__Bacteria;D_1__SBR1093;D_2__uncultured bacterium;D_3__uncultured bacterium;D_4__uncultured bacterium |
| Minerotrophic | D_0__Archaea;D_1__Euryarchaeota;D_2__Methanobacteria;D_3__Methanobacteriales;D_4__Methanobacteriaceae | D_0__Bacteria;D_1__Proteobacteria;D_2__Gammaproteobacteria;D_3__Xanthomonadales;D_4__Xanthomonadaceae |
| Minerotrophic | D_0__Archaea;D_1__Euryarchaeota;D_2__Methanomicrobia;D_3__Methanosarcinales;D_4__Methanosarcinaceae | D_0__Bacteria;D_1__Proteobacteria;D_2__Betaproteobacteria;D_3__Methylophilales;D_4__Methylophilaceae |
| Minerotrophic | D_0__Archaea;D_1__Euryarchaeota;D_2__Methanomicrobia;D_3__Methanosarcinales;D_4__GOM Arc I | D_0__Bacteria;D_1__Chloroflexi;D_2__Ktedonobacteria;D_3__Ktedonobacterales;__ |
| Minerotrophic | D_0__Archaea;D_1__Euryarchaeota;D_2__Methanomicrobia;D_3__Methanosarcinales;D_4__Methanosarcinaceae | D_0__Bacteria;D_1__Aminicenantes;__;__;__ |
| Minerotrophic | D_0__Archaea;D_1__Euryarchaeota;D_2__Methanomicrobia;D_3__Methanosarcinales;__ | D_0__Bacteria;D_1__Actinobacteria;D_2__Actinobacteria;D_3__Kineosporiales;D_4__Kineosporiaceae |
| Minerotrophic | D_0__Archaea;D_1__Euryarchaeota;D_2__Methanomicrobia;D_3__Methanomicrobiales;D_4__Methanoregulaceae | D_0__Bacteria;D_1__Firmicutes;D_2__Clostridia;D_3__Clostridiales;D_4__Christensenellaceae |
| Minerotrophic | D_0__Archaea;D_1__Euryarchaeota;D_2__Methanomicrobia;D_3__Methanomicrobiales;D_4__Methanospirillaceae | D_0__Bacteria;D_1__Proteobacteria;D_2__Alphaproteobacteria;D_3__Caulobacterales;D_4__Caulobacteraceae |
| Minerotrophic | D_0__Bacteria;D_1__Proteobacteria;D_2__Gammaproteobacteria;D_3__Methylococcales;D_4__Methylococcaceae | D_0__Bacteria;D_1__Saccharibacteria;D_2__uncultured bacterium;D_3__uncultured bacterium;D_4__uncultured bacterium |
| Minerotrophic | D_0__Archaea;D_1__Euryarchaeota;D_2__Methanomicrobia;D_3__Methanosarcinales;D_4__GOM Arc I | D_0__Bacteria;D_1__Proteobacteria;D_2__Alphaproteobacteria;D_3__Rickettsiales;D_4__Rickettsiales Incertae Sedis |
| Minerotrophic | D_0__Archaea;D_1__Euryarchaeota;D_2__Methanomicrobia;D_3__Methanosarcinales;D_4__Methanosarcinaceae | D_0__Bacteria;D_1__Chloroflexi;D_2__P2-11E;D_3__uncultured bacterium;D_4__uncultured bacterium |
| Minerotrophic | D_0__Archaea;D_1__Euryarchaeota;D_2__Methanobacteria;D_3__Methanobacteriales;D_4__Methanobacteriaceae | D_0__Bacteria;D_1__Spirochaetae;D_2__Spirochaetes;D_3__Spirochaetales;D_4__Spirochaetaceae |
| Minerotrophic | D_0__Archaea;D_1__Euryarchaeota;D_2__Methanomicrobia;D_3__Methanosarcinales;D_4__Methanosaetaceae | D_0__Bacteria;D_1__Proteobacteria;D_2__Deltaproteobacteria;D_3__Oligoflexales;D_4__0319-6G20 |
| Minerotrophic | D_0__Archaea;D_1__Euryarchaeota;D_2__Methanomicrobia;D_3__Methanosarcinales;D_4__Methanosarcinaceae | D_0__Bacteria;D_1__Actinobacteria;D_2__Acidimicrobiia;D_3__Acidimicrobiales;D_4__Acidimicrobiales Incertae Sedis |
| Minerotrophic | D_0__Archaea;D_1__Euryarchaeota;D_2__Methanomicrobia;D_3__Methanomicrobiales;D_4__Methanoregulaceae | D_0__Bacteria;D_1__Ignavibacteriae;D_2__Ignavibacteria;D_3__Ignavibacteriales;D_4__SR-FBR-L83 |
| Minerotrophic | D_0__Archaea;D_1__Euryarchaeota;D_2__Methanomicrobia;D_3__Methanosarcinales;__ | D_0__Bacteria;D_1__Chloroflexi;D_2__SJA-68;D_3__uncultured bacterium;D_4__uncultured bacterium |
| Minerotrophic | D_0__Bacteria;D_1__Elusimicrobia;D_2__Elusimicrobia;D_3__Lineage IIa;D_4__uncultured bacterium | D_0__Bacteria;D_1__Proteobacteria;D_2__Gammaproteobacteria;D_3__Methylococcales;D_4__Methylococcaceae |
| Minerotrophic | D_0__Archaea;D_1__Euryarchaeota;D_2__Methanobacteria;D_3__Methanobacteriales;D_4__Methanobacteriaceae | D_0__Bacteria;D_1__Actinobacteria;D_2__Thermoleophilia;D_3__Solirubrobacterales;D_4__Patulibacteraceae |
| Minerotrophic | D_0__Bacteria;D_1__Proteobacteria;D_2__Gammaproteobacteria;D_3__Methylococcales;D_4__Methylococcaceae | D_0__Bacteria;D_1__Verrucomicrobia;D_2__S-BQ2-57 soil group;D_3__uncultured bacterium;D_4__uncultured bacterium |
| Minerotrophic | D_0__Archaea;D_1__Euryarchaeota;D_2__Methanomicrobia;D_3__Methanosarcinales;D_4__GOM Arc I | D_0__Bacteria;D_1__Bacteroidetes;D_2__Sphingobacteriia;D_3__Sphingobacteriales;__ |
| Minerotrophic | D_0__Archaea;D_1__Euryarchaeota;D_2__Methanomicrobia;D_3__Methanomicrobiales;D_4__Methanospirillaceae | D_0__Bacteria;D_1__Actinobacteria;D_2__Actinobacteria;D_3__Micromonosporales;D_4__Micromonosporaceae |
| Minerotrophic | D_0__Archaea;D_1__Euryarchaeota;D_2__Methanomicrobia;D_3__Methanosarcinales;D_4__Methanosaetaceae | D_0__Bacteria;D_1__PAUC34f;__;__;__ |
| Minerotrophic | D_0__Bacteria;D_1__Proteobacteria;D_2__Alphaproteobacteria;D_3__Rhizobiales;D_4__Methylocystaceae | D_0__Bacteria;D_1__Proteobacteria;D_2__Deltaproteobacteria;D_3__Oligoflexales;D_4__0319-6G20 |
| Minerotrophic | D_0__Archaea;D_1__Euryarchaeota;D_2__Methanomicrobia;D_3__Methanosarcinales;__ | D_0__Bacteria;D_1__Ignavibacteriae;D_2__Ignavibacteria;D_3__Ignavibacteriales;D_4__BSV26 |
| Minerotrophic | D_0__Archaea;D_1__Euryarchaeota;D_2__Methanomicrobia;D_3__Methanosarcinales;__ | D_0__Bacteria;D_1__Firmicutes;D_2__Bacilli;D_3__Bacillales;D_4__Paenibacillaceae |
| Minerotrophic | D_0__Archaea;D_1__Euryarchaeota;D_2__Methanomicrobia;D_3__Methanomicrobiales;D_4__Methanospirillaceae | D_0__Bacteria;D_1__Firmicutes;D_2__Bacilli;D_3__Lactobacillales;D_4__Leuconostocaceae |
| Minerotrophic | D_0__Archaea;D_1__Euryarchaeota;D_2__Methanomicrobia;D_3__Methanosarcinales;D_4__GOM Arc I | D_0__Bacteria;D_1__Firmicutes;D_2__Clostridia;D_3__Clostridiales;D_4__Peptococcaceae |
| Minerotrophic | D_0__Archaea;D_1__Euryarchaeota;D_2__Methanobacteria;D_3__Methanobacteriales;D_4__Methanobacteriaceae | D_0__Bacteria;D_1__Actinobacteria;D_2__Acidimicrobiia;D_3__Acidimicrobiales;D_4__Acidimicrobiales Incertae Sedis |
| Minerotrophic | D_0__Bacteria;D_1__Proteobacteria;D_2__Deltaproteobacteria;D_3__Myxococcales;D_4__Blfdi19 | D_0__Bacteria;D_1__Proteobacteria;D_2__Gammaproteobacteria;D_3__Methylococcales;D_4__Methylococcaceae |
| Minerotrophic | D_0__Archaea;D_1__Euryarchaeota;D_2__Methanobacteria;D_3__Methanobacteriales;D_4__Methanobacteriaceae | D_0__Bacteria;D_1__Actinobacteria;D_2__Actinobacteria;D_3__Micrococcales;D_4__Intrasporangiaceae |
| Minerotrophic | D_0__Archaea;D_1__Euryarchaeota;D_2__Methanomicrobia;D_3__Methanomicrobiales;D_4__Methanospirillaceae | D_0__Bacteria;D_1__Actinobacteria;D_2__Acidimicrobiia;D_3__Acidimicrobiales;D_4__uncultured |
| Minerotrophic | D_0__Archaea;D_1__Euryarchaeota;D_2__Methanobacteria;D_3__Methanobacteriales;D_4__Methanobacteriaceae | D_0__Bacteria;D_1__RBG-1 (Zixibacteria);D_2__uncultured bacterium;D_3__uncultured bacterium;D_4__uncultured bacterium |
| Minerotrophic | D_0__Bacteria;D_1__Microgenomates;D_2__uncultured bacterium;D_3__uncultured bacterium;D_4__uncultured bacterium | D_0__Bacteria;D_1__Proteobacteria;D_2__Gammaproteobacteria;D_3__Methylococcales;D_4__Methylococcaceae |
| Minerotrophic | D_0__Archaea;D_1__Euryarchaeota;D_2__Methanomicrobia;D_3__Methanosarcinales;D_4__GOM Arc I | D_0__Bacteria;D_1__Parcubacteria;D_2__uncultured bacterium;D_3__uncultured bacterium;D_4__uncultured bacterium |
| Minerotrophic | D_0__Bacteria;D_1__Proteobacteria;D_2__Betaproteobacteria;D_3__Nitrosomonadales;D_4__Nitrosomonadaceae | D_0__Bacteria;D_1__Proteobacteria;D_2__Gammaproteobacteria;D_3__Methylococcales;D_4__Methylococcaceae |
| Minerotrophic | D_0__Archaea;D_1__Euryarchaeota;D_2__Methanomicrobia;D_3__Methanosarcinales;__ | D_0__Bacteria;D_1__Firmicutes;D_2__Bacilli;D_3__Bacillales;D_4__Bacillaceae |
| Minerotrophic | D_0__Archaea;D_1__Euryarchaeota;D_2__Methanobacteria;D_3__Methanobacteriales;D_4__Methanobacteriaceae | D_0__Bacteria;D_1__LCP-89;D_2__uncultured bacterium;D_3__uncultured bacterium;D_4__uncultured bacterium |
| Minerotrophic | D_0__Archaea;D_1__Euryarchaeota;D_2__Methanomicrobia;D_3__Methanosarcinales;D_4__Methanosaetaceae | D_0__Bacteria;D_1__Firmicutes;D_2__Clostridia;D_3__Clostridiales;D_4__Lachnospiraceae |
| Minerotrophic | D_0__Archaea;D_1__Euryarchaeota;D_2__Methanomicrobia;D_3__Methanosarcinales;__ | D_0__Bacteria;D_1__Bacteroidetes;D_2__Bacteroidia;D_3__Bacteroidales;D_4__Prolixibacteraceae |
| Minerotrophic | D_0__Archaea;D_1__Euryarchaeota;D_2__Methanomicrobia;D_3__Methanosarcinales;__ | D_0__Bacteria;D_1__Chloroflexi;D_2__uncultured;__;__ |
| Minerotrophic | D_0__Archaea;D_1__Euryarchaeota;D_2__Methanobacteria;D_3__Methanobacteriales;D_4__Methanobacteriaceae | D_0__Archaea;D_1__Lokiarchaeota;Ambiguous_taxa;Ambiguous_taxa;Ambiguous_taxa |
| Minerotrophic | D_0__Archaea;D_1__Euryarchaeota;D_2__Methanomicrobia;D_3__Methanosarcinales;__ | D_0__Bacteria;D_1__Proteobacteria;D_2__Deltaproteobacteria;D_3__Myxococcales;D_4__Haliangiaceae |
| Minerotrophic | D_0__Archaea;D_1__Euryarchaeota;D_2__Methanobacteria;D_3__Methanobacteriales;D_4__Methanobacteriaceae | D_0__Bacteria;D_1__Bacteroidetes;D_2__Bacteroidetes vadinHA17;D_3__uncultured bacterium;D_4__uncultured bacterium |
| Minerotrophic | D_0__Archaea;D_1__Euryarchaeota;D_2__Methanomicrobia;D_3__Methanosarcinales;D_4__Methanosaetaceae | D_0__Bacteria;D_1__Planctomycetes;D_2__Phycisphaerae;D_3__Pla1 lineage;__ |
| Minerotrophic | D_0__Archaea;D_1__Euryarchaeota;D_2__Methanomicrobia;D_3__Methanosarcinales;D_4__Methanosaetaceae | D_0__Bacteria;D_1__Ignavibacteriae;D_2__Ignavibacteria;D_3__Ignavibacteriales;D_4__PHOS-HE36 |
| Minerotrophic | D_0__Archaea;D_1__Euryarchaeota;D_2__Methanomicrobia;D_3__Methanomicrobiales;D_4__Methanoregulaceae | D_0__Bacteria;D_1__Chloroflexi;D_2__Dehalococcoidia;D_3__GIF9;__ |
| Minerotrophic | D_0__Bacteria;D_1__Actinobacteria;D_2__Thermoleophilia;D_3__Solirubrobacterales;D_4__Solirubrobacteraceae | D_0__Bacteria;D_1__Proteobacteria;D_2__Gammaproteobacteria;D_3__Methylococcales;D_4__Methylococcaceae |
| Minerotrophic | D_0__Archaea;D_1__Euryarchaeota;D_2__Methanomicrobia;D_3__Methanomicrobiales;D_4__Methanoregulaceae | D_0__Bacteria;D_1__Planctomycetes;D_2__Phycisphaerae;__;__ |
| Minerotrophic | D_0__Archaea;D_1__Euryarchaeota;D_2__Methanomicrobia;D_3__Methanosarcinales;D_4__Methanosaetaceae | D_0__Bacteria;D_1__Bacteroidetes;D_2__Sphingobacteriia;D_3__Sphingobacteriales;D_4__Lentimicrobiaceae |
| Minerotrophic | D_0__Archaea;D_1__Bathyarchaeota;Ambiguous_taxa;Ambiguous_taxa;Ambiguous_taxa | D_0__Archaea;D_1__Euryarchaeota;D_2__Methanomicrobia;D_3__Methanocellales;D_4__Methanocellaceae |
| Minerotrophic | D_0__Bacteria;D_1__Chloroflexi;D_2__Thermomicrobia;D_3__AKYG1722;D_4__uncultured bacterium | D_0__Bacteria;D_1__Proteobacteria;D_2__Gammaproteobacteria;D_3__Methylococcales;D_4__Methylococcaceae |
| Minerotrophic | D_0__Bacteria;D_1__Planctomycetes;D_2__Phycisphaerae;D_3__Phycisphaerales;D_4__Phycisphaeraceae | D_0__Bacteria;D_1__Proteobacteria;D_2__Gammaproteobacteria;D_3__Methylococcales;D_4__Methylococcaceae |
| Minerotrophic | D_0__Bacteria;D_1__Proteobacteria;D_2__Betaproteobacteria;D_3__uncultured;D_4__uncultured bacterium | D_0__Bacteria;D_1__Proteobacteria;D_2__Gammaproteobacteria;D_3__Methylococcales;D_4__Methylococcaceae |
| Minerotrophic | D_0__Archaea;D_1__Euryarchaeota;D_2__Methanomicrobia;D_3__Methanomicrobiales;D_4__Methanoregulaceae | D_0__Bacteria;D_1__RBG-1 (Zixibacteria);D_2__uncultured bacterium;D_3__uncultured bacterium;D_4__uncultured bacterium |
| Minerotrophic | D_0__Bacteria;D_1__Proteobacteria;D_2__Gammaproteobacteria;D_3__Methylococcales;D_4__Methylococcaceae | D_0__Bacteria;D_1__Proteobacteria;D_2__Gammaproteobacteria;D_3__uncultured;__ |
| Minerotrophic | D_0__Archaea;D_1__Euryarchaeota;D_2__Methanomicrobia;D_3__Methanosarcinales;D_4__GOM Arc I | D_0__Bacteria;D_1__Chloroflexi;D_2__Ktedonobacteria;D_3__Ktedonobacterales;D_4__Ktedonobacteraceae |
| Minerotrophic | D_0__Archaea;D_1__Euryarchaeota;D_2__Methanomicrobia;D_3__Methanomicrobiales;D_4__Methanoregulaceae | D_0__Bacteria;D_1__Ignavibacteriae;D_2__Ignavibacteria;D_3__Ignavibacteriales;D_4__BSV26 |
| Minerotrophic | D_0__Archaea;D_1__Euryarchaeota;D_2__Methanomicrobia;D_3__Methanosarcinales;D_4__Methanosaetaceae | D_0__Bacteria;D_1__Firmicutes;D_2__Bacilli;D_3__Bacillales;D_4__Bacillaceae |
| Minerotrophic | D_0__Archaea;D_1__Euryarchaeota;D_2__Methanomicrobia;D_3__Methanosarcinales;D_4__Methanosaetaceae | D_0__Bacteria;D_1__Actinobacteria;D_2__OPB41;D_3__uncultured bacterium;D_4__uncultured bacterium |
| Minerotrophic | D_0__Archaea;D_1__Euryarchaeota;D_2__Methanomicrobia;D_3__Methanomicrobiales;D_4__Methanoregulaceae | D_0__Bacteria;D_1__Cyanobacteria;D_2__ML635J-21;D_3__uncultured bacterium;D_4__uncultured bacterium |
| Minerotrophic | D_0__Archaea;D_1__Euryarchaeota;D_2__Methanomicrobia;D_3__Methanomicrobiales;D_4__Methanospirillaceae | D_0__Bacteria;D_1__Proteobacteria;D_2__Betaproteobacteria;D_3__Methylophilales;D_4__Methylophilaceae |
| Minerotrophic | D_0__Archaea;D_1__Euryarchaeota;D_2__Methanomicrobia;D_3__Methanomicrobiales;D_4__Methanospirillaceae | D_0__Archaea;D_1__Euryarchaeota;D_2__Methanomicrobia;D_3__Methanosarcinales;D_4__Methanosarcinaceae |
| Minerotrophic | D_0__Bacteria;D_1__Ignavibacteriae;D_2__Ignavibacteria;D_3__Ignavibacteriales;D_4__PHOS-HE36 | D_0__Bacteria;D_1__Proteobacteria;D_2__Alphaproteobacteria;D_3__Rhizobiales;D_4__Methylocystaceae |
| Minerotrophic | D_0__Archaea;D_1__Euryarchaeota;D_2__Methanomicrobia;D_3__Methanomicrobiales;D_4__Methanoregulaceae | D_0__Bacteria;D_1__Proteobacteria;D_2__Alphaproteobacteria;D_3__Rhizobiales;D_4__Rhizobiales Incertae Sedis |
| Minerotrophic | D_0__Archaea;D_1__Euryarchaeota;D_2__Methanomicrobia;D_3__Methanosarcinales;D_4__Methanosaetaceae | D_0__Bacteria;D_1__Actinobacteria;D_2__Actinobacteria;D_3__Frankiales;D_4__Sporichthyaceae |
| Minerotrophic | D_0__Archaea;D_1__Euryarchaeota;D_2__Methanomicrobia;D_3__Methanosarcinales;D_4__Methanosaetaceae | D_0__Bacteria;D_1__Ignavibacteriae;D_2__Ignavibacteria;D_3__Ignavibacteriales;D_4__BSV26 |
| Minerotrophic | D_0__Archaea;D_1__Euryarchaeota;D_2__Methanobacteria;D_3__Methanobacteriales;D_4__Methanobacteriaceae | D_0__Archaea;D_1__pMC2A209;D_2__uncultured archaeon;D_3__uncultured archaeon;D_4__uncultured archaeon |
| Minerotrophic | D_0__Archaea;D_1__Euryarchaeota;D_2__Methanobacteria;D_3__Methanobacteriales;D_4__Methanobacteriaceae | D_0__Bacteria;D_1__Firmicutes;D_2__Bacilli;D_3__Lactobacillales;D_4__Leuconostocaceae |
| Minerotrophic | D_0__Archaea;D_1__Euryarchaeota;D_2__Methanobacteria;D_3__Methanobacteriales;D_4__Methanobacteriaceae | D_0__Bacteria;D_1__Deinococcus-Thermus;D_2__Deinococci;D_3__KD3-62;D_4__uncultured bacterium |
| Minerotrophic | D_0__Bacteria;D_1__Chloroflexi;D_2__Thermomicrobia;D_3__JG30-KF-CM45;D_4__uncultured bacterium | D_0__Bacteria;D_1__Proteobacteria;D_2__Gammaproteobacteria;D_3__Methylococcales;D_4__Methylococcaceae |
| Minerotrophic | D_0__Archaea;D_1__Euryarchaeota;D_2__Methanobacteria;D_3__Methanobacteriales;D_4__Methanobacteriaceae | D_0__Bacteria;D_1__Latescibacteria;D_2__Latescibacteria Incertae Sedis;D_3__Unknown Order;D_4__Unknown Family |
| Minerotrophic | D_0__Archaea;D_1__Euryarchaeota;D_2__Methanomicrobia;D_3__Methanosarcinales;D_4__GOM Arc I | D_0__Bacteria;D_1__Chloroflexi;D_2__Ktedonobacteria;D_3__Ktedonobacterales;D_4__FCPS473 |
| Minerotrophic | D_0__Bacteria;D_1__Proteobacteria;D_2__Betaproteobacteria;D_3__Burkholderiales;D_4__Alcaligenaceae | D_0__Bacteria;D_1__Proteobacteria;D_2__Gammaproteobacteria;D_3__Methylococcales;D_4__Methylococcaceae |
| Minerotrophic | D_0__Archaea;D_1__Euryarchaeota;D_2__Methanomicrobia;D_3__Methanocellales;D_4__Methanocellaceae | D_0__Bacteria;D_1__Proteobacteria;D_2__Deltaproteobacteria;D_3__Myxococcales;D_4__VHS-B3-70 |
| Minerotrophic | D_0__Archaea;D_1__Euryarchaeota;D_2__Methanomicrobia;D_3__Methanomicrobiales;D_4__Methanoregulaceae | D_0__Bacteria;D_1__Armatimonadetes;__;__;__ |
| Minerotrophic | D_0__Archaea;D_1__Euryarchaeota;D_2__Methanomicrobia;D_3__Methanosarcinales;__ | D_0__Bacteria;D_1__Chloroflexi;D_2__Dehalococcoidia;D_3__vadinBA26;D_4__uncultured bacterium |
| Minerotrophic | D_0__Archaea;D_1__Euryarchaeota;D_2__Methanomicrobia;D_3__Methanomicrobiales;D_4__Methanomicrobiaceae | D_0__Bacteria;D_1__Chloroflexi;D_2__Dehalococcoidia;D_3__GIF3;D_4__uncultured bacterium |
| Minerotrophic | D_0__Archaea;D_1__Euryarchaeota;D_2__Methanobacteria;D_3__Methanobacteriales;D_4__Methanobacteriaceae | D_0__Bacteria;D_1__Hydrogenedentes;D_2__uncultured bacterium;D_3__uncultured bacterium;D_4__uncultured bacterium |
| Minerotrophic | D_0__Archaea;D_1__Euryarchaeota;D_2__Methanomicrobia;D_3__Methanosarcinales;D_4__Methanosaetaceae | D_0__Bacteria;D_1__Proteobacteria;D_2__Deltaproteobacteria;D_3__Deltaproteobacteria Incertae Sedis;D_4__Syntrophorhabdaceae |
| Minerotrophic | D_0__Archaea;D_1__Euryarchaeota;D_2__Methanomicrobia;D_3__Methanomicrobiales;__ | D_0__Bacteria;D_1__Elusimicrobia;D_2__Elusimicrobia;D_3__Lineage IV;__ |
| Minerotrophic | D_0__Archaea;D_1__Euryarchaeota;D_2__Methanomicrobia;D_3__Methanomicrobiales;__ | D_0__Bacteria;D_1__Actinobacteria;D_2__Thermoleophilia;D_3__Gaiellales;__ |
| Minerotrophic | D_0__Bacteria;D_1__Elusimicrobia;D_2__Elusimicrobia;D_3__Lineage IV;D_4__uncultured bacterium | D_0__Bacteria;D_1__Proteobacteria;D_2__Gammaproteobacteria;D_3__Methylococcales;D_4__Methylococcaceae |
| Minerotrophic | D_0__Archaea;D_1__Euryarchaeota;D_2__Methanomicrobia;D_3__Methanosarcinales;D_4__GOM Arc I | D_0__Bacteria;D_1__Chloroflexi;D_2__S085;D_3__uncultured bacterium;D_4__uncultured bacterium |
| Minerotrophic | D_0__Archaea;D_1__Euryarchaeota;D_2__Methanomicrobia;D_3__Methanosarcinales;__ | D_0__Bacteria;D_1__Armatimonadetes;D_2__Chthonomonadetes;D_3__Chthonomonadales;D_4__uncultured bacterium |
| Minerotrophic | D_0__Archaea;D_1__Euryarchaeota;D_2__Methanomicrobia;D_3__Methanomicrobiales;__ | D_0__Bacteria;D_1__Proteobacteria;D_2__Gammaproteobacteria;D_3__Pseudomonadales;D_4__Moraxellaceae |
| Minerotrophic | D_0__Bacteria;D_1__Firmicutes;D_2__Bacilli;D_3__Lactobacillales;D_4__Streptococcaceae | D_0__Bacteria;D_1__Proteobacteria;D_2__Gammaproteobacteria;D_3__Methylococcales;D_4__Methylococcaceae |
| Minerotrophic | D_0__Archaea;D_1__Euryarchaeota;D_2__Methanomicrobia;D_3__Methanosarcinales;D_4__Methanosarcinaceae | D_0__Bacteria;D_1__Spirochaetae;D_2__Spirochaetes;D_3__Spirochaetales;D_4__Spirochaetaceae |
| Minerotrophic | D_0__Archaea;D_1__Euryarchaeota;D_2__Methanomicrobia;D_3__Methanosarcinales;D_4__GOM Arc I | D_0__Bacteria;D_1__Acidobacteria;D_2__Holophagae;D_3__Subgroup 10;D_4__ABS-19 |
| Minerotrophic | D_0__Archaea;D_1__Euryarchaeota;D_2__Methanomicrobia;D_3__Methanosarcinales;__ | D_0__Bacteria;D_1__Firmicutes;D_2__Bacilli;D_3__Lactobacillales;D_4__Leuconostocaceae |
| Minerotrophic | D_0__Archaea;D_1__Euryarchaeota;D_2__Methanomicrobia;D_3__Methanomicrobiales;D_4__Methanoregulaceae | D_0__Bacteria;D_1__Deinococcus-Thermus;D_2__Deinococci;D_3__KD3-62;D_4__uncultured bacterium |
| Minerotrophic | D_0__Bacteria;D_1__Proteobacteria;D_2__Alphaproteobacteria;D_3__Rhizobiales;D_4__Methylocystaceae | D_0__Bacteria;D_1__Proteobacteria;D_2__Deltaproteobacteria;D_3__Deltaproteobacteria Incertae Sedis;D_4__Syntrophorhabdaceae |
| Minerotrophic | D_0__Bacteria;D_1__Proteobacteria;D_2__Betaproteobacteria;D_3__TRA3-20;__ | D_0__Bacteria;D_1__Proteobacteria;D_2__Gammaproteobacteria;D_3__Methylococcales;D_4__Methylococcaceae |
| Minerotrophic | D_0__Archaea;D_1__Euryarchaeota;D_2__Methanobacteria;D_3__Methanobacteriales;D_4__Methanobacteriaceae | D_0__Bacteria;D_1__Planctomycetes;D_2__Phycisphaerae;D_3__mle1-8;D_4__uncultured organism |
| Minerotrophic | D_0__Archaea;D_1__Euryarchaeota;D_2__Methanomicrobia;D_3__Methanosarcinales;D_4__GOM Arc I | D_0__Bacteria;D_1__Chloroflexi;D_2__SHA-26;__;__ |
| Minerotrophic | D_0__Archaea;D_1__Euryarchaeota;D_2__Methanomicrobia;D_3__Methanosarcinales;__ | D_0__Archaea;D_1__Lokiarchaeota;D_2__uncultured archaeon;D_3__uncultured archaeon;D_4__uncultured archaeon |
| Minerotrophic | D_0__Archaea;D_1__Euryarchaeota;D_2__Methanomicrobia;D_3__Methanomicrobiales;D_4__Methanospirillaceae | D_0__Bacteria;D_1__Proteobacteria;D_2__Deltaproteobacteria;D_3__Myxococcales;D_4__VHS-B3-70 |
| Minerotrophic | D_0__Archaea;D_1__Euryarchaeota;D_2__Methanomicrobia;D_3__Methanomicrobiales;D_4__Methanomicrobiaceae | D_0__Archaea;D_1__Euryarchaeota;D_2__Methanomicrobia;D_3__Methanomicrobiales;D_4__Methanospirillaceae |
| Minerotrophic | D_0__Archaea;D_1__Euryarchaeota;D_2__Methanomicrobia;D_3__Methanosarcinales;D_4__Methanosaetaceae | D_0__Archaea;D_1__Euryarchaeota;D_2__Thermoplasmata;D_3__Thermoplasmatales;D_4__Marine Benthic Group D and DHVEG-1 |
| Minerotrophic | D_0__Archaea;D_1__Euryarchaeota;D_2__Methanomicrobia;D_3__Methanomicrobiales;D_4__Methanospirillaceae | D_0__Bacteria;D_1__Actinobacteria;D_2__Actinobacteria;__;__ |
| Minerotrophic | D_0__Bacteria;D_1__Proteobacteria;D_2__Alphaproteobacteria;D_3__Rhodospirillales;D_4__AKYH478 | D_0__Bacteria;D_1__Proteobacteria;D_2__Gammaproteobacteria;D_3__Methylococcales;D_4__Methylococcaceae |
| Minerotrophic | D_0__Bacteria;D_1__Actinobacteria;D_2__Actinobacteria;D_3__Pseudonocardiales;D_4__Pseudonocardiaceae | D_0__Bacteria;D_1__Proteobacteria;D_2__Gammaproteobacteria;D_3__Methylococcales;D_4__Methylococcaceae |
| Minerotrophic | D_0__Archaea;D_1__Euryarchaeota;D_2__Methanomicrobia;D_3__Methanomicrobiales;D_4__Methanoregulaceae | D_0__Bacteria;D_1__Planctomycetes;D_2__Phycisphaerae;D_3__mle1-8;D_4__uncultured organism |
| Minerotrophic | D_0__Archaea;D_1__Euryarchaeota;D_2__Methanomicrobia;D_3__Methanomicrobiales;D_4__Methanoregulaceae | D_0__Bacteria;D_1__Elusimicrobia;D_2__Elusimicrobia;D_3__Lineage I;D_4__Unknown Family |
| Minerotrophic | D_0__Archaea;D_1__Euryarchaeota;D_2__Methanomicrobia;D_3__Methanosarcinales;D_4__GOM Arc I | D_0__Bacteria;D_1__Proteobacteria;D_2__Deltaproteobacteria;D_3__Desulfurellales;D_4__Desulfurellaceae |
| Minerotrophic | D_0__Archaea;D_1__Euryarchaeota;D_2__Methanomicrobia;D_3__Methanosarcinales;D_4__Methanosaetaceae | D_0__Bacteria;D_1__Elusimicrobia;D_2__Elusimicrobia;D_3__Lineage I;D_4__Unknown Family |
| Minerotrophic | D_0__Bacteria;D_1__Proteobacteria;D_2__Betaproteobacteria;D_3__Burkholderiales;__ | D_0__Bacteria;D_1__Proteobacteria;D_2__Gammaproteobacteria;D_3__Methylococcales;D_4__Methylococcaceae |
| Minerotrophic | D_0__Bacteria;D_1__Chloroflexi;D_2__SJA-15;__;__ | D_0__Bacteria;D_1__Proteobacteria;D_2__Alphaproteobacteria;D_3__Rhizobiales;D_4__Methylocystaceae |
| Minerotrophic | D_0__Archaea;D_1__Euryarchaeota;D_2__Methanomicrobia;D_3__Methanosarcinales;D_4__Methanosarcinaceae | D_0__Bacteria;D_1__Bacteroidetes;D_2__Sphingobacteriia;D_3__Sphingobacteriales;D_4__Saprospiraceae |
| Minerotrophic | D_0__Archaea;D_1__Euryarchaeota;D_2__Methanomicrobia;D_3__Methanosarcinales;D_4__GOM Arc I | D_0__Bacteria;D_1__Gemmatimonadetes;D_2__Gemmatimonadetes;D_3__Gemmatimonadales;D_4__Gemmatimonadaceae |
| Minerotrophic | D_0__Bacteria;D_1__Proteobacteria;D_2__Deltaproteobacteria;D_3__SAR324 clade(Marine group B);D_4__uncultured bacterium | D_0__Bacteria;D_1__Proteobacteria;D_2__Gammaproteobacteria;D_3__Methylococcales;D_4__Methylococcaceae |
| Minerotrophic | D_0__Archaea;D_1__Euryarchaeota;D_2__Methanomicrobia;D_3__Methanosarcinales;__ | D_0__Bacteria;D_1__Elusimicrobia;D_2__Elusimicrobia;D_3__Lineage I;D_4__Unknown Family |
| Minerotrophic | D_0__Archaea;D_1__Euryarchaeota;D_2__Methanomicrobia;D_3__Methanosarcinales;__ | D_0__Bacteria;D_1__Armatimonadetes;D_2__uncultured;D_3__uncultured bacterium;D_4__uncultured bacterium |
| Minerotrophic | D_0__Archaea;D_1__Euryarchaeota;D_2__Methanomicrobia;D_3__Methanocellales;D_4__Methanocellaceae | D_0__Bacteria;D_1__Nitrospinae;D_2__MD2898-B26;__;__ |
| Minerotrophic | D_0__Archaea;D_1__Euryarchaeota;D_2__Methanomicrobia;D_3__Methanomicrobiales;D_4__Methanoregulaceae | D_0__Bacteria;D_1__Firmicutes;D_2__Bacilli;D_3__Bacillales;__ |
| Minerotrophic | D_0__Bacteria;D_1__Parcubacteria;D_2__uncultured bacterium;D_3__uncultured bacterium;D_4__uncultured bacterium | D_0__Bacteria;D_1__Proteobacteria;D_2__Gammaproteobacteria;D_3__Methylococcales;D_4__Methylococcaceae |
| Minerotrophic | D_0__Bacteria;D_1__Actinobacteria;D_2__Actinobacteria;D_3__Streptomycetales;D_4__Streptomycetaceae | D_0__Bacteria;D_1__Proteobacteria;D_2__Gammaproteobacteria;D_3__Methylococcales;D_4__Methylococcaceae |
| Minerotrophic | D_0__Archaea;D_1__Euryarchaeota;D_2__Methanomicrobia;D_3__Methanosarcinales;__ | D_0__Bacteria;D_1__Actinobacteria;D_2__Thermoleophilia;D_3__Solirubrobacterales;D_4__Patulibacteraceae |
| Minerotrophic | D_0__Archaea;D_1__Euryarchaeota;D_2__Methanomicrobia;D_3__Methanosarcinales;D_4__GOM Arc I | D_0__Bacteria;D_1__Nitrospirae;D_2__Nitrospira;D_3__Nitrospirales;D_4__Nitrospiraceae |
| Minerotrophic | D_0__Archaea;D_1__Euryarchaeota;D_2__Methanomicrobia;D_3__Methanosarcinales;D_4__GOM Arc I | D_0__Bacteria;D_1__Chloroflexi;D_2__SBR2076;D_3__uncultured bacterium;D_4__uncultured bacterium |
| Minerotrophic | D_0__Archaea;D_1__Euryarchaeota;D_2__Methanomicrobia;D_3__Methanocellales;D_4__Methanocellaceae | D_0__Bacteria;D_1__Firmicutes;D_2__Clostridia;__;__ |
| Minerotrophic | D_0__Archaea;D_1__Euryarchaeota;D_2__Methanobacteria;D_3__Methanobacteriales;D_4__Methanobacteriaceae | D_0__Archaea;D_1__Euryarchaeota;D_2__Methanomicrobia;D_3__Methanomicrobiales;D_4__Methanoregulaceae |
| Minerotrophic | D_0__Archaea;D_1__Euryarchaeota;D_2__Methanobacteria;D_3__Methanobacteriales;D_4__Methanobacteriaceae | D_0__Bacteria;D_1__Acidobacteria;D_2__Holophagae;D_3__TPD-58;D_4__uncultured bacterium |
| Minerotrophic | D_0__Archaea;D_1__Euryarchaeota;D_2__Methanomicrobia;D_3__Methanosarcinales;D_4__Methanosarcinaceae | D_0__Bacteria;D_1__Verrucomicrobia;D_2__WCHB1-41;D_3__uncultured bacterium;D_4__uncultured bacterium |
| Minerotrophic | D_0__Archaea;D_1__Euryarchaeota;D_2__Methanomicrobia;D_3__Methanomicrobiales;D_4__Methanoregulaceae | D_0__Bacteria;D_1__Bacteroidetes;D_2__Sphingobacteriia;D_3__Sphingobacteriales;D_4__Lentimicrobiaceae |
| Minerotrophic | D_0__Archaea;D_1__Euryarchaeota;D_2__Methanobacteria;D_3__Methanobacteriales;D_4__Methanobacteriaceae | D_0__Archaea;D_1__Thaumarchaeota;__;__;__ |
| Minerotrophic | D_0__Archaea;D_1__Euryarchaeota;D_2__Methanomicrobia;D_3__Methanomicrobiales;D_4__Methanoregulaceae | D_0__Bacteria;D_1__Chloroflexi;D_2__Dehalococcoidia;D_3__vadinBA26;D_4__uncultured bacterium |
| Minerotrophic | D_0__Archaea;D_1__Euryarchaeota;D_2__Methanomicrobia;D_3__Methanosarcinales;D_4__GOM Arc I | D_0__Archaea;D_1__Thaumarchaeota;D_2__South African Gold Mine Gp 1(SAGMCG-1);__;__ |
| Minerotrophic | D_0__Archaea;D_1__Euryarchaeota;D_2__Methanomicrobia;D_3__Methanosarcinales;__ | D_0__Bacteria;D_1__Fibrobacteres;D_2__Fibrobacteria;D_3__Fibrobacterales;__ |
| Minerotrophic | D_0__Archaea;D_1__Candidate division YNPFFA;__;__;__ | D_0__Archaea;D_1__Euryarchaeota;D_2__Methanomicrobia;D_3__Methanocellales;D_4__Methanocellaceae |
| Minerotrophic | D_0__Archaea;D_1__Euryarchaeota;D_2__Methanomicrobia;D_3__Methanomicrobiales;D_4__Methanospirillaceae | D_0__Archaea;D_1__pMC2A209;D_2__uncultured archaeon;D_3__uncultured archaeon;D_4__uncultured archaeon |
| Minerotrophic | D_0__Archaea;D_1__Euryarchaeota;D_2__Methanomicrobia;D_3__Methanocellales;D_4__Methanocellaceae | D_0__Bacteria;D_1__CPR2;__;__;__ |
| Minerotrophic | D_0__Archaea;D_1__Euryarchaeota;D_2__Methanobacteria;D_3__Methanobacteriales;D_4__Methanobacteriaceae | D_0__Bacteria;D_1__Proteobacteria;D_2__Deltaproteobacteria;D_3__Bdellovibrionales;D_4__Bacteriovoracaceae |
| Minerotrophic | D_0__Archaea;D_1__Euryarchaeota;D_2__Methanobacteria;D_3__Methanobacteriales;D_4__Methanobacteriaceae | D_0__Bacteria;D_1__Latescibacteria;__;__;__ |
| Minerotrophic | D_0__Archaea;D_1__Euryarchaeota;D_2__Methanomicrobia;D_3__Methanomicrobiales;D_4__Methanomicrobiaceae | D_0__Bacteria;D_1__Proteobacteria;D_2__Deltaproteobacteria;D_3__Desulfovibrionales;D_4__Desulfovibrionaceae |
| Minerotrophic | D_0__Archaea;D_1__Euryarchaeota;D_2__Methanomicrobia;D_3__Methanomicrobiales;D_4__Methanospirillaceae | D_0__Bacteria;D_1__Aminicenantes;D_2__uncultured bacterium;D_3__uncultured bacterium;D_4__uncultured bacterium |
| Minerotrophic | D_0__Archaea;D_1__Euryarchaeota;D_2__Methanomicrobia;D_3__Methanosarcinales;D_4__GOM Arc I | D_0__Bacteria;D_1__Acetothermia;__;__;__ |
| Minerotrophic | D_0__Archaea;D_1__Euryarchaeota;D_2__Methanomicrobia;D_3__Methanosarcinales;D_4__Methanosaetaceae | D_0__Bacteria;D_1__Chloroflexi;D_2__Dehalococcoidia;D_3__vadinBA26;__ |
| Minerotrophic | D_0__Archaea;D_1__Euryarchaeota;D_2__Methanomicrobia;D_3__Methanomicrobiales;D_4__Methanoregulaceae | D_0__Bacteria;D_1__Planctomycetes;D_2__Phycisphaerae;D_3__MSBL9;__ |
| Minerotrophic | D_0__Archaea;D_1__Euryarchaeota;D_2__Methanomicrobia;D_3__Methanomicrobiales;D_4__Methanoregulaceae | D_0__Bacteria;D_1__Proteobacteria;D_2__Alphaproteobacteria;D_3__Rhodospirillales;D_4__Rhodospirillales Incertae Sedis |
| Minerotrophic | D_0__Archaea;D_1__Euryarchaeota;D_2__Methanomicrobia;D_3__Methanosarcinales;D_4__Methanosaetaceae | D_0__Bacteria;D_1__Armatimonadetes;D_2__uncultured;__;__ |
| Minerotrophic | D_0__Archaea;D_1__Euryarchaeota;D_2__Methanomicrobia;D_3__Methanosarcinales;D_4__Methanosarcinaceae | D_0__Bacteria;D_1__Cyanobacteria;D_2__Melainabacteria;D_3__Gastranaerophilales;D_4__uncultured organism |
| Minerotrophic | D_0__Archaea;D_1__Euryarchaeota;D_2__Methanomicrobia;D_3__Methanosarcinales;__ | D_0__Bacteria;D_1__Aminicenantes;D_2__uncultured bacterium;D_3__uncultured bacterium;D_4__uncultured bacterium |
| Minerotrophic | D_0__Bacteria;D_1__Chloroflexi;D_2__Dehalococcoidia;D_3__vadinBA26;D_4__uncultured bacterium | D_0__Bacteria;D_1__Proteobacteria;D_2__Alphaproteobacteria;D_3__Rhizobiales;D_4__Methylocystaceae |
| Minerotrophic | D_0__Archaea;D_1__Euryarchaeota;D_2__Methanobacteria;D_3__Methanobacteriales;D_4__Methanobacteriaceae | D_0__Bacteria;D_1__Chloroflexi;D_2__Dehalococcoidia;D_3__GIF3;D_4__uncultured bacterium |
| Minerotrophic | D_0__Archaea;D_1__Euryarchaeota;D_2__Methanomicrobia;D_3__Methanosarcinales;D_4__Methanosarcinaceae | D_0__Bacteria;D_1__Chloroflexi;D_2__Dehalococcoidia;D_3__GIF3;D_4__uncultured bacterium |
| Minerotrophic | D_0__Archaea;D_1__Euryarchaeota;D_2__Methanobacteria;D_3__Methanobacteriales;D_4__Methanobacteriaceae | D_0__Bacteria;D_1__Actinobacteria;D_2__Thermoleophilia;D_3__Solirubrobacterales;D_4__0319-6M6 |
| Minerotrophic | D_0__Bacteria;D_1__Elusimicrobia;D_2__Elusimicrobia;D_3__Lineage IIa;__ | D_0__Bacteria;D_1__Proteobacteria;D_2__Gammaproteobacteria;D_3__Methylococcales;D_4__Methylococcaceae |
| Minerotrophic | D_0__Archaea;D_1__Woesearchaeota (DHVEG-6);D_2__uncultured euryarchaeote;D_3__uncultured euryarchaeote;D_4__uncultured euryarchaeote | D_0__Bacteria;D_1__Proteobacteria;D_2__Gammaproteobacteria;D_3__Methylococcales;D_4__Methylococcaceae |
| Minerotrophic | D_0__Archaea;D_1__Euryarchaeota;D_2__Methanobacteria;D_3__Methanobacteriales;D_4__Methanobacteriaceae | D_0__Bacteria;D_1__Proteobacteria;D_2__Alphaproteobacteria;__;__ |
| Minerotrophic | D_0__Bacteria;D_1__Chloroflexi;D_2__Dehalococcoidia;D_3__Dehalococcoidales;D_4__uncultured | D_0__Bacteria;D_1__Proteobacteria;D_2__Alphaproteobacteria;D_3__Rhizobiales;D_4__Methylocystaceae |
| Minerotrophic | D_0__Archaea;D_1__Euryarchaeota;D_2__Methanomicrobia;D_3__Methanomicrobiales;D_4__Methanoregulaceae | D_0__Bacteria;D_1__Proteobacteria;D_2__Alphaproteobacteria;D_3__Rhizobiales;D_4__A0839 |
| Minerotrophic | D_0__Archaea;D_1__Euryarchaeota;D_2__Methanomicrobia;D_3__Methanosarcinales;D_4__GOM Arc I | D_0__Bacteria;D_1__Tectomicrobia;D_2__uncultured bacterium;D_3__uncultured bacterium;D_4__uncultured bacterium |
| Minerotrophic | D_0__Archaea;D_1__Euryarchaeota;D_2__Methanobacteria;D_3__Methanobacteriales;D_4__Methanobacteriaceae | D_0__Bacteria;D_1__Proteobacteria;D_2__Betaproteobacteria;D_3__Methylophilales;D_4__Methylophilaceae |
| Minerotrophic | D_0__Archaea;D_1__Euryarchaeota;D_2__Methanomicrobia;D_3__Methanosarcinales;D_4__Methanosaetaceae | D_0__Bacteria;D_1__Planctomycetes;D_2__Phycisphaerae;D_3__mle1-8;D_4__uncultured organism |
| Minerotrophic | D_0__Archaea;D_1__Euryarchaeota;D_2__Methanomicrobia;D_3__Methanosarcinales;__ | D_0__Bacteria;D_1__Firmicutes;D_2__Negativicutes;D_3__Selenomonadales;D_4__Veillonellaceae |
| Minerotrophic | D_0__Archaea;D_1__Euryarchaeota;D_2__Methanomicrobia;D_3__Methanosarcinales;D_4__Methanosaetaceae | D_0__Bacteria;D_1__Chloroflexi;D_2__Dehalococcoidia;D_3__GIF9;__ |
| Minerotrophic | D_0__Archaea;D_1__Euryarchaeota;D_2__Methanomicrobia;D_3__Methanosarcinales;D_4__GOM Arc I | D_0__Bacteria;D_1__Armatimonadetes;D_2__Armatimonadia;D_3__Armatimonadales;D_4__uncultured bacterium |
| Minerotrophic | D_0__Archaea;D_1__Euryarchaeota;D_2__Methanomicrobia;D_3__Methanosarcinales;__ | D_0__Bacteria;D_1__Bacteroidetes;D_2__SB-5;__;__ |
| Minerotrophic | D_0__Archaea;D_1__Euryarchaeota;D_2__Methanobacteria;D_3__Methanobacteriales;D_4__Methanobacteriaceae | D_0__Bacteria;D_1__Planctomycetes;D_2__Phycisphaerae;D_3__Pla1 lineage;__ |
| Minerotrophic | D_0__Archaea;D_1__Euryarchaeota;D_2__Methanomicrobia;D_3__Methanosarcinales;D_4__Methanosaetaceae | D_0__Bacteria;D_1__Proteobacteria;D_2__Alphaproteobacteria;D_3__Rhizobiales;D_4__Methylocystaceae |
| Minerotrophic | D_0__Archaea;D_1__Euryarchaeota;D_2__Methanomicrobia;D_3__Methanocellales;D_4__Methanocellaceae | D_0__Bacteria;D_1__Actinobacteria;D_2__Actinobacteria;D_3__Propionibacteriales;D_4__Nocardioidaceae |
| Minerotrophic | D_0__Bacteria;D_1__Chloroflexi;D_2__Ktedonobacteria;__;__ | D_0__Bacteria;D_1__Proteobacteria;D_2__Gammaproteobacteria;D_3__Methylococcales;D_4__Methylococcaceae |
| Minerotrophic | D_0__Archaea;D_1__Euryarchaeota;D_2__Methanomicrobia;D_3__Methanosarcinales;D_4__Methanosaetaceae | D_0__Bacteria;D_1__Verrucomicrobia;D_2__WCHB1-41;D_3__uncultured bacterium;D_4__uncultured bacterium |
| Minerotrophic | D_0__Archaea;D_1__Euryarchaeota;D_2__Methanomicrobia;D_3__Methanosarcinales;D_4__Methanosaetaceae | D_0__Bacteria;D_1__Proteobacteria;D_2__Deltaproteobacteria;D_3__FW113;__ |
| Minerotrophic | D_0__Bacteria;D_1__Planctomycetes;D_2__Phycisphaerae;D_3__mle1-8;D_4__uncultured organism | D_0__Bacteria;D_1__Proteobacteria;D_2__Alphaproteobacteria;D_3__Rhizobiales;D_4__Methylocystaceae |
| Minerotrophic | D_0__Bacteria;D_1__Proteobacteria;D_2__Betaproteobacteria;D_3__Nitrosomonadales;__ | D_0__Bacteria;D_1__Proteobacteria;D_2__Gammaproteobacteria;D_3__Methylococcales;D_4__Methylococcaceae |
| Minerotrophic | D_0__Archaea;D_1__Euryarchaeota;D_2__Methanomicrobia;D_3__Methanosarcinales;D_4__Methanosaetaceae | D_0__Bacteria;D_1__Chloroflexi;D_2__Anaerolineae;D_3__Anaerolineales;D_4__Anaerolineaceae |
| Minerotrophic | D_0__Archaea;D_1__Euryarchaeota;D_2__Methanomicrobia;D_3__Methanosarcinales;__ | D_0__Bacteria;D_1__Proteobacteria;D_2__Alphaproteobacteria;D_3__Rhizobiales;D_4__A0839 |
| Minerotrophic | D_0__Bacteria;D_1__Omnitrophica;__;__;__ | D_0__Bacteria;D_1__Proteobacteria;D_2__Gammaproteobacteria;D_3__Methylococcales;D_4__Methylococcaceae |
| Minerotrophic | D_0__Archaea;D_1__Euryarchaeota;D_2__Methanomicrobia;D_3__Methanosarcinales;D_4__GOM Arc I | D_0__Bacteria;D_1__Acidobacteria;D_2__Subgroup 11;D_3__uncultured Acidobacteria bacterium;D_4__uncultured Acidobacteria bacterium |
| Minerotrophic | D_0__Archaea;D_1__Euryarchaeota;D_2__Methanomicrobia;D_3__Methanomicrobiales;D_4__Methanospirillaceae | D_0__Bacteria;D_1__Chloroflexi;D_2__Dehalococcoidia;D_3__GIF9;D_4__uncultured bacterium |
| Minerotrophic | D_0__Archaea;D_1__Euryarchaeota;D_2__Methanomicrobia;D_3__Methanosarcinales;D_4__Methanosaetaceae | D_0__Bacteria;D_1__Fibrobacteres;D_2__Fibrobacteria;D_3__Fibrobacterales;__ |
| Minerotrophic | D_0__Bacteria;D_1__Proteobacteria;D_2__Gammaproteobacteria;D_3__KI89A clade;D_4__uncultured bacterium | D_0__Bacteria;D_1__Proteobacteria;D_2__Gammaproteobacteria;D_3__Methylococcales;D_4__Methylococcaceae |
| Minerotrophic | D_0__Archaea;D_1__Euryarchaeota;D_2__Methanomicrobia;D_3__Methanosarcinales;D_4__GOM Arc I | D_0__Bacteria;D_1__Planctomycetes;D_2__Pla4 lineage;__;__ |
| Minerotrophic | D_0__Archaea;D_1__Euryarchaeota;D_2__Methanobacteria;D_3__Methanobacteriales;D_4__Methanobacteriaceae | D_0__Bacteria;D_1__Chloroflexi;D_2__S085;__;__ |
| Minerotrophic | D_0__Archaea;D_1__Euryarchaeota;D_2__Methanomicrobia;D_3__Methanomicrobiales;D_4__Methanospirillaceae | D_0__Bacteria;D_1__Bacteroidetes;__;__;__ |
| Minerotrophic | D_0__Archaea;D_1__Euryarchaeota;D_2__Methanobacteria;D_3__Methanobacteriales;D_4__Methanobacteriaceae | D_0__Bacteria;D_1__Acidobacteria;D_2__Subgroup 25;__;__ |
| Minerotrophic | D_0__Archaea;D_1__Euryarchaeota;D_2__Methanobacteria;D_3__Methanobacteriales;D_4__Methanobacteriaceae | D_0__Bacteria;D_1__Ignavibacteriae;D_2__Ignavibacteria;D_3__Ignavibacteriales;__ |
| Minerotrophic | D_0__Archaea;D_1__Euryarchaeota;D_2__Methanomicrobia;D_3__Methanosarcinales;__ | D_0__Bacteria;D_1__Actinobacteria;D_2__Thermoleophilia;D_3__Gaiellales;D_4__uncultured |
| Minerotrophic | D_0__Archaea;D_1__Euryarchaeota;D_2__Methanomicrobia;D_3__Methanomicrobiales;D_4__Methanoregulaceae | D_0__Bacteria;D_1__Gemmatimonadetes;D_2__S0134 terrestrial group;D_3__uncultured bacterium;D_4__uncultured bacterium |
| Minerotrophic | D_0__Archaea;D_1__Euryarchaeota;D_2__Methanomicrobia;D_3__Methanosarcinales;__ | D_0__Bacteria;D_1__Proteobacteria;D_2__Deltaproteobacteria;__;__ |
| Minerotrophic | D_0__Archaea;D_1__Euryarchaeota;D_2__Methanomicrobia;D_3__Methanocellales;D_4__Methanocellaceae | D_0__Bacteria;D_1__Bacteroidetes;D_2__Flavobacteriia;D_3__Flavobacteriales;D_4__Flavobacteriaceae |
| Minerotrophic | D_0__Archaea;D_1__Euryarchaeota;D_2__Methanomicrobia;D_3__Methanosarcinales;D_4__Methanosaetaceae | D_0__Bacteria;D_1__Bacteroidetes;D_2__Bacteroidetes vadinHA17;__;__ |
| Minerotrophic | D_0__Archaea;D_1__Euryarchaeota;D_2__Methanomicrobia;D_3__Methanosarcinales;D_4__GOM Arc I | D_0__Bacteria;D_1__Chloroflexi;D_2__Thermomicrobia;__;__ |
| Minerotrophic | D_0__Archaea;D_1__Euryarchaeota;D_2__Methanomicrobia;D_3__Methanosarcinales;D_4__GOM Arc I | D_0__Bacteria;D_1__Proteobacteria;D_2__Gammaproteobacteria;D_3__Pseudomonadales;D_4__Pseudomonadaceae |
| Minerotrophic | D_0__Archaea;D_1__Euryarchaeota;D_2__Methanobacteria;D_3__Methanobacteriales;D_4__Methanobacteriaceae | D_0__Bacteria;D_1__Proteobacteria;D_2__Deltaproteobacteria;D_3__Myxococcales;D_4__VHS-B3-70 |
| Minerotrophic | D_0__Archaea;D_1__Euryarchaeota;D_2__Methanomicrobia;D_3__Methanosarcinales;D_4__GOM Arc I | D_0__Archaea;D_1__Thaumarchaeota;D_2__FHMa11 terrestrial group;__;__ |
| Minerotrophic | D_0__Archaea;D_1__Euryarchaeota;D_2__Methanobacteria;D_3__Methanobacteriales;D_4__Methanobacteriaceae | D_0__Bacteria;D_1__Planctomycetes;D_2__Phycisphaerae;D_3__Phycisphaerales;__ |
| Minerotrophic | D_0__Bacteria;D_1__Proteobacteria;D_2__Alphaproteobacteria;D_3__Rickettsiales;__ | D_0__Bacteria;D_1__Proteobacteria;D_2__Gammaproteobacteria;D_3__Methylococcales;D_4__Methylococcaceae |
| Minerotrophic | D_0__Bacteria;D_1__Proteobacteria;D_2__Betaproteobacteria;D_3__TRA3-20;D_4__uncultured bacterium | D_0__Bacteria;D_1__Proteobacteria;D_2__Gammaproteobacteria;D_3__Methylococcales;D_4__Methylococcaceae |
| Minerotrophic | D_0__Archaea;D_1__Euryarchaeota;D_2__Methanomicrobia;D_3__Methanomicrobiales;D_4__Methanospirillaceae | D_0__Bacteria;D_1__LCP-89;D_2__uncultured bacterium;D_3__uncultured bacterium;D_4__uncultured bacterium |
| Minerotrophic | D_0__Archaea;D_1__Euryarchaeota;D_2__Methanomicrobia;D_3__Methanosarcinales;D_4__Methanosaetaceae | D_0__Bacteria;D_1__Proteobacteria;D_2__Alphaproteobacteria;D_3__Rhizobiales;D_4__Rhizobiales Incertae Sedis |
| Minerotrophic | D_0__Archaea;D_1__Euryarchaeota;D_2__Methanomicrobia;D_3__Methanosarcinales;D_4__Methanosaetaceae | D_0__Bacteria;D_1__Proteobacteria;D_2__Deltaproteobacteria;D_3__Desulfarculales;D_4__Desulfarculaceae |
| Minerotrophic | D_0__Archaea;D_1__Euryarchaeota;D_2__Methanomicrobia;D_3__Methanomicrobiales;D_4__Methanospirillaceae | D_0__Bacteria;D_1__Actinobacteria;D_2__Actinobacteria;D_3__Propionibacteriales;D_4__Nocardioidaceae |
| Minerotrophic | D_0__Archaea;D_1__Euryarchaeota;D_2__Methanobacteria;D_3__Methanobacteriales;D_4__Methanobacteriaceae | D_0__Bacteria;D_1__Armatimonadetes;D_2__uncultured;__;__ |
| Minerotrophic | D_0__Archaea;D_1__Euryarchaeota;D_2__Methanobacteria;D_3__Methanobacteriales;D_4__Methanobacteriaceae | D_0__Bacteria;D_1__PAUC34f;D_2__uncultured bacterium;D_3__uncultured bacterium;D_4__uncultured bacterium |
| Minerotrophic | D_0__Archaea;D_1__Euryarchaeota;D_2__Methanomicrobia;D_3__Methanomicrobiales;D_4__Methanospirillaceae | D_0__Bacteria;D_1__Actinobacteria;D_2__OPB41;__;__ |
| Minerotrophic | D_0__Archaea;D_1__Euryarchaeota;D_2__Methanobacteria;D_3__Methanobacteriales;D_4__Methanobacteriaceae | D_0__Bacteria;D_1__Actinobacteria;D_2__OPB41;D_3__uncultured bacterium;D_4__uncultured bacterium |
| Minerotrophic | D_0__Bacteria;D_1__Actinobacteria;D_2__MB-A2-108;__;__ | D_0__Bacteria;D_1__Proteobacteria;D_2__Gammaproteobacteria;D_3__Methylococcales;D_4__Methylococcaceae |
| Minerotrophic | D_0__Archaea;D_1__Euryarchaeota;D_2__Methanomicrobia;D_3__Methanosarcinales;D_4__GOM Arc I | D_0__Bacteria;D_1__Chloroflexi;D_2__SHA-26;D_3__uncultured bacterium;D_4__uncultured bacterium |
| Minerotrophic | D_0__Archaea;D_1__Euryarchaeota;D_2__Methanomicrobia;D_3__Methanosarcinales;D_4__GOM Arc I | D_0__Bacteria;D_1__Firmicutes;D_2__Bacilli;D_3__Bacillales;D_4__Alicyclobacillaceae |
| Minerotrophic | D_0__Archaea;D_1__Euryarchaeota;D_2__Methanomicrobia;D_3__Methanosarcinales;D_4__Methanosaetaceae | D_0__Bacteria;D_1__Acidobacteria;D_2__Subgroup 25;__;__ |
| Minerotrophic | D_0__Archaea;D_1__Euryarchaeota;D_2__Methanomicrobia;D_3__Methanosarcinales;D_4__GOM Arc I | D_0__Bacteria;D_1__Chloroflexi;D_2__Chloroflexia;D_3__Chloroflexales;D_4__Roseiflexaceae |
| Minerotrophic | D_0__Bacteria;D_1__Proteobacteria;D_2__Gammaproteobacteria;D_3__Acidiferrobacterales;D_4__Acidiferrobacteraceae | D_0__Bacteria;D_1__Proteobacteria;D_2__Gammaproteobacteria;D_3__Methylococcales;D_4__Methylococcaceae |
| Minerotrophic | D_0__Archaea;D_1__Euryarchaeota;D_2__Methanomicrobia;D_3__Methanosarcinales;D_4__Methanosarcinaceae | D_0__Bacteria;D_1__Chloroflexi;D_2__Dehalococcoidia;D_3__GIF9;D_4__uncultured bacterium |
| Minerotrophic | D_0__Archaea;D_1__Euryarchaeota;D_2__Methanomicrobia;D_3__Methanosarcinales;D_4__Methanosaetaceae | D_0__Bacteria;D_1__Proteobacteria;D_2__Alphaproteobacteria;D_3__Rhizobiales;D_4__A0839 |
| Minerotrophic | D_0__Archaea;D_1__Euryarchaeota;D_2__Methanobacteria;D_3__Methanobacteriales;D_4__Methanobacteriaceae | D_0__Bacteria;D_1__Bacteroidetes;D_2__SB-5;D_3__uncultured bacterium;D_4__uncultured bacterium |
| Minerotrophic | D_0__Bacteria;D_1__Omnitrophica;D_2__uncultured bacterium;D_3__uncultured bacterium;D_4__uncultured bacterium | D_0__Bacteria;D_1__Proteobacteria;D_2__Gammaproteobacteria;D_3__Methylococcales;D_4__Methylococcaceae |
| Minerotrophic | D_0__Archaea;D_1__Euryarchaeota;D_2__Methanomicrobia;D_3__Methanosarcinales;D_4__Methanosarcinaceae | D_0__Bacteria;D_1__Actinobacteria;D_2__Coriobacteriia;D_3__Coriobacteriales;D_4__Coriobacteriaceae |
| Minerotrophic | D_0__Archaea;D_1__Euryarchaeota;D_2__Methanomicrobia;D_3__Methanomicrobiales;D_4__Methanoregulaceae | D_0__Bacteria;D_1__Bacteroidetes;D_2__Bacteroidetes vadinHA17;D_3__uncultured bacterium;D_4__uncultured bacterium |
| Minerotrophic | D_0__Bacteria;D_1__Proteobacteria;D_2__Deltaproteobacteria;D_3__Desulfovibrionales;D_4__Desulfovibrionaceae | D_0__Bacteria;D_1__Proteobacteria;D_2__Gammaproteobacteria;D_3__Methylococcales;D_4__Methylococcaceae |
| Minerotrophic | D_0__Bacteria;D_1__Acidobacteria;D_2__Blastocatellia;D_3__Blastocatellales;D_4__Blastocatellaceae (Subgroup 4) | D_0__Bacteria;D_1__Proteobacteria;D_2__Gammaproteobacteria;D_3__Methylococcales;D_4__Methylococcaceae |
| Minerotrophic | D_0__Archaea;D_1__Euryarchaeota;D_2__Methanomicrobia;D_3__Methanosarcinales;D_4__GOM Arc I | D_0__Bacteria;D_1__Nitrospirae;D_2__Nitrospira;D_3__Nitrospirales;D_4__Sh765B-TzT-35 |
| Minerotrophic | D_0__Archaea;D_1__Euryarchaeota;D_2__Methanobacteria;D_3__Methanobacteriales;D_4__Methanobacteriaceae | D_0__Bacteria;D_1__Aminicenantes;D_2__uncultured bacterium;D_3__uncultured bacterium;D_4__uncultured bacterium |
| Minerotrophic | D_0__Archaea;D_1__Euryarchaeota;D_2__Methanomicrobia;D_3__Methanomicrobiales;D_4__Methanoregulaceae | D_0__Bacteria;D_1__Chloroflexi;D_2__SJA-68;D_3__uncultured bacterium;D_4__uncultured bacterium |
| Minerotrophic | D_0__Archaea;D_1__Euryarchaeota;D_2__Methanomicrobia;D_3__Methanomicrobiales;D_4__Methanoregulaceae | D_0__Bacteria;D_1__LCP-89;D_2__uncultured bacterium;D_3__uncultured bacterium;D_4__uncultured bacterium |
| Minerotrophic | D_0__Bacteria;D_1__Cyanobacteria;D_2__Melainabacteria;D_3__Obscuribacterales;D_4__uncultured bacterium | D_0__Bacteria;D_1__Proteobacteria;D_2__Gammaproteobacteria;D_3__Methylococcales;D_4__Methylococcaceae |
| Minerotrophic | D_0__Bacteria;D_1__Chloroflexi;D_2__Ktedonobacteria;D_3__C0119;D_4__uncultured bacterium | D_0__Bacteria;D_1__Proteobacteria;D_2__Gammaproteobacteria;D_3__Methylococcales;D_4__Methylococcaceae |
| Minerotrophic | D_0__Archaea;D_1__Euryarchaeota;D_2__Methanomicrobia;D_3__Methanomicrobiales;D_4__Methanospirillaceae | D_0__Bacteria;D_1__Verrucomicrobia;D_2__WCHB1-41;D_3__uncultured bacterium;D_4__uncultured bacterium |
| Minerotrophic | D_0__Archaea;D_1__Euryarchaeota;D_2__Methanomicrobia;D_3__Methanosarcinales;D_4__Methanosarcinaceae | D_0__Bacteria;D_1__PAUC34f;D_2__uncultured bacterium;D_3__uncultured bacterium;D_4__uncultured bacterium |
| Minerotrophic | D_0__Archaea;D_1__Euryarchaeota;D_2__Methanomicrobia;D_3__Methanosarcinales;D_4__Methanosarcinaceae | D_0__Bacteria;D_1__Actinobacteria;D_2__Acidimicrobiia;D_3__Acidimicrobiales;D_4__Acidimicrobiaceae |
| Minerotrophic | D_0__Archaea;D_1__Euryarchaeota;D_2__Methanomicrobia;D_3__Methanomicrobiales;D_4__Methanoregulaceae | D_0__Bacteria;D_1__Chloroflexi;D_2__Anaerolineae;D_3__Anaerolineales;D_4__Anaerolineaceae |
| Minerotrophic | D_0__Archaea;D_1__Euryarchaeota;D_2__Methanomicrobia;D_3__Methanosarcinales;__ | D_0__Bacteria;D_1__Acidobacteria;D_2__Holophagae;D_3__TPD-58;D_4__uncultured bacterium |
| Minerotrophic | D_0__Bacteria;D_1__Armatimonadetes;D_2__Chthonomonadetes;D_3__Chthonomonadales;D_4__Chthonomonadaceae | D_0__Bacteria;D_1__Proteobacteria;D_2__Gammaproteobacteria;D_3__Methylococcales;D_4__Methylococcaceae |
| Minerotrophic | D_0__Archaea;D_1__Euryarchaeota;D_2__Methanomicrobia;D_3__Methanosarcinales;D_4__Methanosarcinaceae | D_0__Bacteria;D_1__Actinobacteria;D_2__Actinobacteria;D_3__Propionibacteriales;D_4__Nocardioidaceae |
| Minerotrophic | D_0__Archaea;D_1__Euryarchaeota;D_2__Methanomicrobia;D_3__Methanosarcinales;D_4__Methanosaetaceae | D_0__Bacteria;D_1__Proteobacteria;D_2__Gammaproteobacteria;D_3__X35;__ |
| Minerotrophic | D_0__Bacteria;D_1__Acidobacteria;D_2__Subgroup 22;D_3__uncultured bacterium;D_4__uncultured bacterium | D_0__Bacteria;D_1__Proteobacteria;D_2__Gammaproteobacteria;D_3__Methylococcales;D_4__Methylococcaceae |
| Minerotrophic | D_0__Archaea;D_1__Euryarchaeota;D_2__Methanomicrobia;D_3__Methanocellales;D_4__Methanocellaceae | D_0__Bacteria;D_1__Proteobacteria;D_2__Gammaproteobacteria;D_3__Xanthomonadales;D_4__Xanthomonadaceae |
| Minerotrophic | D_0__Archaea;D_1__Euryarchaeota;D_2__Methanomicrobia;D_3__Methanosarcinales;D_4__GOM Arc I | D_0__Bacteria;D_1__GAL15;D_2__uncultured bacterium;D_3__uncultured bacterium;D_4__uncultured bacterium |
| Minerotrophic | D_0__Bacteria;D_1__Acetothermia;D_2__uncultured bacterium;D_3__uncultured bacterium;D_4__uncultured bacterium | D_0__Bacteria;D_1__Proteobacteria;D_2__Gammaproteobacteria;D_3__Methylococcales;D_4__Methylococcaceae |
| Minerotrophic | D_0__Bacteria;D_1__Actinobacteria;D_2__Thermoleophilia;D_3__Gaiellales;D_4__Gaiellaceae | D_0__Bacteria;D_1__Proteobacteria;D_2__Gammaproteobacteria;D_3__Methylococcales;D_4__Methylococcaceae |
| Minerotrophic | D_0__Archaea;D_1__Euryarchaeota;D_2__Methanomicrobia;D_3__Methanosarcinales;D_4__Methanosaetaceae | D_0__Bacteria;D_1__Latescibacteria;D_2__Latescibacteria Incertae Sedis;D_3__Unknown Order;D_4__Unknown Family |
| Minerotrophic | D_0__Archaea;D_1__Euryarchaeota;D_2__Methanomicrobia;D_3__Methanosarcinales;D_4__Methanosaetaceae | D_0__Bacteria;D_1__Actinobacteria;D_2__Actinobacteria;D_3__Micrococcales;D_4__Intrasporangiaceae |
| Minerotrophic | D_0__Archaea;D_1__Euryarchaeota;D_2__Methanomicrobia;D_3__Methanomicrobiales;__ | D_0__Bacteria;D_1__KSB3 (Modulibacteria);D_2__uncultured organism;D_3__uncultured organism;D_4__uncultured organism |
| Minerotrophic | D_0__Archaea;D_1__Euryarchaeota;D_2__Methanobacteria;D_3__Methanobacteriales;D_4__Methanobacteriaceae | D_0__Bacteria;D_1__Proteobacteria;D_2__Alphaproteobacteria;D_3__Caulobacterales;D_4__Caulobacteraceae |
| Minerotrophic | D_0__Archaea;D_1__Aenigmarchaeota;D_2__Deep Sea Euryarchaeotic Group(DSEG);__;__ | D_0__Bacteria;D_1__Proteobacteria;D_2__Alphaproteobacteria;D_3__Rhizobiales;D_4__Methylocystaceae |
| Minerotrophic | D_0__Archaea;D_1__Euryarchaeota;D_2__Methanomicrobia;D_3__Methanocellales;D_4__Methanocellaceae | D_0__Bacteria;D_1__Proteobacteria;D_2__Betaproteobacteria;D_3__Burkholderiales;D_4__Comamonadaceae |
| Minerotrophic | D_0__Archaea;D_1__Euryarchaeota;D_2__Methanomicrobia;D_3__Methanomicrobiales;D_4__Methanospirillaceae | D_0__Bacteria;D_1__Actinobacteria;D_2__Actinobacteria;D_3__Frankiales;D_4__Sporichthyaceae |
| Minerotrophic | D_0__Archaea;D_1__Aigarchaeota;D_2__Terrestrial Hot Spring Gp(THSCG);D_3__uncultured archaeon;D_4__uncultured archaeon | D_0__Archaea;D_1__Euryarchaeota;D_2__Methanomicrobia;D_3__Methanosarcinales;D_4__GOM Arc I |
| Minerotrophic | D_0__Archaea;D_1__Euryarchaeota;D_2__Methanomicrobia;D_3__Methanomicrobiales;D_4__Methanoregulaceae | D_0__Bacteria;D_1__Firmicutes;D_2__Bacilli;D_3__Bacillales;D_4__Bacillaceae |
| Minerotrophic | D_0__Archaea;D_1__Euryarchaeota;D_2__Methanomicrobia;D_3__Methanomicrobiales;D_4__Methanospirillaceae | D_0__Bacteria;D_1__Chloroflexi;D_2__Dehalococcoidia;D_3__GIF3;D_4__uncultured bacterium |
| Minerotrophic | D_0__Bacteria;D_1__Firmicutes;__;__;__ | D_0__Bacteria;D_1__Proteobacteria;D_2__Alphaproteobacteria;D_3__Rhizobiales;D_4__Methylocystaceae |
| Minerotrophic | D_0__Archaea;D_1__Euryarchaeota;D_2__Methanomicrobia;D_3__Methanosarcinales;D_4__GOM Arc I | D_0__Bacteria;D_1__Chlorobi;D_2__Chlorobia;D_3__Chlorobiales;D_4__OPB56 |
| Minerotrophic | D_0__Archaea;D_1__Euryarchaeota;D_2__Methanobacteria;D_3__Methanobacteriales;D_4__Methanobacteriaceae | D_0__Archaea;D_1__Lokiarchaeota;D_2__uncultured archaeon;D_3__uncultured archaeon;D_4__uncultured archaeon |
| Minerotrophic | D_0__Archaea;D_1__Euryarchaeota;D_2__Methanobacteria;D_3__Methanobacteriales;D_4__Methanobacteriaceae | D_0__Bacteria;D_1__Verrucomicrobia;D_2__WCHB1-41;D_3__uncultured bacterium;D_4__uncultured bacterium |
| Minerotrophic | D_0__Bacteria;D_1__Actinobacteria;D_2__Actinobacteria;D_3__Propionibacteriales;D_4__Propionibacteriaceae | D_0__Bacteria;D_1__Proteobacteria;D_2__Gammaproteobacteria;D_3__Methylococcales;D_4__Methylococcaceae |
| Minerotrophic | D_0__Archaea;D_1__Euryarchaeota;D_2__Methanomicrobia;D_3__Methanosarcinales;D_4__GOM Arc I | D_0__Bacteria;D_1__Proteobacteria;D_2__Alphaproteobacteria;D_3__Rhizobiales;D_4__Rhodobiaceae |
| Minerotrophic | D_0__Archaea;D_1__Euryarchaeota;D_2__Methanomicrobia;D_3__Methanosarcinales;D_4__GOM Arc I | D_0__Archaea;D_1__Thaumarchaeota;D_2__FHMa11 terrestrial group;D_3__uncultured archaeon;D_4__uncultured archaeon |
| Minerotrophic | D_0__Archaea;D_1__Euryarchaeota;D_2__Methanobacteria;D_3__Methanobacteriales;D_4__Methanobacteriaceae | D_0__Bacteria;D_1__PAUC34f;__;__;__ |
| Minerotrophic | D_0__Archaea;D_1__Thaumarchaeota;D_2__South African Gold Mine Gp 1(SAGMCG-1);D_3__uncultured archaeon;D_4__uncultured archaeon | D_0__Bacteria;D_1__Proteobacteria;D_2__Gammaproteobacteria;D_3__Methylococcales;D_4__Methylococcaceae |
| Minerotrophic | D_0__Archaea;D_1__Euryarchaeota;D_2__Methanomicrobia;D_3__Methanosarcinales;D_4__Methanosarcinaceae | D_0__Bacteria;D_1__Fibrobacteres;D_2__Fibrobacteria;D_3__Fibrobacterales;D_4__possible family 01 |
| Minerotrophic | D_0__Archaea;D_1__Euryarchaeota;D_2__Methanobacteria;D_3__Methanobacteriales;D_4__Methanobacteriaceae | D_0__Bacteria;D_1__Acidobacteria;D_2__Holophagae;D_3__Holophagales;D_4__Holophagaceae |
| Minerotrophic | D_0__Archaea;D_1__Euryarchaeota;D_2__Methanomicrobia;D_3__Methanosarcinales;D_4__GOM Arc I | D_0__Bacteria;D_1__Firmicutes;D_2__Negativicutes;D_3__Selenomonadales;D_4__uncultured |
| Minerotrophic | D_0__Archaea;D_1__Euryarchaeota;D_2__Methanomicrobia;D_3__Methanomicrobiales;D_4__Methanoregulaceae | D_0__Archaea;D_1__Euryarchaeota;D_2__Methanomicrobia;D_3__Methanosarcinales;D_4__Methanosaetaceae |
| Minerotrophic | D_0__Archaea;D_1__Euryarchaeota;D_2__Methanomicrobia;D_3__Methanosarcinales;__ | D_0__Bacteria;D_1__Actinobacteria;D_2__Actinobacteria;__;__ |
| Minerotrophic | D_0__Archaea;D_1__Euryarchaeota;D_2__Methanobacteria;D_3__Methanobacteriales;D_4__Methanobacteriaceae | D_0__Bacteria;D_1__Proteobacteria;D_2__Deltaproteobacteria;__;__ |
| Minerotrophic | D_0__Archaea;D_1__Euryarchaeota;D_2__Methanomicrobia;D_3__Methanomicrobiales;D_4__Methanospirillaceae | D_0__Bacteria;D_1__Proteobacteria;D_2__Deltaproteobacteria;D_3__Myxococcales;D_4__Archangiaceae |
| Minerotrophic | D_0__Archaea;D_1__Euryarchaeota;D_2__Methanomicrobia;D_3__Methanomicrobiales;D_4__Methanospirillaceae | D_0__Bacteria;D_1__Proteobacteria;D_2__Gammaproteobacteria;D_3__Xanthomonadales;D_4__Xanthomonadaceae |
| Minerotrophic | D_0__Archaea;D_1__Euryarchaeota;D_2__Methanomicrobia;D_3__Methanosarcinales;D_4__Methanosaetaceae | D_0__Bacteria;D_1__Proteobacteria;D_2__Deltaproteobacteria;D_3__Myxococcales;D_4__Myxococcaceae |
| Minerotrophic | D_0__Archaea;D_1__Euryarchaeota;D_2__Methanomicrobia;D_3__Methanosarcinales;D_4__Methanosaetaceae | D_0__Archaea;D_1__Euryarchaeota;D_2__Methanomicrobia;D_3__Methanosarcinales;__ |
| Minerotrophic | D_0__Archaea;D_1__Euryarchaeota;D_2__Methanomicrobia;D_3__Methanocellales;D_4__Methanocellaceae | D_0__Bacteria;D_1__Firmicutes;D_2__Erysipelotrichia;D_3__Erysipelotrichales;D_4__Erysipelotrichaceae |
| Minerotrophic | D_0__Bacteria;D_1__Bacteroidetes;D_2__SM1A07;D_3__uncultured bacterium;D_4__uncultured bacterium | D_0__Bacteria;D_1__Proteobacteria;D_2__Gammaproteobacteria;D_3__Methylococcales;D_4__Methylococcaceae |
| Minerotrophic | D_0__Archaea;D_1__Euryarchaeota;D_2__Methanomicrobia;D_3__Methanosarcinales;D_4__Methanosaetaceae | D_0__Bacteria;D_1__Deferribacteres;D_2__Deferribacteres Incertae Sedis;D_3__Unknown Order;D_4__Unknown Family |
| Minerotrophic | D_0__Bacteria;D_1__Acidobacteria;D_2__Subgroup 25;__;__ | D_0__Bacteria;D_1__Proteobacteria;D_2__Alphaproteobacteria;D_3__Rhizobiales;D_4__Methylocystaceae |
| Minerotrophic | D_0__Archaea;D_1__Euryarchaeota;D_2__Methanobacteria;D_3__Methanobacteriales;D_4__Methanobacteriaceae | D_0__Bacteria;D_1__Proteobacteria;D_2__Gammaproteobacteria;D_3__X35;__ |
| Minerotrophic | D_0__Archaea;D_1__Euryarchaeota;D_2__Methanobacteria;D_3__Methanobacteriales;D_4__Methanobacteriaceae | D_0__Bacteria;D_1__Actinobacteria;D_2__Acidimicrobiia;D_3__Acidimicrobiales;D_4__uncultured |
| Minerotrophic | D_0__Archaea;D_1__Euryarchaeota;D_2__Methanobacteria;D_3__Methanobacteriales;D_4__Methanobacteriaceae | D_0__Archaea;D_1__Thaumarchaeota;D_2__AK59;__;__ |
| Minerotrophic | D_0__Archaea;D_1__Euryarchaeota;D_2__Methanomicrobia;D_3__Methanosarcinales;D_4__Methanosarcinaceae | D_0__Archaea;D_1__Euryarchaeota;D_2__Thermoplasmata;D_3__Thermoplasmatales;D_4__Terrestrial Miscellaneous Gp(TMEG) |
| Minerotrophic | D_0__Archaea;D_1__Euryarchaeota;D_2__Methanomicrobia;D_3__Methanomicrobiales;D_4__Methanospirillaceae | D_0__Bacteria;D_1__Proteobacteria;D_2__Deltaproteobacteria;__;__ |
| Minerotrophic | D_0__Archaea;D_1__Euryarchaeota;D_2__Methanomicrobia;D_3__Methanomicrobiales;D_4__Methanoregulaceae | D_0__Bacteria;D_1__Proteobacteria;D_2__Deltaproteobacteria;__;__ |
| Minerotrophic | D_0__Archaea;D_1__Euryarchaeota;D_2__Methanomicrobia;D_3__Methanosarcinales;D_4__GOM Arc I | D_0__Archaea;D_1__Thaumarchaeota;D_2__Soil Crenarchaeotic Group(SCG);__;__ |
| Minerotrophic | D_0__Archaea;D_1__Euryarchaeota;D_2__Methanomicrobia;D_3__Methanosarcinales;D_4__Methanosaetaceae | D_0__Bacteria;D_1__Chloroflexi;D_2__Dehalococcoidia;D_3__Dehalococcoidales;D_4__uncultured |
| Minerotrophic | D_0__Archaea;D_1__Euryarchaeota;D_2__Methanobacteria;D_3__Methanobacteriales;D_4__Methanobacteriaceae | D_0__Bacteria;D_1__Proteobacteria;D_2__Alphaproteobacteria;D_3__Rhodospirillales;D_4__Rhodospirillales Incertae Sedis |
| Minerotrophic | D_0__Archaea;D_1__Euryarchaeota;D_2__Methanomicrobia;D_3__Methanomicrobiales;D_4__Methanospirillaceae | D_0__Bacteria;D_1__Chloroflexi;D_2__Dehalococcoidia;D_3__GIF9;__ |
| Minerotrophic | D_0__Archaea;D_1__Euryarchaeota;D_2__Methanomicrobia;D_3__Methanosarcinales;__ | D_0__Bacteria;D_1__Chloroflexi;D_2__Anaerolineae;D_3__Anaerolineales;D_4__Anaerolineaceae |
| Minerotrophic | D_0__Bacteria;D_1__Elusimicrobia;D_2__Elusimicrobia;D_3__MVP-88;D_4__uncultured bacterium | D_0__Bacteria;D_1__Proteobacteria;D_2__Gammaproteobacteria;D_3__Methylococcales;D_4__Methylococcaceae |
| Minerotrophic | D_0__Bacteria;D_1__Proteobacteria;D_2__Alphaproteobacteria;D_3__Rhizobiales;D_4__Methylocystaceae | D_0__Bacteria;D_1__Proteobacteria;D_2__Alphaproteobacteria;D_3__Rhodospirillales;D_4__Rhodospirillales Incertae Sedis |
| Minerotrophic | D_0__Archaea;D_1__Euryarchaeota;D_2__Methanomicrobia;D_3__Methanosarcinales;D_4__GOM Arc I | D_0__Archaea;D_1__Euryarchaeota;D_2__Thermoplasmata;D_3__Thermoplasmatales;D_4__ASC21 |
| Minerotrophic | D_0__Archaea;D_1__Euryarchaeota;D_2__Methanomicrobia;D_3__Methanosarcinales;D_4__Methanosaetaceae | D_0__Bacteria;D_1__Actinobacteria;D_2__Thermoleophilia;D_3__Solirubrobacterales;D_4__Patulibacteraceae |
| Minerotrophic | D_0__Bacteria;D_1__Proteobacteria;D_2__Gammaproteobacteria;D_3__Methylococcales;D_4__Methylococcaceae | D_0__Bacteria;D_1__Spirochaetae;D_2__Spirochaetes;D_3__Spirochaetales;D_4__LH041 |
| Minerotrophic | D_0__Archaea;D_1__Euryarchaeota;D_2__Methanomicrobia;D_3__Methanosarcinales;D_4__GOM Arc I | D_0__Bacteria;D_1__Chloroflexi;D_2__Ktedonobacteria;D_3__Ktedonobacterales;D_4__HSB OF53-F07 |
| Minerotrophic | D_0__Archaea;D_1__Euryarchaeota;D_2__Methanomicrobia;D_3__Methanosarcinales;D_4__Methanosarcinaceae | D_0__Bacteria;D_1__WS2;__;__;__ |
| Minerotrophic | D_0__Archaea;D_1__Euryarchaeota;D_2__Methanomicrobia;D_3__Methanosarcinales;D_4__Methanosarcinaceae | D_0__Bacteria;D_1__Proteobacteria;D_2__Deltaproteobacteria;D_3__Bdellovibrionales;D_4__Bacteriovoracaceae |
| Minerotrophic | D_0__Archaea;D_1__Euryarchaeota;D_2__Methanomicrobia;D_3__Methanosarcinales;D_4__Methanosaetaceae | D_0__Bacteria;D_1__Chloroflexi;D_2__uncultured;__;__ |
| Minerotrophic | D_0__Bacteria;D_1__Actinobacteria;D_2__Actinobacteria;D_3__Frankiales;D_4__Acidothermaceae | D_0__Bacteria;D_1__Proteobacteria;D_2__Gammaproteobacteria;D_3__Methylococcales;D_4__Methylococcaceae |
| Minerotrophic | D_0__Archaea;D_1__Euryarchaeota;D_2__Methanomicrobia;D_3__Methanosarcinales;D_4__GOM Arc I | D_0__Bacteria;D_1__Actinobacteria;D_2__Actinobacteria;D_3__Streptomycetales;D_4__Streptomycetaceae |
| Minerotrophic | D_0__Archaea;D_1__Euryarchaeota;D_2__Methanomicrobia;D_3__Methanomicrobiales;D_4__Methanoregulaceae | D_0__Bacteria;__;__;__;__ |
| Minerotrophic | D_0__Archaea;D_1__Euryarchaeota;D_2__Methanobacteria;D_3__Methanobacteriales;D_4__Methanobacteriaceae | D_0__Bacteria;D_1__Actinobacteria;D_2__Coriobacteriia;D_3__Coriobacteriales;D_4__Coriobacteriaceae |
| Minerotrophic | D_0__Archaea;D_1__Euryarchaeota;D_2__Methanomicrobia;D_3__Methanomicrobiales;D_4__Methanomicrobiaceae | D_0__Archaea;D_1__Euryarchaeota;D_2__Methanomicrobia;D_3__Methanosarcinales;D_4__Methanosarcinaceae |
| Minerotrophic | D_0__Archaea;D_1__Euryarchaeota;D_2__Methanomicrobia;D_3__Methanosarcinales;D_4__Methanosarcinaceae | D_0__Bacteria;D_1__Proteobacteria;D_2__Deltaproteobacteria;D_3__Myxococcales;D_4__Archangiaceae |
| Minerotrophic | D_0__Archaea;D_1__Euryarchaeota;D_2__Methanobacteria;D_3__Methanobacteriales;D_4__Methanobacteriaceae | D_0__Archaea;D_1__Euryarchaeota;D_2__Methanomicrobia;D_3__Methanosarcinales;__ |
| Minerotrophic | D_0__Archaea;D_1__Euryarchaeota;D_2__Methanomicrobia;D_3__Methanosarcinales;D_4__Methanosaetaceae | D_0__Bacteria;D_1__Proteobacteria;D_2__Deltaproteobacteria;__;__ |
| Minerotrophic | D_0__Bacteria;D_1__Proteobacteria;D_2__Deltaproteobacteria;D_3__NB1-j;__ | D_0__Bacteria;D_1__Proteobacteria;D_2__Gammaproteobacteria;D_3__Methylococcales;D_4__Methylococcaceae |
| Minerotrophic | D_0__Archaea;D_1__Euryarchaeota;D_2__Methanomicrobia;D_3__Methanosarcinales;D_4__Methanosarcinaceae | D_0__Bacteria;D_1__Proteobacteria;D_2__Gammaproteobacteria;D_3__Pseudomonadales;D_4__Moraxellaceae |
| Minerotrophic | D_0__Archaea;D_1__Euryarchaeota;D_2__Methanomicrobia;D_3__Methanosarcinales;__ | D_0__Bacteria;D_1__Actinobacteria;__;__;__ |
| Minerotrophic | D_0__Bacteria;D_1__Firmicutes;D_2__Clostridia;D_3__Halanaerobiales;D_4__ODP1230B8.23 | D_0__Bacteria;D_1__Proteobacteria;D_2__Alphaproteobacteria;D_3__Rhizobiales;D_4__Methylocystaceae |
| Minerotrophic | D_0__Archaea;D_1__Euryarchaeota;D_2__Methanomicrobia;D_3__Methanosarcinales;__ | D_0__Bacteria;D_1__Actinobacteria;D_2__Actinobacteria;D_3__Micrococcales;D_4__Intrasporangiaceae |
| Minerotrophic | D_0__Archaea;D_1__Euryarchaeota;D_2__Methanomicrobia;D_3__Methanomicrobiales;D_4__Methanoregulaceae | D_0__Bacteria;D_1__Verrucomicrobia;__;__;__ |
| Minerotrophic | D_0__Archaea;D_1__Euryarchaeota;D_2__Methanomicrobia;D_3__Methanomicrobiales;D_4__Methanoregulaceae | D_0__Bacteria;D_1__Chloroflexi;D_2__SJA-15;D_3__uncultured bacterium;D_4__uncultured bacterium |
| Minerotrophic | D_0__Bacteria;D_1__Elusimicrobia;D_2__Elusimicrobia;D_3__Lineage IIb;D_4__uncultured bacterium | D_0__Bacteria;D_1__Proteobacteria;D_2__Gammaproteobacteria;D_3__Methylococcales;D_4__Methylococcaceae |
| Minerotrophic | D_0__Archaea;D_1__Euryarchaeota;D_2__Methanomicrobia;D_3__Methanocellales;D_4__Methanocellaceae | D_0__Bacteria;D_1__Proteobacteria;D_2__Gammaproteobacteria;D_3__Pseudomonadales;D_4__Moraxellaceae |
| Minerotrophic | D_0__Archaea;D_1__Euryarchaeota;D_2__Methanomicrobia;D_3__Methanomicrobiales;D_4__Methanospirillaceae | D_0__Bacteria;D_1__Actinobacteria;D_2__Acidimicrobiia;D_3__Acidimicrobiales;__ |
| Minerotrophic | D_0__Bacteria;D_1__Proteobacteria;D_2__Gammaproteobacteria;D_3__Methylococcales;D_4__Methylococcaceae | D_0__Bacteria;D_1__Spirochaetae;D_2__Spirochaetes;D_3__Spirochaetales;D_4__Leptospiraceae |
| Minerotrophic | D_0__Bacteria;D_1__Proteobacteria;D_2__Gammaproteobacteria;D_3__Methylococcales;D_4__Methylococcaceae | D_0__Bacteria;D_1__Tectomicrobia;D_2__uncultured bacterium;D_3__uncultured bacterium;D_4__uncultured bacterium |
| Minerotrophic | D_0__Bacteria;D_1__Proteobacteria;D_2__Alphaproteobacteria;D_3__Rhizobiales;D_4__KF-JG30-B3 | D_0__Bacteria;D_1__Proteobacteria;D_2__Gammaproteobacteria;D_3__Methylococcales;D_4__Methylococcaceae |
| Minerotrophic | D_0__Archaea;D_1__Euryarchaeota;D_2__Methanomicrobia;D_3__Methanosarcinales;D_4__GOM Arc I | D_0__Bacteria;D_1__Nitrospirae;D_2__Nitrospira;D_3__Nitrospirales;D_4__0319-6A21 |
| Minerotrophic | D_0__Bacteria;D_1__FCPU426;__;__;__ | D_0__Bacteria;D_1__Proteobacteria;D_2__Gammaproteobacteria;D_3__Methylococcales;D_4__Methylococcaceae |
| Minerotrophic | D_0__Archaea;D_1__Euryarchaeota;D_2__Methanomicrobia;D_3__Methanosarcinales;__ | D_0__Archaea;D_1__Lokiarchaeota;Ambiguous_taxa;Ambiguous_taxa;Ambiguous_taxa |
| Minerotrophic | D_0__Archaea;D_1__Euryarchaeota;D_2__Methanobacteria;D_3__Methanobacteriales;D_4__Methanobacteriaceae | D_0__Bacteria;D_1__Planctomycetes;D_2__BD7-11;D_3__uncultured bacterium;D_4__uncultured bacterium |
| Minerotrophic | D_0__Archaea;D_1__Euryarchaeota;D_2__Methanomicrobia;D_3__Methanosarcinales;D_4__Methanosaetaceae | D_0__Bacteria;D_1__Planctomycetes;D_2__Phycisphaerae;__;__ |
| Minerotrophic | D_0__Archaea;D_1__Euryarchaeota;D_2__Methanobacteria;D_3__Methanobacteriales;D_4__Methanobacteriaceae | D_0__Bacteria;D_1__Proteobacteria;D_2__Deltaproteobacteria;D_3__Oligoflexales;D_4__0319-6G20 |
| Minerotrophic | D_0__Archaea;D_1__Euryarchaeota;D_2__Methanomicrobia;D_3__Methanosarcinales;D_4__GOM Arc I | D_0__Archaea;D_1__Thaumarchaeota;D_2__AK59;D_3__uncultured archaeon;D_4__uncultured archaeon |
| Minerotrophic | D_0__Archaea;D_1__Euryarchaeota;D_2__Methanobacteria;D_3__Methanobacteriales;D_4__Methanobacteriaceae | D_0__Bacteria;D_1__Planctomycetes;D_2__Phycisphaerae;__;__ |
| Minerotrophic | D_0__Bacteria;D_1__Chloroflexi;D_2__uncultured;__;__ | D_0__Bacteria;D_1__Proteobacteria;D_2__Alphaproteobacteria;D_3__Rhizobiales;D_4__Methylocystaceae |
| Minerotrophic | D_0__Archaea;D_1__Euryarchaeota;D_2__Methanomicrobia;D_3__Methanocellales;D_4__Methanocellaceae | D_0__Bacteria;D_1__Actinobacteria;D_2__OPB41;__;__ |
| Minerotrophic | D_0__Archaea;D_1__Euryarchaeota;D_2__Methanomicrobia;D_3__Methanosarcinales;D_4__Methanosarcinaceae | D_0__Bacteria;D_1__Actinobacteria;D_2__Acidimicrobiia;D_3__Acidimicrobiales;__ |
| Minerotrophic | D_0__Archaea;D_1__Euryarchaeota;D_2__Methanomicrobia;D_3__Methanomicrobiales;D_4__Methanomicrobiaceae | D_0__Bacteria;D_1__Nitrospinae;D_2__MD2898-B26;__;__ |
| Minerotrophic | D_0__Bacteria;D_1__Planctomycetes;D_2__Pla4 lineage;D_3__uncultured bacterium;D_4__uncultured bacterium | D_0__Bacteria;D_1__Proteobacteria;D_2__Gammaproteobacteria;D_3__Methylococcales;D_4__Methylococcaceae |
| Minerotrophic | D_0__Archaea;D_1__Euryarchaeota;D_2__Methanomicrobia;D_3__Methanomicrobiales;D_4__Methanospirillaceae | D_0__Bacteria;D_1__Actinobacteria;D_2__Actinobacteria;D_3__Frankiales;__ |
| Minerotrophic | D_0__Archaea;D_1__Euryarchaeota;D_2__Methanomicrobia;D_3__Methanosarcinales;D_4__Methanosaetaceae | D_0__Bacteria;D_1__WS2;__;__;__ |
| Minerotrophic | D_0__Archaea;D_1__Euryarchaeota;D_2__Methanomicrobia;D_3__Methanomicrobiales;__ | D_0__Bacteria;D_1__Proteobacteria;D_2__Betaproteobacteria;D_3__Methylophilales;D_4__Methylophilaceae |
| Minerotrophic | D_0__Archaea;D_1__Euryarchaeota;D_2__Methanomicrobia;D_3__Methanosarcinales;D_4__Methanosaetaceae | D_0__Bacteria;D_1__Chloroflexi;D_2__Dehalococcoidia;D_3__vadinBA26;D_4__uncultured bacterium |
| Minerotrophic | D_0__Bacteria;D_1__Armatimonadetes;D_2__Fimbriimonadia;D_3__Fimbriimonadales;__ | D_0__Bacteria;D_1__Proteobacteria;D_2__Gammaproteobacteria;D_3__Methylococcales;D_4__Methylococcaceae |
| Minerotrophic | D_0__Archaea;D_1__Euryarchaeota;D_2__Methanobacteria;D_3__Methanobacteriales;D_4__Methanobacteriaceae | D_0__Bacteria;D_1__Chloroflexi;D_2__Dehalococcoidia;D_3__MSBL5;D_4__uncultured bacterium |
| Minerotrophic | D_0__Bacteria;D_1__Microgenomates;D_2__Candidatus Curtissbacteria;__;__ | D_0__Bacteria;D_1__Proteobacteria;D_2__Gammaproteobacteria;D_3__Methylococcales;D_4__Methylococcaceae |
| Minerotrophic | D_0__Archaea;D_1__Euryarchaeota;D_2__Methanomicrobia;D_3__Methanosarcinales;__ | D_0__Bacteria;D_1__Planctomycetes;D_2__Phycisphaerae;D_3__Pla1 lineage;__ |
| Minerotrophic | D_0__Archaea;D_1__Euryarchaeota;D_2__Methanomicrobia;D_3__Methanosarcinales;__ | D_0__Bacteria;D_1__Bacteroidetes;D_2__Bacteroidia;D_3__Bacteroidales;D_4__Porphyromonadaceae |
| Minerotrophic | D_0__Archaea;D_1__Euryarchaeota;D_2__Methanomicrobia;D_3__Methanosarcinales;D_4__Methanosaetaceae | D_0__Archaea;D_1__Lokiarchaeota;D_2__uncultured archaeon;D_3__uncultured archaeon;D_4__uncultured archaeon |
| Minerotrophic | D_0__Archaea;D_1__Euryarchaeota;D_2__Methanomicrobia;D_3__Methanomicrobiales;D_4__Methanospirillaceae | D_0__Bacteria;D_1__Cyanobacteria;D_2__ML635J-21;__;__ |
| Minerotrophic | D_0__Archaea;D_1__Euryarchaeota;D_2__Methanomicrobia;D_3__Methanosarcinales;D_4__Methanosaetaceae | D_0__Bacteria;D_1__Spirochaetae;D_2__Spirochaetes;D_3__Spirochaetales;D_4__Spirochaetales Incertae Sedis |
| Minerotrophic | D_0__Archaea;D_1__Euryarchaeota;D_2__Methanomicrobia;D_3__Methanosarcinales;D_4__GOM Arc I | D_0__Bacteria;D_1__Candidatus Berkelbacteria;__;__;__ |
| Minerotrophic | D_0__Archaea;D_1__Euryarchaeota;D_2__Methanomicrobia;D_3__Methanosarcinales;D_4__Methanosaetaceae | D_0__Bacteria;D_1__RBG-1 (Zixibacteria);D_2__uncultured bacterium;D_3__uncultured bacterium;D_4__uncultured bacterium |
| Minerotrophic | D_0__Archaea;D_1__Euryarchaeota;D_2__Methanomicrobia;D_3__Methanosarcinales;D_4__Methanosaetaceae | D_0__Bacteria;D_1__Proteobacteria;D_2__Alphaproteobacteria;D_3__Rhodospirillales;D_4__Rhodospirillales Incertae Sedis |
| Minerotrophic | D_0__Archaea;D_1__Euryarchaeota;D_2__Methanomicrobia;D_3__Methanosarcinales;D_4__Methanosaetaceae | D_0__Bacteria;D_1__Chloroflexi;D_2__SJA-15;__;__ |
| Minerotrophic | D_0__Archaea;D_1__Euryarchaeota;D_2__Methanomicrobia;D_3__Methanomicrobiales;D_4__Methanomicrobiaceae | D_0__Bacteria;D_1__Proteobacteria;D_2__Gammaproteobacteria;D_3__Pseudomonadales;D_4__Moraxellaceae |
| Minerotrophic | D_0__Archaea;D_1__Euryarchaeota;D_2__Methanobacteria;D_3__Methanobacteriales;D_4__Methanobacteriaceae | D_0__Bacteria;D_1__Bacteroidetes;D_2__Sphingobacteriia;D_3__Sphingobacteriales;D_4__Lentimicrobiaceae |
| Minerotrophic | D_0__Archaea;D_1__Euryarchaeota;D_2__Methanobacteria;D_3__Methanobacteriales;D_4__Methanobacteriaceae | D_0__Bacteria;D_1__Firmicutes;D_2__Clostridia;D_3__Clostridiales;D_4__Christensenellaceae |
| Minerotrophic | D_0__Archaea;D_1__Euryarchaeota;D_2__Methanomicrobia;D_3__Methanosarcinales;__ | D_0__Bacteria;D_1__Proteobacteria;D_2__Deltaproteobacteria;D_3__Myxococcales;D_4__Phaselicystidaceae |
| Minerotrophic | D_0__Archaea;D_1__Euryarchaeota;D_2__Methanobacteria;D_3__Methanobacteriales;D_4__Methanobacteriaceae | D_0__Bacteria;D_1__Fibrobacteres;D_2__Fibrobacteria;D_3__Fibrobacterales;__ |
| Minerotrophic | D_0__Bacteria;D_1__Bacteroidetes;D_2__Sphingobacteriia;D_3__Sphingobacteriales;D_4__KD3-93 | D_0__Bacteria;D_1__Proteobacteria;D_2__Gammaproteobacteria;D_3__Methylococcales;D_4__Methylococcaceae |
| Minerotrophic | D_0__Archaea;D_1__Euryarchaeota;D_2__Methanomicrobia;D_3__Methanosarcinales;__ | D_0__Bacteria;D_1__Actinobacteria;D_2__Actinobacteria;D_3__Frankiales;__ |
| Minerotrophic | D_0__Archaea;D_1__Euryarchaeota;D_2__Methanobacteria;D_3__Methanobacteriales;D_4__Methanobacteriaceae | D_0__Bacteria;D_1__Chloroflexi;D_2__Dehalococcoidia;D_3__GIF9;D_4__uncultured bacterium |
| Minerotrophic | D_0__Archaea;D_1__Euryarchaeota;D_2__Methanobacteria;D_3__Methanobacteriales;D_4__Methanobacteriaceae | D_0__Bacteria;D_1__Proteobacteria;D_2__Alphaproteobacteria;D_3__Rhizobiales;__ |
| Minerotrophic | D_0__Bacteria;D_1__Planctomycetes;D_2__OM190;__;__ | D_0__Bacteria;D_1__Proteobacteria;D_2__Alphaproteobacteria;D_3__Rhizobiales;D_4__Methylocystaceae |
| Minerotrophic | D_0__Archaea;D_1__Euryarchaeota;D_2__Methanomicrobia;D_3__Methanomicrobiales;D_4__Methanospirillaceae | D_0__Bacteria;D_1__WS2;__;__;__ |
| Minerotrophic | D_0__Archaea;D_1__Euryarchaeota;D_2__Methanobacteria;D_3__Methanobacteriales;D_4__Methanobacteriaceae | D_0__Bacteria;D_1__Actinobacteria;D_2__Acidimicrobiia;D_3__Acidimicrobiales;__ |
| Minerotrophic | D_0__Archaea;D_1__Euryarchaeota;D_2__Methanomicrobia;D_3__Methanosarcinales;__ | D_0__Bacteria;D_1__Actinobacteria;D_2__Thermoleophilia;D_3__Solirubrobacterales;__ |
| Minerotrophic | D_0__Archaea;D_1__Euryarchaeota;D_2__Methanomicrobia;D_3__Methanosarcinales;D_4__Methanosaetaceae | D_0__Bacteria;D_1__Deinococcus-Thermus;D_2__Deinococci;D_3__KD3-62;D_4__uncultured bacterium |
| Minerotrophic | D_0__Archaea;D_1__Euryarchaeota;D_2__Methanomicrobia;D_3__Methanosarcinales;D_4__GOM Arc I | D_0__Bacteria;D_1__Proteobacteria;D_2__Deltaproteobacteria;D_3__SAR324 clade(Marine group B);D_4__uncultured bacterium |
| Minerotrophic | D_0__Bacteria;D_1__Bacteroidetes;D_2__Sphingobacteriia;D_3__Sphingobacteriales;D_4__uncultured | D_0__Bacteria;D_1__Proteobacteria;D_2__Gammaproteobacteria;D_3__Methylococcales;D_4__Methylococcaceae |
| Minerotrophic | D_0__Archaea;D_1__Euryarchaeota;D_2__Methanomicrobia;D_3__Methanomicrobiales;D_4__Methanomicrobiaceae | D_0__Bacteria;D_1__Actinobacteria;D_2__OPB41;__;__ |
| Minerotrophic | D_0__Archaea;D_1__Euryarchaeota;D_2__Methanomicrobia;D_3__Methanomicrobiales;D_4__Methanospirillaceae | D_0__Bacteria;D_1__Proteobacteria;D_2__Betaproteobacteria;D_3__Burkholderiales;D_4__Comamonadaceae |
| Minerotrophic | D_0__Archaea;D_1__Euryarchaeota;D_2__Methanomicrobia;D_3__Methanomicrobiales;D_4__Methanospirillaceae | D_0__Bacteria;D_1__Actinobacteria;D_2__Thermoleophilia;D_3__Solirubrobacterales;D_4__Patulibacteraceae |
| Minerotrophic | D_0__Archaea;D_1__Euryarchaeota;D_2__Methanomicrobia;D_3__Methanosarcinales;D_4__Methanosarcinaceae | D_0__Bacteria;D_1__Proteobacteria;D_2__Betaproteobacteria;D_3__Burkholderiales;D_4__Comamonadaceae |
| Minerotrophic | D_0__Archaea;D_1__Euryarchaeota;D_2__Methanomicrobia;D_3__Methanosarcinales;D_4__Methanosaetaceae | D_0__Bacteria;D_1__Chloroflexi;D_2__SJA-68;D_3__uncultured bacterium;D_4__uncultured bacterium |
| Minerotrophic | D_0__Archaea;D_1__Euryarchaeota;D_2__Methanomicrobia;D_3__Methanomicrobiales;D_4__Methanomicrobiaceae | D_0__Bacteria;D_1__Proteobacteria;D_2__Betaproteobacteria;D_3__Rhodocyclales;D_4__Rhodocyclaceae |
| Minerotrophic | D_0__Archaea;D_1__Euryarchaeota;D_2__Methanomicrobia;D_3__Methanosarcinales;D_4__Methanosaetaceae | D_0__Bacteria;D_1__LCP-89;D_2__uncultured bacterium;D_3__uncultured bacterium;D_4__uncultured bacterium |
| Minerotrophic | D_0__Bacteria;D_1__Bacteroidetes;D_2__Cytophagia;D_3__Cytophagales;D_4__Cytophagaceae | D_0__Bacteria;D_1__Proteobacteria;D_2__Gammaproteobacteria;D_3__Methylococcales;D_4__Methylococcaceae |
| Minerotrophic | D_0__Bacteria;D_1__Proteobacteria;D_2__Alphaproteobacteria;D_3__Rhizobiales;D_4__Bradyrhizobiaceae | D_0__Bacteria;D_1__Proteobacteria;D_2__Alphaproteobacteria;D_3__Rhizobiales;D_4__Methylocystaceae |
| Minerotrophic | D_0__Bacteria;D_1__Deinococcus-Thermus;D_2__Deinococci;D_3__KD3-62;__ | D_0__Bacteria;D_1__Proteobacteria;D_2__Gammaproteobacteria;D_3__Methylococcales;D_4__Methylococcaceae |
| Minerotrophic | D_0__Bacteria;D_1__Planctomycetes;D_2__Phycisphaerae;D_3__Phycisphaerales;__ | D_0__Bacteria;D_1__Proteobacteria;D_2__Alphaproteobacteria;D_3__Rhizobiales;D_4__Methylocystaceae |
| Minerotrophic | D_0__Archaea;D_1__Euryarchaeota;D_2__Methanomicrobia;D_3__Methanosarcinales;D_4__GOM Arc I | D_0__Bacteria;D_1__Proteobacteria;D_2__Alphaproteobacteria;D_3__Rhizobiales;D_4__KF-JG30-B3 |
| Minerotrophic | D_0__Archaea;D_1__Euryarchaeota;D_2__Methanobacteria;D_3__Methanobacteriales;D_4__Methanobacteriaceae | D_0__Bacteria;D_1__Actinobacteria;D_2__Actinobacteria;D_3__Frankiales;D_4__Sporichthyaceae |
| Minerotrophic | D_0__Archaea;D_1__Euryarchaeota;D_2__Methanomicrobia;D_3__Methanomicrobiales;D_4__Methanoregulaceae | D_0__Bacteria;D_1__WS2;D_2__uncultured bacterium;D_3__uncultured bacterium;D_4__uncultured bacterium |
| Minerotrophic | D_0__Archaea;D_1__Euryarchaeota;D_2__Methanomicrobia;D_3__Methanosarcinales;D_4__Methanosaetaceae | D_0__Bacteria;D_1__Cloacimonetes;__;__;__ |
| Minerotrophic | D_0__Archaea;D_1__Euryarchaeota;D_2__Methanomicrobia;D_3__Methanosarcinales;D_4__GOM Arc I | D_0__Archaea;D_1__Thaumarchaeota;D_2__Marine Benthic Group A;__;__ |
| Minerotrophic | D_0__Archaea;D_1__Euryarchaeota;D_2__Methanomicrobia;D_3__Methanosarcinales;D_4__Methanosarcinaceae | D_0__Bacteria;D_1__Proteobacteria;D_2__Deltaproteobacteria;D_3__Myxococcales;D_4__VHS-B3-70 |
| Minerotrophic | D_0__Archaea;D_1__Euryarchaeota;D_2__Methanomicrobia;D_3__Methanosarcinales;D_4__Methanosarcinaceae | D_0__Bacteria;D_1__Bacteroidetes;D_2__Bacteroidetes VC2.1 Bac22;D_3__uncultured bacterium;D_4__uncultured bacterium |
| Minerotrophic | D_0__Bacteria;D_1__Omnitrophica;D_2__Omnitrophica Incertae Sedis;D_3__Unknown Order;D_4__Unknown Family | D_0__Bacteria;D_1__Proteobacteria;D_2__Gammaproteobacteria;D_3__Methylococcales;D_4__Methylococcaceae |
| Minerotrophic | D_0__Archaea;D_1__Euryarchaeota;D_2__Methanomicrobia;D_3__Methanosarcinales;D_4__GOM Arc I | D_0__Bacteria;D_1__Proteobacteria;D_2__Betaproteobacteria;D_3__B1-7BS;D_4__uncultured bacterium |
| Minerotrophic | D_0__Archaea;D_1__Euryarchaeota;D_2__Methanomicrobia;D_3__Methanomicrobiales;D_4__Methanomicrobiaceae | D_0__Archaea;D_1__Euryarchaeota;D_2__Methanomicrobia;D_3__Methanomicrobiales;__ |
| Minerotrophic | D_0__Archaea;D_1__Euryarchaeota;D_2__Methanomicrobia;D_3__Methanomicrobiales;D_4__Methanospirillaceae | D_0__Bacteria;D_1__Chloroflexi;D_2__S085;__;__ |
| Minerotrophic | D_0__Archaea;D_1__Euryarchaeota;D_2__Methanobacteria;D_3__Methanobacteriales;D_4__Methanobacteriaceae | D_0__Bacteria;D_1__Proteobacteria;D_2__Deltaproteobacteria;D_3__Myxococcales;D_4__Archangiaceae |
| Mixed | D_0__Bacteria;D_1__Armatimonadetes;__;__;__ | D_0__Bacteria;D_1__Proteobacteria;D_2__Alphaproteobacteria;D_3__Rhizobiales;D_4__Methylocystaceae |
| Mixed | D_0__Archaea;D_1__Euryarchaeota;D_2__Methanobacteria;D_3__Methanobacteriales;D_4__Methanobacteriaceae | D_0__Bacteria;D_1__Acidobacteria;D_2__Holophagae;D_3__Subgroup 7;D_4__uncultured bacterium |
| Mixed | D_0__Archaea;D_1__Euryarchaeota;D_2__Methanomicrobia;D_3__Methanocellales;D_4__Methanocellaceae | D_0__Bacteria;D_1__Actinobacteria;D_2__Thermoleophilia;D_3__Gaiellales;D_4__Gaiellaceae |
| Mixed | D_0__Archaea;D_1__Euryarchaeota;D_2__Methanomicrobia;D_3__Methanosarcinales;D_4__Methanosaetaceae | D_0__Bacteria;D_1__Firmicutes;D_2__Clostridia;D_3__Clostridiales;D_4__Peptococcaceae |
| Mixed | D_0__Archaea;D_1__Euryarchaeota;D_2__Methanomicrobia;D_3__Methanosarcinales;D_4__Methanosarcinaceae | D_0__Bacteria;D_1__Proteobacteria;D_2__Deltaproteobacteria;D_3__Desulfurellales;D_4__Desulfurellaceae |
| Mixed | D_0__Archaea;D_1__Euryarchaeota;D_2__Methanobacteria;D_3__Methanobacteriales;D_4__Methanobacteriaceae | D_0__Bacteria;D_1__Proteobacteria;D_2__Alphaproteobacteria;D_3__Rhodospirillales;D_4__B79 |
| Mixed | D_0__Archaea;D_1__Euryarchaeota;D_2__Methanomicrobia;D_3__Methanomicrobiales;D_4__Methanoregulaceae | D_0__Bacteria;D_1__Firmicutes;D_2__Bacilli;D_3__Lactobacillales;D_4__Enterococcaceae |
| Mixed | D_0__Archaea;D_1__Euryarchaeota;D_2__Methanomicrobia;D_3__Methanosarcinales;D_4__Methanosaetaceae | D_0__Bacteria;D_1__Actinobacteria;D_2__Actinobacteria;D_3__Propionibacteriales;D_4__Propionibacteriaceae |
| Mixed | D_0__Archaea;D_1__Euryarchaeota;D_2__Methanomicrobia;__;__ | D_0__Archaea;D_1__Euryarchaeota;D_2__Thermoplasmata;D_3__Thermoplasmatales;D_4__Terrestrial Miscellaneous Gp(TMEG) |
| Mixed | D_0__Archaea;D_1__Euryarchaeota;D_2__Methanomicrobia;D_3__Methanocellales;D_4__Methanocellaceae | D_0__Bacteria;D_1__Bacteroidetes;D_2__Bacteroidia;D_3__Bacteroidales;D_4__Prolixibacteraceae |
| Mixed | D_0__Archaea;D_1__Euryarchaeota;D_2__Methanomicrobia;D_3__Methanocellales;D_4__Methanocellaceae | D_0__Bacteria;D_1__Bacteroidetes;D_2__Bacteroidetes vadinHA17;__;__ |
| Mixed | D_0__Archaea;D_1__Euryarchaeota;D_2__Methanomicrobia;D_3__Methanosarcinales;D_4__Methanosarcinaceae | D_0__Bacteria;D_1__Microgenomates;D_2__Candidatus Levybacteria;D_3__uncultured bacterium;D_4__uncultured bacterium |
| Mixed | D_0__Archaea;D_1__Euryarchaeota;D_2__Methanomicrobia;D_3__Methanosarcinales;D_4__Methanosarcinaceae | D_0__Bacteria;D_1__Proteobacteria;D_2__Betaproteobacteria;D_3__SC-I-84;__ |
| Mixed | D_0__Archaea;D_1__Euryarchaeota;D_2__Methanomicrobia;D_3__Methanomicrobiales;D_4__Methanomicrobiaceae | D_0__Bacteria;D_1__Proteobacteria;D_2__Gammaproteobacteria;D_3__Aeromonadales;D_4__Aeromonadaceae |
| Mixed | D_0__Archaea;D_1__Euryarchaeota;D_2__Methanomicrobia;D_3__Methanomicrobiales;D_4__Methanomicrobiaceae | D_0__Bacteria;D_1__Chloroflexi;D_2__Dehalococcoidia;D_3__vadinBA26;__ |
| Mixed | D_0__Archaea;D_1__Euryarchaeota;D_2__Methanobacteria;D_3__Methanobacteriales;D_4__Methanobacteriaceae | D_0__Bacteria;D_1__Chloroflexi;D_2__JG30-KF-CM66;D_3__uncultured bacterium;D_4__uncultured bacterium |
| Mixed | D_0__Bacteria;D_1__Proteobacteria;D_2__Alphaproteobacteria;D_3__Rhizobiales;D_4__Methylocystaceae | D_0__Bacteria;D_1__Proteobacteria;D_2__Deltaproteobacteria;D_3__Myxococcales;D_4__KD3-10 |
| Mixed | D_0__Archaea;D_1__Euryarchaeota;D_2__Methanomicrobia;D_3__Methanocellales;D_4__Methanocellaceae | D_0__Bacteria;D_1__Actinobacteria;D_2__Actinobacteria;D_3__Corynebacteriales;D_4__Mycobacteriaceae |
| Mixed | D_0__Archaea;D_1__Euryarchaeota;D_2__Methanomicrobia;D_3__Methanomicrobiales;D_4__Methanoregulaceae | D_0__Archaea;D_1__Parvarchaeota;D_2__uncultured archaeon;D_3__uncultured archaeon;D_4__uncultured archaeon |
| Mixed | D_0__Archaea;D_1__Euryarchaeota;D_2__Methanomicrobia;__;__ | D_0__Bacteria;D_1__Proteobacteria;D_2__Deltaproteobacteria;D_3__Deltaproteobacteria Incertae Sedis;D_4__Syntrophorhabdaceae |
| Mixed | D_0__Archaea;D_1__Euryarchaeota;D_2__Methanomicrobia;D_3__Methanomicrobiales;D_4__Methanomicrobiaceae | D_0__Bacteria;D_1__Bacteroidetes;D_2__Bacteroidetes vadinHA17;__;__ |
| Mixed | D_0__Archaea;D_1__Euryarchaeota;D_2__Methanobacteria;D_3__Methanobacteriales;D_4__Methanobacteriaceae | D_0__Bacteria;D_1__Proteobacteria;D_2__Gammaproteobacteria;D_3__Enterobacteriales;D_4__Enterobacteriaceae |
| Mixed | D_0__Bacteria;D_1__Proteobacteria;D_2__Alphaproteobacteria;D_3__Rhizobiales;D_4__Beijerinckiaceae | D_0__Bacteria;D_1__Proteobacteria;D_2__Alphaproteobacteria;D_3__Rhizobiales;D_4__Methylocystaceae |
| Mixed | D_0__Archaea;D_1__Euryarchaeota;D_2__Methanomicrobia;D_3__Methanocellales;D_4__Methanocellaceae | D_0__Bacteria;D_1__WS1;D_2__uncultured bacterium;D_3__uncultured bacterium;D_4__uncultured bacterium |
| Mixed | D_0__Archaea;D_1__Euryarchaeota;D_2__Methanobacteria;D_3__Methanobacteriales;D_4__Methanobacteriaceae | D_0__Bacteria;D_1__Actinobacteria;D_2__Actinobacteria;D_3__Streptomycetales;D_4__Streptomycetaceae |
| Mixed | D_0__Archaea;D_1__Euryarchaeota;D_2__Methanomicrobia;D_3__Methanosarcinales;D_4__Methanosarcinaceae | D_0__Bacteria;D_1__Proteobacteria;D_2__Alphaproteobacteria;D_3__Rickettsiales;D_4__uncultured |
| Mixed | D_0__Archaea;D_1__Euryarchaeota;D_2__Methanobacteria;D_3__Methanobacteriales;D_4__Methanobacteriaceae | D_0__Bacteria;D_1__Chloroflexi;D_2__Dehalococcoidia;__;__ |
| Mixed | D_0__Archaea;D_1__Euryarchaeota;D_2__Methanomicrobia;D_3__Methanomicrobiales;D_4__Methanoregulaceae | D_0__Bacteria;D_1__Proteobacteria;D_2__Alphaproteobacteria;D_3__Rhodospirillales;D_4__Acetobacteraceae |
| Mixed | D_0__Bacteria;D_1__Elusimicrobia;D_2__Elusimicrobia;D_3__Lineage IIb;D_4__uncultured bacterium | D_0__Bacteria;D_1__Proteobacteria;D_2__Alphaproteobacteria;D_3__Rhizobiales;D_4__Methylocystaceae |
| Mixed | D_0__Archaea;D_1__Euryarchaeota;D_2__Methanomicrobia;D_3__Methanosarcinales;__ | D_0__Bacteria;D_1__Bacteroidetes;__;__;__ |
| Mixed | D_0__Bacteria;D_1__Proteobacteria;D_2__Alphaproteobacteria;D_3__Rhizobiales;D_4__Methylocystaceae | D_0__Bacteria;D_1__Proteobacteria;D_2__Gammaproteobacteria;__;__ |
| Mixed | D_0__Archaea;D_1__Euryarchaeota;D_2__Methanobacteria;D_3__Methanobacteriales;D_4__Methanobacteriaceae | D_0__Bacteria;D_1__Proteobacteria;D_2__Deltaproteobacteria;D_3__Desulfobacterales;D_4__Desulfobacteraceae |
| Mixed | D_0__Archaea;D_1__Euryarchaeota;D_2__Methanomicrobia;D_3__Methanosarcinales;D_4__Methanosarcinaceae | D_0__Bacteria;D_1__Planctomycetes;D_2__Pla4 lineage;D_3__uncultured bacterium;D_4__uncultured bacterium |
| Mixed | D_0__Archaea;D_1__Euryarchaeota;D_2__Methanobacteria;D_3__Methanobacteriales;D_4__Methanobacteriaceae | D_0__Archaea;D_1__Euryarchaeota;D_2__Methanomicrobia;D_3__Methanocellales;D_4__Methanocellaceae |
| Mixed | D_0__Archaea;D_1__Euryarchaeota;D_2__Methanomicrobia;D_3__Methanomicrobiales;D_4__Methanomicrobiaceae | D_0__Archaea;D_1__Euryarchaeota;D_2__Methanomicrobia;__;__ |
| Mixed | D_0__Archaea;D_1__Euryarchaeota;D_2__Methanomicrobia;D_3__Methanomicrobiales;D_4__Methanoregulaceae | D_0__Bacteria;D_1__Verrucomicrobia;D_2__S-BQ2-57 soil group;D_3__uncultured bacterium;D_4__uncultured bacterium |
| Mixed | D_0__Archaea;D_1__Euryarchaeota;D_2__Methanobacteria;D_3__Methanobacteriales;D_4__Methanobacteriaceae | D_0__Bacteria;D_1__Planctomycetes;D_2__Phycisphaerae;D_3__MSBL9;D_4__uncultured bacterium |
| Mixed | D_0__Archaea;D_1__Euryarchaeota;D_2__Methanomicrobia;D_3__Methanosarcinales;__ | D_0__Bacteria;D_1__GAL15;D_2__uncultured bacterium;D_3__uncultured bacterium;D_4__uncultured bacterium |
| Mixed | D_0__Bacteria;D_1__Proteobacteria;D_2__Alphaproteobacteria;D_3__Rhizobiales;D_4__Methylocystaceae | D_0__Bacteria;D_1__Proteobacteria;D_2__Betaproteobacteria;D_3__Burkholderiales;D_4__Oxalobacteraceae |
| Mixed | D_0__Archaea;D_1__Bathyarchaeota;D_2__uncultured archaeon;D_3__uncultured archaeon;D_4__uncultured archaeon | D_0__Archaea;D_1__Euryarchaeota;D_2__Methanomicrobia;D_3__Methanomicrobiales;D_4__Methanomicrobiaceae |
| Mixed | D_0__Archaea;D_1__Euryarchaeota;D_2__Methanobacteria;D_3__Methanobacteriales;D_4__Methanobacteriaceae | D_0__Bacteria;D_1__Planctomycetes;D_2__Phycisphaerae;D_3__S-70;D_4__uncultured bacterium |
| Mixed | D_0__Archaea;D_1__Euryarchaeota;D_2__Methanomicrobia;D_3__Methanocellales;D_4__Methanocellaceae | D_0__Bacteria;D_1__Proteobacteria;D_2__Betaproteobacteria;D_3__B1-7BS;__ |
| Mixed | D_0__Archaea;D_1__Euryarchaeota;D_2__Methanomicrobia;D_3__Methanosarcinales;D_4__Methanosarcinaceae | D_0__Bacteria;D_1__Proteobacteria;__;__;__ |
| Mixed | D_0__Archaea;D_1__Euryarchaeota;D_2__Methanomicrobia;D_3__Methanosarcinales;D_4__Methanosarcinaceae | D_0__Bacteria;D_1__Deinococcus-Thermus;D_2__Deinococci;D_3__KD3-62;__ |
| Mixed | D_0__Archaea;D_1__Euryarchaeota;D_2__Methanomicrobia;D_3__Methanosarcinales;D_4__Methanosarcinaceae | D_0__Bacteria;D_1__Elusimicrobia;D_2__Elusimicrobia;D_3__Lineage IV;D_4__uncultured bacterium |
| Mixed | D_0__Bacteria;D_1__Elusimicrobia;D_2__Elusimicrobia;D_3__Lineage IV;D_4__uncultured bacterium | D_0__Bacteria;D_1__Proteobacteria;D_2__Alphaproteobacteria;D_3__Rhizobiales;D_4__Methylocystaceae |
| Mixed | D_0__Archaea;D_1__Miscellaneous Euryarchaeotic Group(MEG);D_2__uncultured archaeon;D_3__uncultured archaeon;D_4__uncultured archaeon | D_0__Bacteria;D_1__Proteobacteria;D_2__Alphaproteobacteria;D_3__Rhizobiales;D_4__Methylocystaceae |
| Mixed | D_0__Archaea;D_1__Euryarchaeota;D_2__Methanobacteria;D_3__Methanobacteriales;D_4__Methanobacteriaceae | D_0__Bacteria;D_1__Chloroflexi;D_2__Ktedonobacteria;D_3__C0119;D_4__uncultured bacterium |
| Mixed | D_0__Archaea;D_1__Euryarchaeota;D_2__Methanomicrobia;D_3__Methanosarcinales;D_4__Methanosarcinaceae | D_0__Bacteria;D_1__Proteobacteria;D_2__Deltaproteobacteria;D_3__Myxococcales;__ |
| Mixed | D_0__Archaea;D_1__Euryarchaeota;D_2__Methanomicrobia;D_3__Methanomicrobiales;D_4__Methanospirillaceae | D_0__Bacteria;D_1__Ignavibacteriae;D_2__Ignavibacteria;D_3__Ignavibacteriales;__ |
| Mixed | D_0__Archaea;D_1__Euryarchaeota;D_2__Methanobacteria;D_3__Methanobacteriales;D_4__Methanobacteriaceae | D_0__Bacteria;D_1__Proteobacteria;D_2__Gammaproteobacteria;D_3__Pseudomonadales;D_4__Pseudomonadaceae |
| Mixed | D_0__Bacteria;D_1__Atribacteria;__;__;__ | D_0__Bacteria;D_1__Proteobacteria;D_2__Alphaproteobacteria;D_3__Rhizobiales;D_4__Methylocystaceae |
| Mixed | D_0__Archaea;D_1__Euryarchaeota;D_2__Methanomicrobia;D_3__Methanosarcinales;__ | D_0__Bacteria;D_1__Spirochaetae;D_2__Spirochaetes;D_3__Spirochaetales;D_4__LH041 |
| Mixed | D_0__Archaea;D_1__Euryarchaeota;D_2__Methanomicrobia;D_3__Methanosarcinales;D_4__Methanosaetaceae | D_0__Bacteria;D_1__Acidobacteria;D_2__Holophagae;D_3__Holophagales;D_4__Holophagaceae |
| Mixed | D_0__Archaea;D_1__Euryarchaeota;D_2__Methanobacteria;D_3__Methanobacteriales;D_4__Methanobacteriaceae | D_0__Bacteria;D_1__Actinobacteria;D_2__Actinobacteria;D_3__Catenulisporales;D_4__Actinospicaceae |
| Mixed | D_0__Archaea;D_1__Euryarchaeota;D_2__Methanobacteria;D_3__Methanobacteriales;D_4__Methanobacteriaceae | D_0__Bacteria;D_1__Firmicutes;D_2__Clostridia;D_3__Clostridiales;D_4__Clostridiaceae 1 |
| Mixed | D_0__Archaea;D_1__Euryarchaeota;D_2__Methanomicrobia;D_3__Methanocellales;D_4__Methanocellaceae | D_0__Bacteria;D_1__Proteobacteria;D_2__Gammaproteobacteria;D_3__Aeromonadales;D_4__Aeromonadaceae |
| Mixed | D_0__Archaea;D_1__Euryarchaeota;D_2__Methanomicrobia;D_3__Methanosarcinales;D_4__Methanosarcinaceae | D_0__Bacteria;D_1__Acidobacteria;D_2__Subgroup 2;__;__ |
| Mixed | D_0__Archaea;D_1__Parvarchaeota;D_2__uncultured archaeon;D_3__uncultured archaeon;D_4__uncultured archaeon | D_0__Bacteria;D_1__Proteobacteria;D_2__Alphaproteobacteria;D_3__Rhizobiales;D_4__Methylocystaceae |
| Mixed | D_0__Archaea;D_1__Euryarchaeota;D_2__Methanomicrobia;D_3__Methanomicrobiales;D_4__Methanoregulaceae | D_0__Bacteria;D_1__Acidobacteria;D_2__Solibacteres;D_3__Solibacterales;D_4__Solibacteraceae (Subgroup 3) |
| Mixed | D_0__Bacteria;D_1__Planctomycetes;D_2__Phycisphaerae;D_3__Tepidisphaerales;D_4__Tepidisphaeraceae | D_0__Bacteria;D_1__Proteobacteria;D_2__Alphaproteobacteria;D_3__Rhizobiales;D_4__Methylocystaceae |
| Mixed | D_0__Archaea;D_1__Euryarchaeota;D_2__Methanomicrobia;D_3__Methanosarcinales;__ | D_0__Bacteria;D_1__Chloroflexi;D_2__Dehalococcoidia;D_3__vadinBA26;__ |
| Mixed | D_0__Archaea;D_1__Euryarchaeota;D_2__Methanomicrobia;D_3__Methanosarcinales;__ | D_0__Bacteria;D_1__Deinococcus-Thermus;D_2__Deinococci;D_3__KD3-62;__ |
| Mixed | D_0__Archaea;D_1__Euryarchaeota;D_2__Methanomicrobia;D_3__Methanomicrobiales;D_4__Methanospirillaceae | D_0__Bacteria;D_1__Bacteroidetes;D_2__SB-5;D_3__uncultured bacterium;D_4__uncultured bacterium |
| Mixed | D_0__Bacteria;D_1__Cyanobacteria;D_2__Melainabacteria;D_3__Obscuribacterales;D_4__uncultured bacterium | D_0__Bacteria;D_1__Proteobacteria;D_2__Alphaproteobacteria;D_3__Rhizobiales;D_4__Methylocystaceae |
| Mixed | D_0__Archaea;D_1__Euryarchaeota;D_2__Methanomicrobia;D_3__Methanomicrobiales;D_4__Methanoregulaceae | D_0__Bacteria;D_1__Acidobacteria;D_2__Subgroup 12;D_3__uncultured bacterium;D_4__uncultured bacterium |
| Mixed | D_0__Archaea;D_1__Euryarchaeota;D_2__Methanomicrobia;D_3__Methanocellales;D_4__Methanocellaceae | D_0__Bacteria;D_1__Chloroflexi;D_2__Ktedonobacteria;D_3__Ktedonobacterales;D_4__Ktedonobacteraceae |
| Mixed | D_0__Archaea;D_1__Euryarchaeota;D_2__Methanomicrobia;__;__ | D_0__Bacteria;D_1__Planctomycetes;D_2__Phycisphaerae;D_3__Phycisphaerales;D_4__ODP1230B30.02 sediment group |
| Mixed | D_0__Archaea;D_1__Euryarchaeota;D_2__Methanomicrobia;D_3__Methanosarcinales;__ | D_0__Bacteria;D_1__Verrucomicrobia;D_2__Spartobacteria;D_3__Chthoniobacterales;D_4__Xiphinematobacteraceae |
| Mixed | D_0__Archaea;D_1__Euryarchaeota;D_2__Methanobacteria;D_3__Methanobacteriales;D_4__Methanobacteriaceae | D_0__Bacteria;D_1__Bacteroidetes;D_2__SB-5;__;__ |
| Mixed | D_0__Archaea;D_1__Bathyarchaeota;D_2__uncultured archaeon;D_3__uncultured archaeon;D_4__uncultured archaeon | D_0__Archaea;D_1__Euryarchaeota;D_2__Methanomicrobia;D_3__Methanosarcinales;D_4__Methanosarcinaceae |
| Mixed | D_0__Archaea;D_1__Euryarchaeota;D_2__Methanomicrobia;D_3__Methanosarcinales;D_4__Methanosarcinaceae | D_0__Bacteria;D_1__Cyanobacteria;D_2__Melainabacteria;D_3__Vampirovibrionales;D_4__uncultured bacterium |
| Mixed | D_0__Archaea;D_1__Euryarchaeota;D_2__Methanomicrobia;D_3__Methanosarcinales;D_4__Methanosarcinaceae | D_0__Bacteria;D_1__Acidobacteria;D_2__Subgroup 17;__;__ |
| Mixed | D_0__Bacteria;D_1__Proteobacteria;D_2__Alphaproteobacteria;D_3__Rhizobiales;D_4__Methylocystaceae | D_0__Bacteria;D_1__Proteobacteria;D_2__Alphaproteobacteria;D_3__Rhizobiales;D_4__Xanthobacteraceae |
| Mixed | D_0__Archaea;D_1__Euryarchaeota;D_2__Methanomicrobia;D_3__Methanomicrobiales;D_4__Methanomicrobiaceae | D_0__Bacteria;D_1__Chloroflexi;D_2__SJA-15;D_3__uncultured bacterium;D_4__uncultured bacterium |
| Mixed | D_0__Archaea;D_1__Euryarchaeota;D_2__Methanomicrobia;D_3__Methanocellales;D_4__Methanocellaceae | D_0__Bacteria;D_1__Firmicutes;D_2__Clostridia;D_3__Thermoanaerobacterales;D_4__Thermoanaerobacteraceae |
| Mixed | D_0__Bacteria;D_1__FCPU426;D_2__uncultured bacterium;D_3__uncultured bacterium;D_4__uncultured bacterium | D_0__Bacteria;D_1__Proteobacteria;D_2__Alphaproteobacteria;D_3__Rhizobiales;D_4__Methylocystaceae |
| Mixed | D_0__Archaea;D_1__Bathyarchaeota;D_2__uncultured archaeon;D_3__uncultured archaeon;D_4__uncultured archaeon | D_0__Bacteria;D_1__Proteobacteria;D_2__Alphaproteobacteria;D_3__Rhizobiales;D_4__Methylocystaceae |
| Mixed | D_0__Archaea;D_1__Euryarchaeota;D_2__Methanomicrobia;D_3__Methanosarcinales;__ | D_0__Archaea;D_1__Thaumarchaeota;D_2__South African Gold Mine Gp 1(SAGMCG-1);D_3__uncultured archaeon;D_4__uncultured archaeon |
| Mixed | D_0__Archaea;D_1__Euryarchaeota;D_2__Methanomicrobia;D_3__Methanocellales;D_4__Methanocellaceae | D_0__Bacteria;D_1__Acidobacteria;D_2__Subgroup 22;D_3__uncultured bacterium;D_4__uncultured bacterium |
| Mixed | D_0__Archaea;D_1__Euryarchaeota;D_2__Methanomicrobia;D_3__Methanosarcinales;D_4__Methanosarcinaceae | D_0__Bacteria;D_1__Proteobacteria;D_2__Betaproteobacteria;D_3__Rhodocyclales;D_4__Rhodocyclaceae |
| Mixed | D_0__Archaea;D_1__Euryarchaeota;D_2__Methanomicrobia;D_3__Methanosarcinales;D_4__Methanosarcinaceae | D_0__Bacteria;D_1__Firmicutes;__;__;__ |
| Mixed | D_0__Archaea;D_1__Euryarchaeota;D_2__Methanomicrobia;D_3__Methanosarcinales;D_4__Methanosarcinaceae | D_0__Bacteria;D_1__Planctomycetes;D_2__BD7-11;D_3__uncultured bacterium;D_4__uncultured bacterium |
| Mixed | D_0__Archaea;D_1__Euryarchaeota;D_2__Methanomicrobia;D_3__Methanomicrobiales;D_4__Methanospirillaceae | D_0__Bacteria;D_1__Planctomycetes;D_2__OM190;__;__ |
| Mixed | D_0__Archaea;D_1__Euryarchaeota;D_2__Methanobacteria;D_3__Methanobacteriales;D_4__Methanobacteriaceae | D_0__Bacteria;D_1__Actinobacteria;D_2__Thermoleophilia;D_3__Solirubrobacterales;D_4__Gsoil-1167 |
| Mixed | D_0__Archaea;D_1__Euryarchaeota;D_2__Methanomicrobia;D_3__Methanosarcinales;D_4__Methanosarcinaceae | D_0__Bacteria;D_1__Armatimonadetes;__;__;__ |
| Mixed | D_0__Archaea;D_1__Euryarchaeota;D_2__Methanobacteria;D_3__Methanobacteriales;D_4__Methanobacteriaceae | D_0__Bacteria;D_1__Planctomycetes;D_2__Phycisphaerae;D_3__CPla-3 termite group;__ |
| Mixed | D_0__Archaea;D_1__Bathyarchaeota;D_2__uncultured archaeon;D_3__uncultured archaeon;D_4__uncultured archaeon | D_0__Archaea;D_1__Euryarchaeota;D_2__Methanomicrobia;D_3__Methanosarcinales;__ |
| Mixed | D_0__Bacteria;D_1__Proteobacteria;D_2__Alphaproteobacteria;D_3__Rhizobiales;D_4__Methylocystaceae | D_0__Bacteria;D_1__Proteobacteria;D_2__Alphaproteobacteria;D_3__Rhizobiales;D_4__Rhizobiales Incertae Sedis |
| Mixed | D_0__Archaea;D_1__Euryarchaeota;D_2__Methanomicrobia;D_3__Methanomicrobiales;D_4__Methanospirillaceae | D_0__Bacteria;D_1__Elusimicrobia;D_2__Elusimicrobia;D_3__Lineage I;D_4__Unknown Family |
| Mixed | D_0__Archaea;D_1__Euryarchaeota;D_2__Methanomicrobia;D_3__Methanosarcinales;D_4__Methanosarcinaceae | D_0__Bacteria;D_1__Proteobacteria;D_2__Alphaproteobacteria;D_3__Rhizobiales;D_4__Methylocystaceae |
| Mixed | D_0__Archaea;D_1__Euryarchaeota;D_2__Methanomicrobia;D_3__Methanosarcinales;D_4__Methanosarcinaceae | D_0__Bacteria;D_1__Proteobacteria;D_2__Deltaproteobacteria;D_3__Oligoflexales;D_4__Oligoflexaceae |
| Mixed | D_0__Archaea;D_1__Euryarchaeota;D_2__Methanomicrobia;D_3__Methanomicrobiales;D_4__Methanomicrobiaceae | D_0__Bacteria;D_1__Chloroflexi;D_2__Dehalococcoidia;D_3__vadinBA26;D_4__uncultured bacterium |
| Mixed | D_0__Bacteria;D_1__Acidobacteria;D_2__Acidobacteria;D_3__Acidobacteriales;D_4__Acidobacteriaceae (Subgroup 1) | D_0__Bacteria;D_1__Proteobacteria;D_2__Alphaproteobacteria;D_3__Rhizobiales;D_4__Methylocystaceae |
| Mixed | D_0__Archaea;D_1__Euryarchaeota;D_2__Methanomicrobia;D_3__Methanosarcinales;__ | D_0__Bacteria;D_1__Planctomycetes;__;__;__ |
| Mixed | D_0__Archaea;D_1__Euryarchaeota;D_2__Methanomicrobia;D_3__Methanocellales;D_4__Methanocellaceae | D_0__Bacteria;D_1__Proteobacteria;D_2__Deltaproteobacteria;D_3__Desulfobacterales;D_4__Desulfobacteraceae |
| Mixed | D_0__Archaea;D_1__Euryarchaeota;D_2__Methanomicrobia;D_3__Methanosarcinales;D_4__Methanosarcinaceae | D_0__Bacteria;D_1__Acidobacteria;D_2__Subgroup 25;D_3__uncultured bacterium;D_4__uncultured bacterium |
| Mixed | D_0__Archaea;D_1__Euryarchaeota;D_2__Methanobacteria;D_3__Methanobacteriales;D_4__Methanobacteriaceae | D_0__Bacteria;D_1__Actinobacteria;__;__;__ |
| Mixed | D_0__Archaea;D_1__Euryarchaeota;D_2__Methanobacteria;D_3__Methanobacteriales;D_4__Methanobacteriaceae | D_0__Bacteria;D_1__Firmicutes;D_2__Clostridia;D_3__Clostridiales;D_4__Lachnospiraceae |
| Mixed | D_0__Archaea;D_1__Euryarchaeota;D_2__Methanomicrobia;D_3__Methanomicrobiales;D_4__Methanospirillaceae | D_0__Bacteria;D_1__Firmicutes;D_2__Bacilli;D_3__Bacillales;D_4__Paenibacillaceae |
| Mixed | D_0__Archaea;D_1__Euryarchaeota;D_2__Methanomicrobia;D_3__Methanosarcinales;D_4__Methanosarcinaceae | D_0__Bacteria;D_1__RBG-1 (Zixibacteria);D_2__uncultured bacterium;D_3__uncultured bacterium;D_4__uncultured bacterium |
| Mixed | D_0__Archaea;D_1__Euryarchaeota;D_2__Methanomicrobia;D_3__Methanomicrobiales;D_4__Methanoregulaceae | D_0__Bacteria;D_1__Proteobacteria;D_2__Betaproteobacteria;D_3__Burkholderiales;D_4__Oxalobacteraceae |
| Mixed | D_0__Archaea;D_1__Euryarchaeota;D_2__Methanobacteria;D_3__Methanobacteriales;D_4__Methanobacteriaceae | D_0__Bacteria;D_1__Actinobacteria;D_2__Thermoleophilia;D_3__Solirubrobacterales;D_4__Elev-16S-1332 |
| Mixed | D_0__Archaea;D_1__Euryarchaeota;D_2__Methanomicrobia;D_3__Methanosarcinales;__ | D_0__Archaea;D_1__Lokiarchaeota;D_2__uncultured archaeon;D_3__uncultured archaeon;D_4__uncultured archaeon |
| Mixed | D_0__Archaea;D_1__Euryarchaeota;D_2__Methanomicrobia;__;__ | D_0__Bacteria;D_1__Acidobacteria;D_2__Subgroup 18;__;__ |
| Mixed | D_0__Archaea;D_1__Euryarchaeota;D_2__Methanobacteria;D_3__Methanobacteriales;D_4__Methanobacteriaceae | D_0__Bacteria;D_1__Actinobacteria;D_2__Acidimicrobiia;D_3__Acidimicrobiales;D_4__Acidimicrobiaceae |
| Mixed | D_0__Archaea;D_1__Euryarchaeota;D_2__Methanomicrobia;D_3__Methanosarcinales;D_4__Methanosarcinaceae | D_0__Bacteria;D_1__Verrucomicrobia;D_2__Opitutae;__;__ |
| Mixed | D_0__Archaea;D_1__Euryarchaeota;D_2__Methanobacteria;D_3__Methanobacteriales;D_4__Methanobacteriaceae | D_0__Bacteria;D_1__Actinobacteria;D_2__Thermoleophilia;D_3__Gaiellales;D_4__Gaiellaceae |
| Mixed | D_0__Archaea;D_1__Euryarchaeota;D_2__Methanomicrobia;D_3__Methanosarcinales;D_4__Methanosarcinaceae | D_0__Bacteria;D_1__Planctomycetes;D_2__Planctomycetacia;D_3__Planctomycetales;D_4__Planctomycetaceae |
| Mixed | D_0__Archaea;D_1__Euryarchaeota;D_2__Methanobacteria;D_3__Methanobacteriales;D_4__Methanobacteriaceae | D_0__Archaea;D_1__Thaumarchaeota;D_2__Soil Crenarchaeotic Group(SCG);__;__ |
| Mixed | D_0__Archaea;D_1__Euryarchaeota;D_2__Methanomicrobia;__;__ | D_0__Bacteria;D_1__Bacteroidetes;D_2__Bacteroidetes vadinHA17;D_3__uncultured bacterium;D_4__uncultured bacterium |
| Mixed | D_0__Archaea;D_1__Euryarchaeota;D_2__Methanomicrobia;D_3__Methanomicrobiales;D_4__Methanospirillaceae | D_0__Bacteria;D_1__Armatimonadetes;D_2__uncultured;D_3__uncultured bacterium;D_4__uncultured bacterium |
| Mixed | D_0__Archaea;D_1__Euryarchaeota;D_2__Methanomicrobia;D_3__Methanosarcinales;__ | D_0__Bacteria;D_1__Cyanobacteria;D_2__Melainabacteria;D_3__Gastranaerophilales;__ |
| Mixed | D_0__Archaea;D_1__Euryarchaeota;D_2__Methanomicrobia;D_3__Methanocellales;D_4__Methanocellaceae | D_0__Bacteria;D_1__Proteobacteria;D_2__Deltaproteobacteria;D_3__Desulfovibrionales;D_4__Desulfovibrionaceae |
| Mixed | D_0__Archaea;D_1__Euryarchaeota;D_2__Methanomicrobia;D_3__Methanomicrobiales;D_4__Methanoregulaceae | D_0__Bacteria;D_1__Cyanobacteria;D_2__Melainabacteria;D_3__Gastranaerophilales;__ |
| Mixed | D_0__Archaea;D_1__Euryarchaeota;D_2__Methanobacteria;D_3__Methanobacteriales;D_4__Methanobacteriaceae | D_0__Bacteria;D_1__Nitrospirae;D_2__Nitrospira;D_3__Nitrospirales;D_4__FW13 |
| Mixed | D_0__Archaea;D_1__Euryarchaeota;D_2__Methanomicrobia;D_3__Methanomicrobiales;D_4__Methanomicrobiaceae | D_0__Bacteria;D_1__Planctomycetes;D_2__Pla3 lineage;__;__ |
| Mixed | D_0__Archaea;D_1__Euryarchaeota;D_2__Methanomicrobia;D_3__Methanomicrobiales;D_4__Methanoregulaceae | D_0__Bacteria;D_1__Chloroflexi;D_2__Dehalococcoidia;D_3__vadinBA26;__ |
| Mixed | D_0__Bacteria;D_1__Proteobacteria;D_2__Alphaproteobacteria;D_3__Rhizobiales;D_4__Methylocystaceae | D_0__Bacteria;D_1__Proteobacteria;D_2__Alphaproteobacteria;D_3__Rhizobiales;__ |
| Mixed | D_0__Archaea;D_1__Euryarchaeota;D_2__Methanobacteria;D_3__Methanobacteriales;D_4__Methanobacteriaceae | D_0__Bacteria;D_1__Proteobacteria;D_2__Deltaproteobacteria;D_3__NB1-j;D_4__uncultured bacterium |
| Mixed | D_0__Archaea;D_1__Euryarchaeota;D_2__Methanomicrobia;D_3__Methanosarcinales;D_4__Methanosarcinaceae | D_0__Bacteria;D_1__Proteobacteria;D_2__Deltaproteobacteria;D_3__Desulfarculales;D_4__Desulfarculaceae |
| Mixed | D_0__Archaea;D_1__Euryarchaeota;D_2__Methanomicrobia;D_3__Methanomicrobiales;D_4__Methanospirillaceae | D_0__Bacteria;D_1__Nitrospirae;D_2__Nitrospira;D_3__Nitrospirales;D_4__FW13 |
| Mixed | D_0__Bacteria;D_1__Proteobacteria;D_2__Alphaproteobacteria;D_3__Rhizobiales;D_4__Methylocystaceae | D_0__Bacteria;D_1__Proteobacteria;D_2__Betaproteobacteria;D_3__Burkholderiales;D_4__Alcaligenaceae |
| Mixed | D_0__Bacteria;D_1__Proteobacteria;D_2__Alphaproteobacteria;D_3__Rhizobiales;D_4__Methylocystaceae | D_0__Bacteria;D_1__Proteobacteria;D_2__Deltaproteobacteria;D_3__Myxococcales;D_4__Archangiaceae |
| Mixed | D_0__Archaea;D_1__Euryarchaeota;D_2__Methanomicrobia;__;__ | D_0__Bacteria;D_1__Chloroflexi;D_2__Dehalococcoidia;D_3__vadinBA26;__ |
| Mixed | D_0__Archaea;D_1__Euryarchaeota;D_2__Methanomicrobia;__;__ | D_0__Bacteria;D_1__Acidobacteria;D_2__Subgroup 22;D_3__uncultured bacterium;D_4__uncultured bacterium |
| Mixed | D_0__Bacteria;D_1__Proteobacteria;D_2__Alphaproteobacteria;D_3__Rhizobiales;D_4__Methylocystaceae | D_0__Bacteria;D_1__Proteobacteria;D_2__Deltaproteobacteria;D_3__SAR324 clade(Marine group B);D_4__uncultured bacterium |
| Mixed | D_0__Bacteria;D_1__Proteobacteria;D_2__Alphaproteobacteria;D_3__Rhizobiales;D_4__Methylocystaceae | D_0__Bacteria;D_1__Proteobacteria;D_2__Betaproteobacteria;D_3__Nitrosomonadales;__ |
| Mixed | D_0__Archaea;D_1__Euryarchaeota;D_2__Methanomicrobia;D_3__Methanomicrobiales;D_4__Methanomicrobiaceae | D_0__Archaea;D_1__Lokiarchaeota;Ambiguous_taxa;Ambiguous_taxa;Ambiguous_taxa |
| Mixed | D_0__Archaea;D_1__Euryarchaeota;D_2__Methanomicrobia;D_3__Methanomicrobiales;D_4__Methanomicrobiaceae | D_0__Bacteria;D_1__Proteobacteria;D_2__Gammaproteobacteria;D_3__uncultured;__ |
| Mixed | D_0__Bacteria;D_1__Proteobacteria;D_2__Alphaproteobacteria;D_3__Rhizobiales;D_4__Methylocystaceae | D_0__Bacteria;D_1__Proteobacteria;D_2__Deltaproteobacteria;D_3__Myxococcales;__ |
| Mixed | D_0__Bacteria;D_1__Proteobacteria;D_2__Alphaproteobacteria;D_3__Rhizobiales;D_4__Methylocystaceae | D_0__Bacteria;D_1__Proteobacteria;D_2__Alphaproteobacteria;D_3__Rickettsiales;D_4__SM2D12 |
| Mixed | D_0__Bacteria;D_1__Elusimicrobia;D_2__Elusimicrobia;__;__ | D_0__Bacteria;D_1__Proteobacteria;D_2__Alphaproteobacteria;D_3__Rhizobiales;D_4__Methylocystaceae |
| Mixed | D_0__Archaea;D_1__Euryarchaeota;D_2__Methanobacteria;D_3__Methanobacteriales;D_4__Methanobacteriaceae | D_0__Bacteria;D_1__Actinobacteria;D_2__Actinobacteria;D_3__Frankiales;D_4__Acidothermaceae |
| Mixed | D_0__Archaea;D_1__Euryarchaeota;D_2__Methanomicrobia;D_3__Methanocellales;D_4__Methanocellaceae | D_0__Bacteria;D_1__Proteobacteria;D_2__Gammaproteobacteria;D_3__HOC36;D_4__uncultured bacterium |
| Mixed | D_0__Archaea;D_1__Euryarchaeota;D_2__Methanomicrobia;D_3__Methanomicrobiales;D_4__Methanospirillaceae | D_0__Bacteria;D_1__Proteobacteria;D_2__Betaproteobacteria;D_3__Neisseriales;D_4__Neisseriaceae |
| Mixed | D_0__Archaea;D_1__Euryarchaeota;D_2__Methanomicrobia;D_3__Methanosarcinales;D_4__Methanosarcinaceae | D_0__Bacteria;D_1__Atribacteria;__;__;__ |
| Mixed | D_0__Archaea;D_1__Euryarchaeota;D_2__Methanomicrobia;D_3__Methanomicrobiales;D_4__Methanoregulaceae | D_0__Bacteria;D_1__Proteobacteria;D_2__Deltaproteobacteria;D_3__Deltaproteobacteria Incertae Sedis;D_4__Syntrophorhabdaceae |
| Mixed | D_0__Archaea;D_1__Euryarchaeota;D_2__Methanomicrobia;D_3__Methanocellales;D_4__Methanocellaceae | D_0__Bacteria;D_1__Cyanobacteria;D_2__Melainabacteria;D_3__Gastranaerophilales;D_4__uncultured bacterium |
| Mixed | D_0__Archaea;D_1__Euryarchaeota;D_2__Methanomicrobia;D_3__Methanomicrobiales;D_4__Methanomicrobiaceae | D_0__Bacteria;D_1__Proteobacteria;D_2__Deltaproteobacteria;D_3__Deltaproteobacteria Incertae Sedis;D_4__Syntrophorhabdaceae |
| Mixed | D_0__Archaea;D_1__Bathyarchaeota;__;__;__ | D_0__Archaea;D_1__Euryarchaeota;D_2__Methanomicrobia;D_3__Methanomicrobiales;D_4__Methanospirillaceae |
| Mixed | D_0__Archaea;D_1__Euryarchaeota;D_2__Methanomicrobia;D_3__Methanosarcinales;D_4__Methanosarcinaceae | D_0__Bacteria;D_1__Spirochaetae;D_2__Spirochaetes;D_3__Spirochaetales;D_4__Leptospiraceae |
| Mixed | D_0__Archaea;D_1__Candidate division YNPFFA;__;__;__ | D_0__Archaea;D_1__Euryarchaeota;D_2__Methanomicrobia;D_3__Methanomicrobiales;D_4__Methanomicrobiaceae |
| Mixed | D_0__Archaea;D_1__Euryarchaeota;D_2__Methanomicrobia;D_3__Methanosarcinales;D_4__Methanosarcinaceae | D_0__Bacteria;D_1__Acidobacteria;D_2__Subgroup 2;Ambiguous_taxa;Ambiguous_taxa |
| Mixed | D_0__Archaea;D_1__Euryarchaeota;D_2__Methanomicrobia;D_3__Methanomicrobiales;D_4__Methanospirillaceae | D_0__Bacteria;D_1__Planctomycetes;D_2__Phycisphaerae;D_3__Phycisphaerales;D_4__AKAU3564 sediment group |
| Mixed | D_0__Archaea;D_1__Euryarchaeota;D_2__Methanomicrobia;D_3__Methanosarcinales;__ | D_0__Bacteria;D_1__Proteobacteria;D_2__Betaproteobacteria;D_3__Hydrogenophilales;D_4__Hydrogenophilaceae |
| Mixed | D_0__Archaea;D_1__Euryarchaeota;D_2__Methanobacteria;D_3__Methanobacteriales;D_4__Methanobacteriaceae | D_0__Bacteria;D_1__Proteobacteria;D_2__Gammaproteobacteria;D_3__Xanthomonadales;D_4__Xanthomonadaceae |
| Mixed | D_0__Archaea;D_1__Euryarchaeota;D_2__Methanobacteria;D_3__Methanobacteriales;D_4__Methanobacteriaceae | D_0__Bacteria;D_1__Proteobacteria;D_2__Deltaproteobacteria;D_3__Sva0485;D_4__uncultured bacterium |
| Mixed | D_0__Archaea;D_1__Euryarchaeota;D_2__Methanomicrobia;D_3__Methanomicrobiales;D_4__Methanospirillaceae | D_0__Bacteria;D_1__Chloroflexi;D_2__SBR2076;__;__ |
| Mixed | D_0__Archaea;D_1__Euryarchaeota;D_2__Methanomicrobia;D_3__Methanocellales;D_4__Methanocellaceae | D_0__Bacteria;D_1__Firmicutes;D_2__Clostridia;D_3__Clostridiales;D_4__Peptostreptococcaceae |
| Mixed | D_0__Archaea;D_1__Euryarchaeota;D_2__Methanobacteria;D_3__Methanobacteriales;D_4__Methanobacteriaceae | D_0__Bacteria;D_1__Chloroflexi;D_2__Ktedonobacteria;D_3__B12-WMSP1;D_4__uncultured bacterium |
| Mixed | D_0__Bacteria;D_1__Acidobacteria;D_2__Holophagae;D_3__Subgroup 7;__ | D_0__Bacteria;D_1__Proteobacteria;D_2__Alphaproteobacteria;D_3__Rhizobiales;D_4__Methylocystaceae |
| Mixed | D_0__Archaea;D_1__Euryarchaeota;D_2__Methanobacteria;D_3__Methanobacteriales;D_4__Methanobacteriaceae | D_0__Bacteria;D_1__Chloroflexi;D_2__Ktedonobacteria;D_3__JG30-KF-AS9;D_4__uncultured bacterium |
| Mixed | D_0__Archaea;D_1__Euryarchaeota;D_2__Methanomicrobia;D_3__Methanosarcinales;D_4__Methanosaetaceae | D_0__Archaea;D_1__Thaumarchaeota;__;__;__ |
| Mixed | D_0__Bacteria;D_1__Proteobacteria;D_2__Alphaproteobacteria;D_3__Rhizobiales;D_4__Methylocystaceae | D_0__Bacteria;D_1__Proteobacteria;D_2__Deltaproteobacteria;D_3__Myxococcales;D_4__P3OB-42 |
| Mixed | D_0__Bacteria;D_1__Acidobacteria;D_2__Subgroup 2;__;__ | D_0__Bacteria;D_1__Proteobacteria;D_2__Alphaproteobacteria;D_3__Rhizobiales;D_4__Methylocystaceae |
| Mixed | D_0__Archaea;D_1__Euryarchaeota;D_2__Methanobacteria;D_3__Methanobacteriales;D_4__Methanobacteriaceae | D_0__Bacteria;D_1__Chloroflexi;D_2__KD4-96;__;__ |
| Mixed | D_0__Archaea;D_1__Euryarchaeota;D_2__Methanomicrobia;D_3__Methanomicrobiales;D_4__Methanomicrobiaceae | D_0__Bacteria;D_1__Acidobacteria;D_2__Subgroup 22;D_3__uncultured bacterium;D_4__uncultured bacterium |
| Mixed | D_0__Bacteria;D_1__Proteobacteria;D_2__Alphaproteobacteria;D_3__Rhizobiales;D_4__Methylocystaceae | D_0__Bacteria;D_1__Proteobacteria;D_2__Gammaproteobacteria;D_3__Xanthomonadales;D_4__Xanthomonadales Incertae Sedis |
| Mixed | D_0__Bacteria;D_1__Proteobacteria;D_2__Alphaproteobacteria;D_3__Rhizobiales;D_4__Methylocystaceae | D_0__Bacteria;D_1__Proteobacteria;D_2__Deltaproteobacteria;D_3__Myxococcales;D_4__Polyangiaceae |
| Mixed | D_0__Archaea;D_1__Euryarchaeota;D_2__Methanomicrobia;D_3__Methanocellales;D_4__Methanocellaceae | D_0__Bacteria;D_1__Chloroflexi;D_2__Dehalococcoidia;D_3__vadinBA26;D_4__uncultured bacterium |
| Mixed | D_0__Archaea;D_1__Euryarchaeota;D_2__Methanomicrobia;D_3__Methanosarcinales;D_4__Methanosarcinaceae | D_0__Bacteria;D_1__Proteobacteria;D_2__Betaproteobacteria;D_3__Burkholderiales;__ |
| Mixed | D_0__Archaea;D_1__Euryarchaeota;D_2__Methanomicrobia;D_3__Methanomicrobiales;D_4__Methanomicrobiaceae | D_0__Archaea;__;__;__;__ |
| Mixed | D_0__Bacteria;D_1__Proteobacteria;D_2__Alphaproteobacteria;D_3__Rhizobiales;D_4__Methylocystaceae | D_0__Bacteria;D_1__Spirochaetae;D_2__Spirochaetes;D_3__Spirochaetales;D_4__LH041 |
| Mixed | D_0__Archaea;D_1__Euryarchaeota;D_2__Methanobacteria;D_3__Methanobacteriales;D_4__Methanobacteriaceae | D_0__Bacteria;D_1__Firmicutes;D_2__Clostridia;D_3__Clostridiales;D_4__Family XVIII |
| Mixed | D_0__Archaea;D_1__Euryarchaeota;D_2__Methanomicrobia;D_3__Methanomicrobiales;D_4__Methanospirillaceae | D_0__Bacteria;D_1__Planctomycetes;D_2__Phycisphaerae;D_3__Phycisphaerales;__ |
| Mixed | D_0__Archaea;D_1__Euryarchaeota;D_2__Methanomicrobia;D_3__Methanosarcinales;D_4__Methanosarcinaceae | D_0__Bacteria;D_1__Firmicutes;D_2__Clostridia;__;__ |
| Mixed | D_0__Bacteria;D_1__Firmicutes;D_2__Erysipelotrichia;D_3__Erysipelotrichales;D_4__Erysipelotrichaceae | D_0__Bacteria;D_1__Proteobacteria;D_2__Alphaproteobacteria;D_3__Rhizobiales;D_4__Methylocystaceae |
| Mixed | D_0__Archaea;D_1__Euryarchaeota;D_2__Methanobacteria;D_3__Methanobacteriales;D_4__Methanobacteriaceae | D_0__Bacteria;D_1__Proteobacteria;D_2__Deltaproteobacteria;D_3__Sva0485;__ |
| Mixed | D_0__Archaea;D_1__Euryarchaeota;D_2__Methanomicrobia;D_3__Methanocellales;D_4__Methanocellaceae | D_0__Bacteria;D_1__Acidobacteria;D_2__Subgroup 18;__;__ |
| Mixed | D_0__Archaea;D_1__Euryarchaeota;D_2__Methanomicrobia;D_3__Methanomicrobiales;D_4__Methanomicrobiaceae | D_0__Bacteria;D_1__Armatimonadetes;__;__;__ |
| Mixed | D_0__Archaea;D_1__Euryarchaeota;D_2__Methanomicrobia;__;__ | D_0__Bacteria;D_1__Proteobacteria;D_2__Deltaproteobacteria;D_3__Syntrophobacterales;D_4__Syntrophobacteraceae |
| Mixed | D_0__Bacteria;D_1__Proteobacteria;D_2__Alphaproteobacteria;D_3__Rhizobiales;D_4__Methylocystaceae | D_0__Bacteria;D_1__Proteobacteria;D_2__Deltaproteobacteria;D_3__Desulfuromonadales;D_4__Geobacteraceae |
| Mixed | D_0__Archaea;D_1__Euryarchaeota;D_2__Methanomicrobia;D_3__Methanosarcinales;D_4__Methanosaetaceae | D_0__Bacteria;D_1__Chloroflexi;D_2__TK10;__;__ |
| Mixed | D_0__Archaea;D_1__Euryarchaeota;D_2__Methanomicrobia;__;__ | D_0__Bacteria;D_1__Spirochaetae;D_2__Spirochaetes;D_3__Spirochaetales;D_4__Spirochaetaceae |
| Mixed | D_0__Archaea;D_1__Euryarchaeota;D_2__Methanomicrobia;D_3__Methanomicrobiales;D_4__Methanomicrobiaceae | D_0__Bacteria;D_1__Proteobacteria;D_2__Deltaproteobacteria;D_3__Sva0485;D_4__uncultured bacterium |
| Mixed | D_0__Archaea;D_1__Euryarchaeota;D_2__Methanomicrobia;D_3__Methanomicrobiales;D_4__Methanomicrobiaceae | D_0__Bacteria;D_1__Chloroflexi;D_2__Ktedonobacteria;D_3__Ktedonobacterales;D_4__JG30a-KF-32 |
| Mixed | D_0__Archaea;D_1__Euryarchaeota;D_2__Methanomicrobia;__;__ | D_0__Bacteria;D_1__Planctomycetes;D_2__Pla3 lineage;__;__ |
| Mixed | D_0__Archaea;D_1__Euryarchaeota;D_2__Methanomicrobia;D_3__Methanosarcinales;D_4__Methanosaetaceae | D_0__Bacteria;D_1__Bacteroidetes;D_2__Sphingobacteriia;D_3__Sphingobacteriales;D_4__Lentimicrobiaceae |
| Mixed | D_0__Archaea;D_1__Euryarchaeota;D_2__Methanomicrobia;D_3__Methanomicrobiales;D_4__Methanomicrobiaceae | D_0__Archaea;D_1__Euryarchaeota;D_2__Methanomicrobia;D_3__Methanosarcinales;__ |
| Mixed | D_0__Archaea;D_1__Euryarchaeota;D_2__Methanomicrobia;D_3__Methanosarcinales;D_4__Methanosarcinaceae | D_0__Bacteria;D_1__Verrucomicrobia;D_2__Spartobacteria;D_3__Chthoniobacterales;D_4__Xiphinematobacteraceae |
| Mixed | D_0__Archaea;D_1__Euryarchaeota;D_2__Methanomicrobia;D_3__Methanocellales;D_4__Methanocellaceae | D_0__Bacteria;D_1__Firmicutes;D_2__Negativicutes;D_3__Selenomonadales;D_4__Veillonellaceae |
| Mixed | D_0__Archaea;D_1__Euryarchaeota;D_2__Methanomicrobia;D_3__Methanomicrobiales;D_4__Methanoregulaceae | D_0__Bacteria;D_1__Verrucomicrobia;D_2__Spartobacteria;D_3__Chthoniobacterales;D_4__Xiphinematobacteraceae |
| Mixed | D_0__Bacteria;D_1__Planctomycetes;D_2__Pla4 lineage;D_3__uncultured bacterium;D_4__uncultured bacterium | D_0__Bacteria;D_1__Proteobacteria;D_2__Alphaproteobacteria;D_3__Rhizobiales;D_4__Methylocystaceae |
| Mixed | D_0__Archaea;D_1__Candidate division YNPFFA;__;__;__ | D_0__Archaea;D_1__Euryarchaeota;D_2__Methanomicrobia;D_3__Methanosarcinales;__ |
| Mixed | D_0__Archaea;D_1__Euryarchaeota;D_2__Methanomicrobia;__;__ | D_0__Bacteria;D_1__Proteobacteria;D_2__Gammaproteobacteria;D_3__Aeromonadales;D_4__Aeromonadaceae |
| Mixed | D_0__Archaea;D_1__Euryarchaeota;D_2__Methanomicrobia;D_3__Methanomicrobiales;D_4__Methanospirillaceae | D_0__Bacteria;D_1__Planctomycetes;D_2__Phycisphaerae;D_3__mle1-8;__ |
| Mixed | D_0__Archaea;D_1__Euryarchaeota;D_2__Methanomicrobia;D_3__Methanocellales;D_4__Methanocellaceae | D_0__Bacteria;D_1__Chloroflexi;D_2__Dehalococcoidia;D_3__GIF3;D_4__uncultured bacterium |
| Mixed | D_0__Archaea;D_1__Euryarchaeota;D_2__Methanomicrobia;D_3__Methanomicrobiales;D_4__Methanospirillaceae | D_0__Bacteria;D_1__Planctomycetes;D_2__Phycisphaerae;__;__ |
| Mixed | D_0__Bacteria;D_1__Acidobacteria;D_2__Subgroup 13;__;__ | D_0__Bacteria;D_1__Proteobacteria;D_2__Alphaproteobacteria;D_3__Rhizobiales;D_4__Methylocystaceae |
| Mixed | D_0__Archaea;D_1__Euryarchaeota;D_2__Methanomicrobia;D_3__Methanosarcinales;__ | D_0__Archaea;D_1__Woesearchaeota (DHVEG-6);__;__;__ |
| Mixed | D_0__Archaea;D_1__Euryarchaeota;D_2__Methanomicrobia;D_3__Methanosarcinales;__ | D_0__Bacteria;D_1__Proteobacteria;D_2__Deltaproteobacteria;D_3__Desulfuromonadales;D_4__Geobacteraceae |
| Mixed | D_0__Archaea;D_1__Euryarchaeota;D_2__Methanomicrobia;D_3__Methanosarcinales;D_4__Methanosarcinaceae | D_0__Archaea;D_1__Thaumarchaeota;D_2__Group C3;D_3__uncultured archaeon;D_4__uncultured archaeon |
| Mixed | D_0__Archaea;D_1__Euryarchaeota;D_2__Methanomicrobia;D_3__Methanosarcinales;D_4__Methanosarcinaceae | D_0__Bacteria;D_1__GAL15;D_2__uncultured bacterium;D_3__uncultured bacterium;D_4__uncultured bacterium |
| Mixed | D_0__Bacteria;D_1__Firmicutes;D_2__Clostridia;D_3__Halanaerobiales;D_4__ODP1230B8.23 | D_0__Bacteria;D_1__Proteobacteria;D_2__Alphaproteobacteria;D_3__Rhizobiales;D_4__Methylocystaceae |
| Mixed | D_0__Archaea;D_1__Euryarchaeota;D_2__Methanomicrobia;__;__ | D_0__Bacteria;D_1__Proteobacteria;D_2__Gammaproteobacteria;D_3__HOC36;__ |
| Mixed | D_0__Bacteria;D_1__Atribacteria;D_2__uncultured bacterium;D_3__uncultured bacterium;D_4__uncultured bacterium | D_0__Bacteria;D_1__Proteobacteria;D_2__Alphaproteobacteria;D_3__Rhizobiales;D_4__Methylocystaceae |
| Mixed | D_0__Archaea;D_1__Euryarchaeota;D_2__Methanomicrobia;D_3__Methanocellales;D_4__Methanocellaceae | D_0__Bacteria;D_1__Planctomycetes;D_2__OM190;D_3__uncultured bacterium;D_4__uncultured bacterium |
| Mixed | D_0__Archaea;D_1__Euryarchaeota;D_2__Methanomicrobia;D_3__Methanomicrobiales;D_4__Methanomicrobiaceae | D_0__Bacteria;D_1__Proteobacteria;D_2__Deltaproteobacteria;D_3__Bdellovibrionales;D_4__Bacteriovoracaceae |
| Mixed | D_0__Archaea;D_1__Euryarchaeota;D_2__Methanomicrobia;D_3__Methanosarcinales;D_4__Methanosarcinaceae | D_0__Bacteria;D_1__Acidobacteria;D_2__Acidobacteria;D_3__Acidobacteriales;D_4__Acidobacteriaceae (Subgroup 1) |
| Mixed | D_0__Bacteria;D_1__Firmicutes;D_2__Bacilli;D_3__Lactobacillales;D_4__Enterococcaceae | D_0__Bacteria;D_1__Proteobacteria;D_2__Alphaproteobacteria;D_3__Rhizobiales;D_4__Methylocystaceae |
| Mixed | D_0__Archaea;D_1__Euryarchaeota;D_2__Methanobacteria;D_3__Methanobacteriales;D_4__Methanobacteriaceae | D_0__Bacteria;D_1__Acidobacteria;D_2__Subgroup 18;__;__ |
| Mixed | D_0__Archaea;D_1__Euryarchaeota;D_2__Methanomicrobia;D_3__Methanosarcinales;__ | D_0__Bacteria;D_1__Proteobacteria;D_2__Deltaproteobacteria;D_3__Deltaproteobacteria Incertae Sedis;D_4__Syntrophorhabdaceae |
| Mixed | D_0__Archaea;D_1__Euryarchaeota;D_2__Methanomicrobia;D_3__Methanocellales;D_4__Methanocellaceae | D_0__Bacteria;D_1__Chloroflexi;D_2__Dehalococcoidia;__;__ |
| Mixed | D_0__Archaea;D_1__Euryarchaeota;D_2__Methanomicrobia;D_3__Methanosarcinales;D_4__Methanosarcinaceae | D_0__Bacteria;D_1__Firmicutes;D_2__Erysipelotrichia;D_3__Erysipelotrichales;D_4__Erysipelotrichaceae |
| Mixed | D_0__Archaea;D_1__Euryarchaeota;D_2__Methanomicrobia;D_3__Methanomicrobiales;D_4__Methanoregulaceae | D_0__Bacteria;D_1__Acidobacteria;D_2__Subgroup 13;D_3__uncultured bacterium;D_4__uncultured bacterium |
| Mixed | D_0__Bacteria;D_1__Acidobacteria;D_2__Subgroup 12;D_3__uncultured bacterium;D_4__uncultured bacterium | D_0__Bacteria;D_1__Proteobacteria;D_2__Alphaproteobacteria;D_3__Rhizobiales;D_4__Methylocystaceae |
| Mixed | D_0__Archaea;D_1__Euryarchaeota;D_2__Methanomicrobia;D_3__Methanosarcinales;__ | D_0__Bacteria;D_1__RBG-1 (Zixibacteria);D_2__uncultured bacterium;D_3__uncultured bacterium;D_4__uncultured bacterium |
| Mixed | D_0__Archaea;D_1__Euryarchaeota;D_2__Methanomicrobia;D_3__Methanomicrobiales;D_4__Methanospirillaceae | D_0__Bacteria;D_1__Planctomycetes;D_2__Phycisphaerae;D_3__MSBL9;__ |
| Mixed | D_0__Archaea;D_1__Euryarchaeota;D_2__Methanomicrobia;D_3__Methanosarcinales;__ | D_0__Bacteria;D_1__Chloroflexi;D_2__Dehalococcoidia;D_3__vadinBA26;D_4__uncultured bacterium |
| Mixed | D_0__Archaea;D_1__Euryarchaeota;D_2__Methanomicrobia;D_3__Methanosarcinales;D_4__Methanosarcinaceae | D_0__Bacteria;D_1__Acidobacteria;D_2__Holophagae;D_3__Subgroup 7;__ |
| Mixed | D_0__Bacteria;D_1__Acidobacteria;D_2__Subgroup 2;Ambiguous_taxa;Ambiguous_taxa | D_0__Bacteria;D_1__Proteobacteria;D_2__Alphaproteobacteria;D_3__Rhizobiales;D_4__Methylocystaceae |
| Mixed | D_0__Archaea;D_1__Euryarchaeota;D_2__Methanomicrobia;D_3__Methanomicrobiales;D_4__Methanospirillaceae | D_0__Bacteria;D_1__Chloroflexi;__;__;__ |
| Mixed | D_0__Archaea;D_1__Euryarchaeota;D_2__Methanobacteria;D_3__Methanobacteriales;D_4__Methanobacteriaceae | D_0__Bacteria;D_1__Firmicutes;D_2__Clostridia;D_3__Clostridiales;D_4__Ruminococcaceae |
| Mixed | D_0__Archaea;D_1__Euryarchaeota;D_2__Methanomicrobia;D_3__Methanomicrobiales;D_4__Methanospirillaceae | D_0__Bacteria;D_1__Fibrobacteres;D_2__Fibrobacteria;D_3__Fibrobacterales;D_4__B122 |
| Mixed | D_0__Archaea;D_1__Euryarchaeota;D_2__Methanomicrobia;D_3__Methanosarcinales;__ | D_0__Bacteria;D_1__Chloroflexi;D_2__Dehalococcoidia;D_3__GIF9;D_4__uncultured bacterium |
| Mixed | D_0__Archaea;D_1__Euryarchaeota;D_2__Methanomicrobia;D_3__Methanosarcinales;D_4__Methanosarcinaceae | D_0__Bacteria;D_1__Planctomycetes;D_2__Phycisphaerae;D_3__Tepidisphaerales;D_4__Tepidisphaeraceae |
| Mixed | D_0__Bacteria;D_1__Planctomycetes;D_2__Planctomycetacia;D_3__Planctomycetales;D_4__Planctomycetaceae | D_0__Bacteria;D_1__Proteobacteria;D_2__Alphaproteobacteria;D_3__Rhizobiales;D_4__Methylocystaceae |
| Mixed | D_0__Archaea;D_1__Euryarchaeota;D_2__Methanomicrobia;__;__ | D_0__Bacteria;D_1__Firmicutes;D_2__Clostridia;D_3__Clostridiales;D_4__Peptostreptococcaceae |
| Mixed | D_0__Archaea;D_1__Euryarchaeota;D_2__Methanomicrobia;D_3__Methanomicrobiales;D_4__Methanoregulaceae | D_0__Archaea;D_1__Miscellaneous Euryarchaeotic Group(MEG);D_2__uncultured archaeon;D_3__uncultured archaeon;D_4__uncultured archaeon |
| Mixed | D_0__Archaea;D_1__Euryarchaeota;D_2__Methanomicrobia;D_3__Methanomicrobiales;D_4__Methanomicrobiaceae | D_0__Bacteria;D_1__Chloroflexi;D_2__Dehalococcoidia;D_3__GIF9;D_4__uncultured bacterium |
| Mixed | D_0__Archaea;D_1__Euryarchaeota;D_2__Methanomicrobia;D_3__Methanosarcinales;D_4__Methanosarcinaceae | D_0__Bacteria;D_1__Proteobacteria;D_2__Alphaproteobacteria;D_3__Rhodospirillales;D_4__Rhodospirillales Incertae Sedis |
| Mixed | D_0__Archaea;D_1__Euryarchaeota;D_2__Methanomicrobia;D_3__Methanosarcinales;__ | D_0__Bacteria;D_1__Proteobacteria;D_2__Alphaproteobacteria;D_3__Rickettsiales;D_4__Holosporaceae |
| Mixed | D_0__Archaea;D_1__Euryarchaeota;D_2__Methanomicrobia;D_3__Methanosarcinales;D_4__Methanosarcinaceae | D_0__Bacteria;D_1__Cyanobacteria;D_2__Melainabacteria;D_3__Obscuribacterales;D_4__uncultured bacterium |
| Mixed | D_0__Archaea;D_1__Euryarchaeota;D_2__Methanomicrobia;__;__ | D_0__Bacteria;D_1__Chloroflexi;D_2__Dehalococcoidia;D_3__vadinBA26;D_4__uncultured bacterium |
| Mixed | D_0__Archaea;D_1__Euryarchaeota;D_2__Methanomicrobia;D_3__Methanosarcinales;D_4__Methanosarcinaceae | D_0__Bacteria;D_1__Proteobacteria;D_2__Gammaproteobacteria;__;__ |
| Mixed | D_0__Archaea;D_1__Euryarchaeota;D_2__Methanobacteria;D_3__Methanobacteriales;D_4__Methanobacteriaceae | D_0__Bacteria;D_1__Proteobacteria;D_2__Alphaproteobacteria;D_3__Rhizobiales;D_4__Phyllobacteriaceae |
| Mixed | D_0__Archaea;D_1__Euryarchaeota;D_2__Methanomicrobia;D_3__Methanosarcinales;__ | D_0__Bacteria;D_1__Acidobacteria;D_2__Subgroup 12;D_3__uncultured bacterium;D_4__uncultured bacterium |
| Mixed | D_0__Archaea;D_1__Euryarchaeota;D_2__Methanomicrobia;D_3__Methanomicrobiales;D_4__Methanospirillaceae | D_0__Bacteria;D_1__Ignavibacteriae;D_2__Ignavibacteria;D_3__Ignavibacteriales;D_4__BSV26 |
| Mixed | D_0__Archaea;D_1__Euryarchaeota;D_2__Methanomicrobia;D_3__Methanomicrobiales;D_4__Methanomicrobiaceae | D_0__Bacteria;D_1__Actinobacteria;D_2__Thermoleophilia;D_3__Gaiellales;D_4__Gaiellaceae |
| Mixed | D_0__Archaea;D_1__Euryarchaeota;D_2__Methanomicrobia;D_3__Methanomicrobiales;D_4__Methanospirillaceae | D_0__Bacteria;D_1__BRC1;D_2__uncultured bacterium;D_3__uncultured bacterium;D_4__uncultured bacterium |
| Mixed | D_0__Archaea;D_1__Euryarchaeota;D_2__Methanomicrobia;D_3__Methanocellales;D_4__Methanocellaceae | D_0__Bacteria;D_1__SBR1093;D_2__uncultured bacterium;D_3__uncultured bacterium;D_4__uncultured bacterium |
| Mixed | D_0__Bacteria;D_1__Proteobacteria;D_2__Alphaproteobacteria;D_3__Rhizobiales;D_4__Methylocystaceae | D_0__Bacteria;D_1__Proteobacteria;D_2__Gammaproteobacteria;D_3__Xanthomonadales;D_4__uncultured |
| Mixed | D_0__Archaea;D_1__Thaumarchaeota;D_2__Marine Benthic Group A;__;__ | D_0__Bacteria;D_1__Proteobacteria;D_2__Alphaproteobacteria;D_3__Rhizobiales;D_4__Methylocystaceae |
| Mixed | D_0__Bacteria;D_1__Proteobacteria;D_2__Alphaproteobacteria;D_3__Rhizobiales;D_4__Methylocystaceae | D_0__Bacteria;D_1__Proteobacteria;D_2__Betaproteobacteria;D_3__Rhodocyclales;D_4__Rhodocyclaceae |
| Mixed | D_0__Archaea;D_1__Euryarchaeota;D_2__Methanobacteria;D_3__Methanobacteriales;D_4__Methanobacteriaceae | D_0__Bacteria;D_1__SBR1093;D_2__uncultured bacterium;D_3__uncultured bacterium;D_4__uncultured bacterium |
| Mixed | D_0__Archaea;D_1__Euryarchaeota;D_2__Methanomicrobia;D_3__Methanosarcinales;D_4__Methanosarcinaceae | D_0__Archaea;D_1__Thaumarchaeota;D_2__Marine Benthic Group A;__;__ |
| Mixed | D_0__Archaea;D_1__Euryarchaeota;D_2__Methanomicrobia;D_3__Methanocellales;D_4__Methanocellaceae | D_0__Bacteria;D_1__Actinobacteria;D_2__Actinobacteria;D_3__Pseudonocardiales;D_4__Pseudonocardiaceae |
| Mixed | D_0__Archaea;D_1__Euryarchaeota;D_2__Methanomicrobia;D_3__Methanosarcinales;__ | D_0__Bacteria;D_1__Proteobacteria;D_2__Alphaproteobacteria;D_3__Rickettsiales;D_4__uncultured |
| Mixed | D_0__Archaea;D_1__Euryarchaeota;D_2__Methanobacteria;D_3__Methanobacteriales;D_4__Methanobacteriaceae | D_0__Bacteria;D_1__Bacteroidetes;D_2__Bacteroidia;D_3__Bacteroidales;D_4__Prolixibacteraceae |
| Mixed | D_0__Bacteria;D_1__Acidobacteria;D_2__Subgroup 15;D_3__uncultured bacterium;D_4__uncultured bacterium | D_0__Bacteria;D_1__Proteobacteria;D_2__Alphaproteobacteria;D_3__Rhizobiales;D_4__Methylocystaceae |
| Mixed | D_0__Archaea;D_1__Euryarchaeota;D_2__Methanomicrobia;D_3__Methanosarcinales;__ | D_0__Bacteria;D_1__Firmicutes;D_2__Bacilli;D_3__Lactobacillales;D_4__Lactobacillaceae |
| Mixed | D_0__Archaea;D_1__Euryarchaeota;D_2__Methanomicrobia;D_3__Methanosarcinales;D_4__Methanosaetaceae | D_0__Bacteria;D_1__Parcubacteria;D_2__uncultured bacterium;D_3__uncultured bacterium;D_4__uncultured bacterium |
| Mixed | D_0__Archaea;D_1__Euryarchaeota;D_2__Methanomicrobia;__;__ | D_0__Bacteria;D_1__Firmicutes;D_2__Bacilli;D_3__Lactobacillales;D_4__Lactobacillaceae |
| Mixed | D_0__Archaea;D_1__Euryarchaeota;D_2__Methanomicrobia;D_3__Methanosarcinales;D_4__Methanosarcinaceae | D_0__Archaea;D_1__Parvarchaeota;D_2__uncultured archaeon;D_3__uncultured archaeon;D_4__uncultured archaeon |
| Mixed | D_0__Archaea;D_1__Euryarchaeota;D_2__Methanomicrobia;D_3__Methanocellales;D_4__Methanocellaceae | D_0__Bacteria;D_1__Chloroflexi;D_2__Ktedonobacteria;D_3__Ktedonobacterales;D_4__JG30a-KF-32 |
| Mixed | D_0__Bacteria;D_1__Proteobacteria;D_2__Alphaproteobacteria;D_3__Rhizobiales;D_4__Methylocystaceae | D_0__Bacteria;D_1__Proteobacteria;D_2__Alphaproteobacteria;D_3__Rhodospirillales;__ |
| Mixed | D_0__Archaea;D_1__Euryarchaeota;D_2__Methanobacteria;D_3__Methanobacteriales;D_4__Methanobacteriaceae | D_0__Bacteria;D_1__Firmicutes;D_2__Negativicutes;D_3__Selenomonadales;D_4__Veillonellaceae |
| Mixed | D_0__Archaea;D_1__Euryarchaeota;D_2__Methanomicrobia;D_3__Methanomicrobiales;D_4__Methanospirillaceae | D_0__Bacteria;D_1__Deinococcus-Thermus;D_2__Deinococci;D_3__KD3-62;D_4__uncultured bacterium |
| Mixed | D_0__Archaea;D_1__Euryarchaeota;D_2__Methanomicrobia;D_3__Methanocellales;D_4__Methanocellaceae | D_0__Bacteria;D_1__Proteobacteria;D_2__Alphaproteobacteria;D_3__Rhizobiales;D_4__Rhodobiaceae |
| Mixed | D_0__Archaea;D_1__Euryarchaeota;D_2__Methanomicrobia;D_3__Methanomicrobiales;D_4__Methanoregulaceae | D_0__Bacteria;D_1__Proteobacteria;D_2__Deltaproteobacteria;D_3__Desulfuromonadales;D_4__Geobacteraceae |
| Mixed | D_0__Archaea;D_1__Euryarchaeota;D_2__Methanomicrobia;D_3__Methanomicrobiales;D_4__Methanomicrobiaceae | D_0__Bacteria;D_1__Proteobacteria;D_2__Deltaproteobacteria;D_3__Desulfovibrionales;D_4__Desulfovibrionaceae |
| Mixed | D_0__Archaea;D_1__Euryarchaeota;D_2__Methanobacteria;D_3__Methanobacteriales;D_4__Methanobacteriaceae | D_0__Bacteria;D_1__Firmicutes;D_2__Negativicutes;D_3__Selenomonadales;D_4__uncultured |
| Mixed | D_0__Archaea;D_1__Euryarchaeota;D_2__Methanomicrobia;D_3__Methanomicrobiales;D_4__Methanoregulaceae | D_0__Archaea;D_1__Euryarchaeota;D_2__Methanomicrobia;D_3__Methanosarcinales;__ |
| Mixed | D_0__Bacteria;D_1__Proteobacteria;D_2__Alphaproteobacteria;D_3__Rhizobiales;D_4__Methylocystaceae | D_0__Bacteria;D_1__Proteobacteria;D_2__Alphaproteobacteria;D_3__Rhizobiales;D_4__Roseiarcaceae |
| Mixed | D_0__Archaea;D_1__Euryarchaeota;D_2__Methanomicrobia;D_3__Methanosarcinales;__ | D_0__Bacteria;D_1__Microgenomates;D_2__Candidatus Levybacteria;D_3__uncultured bacterium;D_4__uncultured bacterium |
| Mixed | D_0__Archaea;D_1__Euryarchaeota;D_2__Methanomicrobia;D_3__Methanosarcinales;D_4__Methanosarcinaceae | D_0__Bacteria;D_1__Proteobacteria;D_2__Gammaproteobacteria;D_3__HTA4;D_4__uncultured bacterium |
| Mixed | D_0__Bacteria;D_1__Proteobacteria;D_2__Alphaproteobacteria;D_3__Rhizobiales;D_4__Methylocystaceae | D_0__Bacteria;D_1__Proteobacteria;D_2__Deltaproteobacteria;D_3__Myxococcales;D_4__27F-1492R |
| Mixed | D_0__Archaea;D_1__Euryarchaeota;D_2__Methanobacteria;D_3__Methanobacteriales;D_4__Methanobacteriaceae | D_0__Bacteria;D_1__Chloroflexi;D_2__Dehalococcoidia;D_3__GIF3;D_4__uncultured bacterium |
| Mixed | D_0__Bacteria;D_1__Acidobacteria;D_2__Blastocatellia;D_3__Blastocatellales;D_4__Blastocatellaceae (Subgroup 4) | D_0__Bacteria;D_1__Proteobacteria;D_2__Alphaproteobacteria;D_3__Rhizobiales;D_4__Methylocystaceae |
| Mixed | D_0__Archaea;D_1__Euryarchaeota;D_2__Methanomicrobia;D_3__Methanosarcinales;D_4__Methanosarcinaceae | D_0__Bacteria;D_1__Proteobacteria;D_2__Betaproteobacteria;D_3__Hydrogenophilales;D_4__Hydrogenophilaceae |
| Mixed | D_0__Archaea;D_1__Euryarchaeota;D_2__Methanomicrobia;D_3__Methanosarcinales;__ | D_0__Bacteria;D_1__Acidobacteria;D_2__Subgroup 15;D_3__uncultured bacterium;D_4__uncultured bacterium |
| Mixed | D_0__Bacteria;D_1__Proteobacteria;D_2__Alphaproteobacteria;D_3__Rhizobiales;D_4__Methylocystaceae | D_0__Bacteria;D_1__Proteobacteria;D_2__Betaproteobacteria;__;__ |
| Mixed | D_0__Archaea;D_1__Euryarchaeota;D_2__Methanomicrobia;D_3__Methanomicrobiales;D_4__Methanoregulaceae | D_0__Bacteria;D_1__Proteobacteria;D_2__Deltaproteobacteria;D_3__Myxococcales;D_4__Blfdi19 |
| Mixed | D_0__Archaea;D_1__Euryarchaeota;D_2__Methanomicrobia;D_3__Methanosarcinales;__ | D_0__Bacteria;D_1__Proteobacteria;D_2__Deltaproteobacteria;D_3__43F-1404R;__ |
| Mixed | D_0__Archaea;D_1__Euryarchaeota;D_2__Methanobacteria;D_3__Methanobacteriales;D_4__Methanobacteriaceae | D_0__Bacteria;D_1__Actinobacteria;D_2__Thermoleophilia;D_3__Solirubrobacterales;__ |
| Mixed | D_0__Archaea;D_1__Euryarchaeota;D_2__Methanomicrobia;D_3__Methanomicrobiales;D_4__Methanoregulaceae | D_0__Archaea;D_1__Thaumarchaeota;D_2__Marine Benthic Group A;__;__ |
| Mixed | D_0__Bacteria;D_1__Proteobacteria;D_2__Alphaproteobacteria;D_3__Rhizobiales;D_4__Methylocystaceae | D_0__Bacteria;D_1__Proteobacteria;D_2__Deltaproteobacteria;D_3__Desulfurellales;D_4__Desulfurellaceae |
| Mixed | D_0__Archaea;D_1__Euryarchaeota;D_2__Methanomicrobia;D_3__Methanocellales;D_4__Methanocellaceae | D_0__Bacteria;D_1__Bacteroidetes;D_2__Bacteroidetes vadinHA17;D_3__uncultured bacterium;D_4__uncultured bacterium |
| Mixed | D_0__Bacteria;D_1__Bacteroidetes;D_2__Cytophagia;D_3__Cytophagales;D_4__Flammeovirgaceae | D_0__Bacteria;D_1__Proteobacteria;D_2__Alphaproteobacteria;D_3__Rhizobiales;D_4__Methylocystaceae |
| Mixed | D_0__Archaea;D_1__Euryarchaeota;D_2__Methanobacteria;D_3__Methanobacteriales;D_4__Methanobacteriaceae | D_0__Bacteria;D_1__Chloroflexi;D_2__Thermomicrobia;D_3__JG30-KF-CM45;__ |
| Mixed | D_0__Bacteria;D_1__Elusimicrobia;D_2__Elusimicrobia;D_3__Lineage IV;__ | D_0__Bacteria;D_1__Proteobacteria;D_2__Alphaproteobacteria;D_3__Rhizobiales;D_4__Methylocystaceae |
| Mixed | D_0__Archaea;D_1__Euryarchaeota;D_2__Methanomicrobia;D_3__Methanosarcinales;D_4__Methanosarcinaceae | D_0__Bacteria;D_1__Proteobacteria;D_2__Betaproteobacteria;D_3__Burkholderiales;D_4__Alcaligenaceae |
| Mixed | D_0__Archaea;D_1__Euryarchaeota;D_2__Methanomicrobia;D_3__Methanosarcinales;__ | D_0__Bacteria;D_1__Bacteroidetes;D_2__Bacteroidia;D_3__Bacteroidales;__ |
| Mixed | D_0__Archaea;D_1__Euryarchaeota;D_2__Methanomicrobia;D_3__Methanosarcinales;D_4__Methanosarcinaceae | D_0__Bacteria;D_1__Proteobacteria;D_2__Deltaproteobacteria;D_3__43F-1404R;__ |
| Mixed | D_0__Archaea;D_1__Euryarchaeota;D_2__Methanomicrobia;D_3__Methanomicrobiales;D_4__Methanoregulaceae | D_0__Bacteria;D_1__Proteobacteria;D_2__Deltaproteobacteria;D_3__Myxococcales;D_4__Polyangiaceae |
| Mixed | D_0__Archaea;D_1__Aenigmarchaeota;D_2__Deep Sea Euryarchaeotic Group(DSEG);D_3__uncultured archaeon;D_4__uncultured archaeon | D_0__Archaea;D_1__Euryarchaeota;D_2__Methanomicrobia;D_3__Methanomicrobiales;D_4__Methanoregulaceae |
| Mixed | D_0__Bacteria;D_1__Bacteroidetes;D_2__Sphingobacteriia;D_3__Sphingobacteriales;D_4__uncultured | D_0__Bacteria;D_1__Proteobacteria;D_2__Alphaproteobacteria;D_3__Rhizobiales;D_4__Methylocystaceae |
| Mixed | D_0__Bacteria;D_1__Proteobacteria;D_2__Alphaproteobacteria;D_3__Rhizobiales;D_4__Methylocystaceae | D_0__Bacteria;D_1__Verrucomicrobia;D_2__Spartobacteria;D_3__Chthoniobacterales;D_4__Xiphinematobacteraceae |
| Mixed | D_0__Archaea;D_1__Euryarchaeota;D_2__Methanomicrobia;__;__ | D_0__Archaea;D_1__Lokiarchaeota;D_2__uncultured archaeon;D_3__uncultured archaeon;D_4__uncultured archaeon |
| Mixed | D_0__Archaea;D_1__Euryarchaeota;D_2__Methanomicrobia;D_3__Methanosarcinales;D_4__Methanosarcinaceae | D_0__Bacteria;D_1__Proteobacteria;D_2__Alphaproteobacteria;D_3__Rhodospirillales;D_4__MND8 |
| Mixed | D_0__Archaea;D_1__Euryarchaeota;D_2__Methanomicrobia;D_3__Methanomicrobiales;D_4__Methanoregulaceae | D_0__Archaea;D_1__Thaumarchaeota;D_2__FHMa11 terrestrial group;__;__ |
| Mixed | D_0__Archaea;D_1__Euryarchaeota;D_2__Methanobacteria;D_3__Methanobacteriales;D_4__Methanobacteriaceae | D_0__Bacteria;D_1__Actinobacteria;D_2__Thermoleophilia;D_3__Solirubrobacterales;D_4__YNPFFP1 |
| Mixed | D_0__Bacteria;D_1__Proteobacteria;D_2__Alphaproteobacteria;D_3__Rhizobiales;D_4__Methylocystaceae | D_0__Bacteria;D_1__Proteobacteria;D_2__Deltaproteobacteria;D_3__Oligoflexales;D_4__Oligoflexaceae |
| Mixed | D_0__Archaea;D_1__Euryarchaeota;D_2__Methanobacteria;D_3__Methanobacteriales;D_4__Methanobacteriaceae | D_0__Bacteria;D_1__Ignavibacteriae;D_2__Ignavibacteria;D_3__Ignavibacteriales;__ |
| Mixed | D_0__Archaea;D_1__Euryarchaeota;D_2__Methanomicrobia;D_3__Methanomicrobiales;D_4__Methanospirillaceae | D_0__Bacteria;D_1__Firmicutes;D_2__Bacilli;D_3__Bacillales;D_4__Planococcaceae |
| Mixed | D_0__Archaea;D_1__Euryarchaeota;D_2__Methanomicrobia;D_3__Methanocellales;D_4__Methanocellaceae | D_0__Bacteria;D_1__Chloroflexi;D_2__Thermomicrobia;D_3__JG30-KF-CM45;__ |
| Mixed | D_0__Archaea;D_1__Euryarchaeota;D_2__Methanomicrobia;D_3__Methanosarcinales;D_4__Methanosarcinaceae | D_0__Bacteria;D_1__Proteobacteria;D_2__Betaproteobacteria;__;__ |
| Mixed | D_0__Bacteria;D_1__Proteobacteria;D_2__Alphaproteobacteria;D_3__Rhizobiales;D_4__Methylocystaceae | D_0__Bacteria;D_1__Proteobacteria;D_2__Alphaproteobacteria;D_3__Rhodospirillales;D_4__Rhodospirillaceae |
| Mixed | D_0__Archaea;D_1__Euryarchaeota;D_2__Methanomicrobia;D_3__Methanomicrobiales;D_4__Methanomicrobiaceae | D_0__Bacteria;D_1__Proteobacteria;D_2__Deltaproteobacteria;D_3__Syntrophobacterales;D_4__Syntrophaceae |
| Mixed | D_0__Archaea;D_1__Euryarchaeota;D_2__Methanomicrobia;D_3__Methanosarcinales;D_4__Methanosarcinaceae | D_0__Bacteria;D_1__Proteobacteria;D_2__Deltaproteobacteria;D_3__Myxococcales;D_4__Polyangiaceae |
| Mixed | D_0__Archaea;D_1__Candidate division YNPFFA;__;__;__ | D_0__Archaea;D_1__Euryarchaeota;D_2__Methanomicrobia;__;__ |
| Mixed | D_0__Bacteria;D_1__Proteobacteria;D_2__Alphaproteobacteria;D_3__Rhizobiales;D_4__Methylocystaceae | D_0__Bacteria;D_1__Proteobacteria;D_2__Betaproteobacteria;D_3__SC-I-84;__ |
| Mixed | D_0__Archaea;D_1__Euryarchaeota;D_2__Methanomicrobia;D_3__Methanosarcinales;D_4__Methanosaetaceae | D_0__Bacteria;D_1__Verrucomicrobia;D_2__OPB35 soil group;D_3__uncultured bacterium;D_4__uncultured bacterium |
| Mixed | D_0__Archaea;D_1__Euryarchaeota;D_2__Methanomicrobia;D_3__Methanocellales;D_4__Methanocellaceae | D_0__Bacteria;D_1__Planctomycetes;D_2__Pla3 lineage;__;__ |
| Mixed | D_0__Archaea;D_1__Euryarchaeota;D_2__Methanomicrobia;D_3__Methanosarcinales;D_4__Methanosarcinaceae | D_0__Bacteria;D_1__Spirochaetae;D_2__Spirochaetes;D_3__Spirochaetales;D_4__LH041 |
| Mixed | D_0__Bacteria;D_1__Chlamydiae;D_2__Chlamydiae;D_3__Chlamydiales;D_4__cvE6 | D_0__Bacteria;D_1__Proteobacteria;D_2__Alphaproteobacteria;D_3__Rhizobiales;D_4__Methylocystaceae |
| Mixed | D_0__Archaea;D_1__Euryarchaeota;D_2__Methanomicrobia;D_3__Methanomicrobiales;D_4__Methanoregulaceae | D_0__Bacteria;D_1__Bacteroidetes;D_2__Bacteroidia;D_3__Bacteroidales;D_4__Porphyromonadaceae |
| Mixed | D_0__Archaea;D_1__Euryarchaeota;D_2__Methanomicrobia;D_3__Methanosarcinales;__ | D_0__Bacteria;D_1__Verrucomicrobia;D_2__Opitutae;__;__ |
| Mixed | D_0__Archaea;D_1__Euryarchaeota;D_2__Methanomicrobia;D_3__Methanomicrobiales;D_4__Methanospirillaceae | D_0__Bacteria;D_1__Chloroflexi;D_2__Ktedonobacteria;D_3__B12-WMSP1;__ |
| Mixed | D_0__Archaea;D_1__Euryarchaeota;D_2__Methanomicrobia;__;__ | D_0__Archaea;D_1__Woesearchaeota (DHVEG-6);__;__;__ |
| Mixed | D_0__Archaea;D_1__Euryarchaeota;D_2__Methanomicrobia;D_3__Methanomicrobiales;D_4__Methanomicrobiaceae | D_0__Archaea;D_1__Thaumarchaeota;D_2__Group C3;D_3__uncultured archaeon;D_4__uncultured archaeon |
| Mixed | D_0__Archaea;D_1__Euryarchaeota;D_2__Methanomicrobia;D_3__Methanosarcinales;D_4__Methanosarcinaceae | D_0__Bacteria;D_1__Proteobacteria;D_2__Gammaproteobacteria;D_3__Xanthomonadales;D_4__Xanthomonadales Incertae Sedis |
| Mixed | D_0__Archaea;D_1__Euryarchaeota;D_2__Methanomicrobia;D_3__Methanosarcinales;__ | D_0__Bacteria;D_1__Proteobacteria;D_2__Deltaproteobacteria;D_3__Bdellovibrionales;D_4__Bacteriovoracaceae |
| Mixed | D_0__Archaea;D_1__Euryarchaeota;D_2__Methanomicrobia;D_3__Methanosarcinales;D_4__Methanosaetaceae | D_0__Archaea;D_1__Thaumarchaeota;D_2__FHMa11 terrestrial group;D_3__uncultured archaeon;D_4__uncultured archaeon |
| Mixed | D_0__Bacteria;D_1__Firmicutes;D_2__Clostridia;D_3__Clostridiales;__ | D_0__Bacteria;D_1__Proteobacteria;D_2__Alphaproteobacteria;D_3__Rhizobiales;D_4__Methylocystaceae |
| Mixed | D_0__Archaea;D_1__Euryarchaeota;D_2__Methanomicrobia;D_3__Methanosarcinales;__ | D_0__Archaea;D_1__Parvarchaeota;D_2__uncultured archaeon;D_3__uncultured archaeon;D_4__uncultured archaeon |
| Mixed | D_0__Archaea;D_1__Euryarchaeota;D_2__Methanomicrobia;D_3__Methanomicrobiales;D_4__Methanomicrobiaceae | D_0__Bacteria;D_1__Chloroflexi;D_2__Ktedonobacteria;D_3__C0119;D_4__uncultured bacterium |
| Mixed | D_0__Archaea;D_1__Euryarchaeota;D_2__Methanomicrobia;D_3__Methanocellales;D_4__Methanocellaceae | D_0__Bacteria;D_1__Chloroflexi;D_2__Ktedonobacteria;D_3__Ktedonobacterales;__ |
| Mixed | D_0__Bacteria;D_1__Proteobacteria;D_2__Alphaproteobacteria;D_3__Rhizobiales;D_4__Methylocystaceae | D_0__Bacteria;D_1__Proteobacteria;D_2__Gammaproteobacteria;D_3__Legionellales;D_4__Coxiellaceae |
| Mixed | D_0__Archaea;D_1__Euryarchaeota;D_2__Methanomicrobia;D_3__Methanomicrobiales;D_4__Methanoregulaceae | D_0__Bacteria;D_1__Proteobacteria;D_2__Alphaproteobacteria;D_3__Rickettsiales;D_4__SM2D12 |
| Mixed | D_0__Bacteria;D_1__Proteobacteria;D_2__Alphaproteobacteria;D_3__Rhizobiales;D_4__Methylocystaceae | D_0__Bacteria;D_1__Proteobacteria;D_2__Alphaproteobacteria;D_3__Rickettsiales;D_4__Holosporaceae |
| Mixed | D_0__Archaea;D_1__Euryarchaeota;D_2__Methanomicrobia;D_3__Methanosarcinales;__ | D_0__Bacteria;D_1__Proteobacteria;D_2__Alphaproteobacteria;D_3__Rhodospirillales;D_4__MND8 |
| Mixed | D_0__Bacteria;D_1__Acidobacteria;D_2__Subgroup 5;D_3__uncultured bacterium;D_4__uncultured bacterium | D_0__Bacteria;D_1__Proteobacteria;D_2__Alphaproteobacteria;D_3__Rhizobiales;D_4__Methylocystaceae |
| Mixed | D_0__Archaea;D_1__Euryarchaeota;D_2__Methanomicrobia;D_3__Methanomicrobiales;D_4__Methanospirillaceae | D_0__Bacteria;D_1__Acidobacteria;D_2__Holophagae;D_3__Holophagae Incertae Sedis;D_4__Unknown Family |
| Mixed | D_0__Archaea;D_1__Euryarchaeota;D_2__Methanomicrobia;D_3__Methanosarcinales;D_4__Methanosarcinaceae | D_0__Bacteria;D_1__Proteobacteria;D_2__Alphaproteobacteria;D_3__Rhizobiales;D_4__Xanthobacteraceae |
| Mixed | D_0__Archaea;D_1__Thaumarchaeota;D_2__South African Gold Mine Gp 1(SAGMCG-1);D_3__uncultured archaeon;D_4__uncultured archaeon | D_0__Bacteria;D_1__Proteobacteria;D_2__Alphaproteobacteria;D_3__Rhizobiales;D_4__Methylocystaceae |
| Mixed | D_0__Archaea;D_1__Euryarchaeota;D_2__Methanomicrobia;D_3__Methanomicrobiales;D_4__Methanomicrobiaceae | D_0__Bacteria;D_1__Proteobacteria;D_2__Gammaproteobacteria;D_3__HOC36;D_4__uncultured bacterium |
| Mixed | D_0__Archaea;D_1__Euryarchaeota;D_2__Methanomicrobia;D_3__Methanocellales;D_4__Methanocellaceae | D_0__Bacteria;D_1__Chloroflexi;D_2__SJA-15;D_3__uncultured bacterium;D_4__uncultured bacterium |
| Mixed | D_0__Archaea;D_1__Euryarchaeota;D_2__Methanobacteria;D_3__Methanobacteriales;D_4__Methanobacteriaceae | D_0__Bacteria;D_1__Firmicutes;D_2__Bacilli;D_3__Bacillales;D_4__Bacillaceae |
| Mixed | D_0__Archaea;D_1__Euryarchaeota;D_2__Methanobacteria;D_3__Methanobacteriales;D_4__Methanobacteriaceae | D_0__Bacteria;D_1__Planctomycetes;D_2__OM190;D_3__uncultured bacterium;D_4__uncultured bacterium |
| Mixed | D_0__Archaea;D_1__Euryarchaeota;D_2__Methanomicrobia;D_3__Methanomicrobiales;D_4__Methanoregulaceae | D_0__Bacteria;D_1__Proteobacteria;D_2__Betaproteobacteria;D_3__Hydrogenophilales;D_4__Hydrogenophilaceae |
| Mixed | D_0__Archaea;D_1__Euryarchaeota;D_2__Methanomicrobia;__;__ | D_0__Bacteria;D_1__Proteobacteria;D_2__Deltaproteobacteria;D_3__Desulfovibrionales;D_4__Desulfovibrionaceae |
| Mixed | D_0__Archaea;D_1__Euryarchaeota;D_2__Methanobacteria;D_3__Methanobacteriales;D_4__Methanobacteriaceae | D_0__Bacteria;D_1__Proteobacteria;D_2__Alphaproteobacteria;D_3__Rhodospirillales;D_4__KCM-B-15 |
| Mixed | D_0__Archaea;D_1__Euryarchaeota;D_2__Methanobacteria;D_3__Methanobacteriales;D_4__Methanobacteriaceae | D_0__Archaea;D_1__Thaumarchaeota;__;__;__ |
| Mixed | D_0__Archaea;D_1__Euryarchaeota;D_2__Methanomicrobia;D_3__Methanosarcinales;D_4__Methanosarcinaceae | D_0__Bacteria;D_1__Chlamydiae;D_2__Chlamydiae;D_3__Chlamydiales;D_4__cvE6 |
| Mixed | D_0__Archaea;D_1__Euryarchaeota;D_2__Methanobacteria;D_3__Methanobacteriales;D_4__Methanobacteriaceae | D_0__Bacteria;D_1__Actinobacteria;D_2__Thermoleophilia;D_3__Gaiellales;D_4__uncultured |
| Mixed | D_0__Archaea;D_1__Euryarchaeota;D_2__Methanomicrobia;D_3__Methanomicrobiales;D_4__Methanospirillaceae | D_0__Bacteria;D_1__Acidobacteria;D_2__Subgroup 18;D_3__uncultured bacterium;D_4__uncultured bacterium |
| Mixed | D_0__Archaea;D_1__Euryarchaeota;D_2__Methanomicrobia;D_3__Methanocellales;D_4__Methanocellaceae | D_0__Bacteria;D_1__Planctomycetes;D_2__Phycisphaerae;D_3__MSBL9;__ |
| Mixed | D_0__Bacteria;D_1__Cyanobacteria;D_2__Melainabacteria;D_3__Vampirovibrionales;D_4__uncultured bacterium | D_0__Bacteria;D_1__Proteobacteria;D_2__Alphaproteobacteria;D_3__Rhizobiales;D_4__Methylocystaceae |
| Mixed | D_0__Archaea;D_1__Euryarchaeota;D_2__Methanomicrobia;D_3__Methanomicrobiales;D_4__Methanomicrobiaceae | D_0__Bacteria;D_1__Chloroflexi;D_2__SBR2076;D_3__uncultured bacterium;D_4__uncultured bacterium |
| Mixed | D_0__Archaea;D_1__Euryarchaeota;D_2__Methanobacteria;D_3__Methanobacteriales;D_4__Methanobacteriaceae | D_0__Bacteria;D_1__Firmicutes;D_2__Clostridia;D_3__Clostridiales;D_4__Peptostreptococcaceae |
| Mixed | D_0__Archaea;D_1__Euryarchaeota;D_2__Methanobacteria;D_3__Methanobacteriales;D_4__Methanobacteriaceae | D_0__Bacteria;D_1__Firmicutes;D_2__Clostridia;D_3__Clostridiales;D_4__Heliobacteriaceae |
| Mixed | D_0__Bacteria;D_1__Proteobacteria;D_2__Alphaproteobacteria;D_3__Rhizobiales;D_4__Methylocystaceae | D_0__Bacteria;D_1__Proteobacteria;D_2__Betaproteobacteria;D_3__Burkholderiales;__ |
| Mixed | D_0__Archaea;D_1__Euryarchaeota;D_2__Methanomicrobia;D_3__Methanosarcinales;D_4__Methanosarcinaceae | D_0__Bacteria;D_1__Proteobacteria;D_2__Alphaproteobacteria;D_3__Rhizobiales;D_4__Roseiarcaceae |
| Mixed | D_0__Archaea;D_1__Euryarchaeota;D_2__Methanomicrobia;D_3__Methanosarcinales;D_4__Methanosarcinaceae | D_0__Bacteria;D_1__Proteobacteria;D_2__Alphaproteobacteria;D_3__Rickettsiales;D_4__TK34 |
| Mixed | D_0__Archaea;D_1__Euryarchaeota;D_2__Methanomicrobia;D_3__Methanosarcinales;D_4__Methanosarcinaceae | D_0__Bacteria;D_1__Proteobacteria;D_2__Betaproteobacteria;D_3__Burkholderiales;D_4__Oxalobacteraceae |
| Mixed | D_0__Archaea;D_1__Euryarchaeota;D_2__Methanomicrobia;D_3__Methanomicrobiales;D_4__Methanoregulaceae | D_0__Bacteria;D_1__Acidobacteria;D_2__Subgroup 15;D_3__uncultured bacterium;D_4__uncultured bacterium |
| Mixed | D_0__Archaea;D_1__Euryarchaeota;D_2__Methanomicrobia;__;__ | D_0__Bacteria;D_1__Proteobacteria;D_2__Deltaproteobacteria;D_3__Syntrophobacterales;D_4__Syntrophaceae |
| Mixed | D_0__Archaea;D_1__Euryarchaeota;D_2__Methanomicrobia;D_3__Methanosarcinales;D_4__Methanosarcinaceae | D_0__Bacteria;D_1__Proteobacteria;D_2__Gammaproteobacteria;D_3__Xanthomonadales;D_4__uncultured |
| Mixed | D_0__Archaea;D_1__Euryarchaeota;D_2__Methanomicrobia;D_3__Methanosarcinales;D_4__Methanosarcinaceae | D_0__Bacteria;D_1__Proteobacteria;D_2__Deltaproteobacteria;D_3__Myxococcales;D_4__KD3-10 |
| Mixed | D_0__Bacteria;D_1__Proteobacteria;D_2__Alphaproteobacteria;D_3__Rhizobiales;D_4__Methylocystaceae | D_0__Bacteria;D_1__Proteobacteria;D_2__Alphaproteobacteria;D_3__Rhodospirillales;D_4__Acetobacteraceae |
| Mixed | D_0__Archaea;D_1__Euryarchaeota;D_2__Methanomicrobia;D_3__Methanosarcinales;D_4__Methanosarcinaceae | D_0__Bacteria;D_1__Elusimicrobia;D_2__Elusimicrobia;__;__ |
| Mixed | D_0__Archaea;D_1__Euryarchaeota;D_2__Methanomicrobia;D_3__Methanocellales;D_4__Methanocellaceae | D_0__Bacteria;D_1__Proteobacteria;D_2__Deltaproteobacteria;D_3__Syntrophobacterales;D_4__Syntrophaceae |
| Mixed | D_0__Archaea;D_1__Euryarchaeota;D_2__Methanomicrobia;__;__ | D_0__Bacteria;D_1__Proteobacteria;D_2__Deltaproteobacteria;D_3__Bdellovibrionales;D_4__Bacteriovoracaceae |
| Mixed | D_0__Archaea;D_1__Euryarchaeota;D_2__Methanomicrobia;__;__ | D_0__Bacteria;D_1__Chloroflexi;D_2__Dehalococcoidia;D_3__GIF9;D_4__uncultured bacterium |
| Mixed | D_0__Archaea;D_1__Euryarchaeota;D_2__Methanomicrobia;D_3__Methanosarcinales;__ | D_0__Bacteria;D_1__Verrucomicrobia;D_2__S-BQ2-57 soil group;D_3__uncultured bacterium;D_4__uncultured bacterium |
| Mixed | D_0__Bacteria;D_1__Armatimonadetes;D_2__Chthonomonadetes;D_3__Chthonomonadales;D_4__Chthonomonadaceae | D_0__Bacteria;D_1__Proteobacteria;D_2__Alphaproteobacteria;D_3__Rhizobiales;D_4__Methylocystaceae |
| Mixed | D_0__Archaea;D_1__Euryarchaeota;D_2__Methanomicrobia;D_3__Methanosarcinales;D_4__Methanosarcinaceae | D_0__Bacteria;D_1__Planctomycetes;__;__;__ |
| Mixed | D_0__Archaea;D_1__Euryarchaeota;D_2__Methanomicrobia;D_3__Methanosarcinales;__ | D_0__Bacteria;D_1__Spirochaetae;D_2__Spirochaetes;D_3__Spirochaetales;D_4__Spirochaetaceae |
| Mixed | D_0__Archaea;D_1__Euryarchaeota;D_2__Methanomicrobia;D_3__Methanomicrobiales;D_4__Methanospirillaceae | D_0__Bacteria;D_1__Chloroflexi;D_2__Ktedonobacteria;D_3__Ktedonobacterales;D_4__HSB OF53-F07 |
| Mixed | D_0__Archaea;D_1__Euryarchaeota;D_2__Methanomicrobia;D_3__Methanocellales;D_4__Methanocellaceae | D_0__Bacteria;D_1__Chloroflexi;D_2__KD4-96;__;__ |
| Mixed | D_0__Archaea;D_1__Euryarchaeota;D_2__Methanobacteria;D_3__Methanobacteriales;D_4__Methanobacteriaceae | D_0__Bacteria;D_1__Chloroflexi;D_2__SBR2076;__;__ |
| Mixed | D_0__Archaea;D_1__Euryarchaeota;D_2__Methanomicrobia;D_3__Methanomicrobiales;D_4__Methanospirillaceae | D_0__Bacteria;D_1__Chloroflexi;D_2__JG37-AG-4;D_3__uncultured bacterium;D_4__uncultured bacterium |
| Mixed | D_0__Archaea;D_1__Euryarchaeota;D_2__Methanomicrobia;D_3__Methanocellales;D_4__Methanocellaceae | D_0__Bacteria;D_1__Firmicutes;D_2__Bacilli;D_3__Bacillales;D_4__Bacillaceae |
| Mixed | D_0__Archaea;D_1__Euryarchaeota;D_2__Methanomicrobia;__;__ | D_0__Bacteria;D_1__Proteobacteria;D_2__Gammaproteobacteria;D_3__HOC36;D_4__uncultured bacterium |
| Mixed | D_0__Bacteria;D_1__Planctomycetes;D_2__BD7-11;D_3__uncultured bacterium;D_4__uncultured bacterium | D_0__Bacteria;D_1__Proteobacteria;D_2__Alphaproteobacteria;D_3__Rhizobiales;D_4__Methylocystaceae |
| Mixed | D_0__Archaea;D_1__Euryarchaeota;D_2__Methanobacteria;D_3__Methanobacteriales;D_4__Methanobacteriaceae | D_0__Bacteria;D_1__Chloroflexi;D_2__TK10;D_3__uncultured bacterium;D_4__uncultured bacterium |
| Mixed | D_0__Archaea;D_1__Euryarchaeota;D_2__Methanomicrobia;D_3__Methanomicrobiales;D_4__Methanospirillaceae | D_0__Bacteria;D_1__Proteobacteria;D_2__Betaproteobacteria;D_3__Nitrosomonadales;D_4__Gallionellaceae |
| Mixed | D_0__Archaea;D_1__Euryarchaeota;D_2__Methanomicrobia;D_3__Methanomicrobiales;D_4__Methanomicrobiaceae | D_0__Archaea;D_1__Woesearchaeota (DHVEG-6);__;__;__ |
| Mixed | D_0__Archaea;D_1__Euryarchaeota;D_2__Methanomicrobia;D_3__Methanomicrobiales;D_4__Methanospirillaceae | D_0__Bacteria;D_1__Planctomycetes;D_2__Pla4 lineage;__;__ |
| Mixed | D_0__Bacteria;D_1__Proteobacteria;D_2__Alphaproteobacteria;D_3__Rhizobiales;D_4__Methylocystaceae | D_0__Bacteria;D_1__Proteobacteria;D_2__Alphaproteobacteria;D_3__Rhodospirillales;D_4__Rhodospirillales Incertae Sedis |
| Mixed | D_0__Archaea;D_1__Euryarchaeota;D_2__Methanomicrobia;D_3__Methanosarcinales;__ | D_0__Archaea;D_1__Euryarchaeota;D_2__Methanomicrobia;__;__ |
| Mixed | D_0__Archaea;D_1__Euryarchaeota;D_2__Methanobacteria;D_3__Methanobacteriales;D_4__Methanobacteriaceae | D_0__Bacteria;D_1__Chloroflexi;D_2__Ktedonobacteria;D_3__Ktedonobacterales;D_4__JG30a-KF-32 |
| Mixed | D_0__Archaea;D_1__Euryarchaeota;D_2__Methanomicrobia;D_3__Methanomicrobiales;D_4__Methanospirillaceae | D_0__Bacteria;D_1__Verrucomicrobia;D_2__OPB35 soil group;__;__ |
| Mixed | D_0__Archaea;D_1__Thaumarchaeota;D_2__FHMa11 terrestrial group;__;__ | D_0__Bacteria;D_1__Proteobacteria;D_2__Alphaproteobacteria;D_3__Rhizobiales;D_4__Methylocystaceae |
| Mixed | D_0__Archaea;D_1__Euryarchaeota;D_2__Methanomicrobia;D_3__Methanomicrobiales;D_4__Methanospirillaceae | D_0__Bacteria;D_1__Planctomycetes;D_2__Phycisphaerae;D_3__Phycisphaerales;D_4__08D2Z94 hypersaline microbial mat group |
| Mixed | D_0__Archaea;D_1__Euryarchaeota;D_2__Methanomicrobia;D_3__Methanomicrobiales;D_4__Methanomicrobiaceae | D_0__Bacteria;D_1__Firmicutes;D_2__Bacilli;D_3__Lactobacillales;D_4__Lactobacillaceae |
| Mixed | D_0__Archaea;D_1__Euryarchaeota;D_2__Methanomicrobia;D_3__Methanosarcinales;D_4__Methanosarcinaceae | D_0__Archaea;D_1__Thaumarchaeota;D_2__South African Gold Mine Gp 1(SAGMCG-1);D_3__uncultured archaeon;D_4__uncultured archaeon |
| Mixed | D_0__Archaea;D_1__Euryarchaeota;D_2__Methanobacteria;D_3__Methanobacteriales;D_4__Methanobacteriaceae | D_0__Bacteria;D_1__Proteobacteria;D_2__Betaproteobacteria;D_3__B1-7BS;__ |
| Mixed | D_0__Archaea;D_1__Euryarchaeota;D_2__Methanomicrobia;D_3__Methanosarcinales;D_4__Methanosarcinaceae | D_0__Bacteria;D_1__Acidobacteria;D_2__Blastocatellia;D_3__Blastocatellales;D_4__Blastocatellaceae (Subgroup 4) |
| Mixed | D_0__Bacteria;D_1__Proteobacteria;D_2__Alphaproteobacteria;D_3__Rhizobiales;D_4__Methylocystaceae | D_0__Bacteria;D_1__Proteobacteria;__;__;__ |
| Mixed | D_0__Archaea;D_1__Euryarchaeota;D_2__Methanomicrobia;D_3__Methanomicrobiales;D_4__Methanoregulaceae | D_0__Bacteria;D_1__Verrucomicrobia;D_2__OPB35 soil group;D_3__uncultured bacterium;D_4__uncultured bacterium |
| Mixed | D_0__Archaea;D_1__Euryarchaeota;D_2__Methanomicrobia;D_3__Methanosarcinales;D_4__Methanosarcinaceae | D_0__Bacteria;D_1__Proteobacteria;D_2__Deltaproteobacteria;D_3__Myxococcales;D_4__P3OB-42 |
| Mixed | D_0__Archaea;D_1__Euryarchaeota;D_2__Methanomicrobia;D_3__Methanocellales;D_4__Methanocellaceae | D_0__Bacteria;D_1__Chloroflexi;D_2__Dehalococcoidia;D_3__Dehalococcoidales;D_4__uncultured |
| Mixed | D_0__Archaea;D_1__Euryarchaeota;D_2__Methanomicrobia;D_3__Methanosarcinales;D_4__Methanosarcinaceae | D_0__Bacteria;D_1__Proteobacteria;D_2__Deltaproteobacteria;D_3__Myxococcales;D_4__Archangiaceae |
| Mixed | D_0__Archaea;D_1__Euryarchaeota;D_2__Methanomicrobia;D_3__Methanosarcinales;D_4__Methanosarcinaceae | D_0__Bacteria;D_1__Proteobacteria;D_2__Deltaproteobacteria;D_3__SAR324 clade(Marine group B);D_4__uncultured bacterium |
| Mixed | D_0__Archaea;D_1__Euryarchaeota;D_2__Methanomicrobia;D_3__Methanomicrobiales;D_4__Methanospirillaceae | D_0__Bacteria;D_1__Nitrospirae;D_2__Nitrospira;D_3__Nitrospirales;D_4__Nitrospiraceae |
| Mixed | D_0__Archaea;D_1__Euryarchaeota;D_2__Methanomicrobia;D_3__Methanosarcinales;D_4__Methanosarcinaceae | D_0__Bacteria;D_1__Proteobacteria;D_2__Alphaproteobacteria;D_3__Rhizobiales;D_4__Beijerinckiaceae |
| Mixed | D_0__Archaea;D_1__Euryarchaeota;D_2__Methanomicrobia;D_3__Methanosarcinales;D_4__Methanosarcinaceae | D_0__Bacteria;D_1__Proteobacteria;D_2__Alphaproteobacteria;D_3__Rickettsiales;D_4__LWSR-14 |
| Mixed | D_0__Archaea;D_1__Euryarchaeota;D_2__Methanomicrobia;D_3__Methanocellales;D_4__Methanocellaceae | D_0__Bacteria;D_1__Proteobacteria;D_2__Deltaproteobacteria;D_3__NB1-j;D_4__uncultured bacterium |
| Mixed | D_0__Archaea;D_1__Euryarchaeota;D_2__Methanomicrobia;D_3__Methanosarcinales;D_4__Methanosarcinaceae | D_0__Bacteria;D_1__FCPU426;D_2__uncultured bacterium;D_3__uncultured bacterium;D_4__uncultured bacterium |
| Mixed | D_0__Bacteria;D_1__Proteobacteria;D_2__Alphaproteobacteria;D_3__Rhizobiales;D_4__Methylocystaceae | D_0__Bacteria;D_1__TM6 (Dependentiae);D_2__uncultured bacterium;D_3__uncultured bacterium;D_4__uncultured bacterium |
| Mixed | D_0__Archaea;D_1__Euryarchaeota;D_2__Methanomicrobia;D_3__Methanomicrobiales;D_4__Methanospirillaceae | D_0__Bacteria;D_1__Actinobacteria;D_2__Thermoleophilia;D_3__Solirubrobacterales;D_4__TM146 |
| Mixed | D_0__Archaea;D_1__Euryarchaeota;D_2__Methanomicrobia;D_3__Methanocellales;D_4__Methanocellaceae | D_0__Bacteria;D_1__Proteobacteria;D_2__Alphaproteobacteria;D_3__Rhodospirillales;D_4__KCM-B-15 |
| Mixed | D_0__Archaea;D_1__Euryarchaeota;D_2__Methanomicrobia;D_3__Methanosarcinales;D_4__Methanosarcinaceae | D_0__Bacteria;D_1__Atribacteria;D_2__uncultured bacterium;D_3__uncultured bacterium;D_4__uncultured bacterium |
| Mixed | D_0__Archaea;D_1__Euryarchaeota;D_2__Methanomicrobia;D_3__Methanomicrobiales;D_4__Methanoregulaceae | D_0__Bacteria;D_1__Proteobacteria;D_2__Betaproteobacteria;D_3__Burkholderiales;__ |
| Mixed | D_0__Archaea;D_1__Euryarchaeota;D_2__Methanomicrobia;__;__ | D_0__Bacteria;D_1__Chloroflexi;D_2__SBR2076;D_3__uncultured bacterium;D_4__uncultured bacterium |
| Mixed | D_0__Archaea;D_1__Euryarchaeota;D_2__Methanomicrobia;D_3__Methanomicrobiales;D_4__Methanospirillaceae | D_0__Bacteria;D_1__Cyanobacteria;D_2__Melainabacteria;D_3__Gastranaerophilales;D_4__uncultured organism |
| Mixed | D_0__Archaea;D_1__Euryarchaeota;D_2__Methanomicrobia;D_3__Methanosarcinales;D_4__Methanosarcinaceae | D_0__Bacteria;D_1__Proteobacteria;D_2__Alphaproteobacteria;D_3__Rhizobiales;__ |
| Mixed | D_0__Archaea;D_1__Euryarchaeota;D_2__Methanomicrobia;D_3__Methanomicrobiales;D_4__Methanospirillaceae | D_0__Bacteria;D_1__Chloroflexi;D_2__Dehalococcoidia;D_3__Dehalococcoidales;D_4__uncultured |
| Mixed | D_0__Archaea;D_1__Euryarchaeota;D_2__Methanobacteria;D_3__Methanobacteriales;D_4__Methanobacteriaceae | D_0__Bacteria;D_1__Bacteroidetes;D_2__Bacteroidia;__;__ |
| Mixed | D_0__Archaea;D_1__Euryarchaeota;D_2__Methanomicrobia;D_3__Methanomicrobiales;D_4__Methanoregulaceae | D_0__Bacteria;D_1__Proteobacteria;D_2__Betaproteobacteria;D_3__Nitrosomonadales;__ |
| Mixed | D_0__Archaea;D_1__Euryarchaeota;D_2__Methanomicrobia;D_3__Methanosarcinales;D_4__Methanosarcinaceae | D_0__Bacteria;D_1__Acidobacteria;D_2__Subgroup 6;Ambiguous_taxa;Ambiguous_taxa |
| Mixed | D_0__Archaea;D_1__Euryarchaeota;D_2__Methanobacteria;D_3__Methanobacteriales;D_4__Methanobacteriaceae | D_0__Bacteria;D_1__Chloroflexi;D_2__Anaerolineae;D_3__Anaerolineales;D_4__Anaerolineaceae |
| Mixed | D_0__Archaea;D_1__Euryarchaeota;D_2__Methanomicrobia;D_3__Methanosarcinales;D_4__Methanosarcinaceae | D_0__Bacteria;D_1__Verrucomicrobia;D_2__S-BQ2-57 soil group;D_3__uncultured bacterium;D_4__uncultured bacterium |
| Mixed | D_0__Archaea;D_1__Euryarchaeota;D_2__Methanomicrobia;D_3__Methanosarcinales;D_4__Methanosarcinaceae | D_0__Bacteria;D_1__Firmicutes;D_2__Clostridia;D_3__Clostridiales;__ |
| Mixed | D_0__Archaea;D_1__Euryarchaeota;D_2__Methanomicrobia;D_3__Methanocellales;D_4__Methanocellaceae | D_0__Bacteria;D_1__Proteobacteria;D_2__Deltaproteobacteria;D_3__Sva0485;D_4__uncultured bacterium |
| Mixed | D_0__Archaea;D_1__Euryarchaeota;D_2__Methanomicrobia;D_3__Methanosarcinales;D_4__Methanosarcinaceae | D_0__Bacteria;D_1__Armatimonadetes;D_2__Chthonomonadetes;D_3__Chthonomonadales;D_4__Chthonomonadaceae |
| Mixed | D_0__Archaea;D_1__Euryarchaeota;D_2__Methanomicrobia;D_3__Methanocellales;D_4__Methanocellaceae | D_0__Bacteria;D_1__Chloroflexi;D_2__Ktedonobacteria;D_3__C0119;D_4__uncultured bacterium |
| Mixed | D_0__Archaea;D_1__Euryarchaeota;D_2__Methanomicrobia;D_3__Methanocellales;D_4__Methanocellaceae | D_0__Bacteria;D_1__Proteobacteria;D_2__Alphaproteobacteria;D_3__Rhizobiales;D_4__Phyllobacteriaceae |
| Mixed | D_0__Archaea;D_1__Euryarchaeota;D_2__Methanomicrobia;D_3__Methanocellales;D_4__Methanocellaceae | D_0__Bacteria;D_1__Actinobacteria;__;__;__ |
| Mixed | D_0__Archaea;D_1__Euryarchaeota;D_2__Methanomicrobia;D_3__Methanosarcinales;D_4__Methanosarcinaceae | D_0__Bacteria;D_1__Proteobacteria;D_2__Alphaproteobacteria;D_3__Rickettsiales;D_4__Holosporaceae |
| Mixed | D_0__Archaea;D_1__Euryarchaeota;D_2__Methanomicrobia;D_3__Methanosarcinales;__ | D_0__Bacteria;D_1__Bacteroidetes;D_2__Bacteroidia;D_3__Bacteroidales;D_4__Porphyromonadaceae |
| Mixed | D_0__Archaea;D_1__Euryarchaeota;D_2__Methanomicrobia;D_3__Methanosarcinales;__ | D_0__Archaea;D_1__Thaumarchaeota;D_2__Group C3;D_3__uncultured archaeon;D_4__uncultured archaeon |
| Mixed | D_0__Archaea;D_1__Euryarchaeota;D_2__Methanobacteria;D_3__Methanobacteriales;D_4__Methanobacteriaceae | D_0__Bacteria;D_1__Saccharibacteria;D_2__uncultured bacterium;D_3__uncultured bacterium;D_4__uncultured bacterium |
| Mixed | D_0__Archaea;D_1__Euryarchaeota;D_2__Methanomicrobia;D_3__Methanosarcinales;D_4__Methanosarcinaceae | D_0__Bacteria;D_1__Proteobacteria;D_2__Alphaproteobacteria;D_3__Rickettsiales;D_4__SM2D12 |
| Mixed | D_0__Archaea;D_1__Euryarchaeota;D_2__Methanomicrobia;__;__ | D_0__Bacteria;D_1__Actinobacteria;D_2__Actinobacteria;D_3__Corynebacteriales;D_4__Mycobacteriaceae |
| Mixed | D_0__Bacteria;D_1__Proteobacteria;D_2__Alphaproteobacteria;D_3__Rhizobiales;D_4__Methylocystaceae | D_0__Bacteria;D_1__Proteobacteria;D_2__Deltaproteobacteria;D_3__Myxococcales;D_4__Blfdi19 |
| Mixed | D_0__Archaea;D_1__Euryarchaeota;D_2__Methanobacteria;D_3__Methanobacteriales;D_4__Methanobacteriaceae | D_0__Bacteria;D_1__Fibrobacteres;D_2__Fibrobacteria;D_3__Fibrobacterales;__ |
| Mixed | D_0__Archaea;D_1__Euryarchaeota;D_2__Methanomicrobia;D_3__Methanocellales;D_4__Methanocellaceae | D_0__Bacteria;D_1__Proteobacteria;D_2__Gammaproteobacteria;D_3__HOC36;__ |
| Mixed | D_0__Archaea;D_1__Euryarchaeota;D_2__Methanomicrobia;D_3__Methanocellales;D_4__Methanocellaceae | D_0__Bacteria;D_1__Firmicutes;D_2__Clostridia;D_3__Clostridiales;D_4__Clostridiaceae 1 |
| Mixed | D_0__Archaea;D_1__Euryarchaeota;D_2__Methanobacteria;D_3__Methanobacteriales;D_4__Methanobacteriaceae | D_0__Bacteria;D_1__Actinobacteria;D_2__Acidimicrobiia;D_3__Acidimicrobiales;__ |
| Mixed | D_0__Bacteria;D_1__Proteobacteria;D_2__Alphaproteobacteria;D_3__Rhizobiales;D_4__Methylocystaceae | D_0__Bacteria;D_1__Proteobacteria;D_2__Betaproteobacteria;D_3__Hydrogenophilales;D_4__Hydrogenophilaceae |
| Mixed | D_0__Archaea;D_1__Euryarchaeota;D_2__Methanobacteria;D_3__Methanobacteriales;D_4__Methanobacteriaceae | D_0__Bacteria;__;__;__;__ |
| Mixed | D_0__Archaea;D_1__Euryarchaeota;D_2__Methanobacteria;D_3__Methanobacteriales;D_4__Methanobacteriaceae | D_0__Bacteria;D_1__Firmicutes;D_2__Bacilli;D_3__Bacillales;D_4__Paenibacillaceae |
| Mixed | D_0__Archaea;D_1__Euryarchaeota;D_2__Methanobacteria;D_3__Methanobacteriales;D_4__Methanobacteriaceae | D_0__Bacteria;D_1__Actinobacteria;D_2__Actinobacteria;D_3__Corynebacteriales;D_4__Mycobacteriaceae |
| Mixed | D_0__Archaea;D_1__Euryarchaeota;D_2__Methanomicrobia;D_3__Methanomicrobiales;D_4__Methanoregulaceae | D_0__Bacteria;D_1__Proteobacteria;D_2__Alphaproteobacteria;D_3__Rickettsiales;D_4__Holosporaceae |
| Mixed | D_0__Archaea;D_1__Euryarchaeota;D_2__Methanomicrobia;D_3__Methanosarcinales;D_4__Methanosaetaceae | D_0__Bacteria;D_1__Actinobacteria;D_2__Acidimicrobiia;D_3__Acidimicrobiales;D_4__uncultured |
| Mixed | D_0__Archaea;D_1__Euryarchaeota;D_2__Methanomicrobia;D_3__Methanosarcinales;D_4__Methanosarcinaceae | D_0__Bacteria;D_1__Proteobacteria;D_2__Betaproteobacteria;D_3__Nitrosomonadales;__ |
| Mixed | D_0__Archaea;D_1__Euryarchaeota;D_2__Methanomicrobia;D_3__Methanosarcinales;D_4__Methanosarcinaceae | D_0__Bacteria;D_1__Firmicutes;D_2__Bacilli;D_3__Lactobacillales;D_4__Streptococcaceae |
| Mixed | D_0__Bacteria;D_1__Proteobacteria;D_2__Alphaproteobacteria;D_3__Rhizobiales;D_4__Bradyrhizobiaceae | D_0__Bacteria;D_1__Proteobacteria;D_2__Alphaproteobacteria;D_3__Rhizobiales;D_4__Methylocystaceae |
| Mixed | D_0__Archaea;D_1__Euryarchaeota;D_2__Methanomicrobia;__;__ | D_0__Bacteria;D_1__Chloroflexi;D_2__SJA-15;D_3__uncultured bacterium;D_4__uncultured bacterium |
| Mixed | D_0__Archaea;D_1__Euryarchaeota;D_2__Methanomicrobia;D_3__Methanosarcinales;__ | D_0__Archaea;D_1__Lokiarchaeota;Ambiguous_taxa;Ambiguous_taxa;Ambiguous_taxa |
| Mixed | D_0__Archaea;D_1__Euryarchaeota;D_2__Methanobacteria;D_3__Methanobacteriales;D_4__Methanobacteriaceae | D_0__Bacteria;D_1__Firmicutes;D_2__Clostridia;D_3__Clostridiales;D_4__Clostridiales vadinBB60 group |
| Mixed | D_0__Archaea;D_1__Euryarchaeota;D_2__Methanomicrobia;D_3__Methanocellales;D_4__Methanocellaceae | D_0__Bacteria;D_1__Chloroflexi;D_2__SBR2076;D_3__uncultured bacterium;D_4__uncultured bacterium |
| Mixed | D_0__Bacteria;D_1__Proteobacteria;D_2__Alphaproteobacteria;D_3__Rhizobiales;D_4__Methylocystaceae | D_0__Bacteria;D_1__Verrucomicrobia;D_2__S-BQ2-57 soil group;D_3__uncultured bacterium;D_4__uncultured bacterium |
| Mixed | D_0__Archaea;D_1__Euryarchaeota;D_2__Methanomicrobia;D_3__Methanosarcinales;D_4__Methanosaetaceae | D_0__Bacteria;D_1__Firmicutes;D_2__Bacilli;D_3__Bacillales;D_4__Alicyclobacillaceae |
| Mixed | D_0__Archaea;D_1__Euryarchaeota;D_2__Methanomicrobia;D_3__Methanosarcinales;D_4__Methanosarcinaceae | D_0__Archaea;D_1__Lokiarchaeota;Ambiguous_taxa;Ambiguous_taxa;Ambiguous_taxa |
| Mixed | D_0__Archaea;D_1__Euryarchaeota;D_2__Methanomicrobia;D_3__Methanosarcinales;__ | D_0__Bacteria;D_1__Armatimonadetes;__;__;__ |
| Mixed | D_0__Archaea;D_1__Euryarchaeota;D_2__Methanobacteria;D_3__Methanobacteriales;D_4__Methanobacteriaceae | D_0__Bacteria;D_1__Chloroflexi;D_2__Ktedonobacteria;D_3__Ktedonobacterales;D_4__HSB OF53-F07 |
| Mixed | D_0__Archaea;D_1__Euryarchaeota;D_2__Methanomicrobia;D_3__Methanomicrobiales;D_4__Methanomicrobiaceae | D_0__Bacteria;D_1__Firmicutes;D_2__Clostridia;D_3__Clostridiales;D_4__Peptostreptococcaceae |
| Mixed | D_0__Archaea;D_1__Euryarchaeota;D_2__Methanomicrobia;D_3__Methanomicrobiales;D_4__Methanoregulaceae | D_0__Bacteria;D_1__Bacteroidetes;D_2__Cytophagia;D_3__Cytophagales;D_4__Flammeovirgaceae |
| Mixed | D_0__Archaea;D_1__Euryarchaeota;D_2__Methanomicrobia;D_3__Methanomicrobiales;D_4__Methanomicrobiaceae | D_0__Bacteria;D_1__Bacteroidetes;D_2__Bacteroidetes vadinHA17;D_3__uncultured bacterium;D_4__uncultured bacterium |
| Mixed | D_0__Archaea;D_1__Euryarchaeota;D_2__Methanomicrobia;D_3__Methanomicrobiales;D_4__Methanoregulaceae | D_0__Bacteria;D_1__Proteobacteria;D_2__Deltaproteobacteria;D_3__Myxococcales;D_4__27F-1492R |
| Mixed | D_0__Archaea;D_1__Euryarchaeota;D_2__Methanomicrobia;D_3__Methanomicrobiales;D_4__Methanoregulaceae | D_0__Bacteria;D_1__Bacteroidetes;__;__;__ |
| Ombrotrophic | D_0__Archaea;D_1__Euryarchaeota;D_2__Methanomicrobia;D_3__Methanomicrobiales;D_4__Methanoregulaceae | D_0__Archaea;D_1__Euryarchaeota;D_2__Methanomicrobia;D_3__Methanosarcinales;D_4__Methanosarcinaceae |
| Ombrotrophic | D_0__Archaea;D_1__Euryarchaeota;D_2__Methanobacteria;D_3__Methanobacteriales;D_4__Methanobacteriaceae | D_0__Bacteria;D_1__Actinobacteria;D_2__Actinobacteria;D_3__Frankiales;D_4__Frankiaceae |
| Ombrotrophic | D_0__Archaea;D_1__Euryarchaeota;D_2__Methanomicrobia;D_3__Methanomicrobiales;D_4__Methanomicrobiaceae | D_0__Archaea;D_1__Miscellaneous Euryarchaeotic Group(MEG);D_2__uncultured archaeon;D_3__uncultured archaeon;D_4__uncultured archaeon |
| Ombrotrophic | D_0__Archaea;D_1__Thaumarchaeota;D_2__Soil Crenarchaeotic Group(SCG);__;__ | D_0__Bacteria;D_1__Proteobacteria;D_2__Alphaproteobacteria;D_3__Rhizobiales;D_4__Methylocystaceae |
| Ombrotrophic | D_0__Archaea;D_1__Euryarchaeota;D_2__Methanomicrobia;D_3__Methanomicrobiales;D_4__Methanoregulaceae | D_0__Bacteria;D_1__Bacteroidetes;D_2__Cytophagia;D_3__Cytophagales;__ |
| Ombrotrophic | D_0__Archaea;D_1__Euryarchaeota;D_2__Methanomicrobia;D_3__Methanosarcinales;D_4__Methanosarcinaceae | D_0__Bacteria;D_1__Proteobacteria;D_2__Deltaproteobacteria;D_3__Myxococcales;D_4__Archangiaceae |
| Ombrotrophic | D_0__Archaea;D_1__Bathyarchaeota;Ambiguous_taxa;Ambiguous_taxa;Ambiguous_taxa | D_0__Archaea;D_1__Euryarchaeota;D_2__Methanomicrobia;D_3__Methanosarcinales;D_4__GOM Arc I |
| Ombrotrophic | D_0__Archaea;D_1__Euryarchaeota;D_2__Methanomicrobia;D_3__Methanosarcinales;D_4__Methanosarcinaceae | D_0__Bacteria;D_1__Planctomycetes;D_2__Pla4 lineage;__;__ |
| Ombrotrophic | D_0__Archaea;D_1__Euryarchaeota;D_2__Methanomicrobia;D_3__Methanosarcinales;__ | D_0__Bacteria;D_1__Chloroflexi;D_2__Dehalococcoidia;D_3__GIF9;D_4__uncultured bacterium |
| Ombrotrophic | D_0__Archaea;D_1__Euryarchaeota;D_2__Methanobacteria;D_3__Methanobacteriales;D_4__Methanobacteriaceae | D_0__Bacteria;D_1__Bacteroidetes;D_2__Cytophagia;D_3__Cytophagales;__ |
| Ombrotrophic | D_0__Archaea;D_1__Euryarchaeota;D_2__Methanomicrobia;D_3__Methanomicrobiales;D_4__Methanoregulaceae | D_0__Bacteria;D_1__Planctomycetes;D_2__Phycisphaerae;D_3__Phycisphaerales;__ |
| Ombrotrophic | D_0__Archaea;D_1__Euryarchaeota;D_2__Methanomicrobia;D_3__Methanocellales;D_4__Methanocellaceae | D_0__Bacteria;D_1__Nitrospirae;D_2__Nitrospira;D_3__Nitrospirales;D_4__FW13 |
| Ombrotrophic | D_0__Archaea;D_1__Euryarchaeota;D_2__Methanomicrobia;D_3__Methanosarcinales;D_4__Methanosaetaceae | D_0__Bacteria;D_1__Actinobacteria;D_2__Thermoleophilia;D_3__Solirubrobacterales;__ |
| Ombrotrophic | D_0__Archaea;D_1__Euryarchaeota;D_2__Methanomicrobia;D_3__Methanosarcinales;D_4__Methanosaetaceae | D_0__Bacteria;D_1__Proteobacteria;D_2__Deltaproteobacteria;D_3__Myxococcales;D_4__Archangiaceae |
| Ombrotrophic | D_0__Archaea;D_1__Euryarchaeota;D_2__Methanomicrobia;D_3__Methanosarcinales;D_4__GOM Arc I | D_0__Bacteria;D_1__Chlorobi;D_2__Chlorobia;D_3__Chlorobiales;D_4__SJA-28 |
| Ombrotrophic | D_0__Archaea;D_1__Euryarchaeota;D_2__Methanobacteria;D_3__Methanobacteriales;D_4__Methanobacteriaceae | D_0__Bacteria;D_1__Proteobacteria;D_2__Alphaproteobacteria;D_3__Rhizobiales;D_4__A0839 |
| Ombrotrophic | D_0__Archaea;D_1__Euryarchaeota;D_2__Methanomicrobia;D_3__Methanosarcinales;D_4__Methanosaetaceae | D_0__Archaea;D_1__Thaumarchaeota;D_2__Soil Crenarchaeotic Group(SCG);__;__ |
| Ombrotrophic | D_0__Archaea;D_1__Euryarchaeota;D_2__Methanomicrobia;D_3__Methanocellales;D_4__Methanocellaceae | D_0__Bacteria;D_1__Planctomycetes;D_2__Phycisphaerae;D_3__Phycisphaerales;__ |
| Ombrotrophic | D_0__Archaea;D_1__Euryarchaeota;D_2__Methanobacteria;D_3__Methanobacteriales;D_4__Methanobacteriaceae | D_0__Archaea;D_1__Thaumarchaeota;__;__;__ |
| Ombrotrophic | D_0__Archaea;D_1__Euryarchaeota;D_2__Methanobacteria;D_3__Methanobacteriales;D_4__Methanobacteriaceae | D_0__Bacteria;D_1__Actinobacteria;D_2__Actinobacteria;D_3__Micrococcales;D_4__Microbacteriaceae |
| Ombrotrophic | D_0__Archaea;D_1__Euryarchaeota;D_2__Methanomicrobia;D_3__Methanomicrobiales;D_4__Methanomicrobiaceae | D_0__Bacteria;D_1__Spirochaetae;D_2__Spirochaetes;D_3__Spirochaetales;D_4__Brevinemataceae |
| Ombrotrophic | D_0__Archaea;D_1__Euryarchaeota;D_2__Methanobacteria;D_3__Methanobacteriales;D_4__Methanobacteriaceae | D_0__Bacteria;D_1__Actinobacteria;D_2__Thermoleophilia;D_3__Gaiellales;D_4__uncultured |
| Ombrotrophic | D_0__Archaea;D_1__Euryarchaeota;D_2__Methanobacteria;D_3__Methanobacteriales;D_4__Methanobacteriaceae | D_0__Bacteria;D_1__Proteobacteria;D_2__Alphaproteobacteria;D_3__Rhizobiales;D_4__Rhizobiaceae |
| Ombrotrophic | D_0__Archaea;D_1__Euryarchaeota;D_2__Methanobacteria;D_3__Methanobacteriales;D_4__Methanobacteriaceae | D_0__Archaea;D_1__Euryarchaeota;D_2__Thermoplasmata;D_3__Thermoplasmatales;D_4__Terrestrial Miscellaneous Gp(TMEG) |
| Ombrotrophic | D_0__Archaea;D_1__Euryarchaeota;D_2__Methanomicrobia;D_3__Methanomicrobiales;D_4__Methanomicrobiaceae | D_0__Bacteria;D_1__Chlorobi;D_2__Chlorobia;D_3__Chlorobiales;D_4__SJA-28 |
| Ombrotrophic | D_0__Archaea;D_1__Euryarchaeota;D_2__Methanomicrobia;D_3__Methanosarcinales;D_4__Methanosarcinaceae | D_0__Bacteria;D_1__Armatimonadetes;D_2__uncultured;__;__ |
| Ombrotrophic | D_0__Bacteria;D_1__Acidobacteria;D_2__Subgroup 2;Ambiguous_taxa;Ambiguous_taxa | D_0__Bacteria;D_1__Proteobacteria;D_2__Alphaproteobacteria;D_3__Rhizobiales;D_4__Methylocystaceae |
| Ombrotrophic | D_0__Archaea;D_1__Euryarchaeota;D_2__Methanomicrobia;D_3__Methanosarcinales;D_4__Methanosarcinaceae | D_0__Bacteria;D_1__Chloroflexi;D_2__SJA-15;D_3__uncultured bacterium;D_4__uncultured bacterium |
| Ombrotrophic | D_0__Archaea;D_1__Euryarchaeota;D_2__Methanomicrobia;__;__ | D_0__Bacteria;D_1__Actinobacteria;D_2__Actinobacteria;D_3__Frankiales;D_4__Acidothermaceae |
| Ombrotrophic | D_0__Archaea;D_1__Euryarchaeota;D_2__Methanomicrobia;D_3__Methanosarcinales;D_4__Methanosarcinaceae | D_0__Bacteria;D_1__Tenericutes;D_2__Mollicutes;D_3__Entomoplasmatales;D_4__Entomoplasmatales Incertae Sedis |
| Ombrotrophic | D_0__Archaea;D_1__Euryarchaeota;D_2__Methanomicrobia;D_3__Methanocellales;D_4__Methanocellaceae | D_0__Archaea;D_1__Thaumarchaeota;D_2__Soil Crenarchaeotic Group(SCG);__;__ |
| Ombrotrophic | D_0__Archaea;D_1__Euryarchaeota;D_2__Methanomicrobia;D_3__Methanosarcinales;D_4__Methanosarcinaceae | D_0__Bacteria;D_1__Bacteroidetes;D_2__Bacteroidetes vadinHA17;__;__ |
| Ombrotrophic | D_0__Archaea;D_1__Euryarchaeota;D_2__Methanomicrobia;D_3__Methanosarcinales;D_4__GOM Arc I | D_0__Bacteria;D_1__Ignavibacteriae;D_2__Ignavibacteria;D_3__Ignavibacteriales;D_4__BSV26 |
| Ombrotrophic | D_0__Archaea;D_1__Euryarchaeota;D_2__Methanobacteria;D_3__Methanobacteriales;D_4__Methanobacteriaceae | D_0__Archaea;D_1__Thaumarchaeota;D_2__South African Gold Mine Gp 1(SAGMCG-1);__;__ |
| Ombrotrophic | D_0__Archaea;D_1__Euryarchaeota;D_2__Methanomicrobia;__;__ | D_0__Bacteria;D_1__Proteobacteria;D_2__Alphaproteobacteria;D_3__Rickettsiales;D_4__Holosporaceae |
| Ombrotrophic | D_0__Archaea;D_1__Euryarchaeota;D_2__Methanomicrobia;D_3__Methanosarcinales;D_4__Methanosaetaceae | D_0__Bacteria;D_1__Acidobacteria;D_2__Subgroup 2;D_3__uncultured bacterium;D_4__uncultured bacterium |
| Ombrotrophic | D_0__Archaea;D_1__Euryarchaeota;D_2__Methanomicrobia;D_3__Methanomicrobiales;D_4__Methanoregulaceae | D_0__Bacteria;D_1__Proteobacteria;D_2__Alphaproteobacteria;D_3__Rhizobiales;D_4__Rhizobiaceae |
| Ombrotrophic | D_0__Archaea;D_1__Euryarchaeota;D_2__Methanomicrobia;D_3__Methanocellales;D_4__Methanocellaceae | D_0__Bacteria;D_1__Firmicutes;D_2__Bacilli;D_3__Bacillales;D_4__Alicyclobacillaceae |
| Ombrotrophic | D_0__Archaea;D_1__Euryarchaeota;D_2__Methanomicrobia;D_3__Methanosarcinales;D_4__GOM Arc I | D_0__Bacteria;D_1__Chloroflexi;D_2__SHA-26;__;__ |
| Ombrotrophic | D_0__Archaea;D_1__Euryarchaeota;D_2__Methanomicrobia;D_3__Methanosarcinales;__ | D_0__Archaea;D_1__Thaumarchaeota;D_2__Group C3;D_3__uncultured archaeon;D_4__uncultured archaeon |
| Ombrotrophic | D_0__Archaea;D_1__Euryarchaeota;D_2__Methanobacteria;D_3__Methanobacteriales;D_4__Methanobacteriaceae | D_0__Bacteria;D_1__Firmicutes;D_2__Clostridia;D_3__Clostridiales;D_4__Ruminococcaceae |
| Ombrotrophic | D_0__Archaea;D_1__Euryarchaeota;D_2__Methanomicrobia;D_3__Methanomicrobiales;D_4__Methanomicrobiaceae | D_0__Bacteria;D_1__Chloroflexi;D_2__Dehalococcoidia;D_3__Dehalococcoidales;D_4__uncultured |
| Ombrotrophic | D_0__Archaea;D_1__Bathyarchaeota;__;__;__ | D_0__Archaea;D_1__Euryarchaeota;D_2__Methanomicrobia;D_3__Methanosarcinales;__ |
| Ombrotrophic | D_0__Archaea;D_1__Euryarchaeota;D_2__Methanomicrobia;D_3__Methanomicrobiales;D_4__Methanomicrobiaceae | D_0__Archaea;D_1__Thaumarchaeota;D_2__Marine Benthic Group A;__;__ |
| Ombrotrophic | D_0__Archaea;D_1__Euryarchaeota;D_2__Methanomicrobia;__;__ | D_0__Bacteria;D_1__Actinobacteria;D_2__Thermoleophilia;D_3__Solirubrobacterales;D_4__YNPFFP1 |
| Ombrotrophic | D_0__Archaea;D_1__Euryarchaeota;D_2__Methanomicrobia;D_3__Methanosarcinales;D_4__GOM Arc I | D_0__Bacteria;D_1__Armatimonadetes;D_2__uncultured;D_3__uncultured bacterium;D_4__uncultured bacterium |
| Ombrotrophic | D_0__Archaea;D_1__Euryarchaeota;D_2__Methanomicrobia;D_3__Methanocellales;D_4__Methanocellaceae | D_0__Bacteria;D_1__Firmicutes;D_2__Clostridia;D_3__Clostridiales;D_4__Clostridiaceae 1 |
| Ombrotrophic | D_0__Archaea;D_1__Euryarchaeota;D_2__Methanomicrobia;D_3__Methanosarcinales;D_4__GOM Arc I | D_0__Archaea;D_1__Thaumarchaeota;D_2__Group C3;D_3__uncultured archaeon;D_4__uncultured archaeon |
| Ombrotrophic | D_0__Archaea;D_1__Euryarchaeota;D_2__Methanomicrobia;D_3__Methanosarcinales;D_4__Methanosaetaceae | D_0__Bacteria;D_1__Actinobacteria;D_2__Acidimicrobiia;D_3__Acidimicrobiales;__ |
| Ombrotrophic | D_0__Archaea;D_1__Euryarchaeota;D_2__Methanobacteria;D_3__Methanobacteriales;D_4__Methanobacteriaceae | D_0__Bacteria;D_1__Proteobacteria;D_2__Betaproteobacteria;D_3__uncultured;D_4__uncultured bacterium |
| Ombrotrophic | D_0__Archaea;D_1__Euryarchaeota;D_2__Methanobacteria;D_3__Methanobacteriales;D_4__Methanobacteriaceae | D_0__Bacteria;D_1__Verrucomicrobia;D_2__OPB35 soil group;__;__ |
| Ombrotrophic | D_0__Bacteria;D_1__Proteobacteria;D_2__Deltaproteobacteria;D_3__Desulfovibrionales;D_4__Desulfovibrionaceae | D_0__Bacteria;D_1__Proteobacteria;D_2__Gammaproteobacteria;D_3__Methylococcales;D_4__Methylococcaceae |
| Ombrotrophic | D_0__Archaea;D_1__Euryarchaeota;D_2__Methanomicrobia;D_3__Methanosarcinales;D_4__Methanosarcinaceae | D_0__Bacteria;D_1__Actinobacteria;D_2__Thermoleophilia;D_3__Solirubrobacterales;D_4__0319-6M6 |
| Ombrotrophic | D_0__Archaea;D_1__Euryarchaeota;D_2__Methanomicrobia;D_3__Methanosarcinales;D_4__Methanosaetaceae | D_0__Bacteria;D_1__Bacteroidetes;D_2__Cytophagia;D_3__Cytophagales;__ |
| Ombrotrophic | D_0__Archaea;D_1__Euryarchaeota;D_2__Methanomicrobia;D_3__Methanosarcinales;__ | D_0__Bacteria;D_1__Proteobacteria;D_2__Gammaproteobacteria;D_3__Enterobacteriales;D_4__Enterobacteriaceae |
| Ombrotrophic | D_0__Archaea;D_1__Euryarchaeota;D_2__Methanomicrobia;D_3__Methanocellales;D_4__Methanocellaceae | D_0__Bacteria;D_1__Deinococcus-Thermus;D_2__Deinococci;D_3__KD3-62;D_4__uncultured bacterium |
| Ombrotrophic | D_0__Archaea;D_1__Thaumarchaeota;D_2__FHMa11 terrestrial group;D_3__uncultured archaeon;D_4__uncultured archaeon | D_0__Bacteria;D_1__Proteobacteria;D_2__Alphaproteobacteria;D_3__Rhizobiales;D_4__Methylocystaceae |
| Ombrotrophic | D_0__Bacteria;D_1__Firmicutes;D_2__Bacilli;D_3__Bacillales;D_4__Alicyclobacillaceae | D_0__Bacteria;D_1__Proteobacteria;D_2__Alphaproteobacteria;D_3__Rhizobiales;D_4__Methylocystaceae |
| Ombrotrophic | D_0__Archaea;D_1__Euryarchaeota;D_2__Methanomicrobia;D_3__Methanomicrobiales;D_4__Methanomicrobiaceae | D_0__Bacteria;D_1__Chloroflexi;D_2__Dehalococcoidia;D_3__vadinBA26;__ |
| Ombrotrophic | D_0__Archaea;D_1__Woesearchaeota (DHVEG-6);__;__;__ | D_0__Bacteria;D_1__Proteobacteria;D_2__Gammaproteobacteria;D_3__Methylococcales;D_4__Methylococcaceae |
| Ombrotrophic | D_0__Archaea;D_1__Bathyarchaeota;__;__;__ | D_0__Archaea;D_1__Euryarchaeota;D_2__Methanomicrobia;D_3__Methanosarcinales;D_4__Methanosaetaceae |
| Ombrotrophic | D_0__Archaea;D_1__Euryarchaeota;D_2__Methanomicrobia;D_3__Methanocellales;D_4__Methanocellaceae | D_0__Bacteria;D_1__Acidobacteria;D_2__Subgroup 13;D_3__uncultured bacterium;D_4__uncultured bacterium |
| Ombrotrophic | D_0__Archaea;D_1__Euryarchaeota;D_2__Methanomicrobia;__;__ | D_0__Bacteria;D_1__Verrucomicrobia;D_2__OPB35 soil group;D_3__uncultured bacterium;D_4__uncultured bacterium |
| Ombrotrophic | D_0__Archaea;D_1__Euryarchaeota;D_2__Methanomicrobia;__;__ | D_0__Bacteria;D_1__Proteobacteria;D_2__Gammaproteobacteria;D_3__Legionellales;D_4__Legionellaceae |
| Ombrotrophic | D_0__Archaea;D_1__Euryarchaeota;D_2__Methanomicrobia;D_3__Methanosarcinales;D_4__Methanosaetaceae | D_0__Bacteria;D_1__Planctomycetes;D_2__Phycisphaerae;D_3__Phycisphaerales;D_4__ODP1230B30.02 sediment group |
| Ombrotrophic | D_0__Archaea;D_1__Euryarchaeota;D_2__Methanomicrobia;__;__ | D_0__Bacteria;D_1__Proteobacteria;D_2__Gammaproteobacteria;D_3__Legionellales;D_4__Coxiellaceae |
| Ombrotrophic | D_0__Archaea;D_1__Euryarchaeota;D_2__Methanomicrobia;D_3__Methanosarcinales;__ | D_0__Bacteria;D_1__Proteobacteria;D_2__Alphaproteobacteria;D_3__Rickettsiales;__ |
| Ombrotrophic | D_0__Archaea;D_1__Bathyarchaeota;Ambiguous_taxa;Ambiguous_taxa;Ambiguous_taxa | D_0__Archaea;D_1__Euryarchaeota;D_2__Methanomicrobia;D_3__Methanosarcinales;__ |
| Ombrotrophic | D_0__Archaea;D_1__Euryarchaeota;D_2__Methanomicrobia;D_3__Methanocellales;D_4__Methanocellaceae | D_0__Bacteria;D_1__Planctomycetes;D_2__Phycisphaerae;__;__ |
| Ombrotrophic | D_0__Bacteria;D_1__Actinobacteria;D_2__Actinobacteria;D_3__Corynebacteriales;D_4__Mycobacteriaceae | D_0__Bacteria;D_1__Proteobacteria;D_2__Alphaproteobacteria;D_3__Rhizobiales;D_4__Methylocystaceae |
| Ombrotrophic | D_0__Archaea;D_1__Euryarchaeota;D_2__Methanomicrobia;D_3__Methanosarcinales;D_4__GOM Arc I | D_0__Bacteria;D_1__Acidobacteria;D_2__Holophagae;D_3__Holophagales;D_4__Holophagaceae |
| Ombrotrophic | D_0__Archaea;D_1__Euryarchaeota;D_2__Methanomicrobia;D_3__Methanocellales;D_4__Methanocellaceae | D_0__Bacteria;D_1__Actinobacteria;D_2__Actinobacteria;D_3__Corynebacteriales;D_4__Mycobacteriaceae |
| Ombrotrophic | D_0__Archaea;D_1__Euryarchaeota;D_2__Methanomicrobia;D_3__Methanosarcinales;D_4__Methanosaetaceae | D_0__Bacteria;D_1__Proteobacteria;D_2__Gammaproteobacteria;D_3__X35;__ |
| Ombrotrophic | D_0__Archaea;D_1__Euryarchaeota;D_2__Methanobacteria;D_3__Methanobacteriales;D_4__Methanobacteriaceae | D_0__Bacteria;D_1__Planctomycetes;D_2__Phycisphaerae;D_3__Phycisphaerales;D_4__ODP1230B30.02 sediment group |
| Ombrotrophic | D_0__Archaea;D_1__Euryarchaeota;D_2__Methanomicrobia;D_3__Methanocellales;D_4__Methanocellaceae | D_0__Bacteria;D_1__Ignavibacteriae;D_2__Ignavibacteria;D_3__Ignavibacteriales;__ |
| Ombrotrophic | D_0__Archaea;D_1__Euryarchaeota;D_2__Methanomicrobia;D_3__Methanosarcinales;D_4__Methanosaetaceae | D_0__Bacteria;D_1__Proteobacteria;D_2__Alphaproteobacteria;D_3__Rhizobiales;D_4__Rhizobiaceae |
| Ombrotrophic | D_0__Bacteria;D_1__Proteobacteria;D_2__Alphaproteobacteria;D_3__Rhizobiales;D_4__Methylocystaceae | D_0__Bacteria;D_1__Proteobacteria;D_2__Deltaproteobacteria;D_3__Myxococcales;D_4__mle1-27 |
| Ombrotrophic | D_0__Archaea;D_1__Euryarchaeota;D_2__Methanomicrobia;D_3__Methanosarcinales;D_4__Methanosaetaceae | D_0__Archaea;D_1__Parvarchaeota;D_2__uncultured archaeon;D_3__uncultured archaeon;D_4__uncultured archaeon |
| Ombrotrophic | D_0__Archaea;D_1__Euryarchaeota;D_2__Methanobacteria;D_3__Methanobacteriales;D_4__Methanobacteriaceae | D_0__Bacteria;D_1__Actinobacteria;D_2__Actinobacteria;D_3__Streptosporangiales;D_4__Thermomonosporaceae |
| Ombrotrophic | D_0__Archaea;D_1__Euryarchaeota;D_2__Methanobacteria;D_3__Methanobacteriales;D_4__Methanobacteriaceae | D_0__Bacteria;D_1__Proteobacteria;D_2__Gammaproteobacteria;D_3__X35;__ |
| Ombrotrophic | D_0__Bacteria;D_1__Planctomycetes;D_2__Pla4 lineage;__;__ | D_0__Bacteria;D_1__Proteobacteria;D_2__Alphaproteobacteria;D_3__Rhizobiales;D_4__Methylocystaceae |
| Ombrotrophic | D_0__Archaea;D_1__Euryarchaeota;D_2__Methanomicrobia;D_3__Methanosarcinales;D_4__Methanosaetaceae | D_0__Bacteria;D_1__Planctomycetes;D_2__Phycisphaerae;D_3__CPla-3 termite group;__ |
| Ombrotrophic | D_0__Archaea;D_1__Euryarchaeota;D_2__Methanomicrobia;D_3__Methanosarcinales;__ | D_0__Bacteria;D_1__Armatimonadetes;D_2__uncultured;D_3__uncultured bacterium;D_4__uncultured bacterium |
| Ombrotrophic | D_0__Archaea;D_1__Euryarchaeota;D_2__Methanomicrobia;D_3__Methanosarcinales;__ | D_0__Bacteria;D_1__Cyanobacteria;D_2__Melainabacteria;D_3__Gastranaerophilales;D_4__uncultured bacterium |
| Ombrotrophic | D_0__Archaea;D_1__Euryarchaeota;D_2__Methanomicrobia;__;__ | D_0__Bacteria;D_1__Actinobacteria;D_2__Actinobacteria;__;__ |
| Ombrotrophic | D_0__Archaea;D_1__Euryarchaeota;D_2__Methanomicrobia;D_3__Methanosarcinales;__ | D_0__Bacteria;D_1__Proteobacteria;D_2__Betaproteobacteria;D_3__Methylophilales;D_4__Methylophilaceae |
| Ombrotrophic | D_0__Archaea;D_1__Euryarchaeota;D_2__Methanomicrobia;D_3__Methanosarcinales;D_4__GOM Arc I | D_0__Bacteria;D_1__Chloroflexi;D_2__Ktedonobacteria;D_3__B12-WMSP1;__ |
| Ombrotrophic | D_0__Bacteria;D_1__Nitrospirae;D_2__Nitrospira;D_3__Nitrospirales;D_4__FW13 | D_0__Bacteria;D_1__Proteobacteria;D_2__Alphaproteobacteria;D_3__Rhizobiales;D_4__Methylocystaceae |
| Ombrotrophic | D_0__Archaea;D_1__Euryarchaeota;D_2__Methanomicrobia;D_3__Methanomicrobiales;D_4__Methanoregulaceae | D_0__Bacteria;D_1__Planctomycetes;D_2__Phycisphaerae;D_3__CPla-3 termite group;__ |
| Ombrotrophic | D_0__Archaea;D_1__Euryarchaeota;D_2__Methanomicrobia;D_3__Methanocellales;D_4__Methanocellaceae | D_0__Bacteria;D_1__Planctomycetes;D_2__Pla4 lineage;__;__ |
| Ombrotrophic | D_0__Archaea;D_1__Euryarchaeota;D_2__Methanomicrobia;D_3__Methanocellales;D_4__Methanocellaceae | D_0__Bacteria;D_1__Proteobacteria;D_2__Deltaproteobacteria;D_3__Deltaproteobacteria Incertae Sedis;D_4__Syntrophorhabdaceae |
| Ombrotrophic | D_0__Archaea;D_1__Euryarchaeota;D_2__Methanobacteria;D_3__Methanobacteriales;D_4__Methanobacteriaceae | D_0__Bacteria;D_1__Actinobacteria;D_2__Acidimicrobiia;D_3__Acidimicrobiales;D_4__uncultured |
| Ombrotrophic | D_0__Archaea;D_1__Euryarchaeota;D_2__Methanomicrobia;D_3__Methanosarcinales;D_4__Methanosaetaceae | D_0__Bacteria;D_1__Proteobacteria;D_2__Alphaproteobacteria;D_3__Rhodospirillales;D_4__DA111 |
| Ombrotrophic | D_0__Archaea;D_1__Euryarchaeota;D_2__Methanomicrobia;D_3__Methanosarcinales;D_4__Methanosaetaceae | D_0__Bacteria;D_1__Actinobacteria;D_2__Acidimicrobiia;D_3__Acidimicrobiales;D_4__uncultured |
| Ombrotrophic | D_0__Archaea;D_1__Euryarchaeota;D_2__Methanomicrobia;D_3__Methanosarcinales;__ | D_0__Bacteria;D_1__Actinobacteria;D_2__Actinobacteria;D_3__Corynebacteriales;D_4__Mycobacteriaceae |
| Ombrotrophic | D_0__Archaea;D_1__Euryarchaeota;D_2__Methanomicrobia;D_3__Methanosarcinales;D_4__GOM Arc I | D_0__Bacteria;D_1__Chloroflexi;D_2__Ktedonobacteria;D_3__Ktedonobacterales;D_4__JG30a-KF-32 |
| Ombrotrophic | D_0__Archaea;D_1__Euryarchaeota;D_2__Methanobacteria;D_3__Methanobacteriales;D_4__Methanobacteriaceae | D_0__Bacteria;D_1__Tenericutes;D_2__Mollicutes;D_3__Entomoplasmatales;D_4__Entomoplasmatales Incertae Sedis |
| Ombrotrophic | D_0__Archaea;D_1__Euryarchaeota;D_2__Methanomicrobia;D_3__Methanosarcinales;D_4__Methanosaetaceae | D_0__Bacteria;D_1__Actinobacteria;D_2__Thermoleophilia;D_3__Gaiellales;D_4__uncultured |
| Ombrotrophic | D_0__Archaea;D_1__Euryarchaeota;D_2__Methanobacteria;D_3__Methanobacteriales;D_4__Methanobacteriaceae | D_0__Archaea;D_1__Euryarchaeota;D_2__Methanomicrobia;D_3__Methanosarcinales;D_4__Methanosaetaceae |
| Ombrotrophic | D_0__Archaea;D_1__Euryarchaeota;D_2__Methanobacteria;D_3__Methanobacteriales;D_4__Methanobacteriaceae | D_0__Archaea;D_1__Euryarchaeota;D_2__Methanomicrobia;D_3__Methanomicrobiales;D_4__Methanoregulaceae |
| Ombrotrophic | D_0__Archaea;D_1__Euryarchaeota;D_2__Methanomicrobia;D_3__Methanomicrobiales;D_4__Methanomicrobiaceae | D_0__Bacteria;D_1__Elusimicrobia;D_2__Elusimicrobia;D_3__Lineage IIa;D_4__uncultured bacterium |
| Ombrotrophic | D_0__Archaea;D_1__Euryarchaeota;D_2__Methanomicrobia;D_3__Methanocellales;D_4__Methanocellaceae | D_0__Bacteria;D_1__Cyanobacteria;D_2__Melainabacteria;D_3__Gastranaerophilales;D_4__uncultured bacterium |
| Ombrotrophic | D_0__Bacteria;D_1__Planctomycetes;D_2__Phycisphaerae;__;__ | D_0__Bacteria;D_1__Proteobacteria;D_2__Alphaproteobacteria;D_3__Rhizobiales;D_4__Methylocystaceae |
| Ombrotrophic | D_0__Bacteria;D_1__Proteobacteria;D_2__Alphaproteobacteria;D_3__Rhizobiales;D_4__Xanthobacteraceae | D_0__Bacteria;D_1__Proteobacteria;D_2__Gammaproteobacteria;D_3__Methylococcales;D_4__Methylococcaceae |
| Ombrotrophic | D_0__Archaea;D_1__Euryarchaeota;D_2__Methanomicrobia;D_3__Methanocellales;D_4__Methanocellaceae | D_0__Bacteria;D_1__Verrucomicrobia;D_2__S-BQ2-57 soil group;D_3__uncultured bacterium;D_4__uncultured bacterium |
| Ombrotrophic | D_0__Bacteria;D_1__Proteobacteria;D_2__Betaproteobacteria;D_3__SC-I-84;__ | D_0__Bacteria;D_1__Proteobacteria;D_2__Gammaproteobacteria;D_3__Methylococcales;D_4__Methylococcaceae |
| Ombrotrophic | D_0__Archaea;D_1__Euryarchaeota;D_2__Methanomicrobia;D_3__Methanocellales;D_4__Methanocellaceae | D_0__Bacteria;D_1__Actinobacteria;D_2__Actinobacteria;D_3__Kineosporiales;D_4__Kineosporiaceae |
| Ombrotrophic | D_0__Archaea;D_1__Euryarchaeota;D_2__Methanomicrobia;D_3__Methanosarcinales;D_4__Methanosaetaceae | D_0__Bacteria;D_1__Proteobacteria;D_2__Gammaproteobacteria;D_3__Xanthomonadales;D_4__Xanthomonadales Incertae Sedis |
| Ombrotrophic | D_0__Archaea;D_1__Euryarchaeota;D_2__Methanomicrobia;__;__ | D_0__Bacteria;D_1__Actinobacteria;D_2__Acidimicrobiia;D_3__Acidimicrobiales;D_4__uncultured |
| Ombrotrophic | D_0__Archaea;D_1__Euryarchaeota;D_2__Methanobacteria;D_3__Methanobacteriales;D_4__Methanobacteriaceae | D_0__Bacteria;D_1__Proteobacteria;D_2__Gammaproteobacteria;D_3__Xanthomonadales;D_4__Xanthomonadales Incertae Sedis |
| Ombrotrophic | D_0__Archaea;D_1__Euryarchaeota;D_2__Methanomicrobia;D_3__Methanomicrobiales;D_4__Methanomicrobiaceae | D_0__Bacteria;D_1__Ignavibacteriae;D_2__Ignavibacteria;D_3__Ignavibacteriales;D_4__BSV26 |
| Ombrotrophic | D_0__Archaea;D_1__Euryarchaeota;D_2__Methanobacteria;D_3__Methanobacteriales;D_4__Methanobacteriaceae | D_0__Bacteria;D_1__Actinobacteria;D_2__Thermoleophilia;D_3__Solirubrobacterales;D_4__Solirubrobacteraceae |
| Ombrotrophic | D_0__Archaea;D_1__Euryarchaeota;D_2__Methanomicrobia;D_3__Methanomicrobiales;D_4__Methanoregulaceae | D_0__Bacteria;D_1__Acidobacteria;D_2__Holophagae;D_3__Holophagales;D_4__Holophagaceae |
| Ombrotrophic | D_0__Archaea;D_1__Euryarchaeota;D_2__Methanomicrobia;D_3__Methanomicrobiales;D_4__Methanomicrobiaceae | D_0__Bacteria;D_1__Firmicutes;__;__;__ |
| Ombrotrophic | D_0__Archaea;D_1__Euryarchaeota;D_2__Methanomicrobia;__;__ | D_0__Bacteria;D_1__Actinobacteria;D_2__Actinobacteria;D_3__Catenulisporales;D_4__Actinospicaceae |
| Ombrotrophic | D_0__Archaea;D_1__Euryarchaeota;D_2__Methanomicrobia;D_3__Methanocellales;D_4__Methanocellaceae | D_0__Bacteria;D_1__Actinobacteria;D_2__Actinobacteria;D_3__Streptosporangiales;D_4__Thermomonosporaceae |
| Ombrotrophic | D_0__Archaea;D_1__Euryarchaeota;D_2__Methanomicrobia;D_3__Methanosarcinales;D_4__GOM Arc I | D_0__Bacteria;D_1__Acidobacteria;D_2__Subgroup 18;__;__ |
| Ombrotrophic | D_0__Archaea;D_1__Euryarchaeota;D_2__Methanomicrobia;D_3__Methanosarcinales;__ | D_0__Bacteria;D_1__FCPU426;__;__;__ |
| Ombrotrophic | D_0__Archaea;D_1__Euryarchaeota;D_2__Methanobacteria;D_3__Methanobacteriales;D_4__Methanobacteriaceae | D_0__Bacteria;D_1__Acidobacteria;D_2__Subgroup 2;D_3__uncultured bacterium;D_4__uncultured bacterium |
| Ombrotrophic | D_0__Archaea;D_1__Bathyarchaeota;Ambiguous_taxa;Ambiguous_taxa;Ambiguous_taxa | D_0__Bacteria;D_1__Proteobacteria;D_2__Alphaproteobacteria;D_3__Rhizobiales;D_4__Methylocystaceae |
| Ombrotrophic | D_0__Archaea;D_1__Euryarchaeota;D_2__Methanomicrobia;__;__ | D_0__Archaea;D_1__Thaumarchaeota;__;__;__ |
| Ombrotrophic | D_0__Bacteria;D_1__Acidobacteria;D_2__Subgroup 18;__;__ | D_0__Bacteria;D_1__Proteobacteria;D_2__Alphaproteobacteria;D_3__Rhizobiales;D_4__Methylocystaceae |
| Ombrotrophic | D_0__Archaea;D_1__Euryarchaeota;D_2__Methanomicrobia;D_3__Methanomicrobiales;D_4__Methanomicrobiaceae | D_0__Bacteria;D_1__Firmicutes;D_2__Clostridia;D_3__Thermoanaerobacterales;D_4__Thermoanaerobacteraceae |
| Ombrotrophic | D_0__Archaea;D_1__Euryarchaeota;D_2__Methanobacteria;D_3__Methanobacteriales;D_4__Methanobacteriaceae | D_0__Bacteria;D_1__Planctomycetes;D_2__Pla4 lineage;__;__ |
| Ombrotrophic | D_0__Archaea;D_1__Euryarchaeota;D_2__Methanomicrobia;D_3__Methanosarcinales;D_4__Methanosaetaceae | D_0__Bacteria;D_1__Bacteroidetes;D_2__Sphingobacteriia;D_3__Sphingobacteriales;D_4__Sphingobacteriaceae |
| Ombrotrophic | D_0__Archaea;D_1__Euryarchaeota;D_2__Methanomicrobia;__;__ | D_0__Archaea;D_1__Euryarchaeota;D_2__Thermoplasmata;D_3__Thermoplasmatales;D_4__Terrestrial Miscellaneous Gp(TMEG) |
| Ombrotrophic | D_0__Archaea;D_1__Euryarchaeota;D_2__Methanomicrobia;D_3__Methanosarcinales;D_4__Methanosaetaceae | D_0__Bacteria;D_1__Proteobacteria;D_2__Alphaproteobacteria;__;__ |
| Ombrotrophic | D_0__Bacteria;D_1__Proteobacteria;D_2__Gammaproteobacteria;D_3__Methylococcales;D_4__Methylococcaceae | D_0__Bacteria;D_1__Proteobacteria;D_2__Gammaproteobacteria;D_3__X35;D_4__uncultured bacterium |
| Ombrotrophic | D_0__Archaea;D_1__Euryarchaeota;D_2__Methanobacteria;D_3__Methanobacteriales;D_4__Methanobacteriaceae | D_0__Bacteria;D_1__Actinobacteria;D_2__Actinobacteria;D_3__Frankiales;D_4__Acidothermaceae |
| Ombrotrophic | D_0__Archaea;D_1__Euryarchaeota;D_2__Methanomicrobia;D_3__Methanosarcinales;D_4__GOM Arc I | D_0__Bacteria;D_1__Proteobacteria;D_2__Betaproteobacteria;D_3__TRA3-20;__ |
| Ombrotrophic | D_0__Archaea;D_1__Euryarchaeota;D_2__Methanomicrobia;D_3__Methanomicrobiales;D_4__Methanoregulaceae | D_0__Bacteria;D_1__Chloroflexi;D_2__SJA-15;D_3__uncultured bacterium;D_4__uncultured bacterium |
| Ombrotrophic | D_0__Archaea;D_1__Euryarchaeota;D_2__Methanomicrobia;D_3__Methanosarcinales;D_4__Methanosaetaceae | D_0__Bacteria;D_1__Verrucomicrobia;D_2__OPB35 soil group;__;__ |
| Ombrotrophic | D_0__Archaea;D_1__Euryarchaeota;D_2__Methanomicrobia;D_3__Methanosarcinales;D_4__Methanosaetaceae | D_0__Bacteria;D_1__Actinobacteria;D_2__Thermoleophilia;D_3__Solirubrobacterales;D_4__Solirubrobacteraceae |
| Ombrotrophic | D_0__Bacteria;D_1__Cyanobacteria;D_2__Melainabacteria;D_3__Gastranaerophilales;D_4__uncultured bacterium | D_0__Bacteria;D_1__Proteobacteria;D_2__Alphaproteobacteria;D_3__Rhizobiales;D_4__Methylocystaceae |
| Ombrotrophic | D_0__Archaea;D_1__Euryarchaeota;D_2__Methanobacteria;D_3__Methanobacteriales;D_4__Methanobacteriaceae | D_0__Archaea;D_1__Euryarchaeota;D_2__Methanomicrobia;D_3__Methanocellales;D_4__Methanocellaceae |
| Ombrotrophic | D_0__Archaea;D_1__Euryarchaeota;D_2__Methanomicrobia;D_3__Methanosarcinales;D_4__Methanosaetaceae | D_0__Bacteria;D_1__Elusimicrobia;D_2__Elusimicrobia;D_3__Lineage IV;__ |
| Ombrotrophic | D_0__Archaea;D_1__Euryarchaeota;D_2__Methanomicrobia;D_3__Methanosarcinales;D_4__Methanosaetaceae | D_0__Bacteria;D_1__Planctomycetes;D_2__Phycisphaerae;D_3__Phycisphaerales;__ |
| Ombrotrophic | D_0__Archaea;D_1__Euryarchaeota;D_2__Methanomicrobia;D_3__Methanosarcinales;D_4__Methanosaetaceae | D_0__Bacteria;D_1__Proteobacteria;D_2__Alphaproteobacteria;D_3__Rhizobiales;D_4__Methylocystaceae |
| Ombrotrophic | D_0__Bacteria;D_1__Deinococcus-Thermus;D_2__Deinococci;D_3__KD3-62;D_4__uncultured bacterium | D_0__Bacteria;D_1__Proteobacteria;D_2__Alphaproteobacteria;D_3__Rhizobiales;D_4__Methylocystaceae |
| Ombrotrophic | D_0__Archaea;D_1__Euryarchaeota;D_2__Methanomicrobia;D_3__Methanosarcinales;D_4__GOM Arc I | D_0__Bacteria;D_1__Proteobacteria;D_2__Gammaproteobacteria;D_3__Enterobacteriales;D_4__Enterobacteriaceae |
| Ombrotrophic | D_0__Archaea;D_1__Euryarchaeota;D_2__Methanomicrobia;D_3__Methanosarcinales;__ | D_0__Bacteria;D_1__Elusimicrobia;D_2__Elusimicrobia;D_3__Lineage IIc;D_4__uncultured bacterium |
| Ombrotrophic | D_0__Archaea;D_1__Euryarchaeota;D_2__Methanomicrobia;__;__ | D_0__Bacteria;D_1__Proteobacteria;D_2__Alphaproteobacteria;D_3__Rickettsiales;D_4__LWSR-14 |
| Ombrotrophic | D_0__Archaea;D_1__Euryarchaeota;D_2__Methanomicrobia;D_3__Methanomicrobiales;D_4__Methanoregulaceae | D_0__Bacteria;D_1__Actinobacteria;D_2__Thermoleophilia;D_3__Solirubrobacterales;D_4__0319-6M6 |
| Ombrotrophic | D_0__Archaea;D_1__Euryarchaeota;D_2__Methanomicrobia;D_3__Methanosarcinales;D_4__Methanosaetaceae | D_0__Bacteria;D_1__Acidobacteria;D_2__Subgroup 2;Ambiguous_taxa;Ambiguous_taxa |
| Ombrotrophic | D_0__Archaea;D_1__Candidate division YNPFFA;__;__;__ | D_0__Bacteria;D_1__Proteobacteria;D_2__Alphaproteobacteria;D_3__Rhizobiales;D_4__Methylocystaceae |
| Ombrotrophic | D_0__Archaea;D_1__Euryarchaeota;D_2__Methanomicrobia;D_3__Methanocellales;D_4__Methanocellaceae | D_0__Bacteria;D_1__Spirochaetae;D_2__Spirochaetes;D_3__Spirochaetales;D_4__Spirochaetaceae |
| Ombrotrophic | D_0__Archaea;D_1__Euryarchaeota;D_2__Methanobacteria;D_3__Methanobacteriales;D_4__Methanobacteriaceae | D_0__Bacteria;D_1__Actinobacteria;D_2__Thermoleophilia;D_3__Solirubrobacterales;D_4__0319-6M6 |
| Ombrotrophic | D_0__Archaea;D_1__Euryarchaeota;D_2__Methanomicrobia;D_3__Methanomicrobiales;D_4__Methanomicrobiaceae | D_0__Bacteria;D_1__WS1;D_2__uncultured bacterium;D_3__uncultured bacterium;D_4__uncultured bacterium |
| Ombrotrophic | D_0__Archaea;D_1__Euryarchaeota;D_2__Methanomicrobia;D_3__Methanomicrobiales;D_4__Methanomicrobiaceae | D_0__Bacteria;D_1__Chloroflexi;D_2__Ktedonobacteria;D_3__Ktedonobacterales;D_4__Ktedonobacteraceae |
| Ombrotrophic | D_0__Archaea;D_1__Aenigmarchaeota;D_2__Deep Sea Euryarchaeotic Group(DSEG);D_3__uncultured archaeon;D_4__uncultured archaeon | D_0__Archaea;D_1__Euryarchaeota;D_2__Methanomicrobia;D_3__Methanocellales;D_4__Methanocellaceae |
| Ombrotrophic | D_0__Archaea;D_1__Euryarchaeota;D_2__Methanomicrobia;D_3__Methanosarcinales;D_4__Methanosaetaceae | D_0__Bacteria;D_1__Saccharibacteria;D_2__uncultured bacterium;D_3__uncultured bacterium;D_4__uncultured bacterium |
| Ombrotrophic | D_0__Archaea;D_1__Euryarchaeota;D_2__Methanomicrobia;D_3__Methanosarcinales;D_4__Methanosaetaceae | D_0__Bacteria;D_1__Chloroflexi;D_2__JG37-AG-4;D_3__uncultured bacterium;D_4__uncultured bacterium |
| Ombrotrophic | D_0__Archaea;D_1__Euryarchaeota;D_2__Methanomicrobia;__;__ | D_0__Bacteria;D_1__Proteobacteria;D_2__Deltaproteobacteria;D_3__Oligoflexales;D_4__0319-6G20 |
| Ombrotrophic | D_0__Archaea;D_1__Euryarchaeota;D_2__Methanobacteria;D_3__Methanobacteriales;D_4__Methanobacteriaceae | D_0__Bacteria;D_1__Proteobacteria;D_2__Gammaproteobacteria;D_3__Xanthomonadales;D_4__Xanthomonadaceae |
| Ombrotrophic | D_0__Bacteria;D_1__Actinobacteria;D_2__Coriobacteriia;D_3__Coriobacteriales;D_4__Coriobacteriaceae | D_0__Bacteria;D_1__Proteobacteria;D_2__Alphaproteobacteria;D_3__Rhizobiales;D_4__Methylocystaceae |
| Ombrotrophic | D_0__Archaea;D_1__Euryarchaeota;D_2__Methanobacteria;D_3__Methanobacteriales;D_4__Methanobacteriaceae | D_0__Bacteria;D_1__Proteobacteria;D_2__Alphaproteobacteria;__;__ |
| Ombrotrophic | D_0__Archaea;D_1__Euryarchaeota;D_2__Methanomicrobia;__;__ | D_0__Bacteria;D_1__Actinobacteria;D_2__Acidimicrobiia;D_3__Acidimicrobiales;__ |
| Ombrotrophic | D_0__Archaea;D_1__Candidate division YNPFFA;__;__;__ | D_0__Archaea;D_1__Euryarchaeota;D_2__Methanomicrobia;D_3__Methanosarcinales;D_4__Methanosaetaceae |
| Ombrotrophic | D_0__Archaea;D_1__Euryarchaeota;D_2__Methanobacteria;D_3__Methanobacteriales;D_4__Methanobacteriaceae | D_0__Bacteria;D_1__Actinobacteria;D_2__Thermoleophilia;D_3__Solirubrobacterales;__ |
| Ombrotrophic | D_0__Archaea;D_1__Euryarchaeota;D_2__Methanomicrobia;D_3__Methanosarcinales;D_4__Methanosaetaceae | D_0__Bacteria;D_1__Aminicenantes;D_2__uncultured bacterium;D_3__uncultured bacterium;D_4__uncultured bacterium |
| Ombrotrophic | D_0__Archaea;D_1__Euryarchaeota;D_2__Methanomicrobia;__;__ | D_0__Bacteria;D_1__Proteobacteria;D_2__Alphaproteobacteria;D_3__Rickettsiales;D_4__[Caedibacter] caryophilus group |
| Ombrotrophic | D_0__Archaea;D_1__Euryarchaeota;D_2__Methanomicrobia;D_3__Methanosarcinales;D_4__GOM Arc I | D_0__Bacteria;D_1__Acidobacteria;D_2__Subgroup 22;D_3__uncultured bacterium;D_4__uncultured bacterium |
| Ombrotrophic | D_0__Archaea;D_1__Euryarchaeota;D_2__Methanomicrobia;D_3__Methanosarcinales;D_4__Methanosaetaceae | D_0__Bacteria;D_1__Actinobacteria;D_2__Actinobacteria;D_3__Catenulisporales;D_4__Actinospicaceae |
| Ombrotrophic | D_0__Archaea;D_1__Euryarchaeota;D_2__Methanomicrobia;D_3__Methanomicrobiales;D_4__Methanomicrobiaceae | D_0__Bacteria;D_1__Chloroflexi;D_2__S085;D_3__uncultured bacterium;D_4__uncultured bacterium |
| Ombrotrophic | D_0__Archaea;D_1__Euryarchaeota;D_2__Methanomicrobia;D_3__Methanosarcinales;D_4__GOM Arc I | D_0__Bacteria;D_1__Bacteroidetes;D_2__Bacteroidetes vadinHA17;D_3__uncultured bacterium;D_4__uncultured bacterium |
| Ombrotrophic | D_0__Archaea;D_1__Euryarchaeota;D_2__Methanomicrobia;D_3__Methanosarcinales;D_4__Methanosarcinaceae | D_0__Bacteria;D_1__Chloroflexi;__;__;__ |
| Ombrotrophic | D_0__Archaea;D_1__Euryarchaeota;D_2__Methanomicrobia;D_3__Methanosarcinales;D_4__Methanosaetaceae | D_0__Bacteria;D_1__Actinobacteria;D_2__Actinobacteria;D_3__Frankiales;D_4__Acidothermaceae |
| Ombrotrophic | D_0__Archaea;D_1__Euryarchaeota;D_2__Methanobacteria;D_3__Methanobacteriales;D_4__Methanobacteriaceae | D_0__Bacteria;D_1__Proteobacteria;D_2__Alphaproteobacteria;D_3__Rickettsiales;D_4__Holosporaceae |
| Ombrotrophic | D_0__Archaea;D_1__Euryarchaeota;D_2__Methanomicrobia;D_3__Methanomicrobiales;D_4__Methanomicrobiaceae | D_0__Bacteria;D_1__Firmicutes;D_2__Clostridia;D_3__Clostridiales;D_4__Peptococcaceae |
| Ombrotrophic | D_0__Archaea;D_1__Euryarchaeota;D_2__Methanomicrobia;D_3__Methanomicrobiales;D_4__Methanoregulaceae | D_0__Bacteria;D_1__Proteobacteria;D_2__Betaproteobacteria;D_3__uncultured;D_4__uncultured bacterium |
| Ombrotrophic | D_0__Archaea;D_1__Euryarchaeota;D_2__Methanomicrobia;D_3__Methanocellales;D_4__Methanocellaceae | D_0__Bacteria;D_1__Acidobacteria;D_2__Subgroup 18;D_3__uncultured bacterium;D_4__uncultured bacterium |
| Ombrotrophic | D_0__Bacteria;D_1__Proteobacteria;D_2__Alphaproteobacteria;D_3__Rhizobiales;D_4__Methylocystaceae | D_0__Bacteria;D_1__Proteobacteria;D_2__Deltaproteobacteria;D_3__Myxococcales;D_4__P3OB-42 |
| Ombrotrophic | D_0__Archaea;D_1__Euryarchaeota;D_2__Methanobacteria;D_3__Methanobacteriales;D_4__Methanobacteriaceae | D_0__Bacteria;D_1__Parcubacteria;D_2__uncultured bacterium;D_3__uncultured bacterium;D_4__uncultured bacterium |
| Ombrotrophic | D_0__Archaea;D_1__Euryarchaeota;D_2__Methanomicrobia;D_3__Methanomicrobiales;D_4__Methanomicrobiaceae | D_0__Bacteria;D_1__Proteobacteria;D_2__Betaproteobacteria;D_3__TRA3-20;__ |
| Ombrotrophic | D_0__Archaea;D_1__Euryarchaeota;D_2__Methanomicrobia;D_3__Methanosarcinales;D_4__Methanosaetaceae | D_0__Bacteria;D_1__Proteobacteria;D_2__Deltaproteobacteria;D_3__Oligoflexales;D_4__0319-6G20 |
| Ombrotrophic | D_0__Bacteria;D_1__Chloroflexi;D_2__Ktedonobacteria;D_3__B10-SB3A;D_4__uncultured bacterium | D_0__Bacteria;D_1__Proteobacteria;D_2__Gammaproteobacteria;D_3__Methylococcales;D_4__Methylococcaceae |
| Ombrotrophic | D_0__Archaea;D_1__Euryarchaeota;D_2__Methanomicrobia;D_3__Methanosarcinales;__ | D_0__Bacteria;D_1__Bacteroidetes;D_2__Bacteroidia;D_3__Bacteroidales;__ |
| Ombrotrophic | D_0__Archaea;D_1__Euryarchaeota;D_2__Methanomicrobia;D_3__Methanosarcinales;D_4__Methanosaetaceae | D_0__Bacteria;D_1__Proteobacteria;D_2__Alphaproteobacteria;D_3__Rickettsiales;D_4__Holosporaceae |
| Ombrotrophic | D_0__Archaea;D_1__Euryarchaeota;D_2__Methanomicrobia;D_3__Methanosarcinales;D_4__Methanosarcinaceae | D_0__Bacteria;D_1__Planctomycetes;D_2__Phycisphaerae;__;__ |
| Ombrotrophic | D_0__Archaea;D_1__Euryarchaeota;D_2__Methanomicrobia;D_3__Methanosarcinales;D_4__Methanosaetaceae | D_0__Bacteria;D_1__Actinobacteria;D_2__Actinobacteria;D_3__Corynebacteriales;D_4__Mycobacteriaceae |
| Ombrotrophic | D_0__Archaea;D_1__Euryarchaeota;D_2__Methanomicrobia;D_3__Methanocellales;D_4__Methanocellaceae | D_0__Archaea;D_1__Thaumarchaeota;D_2__FHMa11 terrestrial group;D_3__uncultured archaeon;D_4__uncultured archaeon |
| Ombrotrophic | D_0__Archaea;D_1__Euryarchaeota;D_2__Methanomicrobia;__;__ | D_0__Bacteria;D_1__Chloroflexi;D_2__Ktedonobacteria;D_3__B10-SB3A;D_4__uncultured bacterium |
| Ombrotrophic | D_0__Archaea;D_1__Euryarchaeota;D_2__Methanomicrobia;D_3__Methanomicrobiales;D_4__Methanomicrobiaceae | D_0__Bacteria;D_1__Proteobacteria;D_2__Gammaproteobacteria;D_3__Enterobacteriales;D_4__Enterobacteriaceae |
| Ombrotrophic | D_0__Archaea;D_1__Euryarchaeota;D_2__Methanomicrobia;D_3__Methanosarcinales;D_4__Methanosaetaceae | D_0__Bacteria;D_1__Actinobacteria;D_2__Thermoleophilia;D_3__Solirubrobacterales;D_4__YNPFFP1 |
| Ombrotrophic | D_0__Archaea;D_1__Euryarchaeota;D_2__Methanobacteria;D_3__Methanobacteriales;D_4__Methanobacteriaceae | D_0__Bacteria;D_1__Actinobacteria;D_2__Actinobacteria;D_3__Catenulisporales;D_4__Actinospicaceae |
| Ombrotrophic | D_0__Archaea;D_1__Euryarchaeota;D_2__Methanomicrobia;D_3__Methanomicrobiales;D_4__Methanoregulaceae | D_0__Bacteria;D_1__Tenericutes;D_2__Mollicutes;D_3__Entomoplasmatales;D_4__Entomoplasmatales Incertae Sedis |
| Ombrotrophic | D_0__Archaea;D_1__Euryarchaeota;D_2__Methanomicrobia;D_3__Methanomicrobiales;D_4__Methanoregulaceae | D_0__Bacteria;D_1__Armatimonadetes;D_2__uncultured;__;__ |
| Ombrotrophic | D_0__Archaea;D_1__Euryarchaeota;D_2__Methanomicrobia;__;__ | D_0__Bacteria;D_1__Fibrobacteres;D_2__Fibrobacteria;D_3__Fibrobacterales;D_4__Fibrobacteraceae |
| Ombrotrophic | D_0__Archaea;D_1__Euryarchaeota;D_2__Methanomicrobia;D_3__Methanomicrobiales;D_4__Methanomicrobiaceae | D_0__Bacteria;D_1__Chloroflexi;D_2__Dehalococcoidia;D_3__GIF9;D_4__uncultured bacterium |
| Ombrotrophic | D_0__Archaea;D_1__Euryarchaeota;D_2__Methanomicrobia;__;__ | D_0__Bacteria;D_1__Armatimonadetes;D_2__Armatimonadia;D_3__Armatimonadales;D_4__uncultured bacterium |
| Ombrotrophic | D_0__Archaea;D_1__Euryarchaeota;D_2__Methanomicrobia;D_3__Methanomicrobiales;D_4__Methanoregulaceae | D_0__Bacteria;D_1__Planctomycetes;D_2__Phycisphaerae;D_3__MSBL9;D_4__uncultured bacterium |
| Ombrotrophic | D_0__Archaea;D_1__Euryarchaeota;D_2__Methanobacteria;D_3__Methanobacteriales;D_4__Methanobacteriaceae | D_0__Bacteria;D_1__Actinobacteria;D_2__Actinobacteria;D_3__Corynebacteriales;D_4__Nocardiaceae |
| Ombrotrophic | D_0__Archaea;D_1__Euryarchaeota;D_2__Methanobacteria;D_3__Methanobacteriales;D_4__Methanobacteriaceae | D_0__Bacteria;D_1__Bacteroidetes;D_2__Cytophagia;D_3__Cytophagales;D_4__Flammeovirgaceae |
| Ombrotrophic | D_0__Archaea;D_1__Euryarchaeota;D_2__Methanomicrobia;__;__ | D_0__Bacteria;D_1__Proteobacteria;D_2__Gammaproteobacteria;D_3__X35;__ |
| Ombrotrophic | D_0__Archaea;D_1__Euryarchaeota;D_2__Methanomicrobia;__;__ | D_0__Archaea;D_1__Thaumarchaeota;D_2__South African Gold Mine Gp 1(SAGMCG-1);__;__ |
| Ombrotrophic | D_0__Archaea;D_1__Euryarchaeota;D_2__Methanomicrobia;D_3__Methanosarcinales;__ | D_0__Bacteria;D_1__FCPU426;D_2__uncultured bacterium;D_3__uncultured bacterium;D_4__uncultured bacterium |
| Ombrotrophic | D_0__Archaea;D_1__Euryarchaeota;D_2__Methanomicrobia;D_3__Methanosarcinales;D_4__Methanosaetaceae | D_0__Bacteria;D_1__Proteobacteria;D_2__Alphaproteobacteria;D_3__Rickettsiales;D_4__LWSR-14 |
| Ombrotrophic | D_0__Bacteria;D_1__Acidobacteria;D_2__Subgroup 18;D_3__uncultured bacterium;D_4__uncultured bacterium | D_0__Bacteria;D_1__Proteobacteria;D_2__Alphaproteobacteria;D_3__Rhizobiales;D_4__Methylocystaceae |
| Ombrotrophic | D_0__Archaea;D_1__Euryarchaeota;D_2__Methanomicrobia;D_3__Methanosarcinales;D_4__Methanosaetaceae | D_0__Bacteria;D_1__Verrucomicrobia;D_2__Opitutae;D_3__Opitutales;D_4__Opitutaceae |
| Ombrotrophic | D_0__Archaea;D_1__Euryarchaeota;D_2__Methanomicrobia;__;__ | D_0__Bacteria;D_1__Bacteroidetes;D_2__Cytophagia;D_3__Cytophagales;D_4__Flammeovirgaceae |
| Ombrotrophic | D_0__Archaea;D_1__Euryarchaeota;D_2__Methanomicrobia;D_3__Methanosarcinales;__ | D_0__Bacteria;D_1__Proteobacteria;D_2__Deltaproteobacteria;D_3__Syntrophobacterales;D_4__Syntrophaceae |
| Ombrotrophic | D_0__Archaea;D_1__Euryarchaeota;D_2__Methanomicrobia;D_3__Methanosarcinales;D_4__Methanosarcinaceae | D_0__Bacteria;D_1__Actinobacteria;D_2__Actinobacteria;D_3__Micrococcales;D_4__Microbacteriaceae |
| Ombrotrophic | D_0__Bacteria;D_1__Proteobacteria;D_2__Alphaproteobacteria;D_3__Rhizobiales;D_4__Methylocystaceae | D_0__Bacteria;D_1__Proteobacteria;D_2__Deltaproteobacteria;D_3__Deltaproteobacteria Incertae Sedis;D_4__Syntrophorhabdaceae |
| Ombrotrophic | D_0__Archaea;D_1__Euryarchaeota;D_2__Methanomicrobia;__;__ | D_0__Bacteria;D_1__Proteobacteria;D_2__Gammaproteobacteria;D_3__HTA4;D_4__uncultured bacterium |
| Ombrotrophic | D_0__Archaea;D_1__Euryarchaeota;D_2__Methanomicrobia;D_3__Methanosarcinales;D_4__GOM Arc I | D_0__Bacteria;D_1__WS1;D_2__uncultured bacterium;D_3__uncultured bacterium;D_4__uncultured bacterium |
| Ombrotrophic | D_0__Archaea;D_1__Euryarchaeota;D_2__Methanomicrobia;D_3__Methanosarcinales;D_4__GOM Arc I | D_0__Bacteria;D_1__Chloroflexi;D_2__Dehalococcoidia;D_3__GIF9;D_4__uncultured bacterium |
| Ombrotrophic | D_0__Bacteria;D_1__Chloroflexi;__;__;__ | D_0__Bacteria;D_1__Proteobacteria;D_2__Alphaproteobacteria;D_3__Rhizobiales;D_4__Methylocystaceae |
| Ombrotrophic | D_0__Archaea;D_1__Thaumarchaeota;D_2__Group C3;D_3__uncultured archaeon;D_4__uncultured archaeon | D_0__Bacteria;D_1__Proteobacteria;D_2__Alphaproteobacteria;D_3__Rhizobiales;D_4__Methylocystaceae |
| Ombrotrophic | D_0__Archaea;D_1__Euryarchaeota;D_2__Methanomicrobia;D_3__Methanocellales;D_4__Methanocellaceae | D_0__Bacteria;D_1__Chloroflexi;__;__;__ |
| Ombrotrophic | D_0__Archaea;D_1__Euryarchaeota;D_2__Methanomicrobia;D_3__Methanomicrobiales;D_4__Methanomicrobiaceae | D_0__Bacteria;D_1__Proteobacteria;D_2__Betaproteobacteria;D_3__Nitrosomonadales;__ |
| Ombrotrophic | D_0__Bacteria;D_1__BRC1;D_2__uncultured bacterium;D_3__uncultured bacterium;D_4__uncultured bacterium | D_0__Bacteria;D_1__Proteobacteria;D_2__Alphaproteobacteria;D_3__Rhizobiales;D_4__Methylocystaceae |
| Ombrotrophic | D_0__Archaea;D_1__Euryarchaeota;D_2__Methanomicrobia;D_3__Methanomicrobiales;D_4__Methanomicrobiaceae | D_0__Bacteria;D_1__Chloroflexi;D_2__Anaerolineae;D_3__Anaerolineales;D_4__Anaerolineaceae |
| Ombrotrophic | D_0__Archaea;D_1__Euryarchaeota;D_2__Methanobacteria;D_3__Methanobacteriales;D_4__Methanobacteriaceae | D_0__Bacteria;D_1__Planctomycetes;D_2__Phycisphaerae;__;__ |
| Ombrotrophic | D_0__Archaea;D_1__Euryarchaeota;D_2__Methanomicrobia;D_3__Methanosarcinales;D_4__Methanosaetaceae | D_0__Bacteria;D_1__Actinobacteria;D_2__Actinobacteria;D_3__Kineosporiales;D_4__Kineosporiaceae |
| Ombrotrophic | D_0__Archaea;D_1__Euryarchaeota;D_2__Methanomicrobia;__;__ | D_0__Archaea;D_1__Thaumarchaeota;D_2__FHMa11 terrestrial group;__;__ |
| Ombrotrophic | D_0__Archaea;D_1__Euryarchaeota;D_2__Methanomicrobia;__;__ | D_0__Bacteria;D_1__Parcubacteria;D_2__uncultured bacterium;D_3__uncultured bacterium;D_4__uncultured bacterium |
| Ombrotrophic | D_0__Archaea;D_1__Euryarchaeota;D_2__Methanobacteria;D_3__Methanobacteriales;D_4__Methanobacteriaceae | D_0__Bacteria;D_1__Verrucomicrobia;D_2__OPB35 soil group;D_3__uncultured bacterium;D_4__uncultured bacterium |
| Ombrotrophic | D_0__Archaea;D_1__Euryarchaeota;D_2__Methanobacteria;D_3__Methanobacteriales;D_4__Methanobacteriaceae | D_0__Bacteria;D_1__Actinobacteria;D_2__Thermoleophilia;D_3__Solirubrobacterales;D_4__YNPFFP1 |
| Ombrotrophic | D_0__Archaea;D_1__Euryarchaeota;D_2__Methanomicrobia;D_3__Methanocellales;D_4__Methanocellaceae | D_0__Bacteria;D_1__Actinobacteria;D_2__Coriobacteriia;D_3__Coriobacteriales;D_4__Coriobacteriaceae |
| Ombrotrophic | D_0__Archaea;D_1__Euryarchaeota;D_2__Methanomicrobia;D_3__Methanosarcinales;D_4__GOM Arc I | D_0__Bacteria;D_1__Planctomycetes;D_2__Pla4 lineage;__;__ |
| Ombrotrophic | D_0__Archaea;D_1__Euryarchaeota;D_2__Methanobacteria;D_3__Methanobacteriales;D_4__Methanobacteriaceae | D_0__Bacteria;D_1__Acidobacteria;D_2__Subgroup 2;Ambiguous_taxa;Ambiguous_taxa |
| Ombrotrophic | D_0__Bacteria;D_1__Proteobacteria;D_2__Alphaproteobacteria;D_3__Rhizobiales;D_4__Methylocystaceae | D_0__Bacteria;D_1__Spirochaetae;D_2__Spirochaetes;D_3__Spirochaetales;D_4__Spirochaetaceae |
| Ombrotrophic | D_0__Archaea;D_1__Euryarchaeota;D_2__Methanomicrobia;D_3__Methanocellales;D_4__Methanocellaceae | D_0__Bacteria;D_1__Acidobacteria;D_2__Subgroup 18;__;__ |
| Ombrotrophic | D_0__Archaea;D_1__Euryarchaeota;D_2__Methanomicrobia;D_3__Methanosarcinales;D_4__GOM Arc I | D_0__Bacteria;D_1__Chloroflexi;D_2__Dehalococcoidia;D_3__vadinBA26;D_4__uncultured bacterium |
| Ombrotrophic | D_0__Archaea;D_1__Euryarchaeota;D_2__Methanomicrobia;D_3__Methanosarcinales;D_4__Methanosaetaceae | D_0__Bacteria;D_1__Proteobacteria;D_2__Gammaproteobacteria;D_3__Xanthomonadales;D_4__Xanthomonadaceae |
| Ombrotrophic | D_0__Archaea;D_1__Euryarchaeota;D_2__Methanomicrobia;D_3__Methanosarcinales;__ | D_0__Bacteria;D_1__Proteobacteria;D_2__Deltaproteobacteria;D_3__Syntrophobacterales;D_4__Syntrophobacteraceae |
| Ombrotrophic | D_0__Archaea;D_1__Euryarchaeota;D_2__Methanomicrobia;D_3__Methanocellales;D_4__Methanocellaceae | D_0__Bacteria;D_1__Armatimonadetes;D_2__uncultured;D_3__uncultured bacterium;D_4__uncultured bacterium |
| Ombrotrophic | D_0__Bacteria;D_1__Chloroflexi;D_2__Ktedonobacteria;D_3__Ktedonobacterales;__ | D_0__Bacteria;D_1__Proteobacteria;D_2__Alphaproteobacteria;D_3__Rhizobiales;D_4__Methylocystaceae |
| Ombrotrophic | D_0__Archaea;D_1__Euryarchaeota;D_2__Methanobacteria;D_3__Methanobacteriales;D_4__Methanobacteriaceae | D_0__Bacteria;D_1__Chloroflexi;D_2__Ktedonobacteria;D_3__JG30-KF-AS9;__ |
| Ombrotrophic | D_0__Archaea;D_1__Euryarchaeota;D_2__Methanomicrobia;__;__ | D_0__Bacteria;D_1__Planctomycetes;D_2__Phycisphaerae;D_3__Phycisphaerales;D_4__ODP1230B30.02 sediment group |
| Ombrotrophic | D_0__Archaea;D_1__Euryarchaeota;D_2__Methanomicrobia;D_3__Methanocellales;D_4__Methanocellaceae | D_0__Bacteria;D_1__Cyanobacteria;D_2__Melainabacteria;D_3__Gastranaerophilales;D_4__uncultured organism |
| Ombrotrophic | D_0__Archaea;D_1__Euryarchaeota;D_2__Methanomicrobia;D_3__Methanosarcinales;D_4__Methanosaetaceae | D_0__Bacteria;D_1__Bacteroidetes;D_2__Cytophagia;D_3__Cytophagales;D_4__Flammeovirgaceae |
| Ombrotrophic | D_0__Archaea;D_1__Euryarchaeota;D_2__Methanomicrobia;D_3__Methanosarcinales;__ | D_0__Bacteria;D_1__Firmicutes;D_2__Clostridia;D_3__Clostridiales;D_4__Peptostreptococcaceae |
| Ombrotrophic | D_0__Archaea;D_1__Lokiarchaeota;D_2__uncultured archaeon;D_3__uncultured archaeon;D_4__uncultured archaeon | D_0__Bacteria;D_1__Proteobacteria;D_2__Alphaproteobacteria;D_3__Rhizobiales;D_4__Methylocystaceae |
| Ombrotrophic | D_0__Archaea;D_1__Euryarchaeota;D_2__Methanomicrobia;D_3__Methanosarcinales;D_4__Methanosarcinaceae | D_0__Bacteria;D_1__Firmicutes;D_2__Clostridia;D_3__Clostridiales;D_4__Ruminococcaceae |
| Ombrotrophic | D_0__Archaea;D_1__Euryarchaeota;D_2__Methanomicrobia;D_3__Methanosarcinales;D_4__Methanosaetaceae | D_0__Bacteria;D_1__Actinobacteria;D_2__Thermoleophilia;D_3__Solirubrobacterales;D_4__0319-6M6 |
| Ombrotrophic | D_0__Archaea;D_1__Thaumarchaeota;D_2__South African Gold Mine Gp 1(SAGMCG-1);D_3__uncultured archaeon;D_4__uncultured archaeon | D_0__Bacteria;D_1__Proteobacteria;D_2__Gammaproteobacteria;D_3__Methylococcales;D_4__Methylococcaceae |
| Ombrotrophic | D_0__Archaea;D_1__Euryarchaeota;D_2__Methanomicrobia;D_3__Methanosarcinales;D_4__Methanosarcinaceae | D_0__Bacteria;D_1__Planctomycetes;D_2__Phycisphaerae;D_3__MSBL9;D_4__uncultured bacterium |
| Ombrotrophic | D_0__Archaea;D_1__Euryarchaeota;D_2__Methanomicrobia;D_3__Methanosarcinales;D_4__Methanosaetaceae | D_0__Bacteria;D_1__Actinobacteria;D_2__Actinobacteria;D_3__Frankiales;D_4__Frankiaceae |
| Ombrotrophic | D_0__Archaea;D_1__Euryarchaeota;D_2__Methanomicrobia;D_3__Methanosarcinales;D_4__Methanosaetaceae | D_0__Bacteria;D_1__Cyanobacteria;D_2__Melainabacteria;D_3__Gastranaerophilales;D_4__uncultured organism |
| Ombrotrophic | D_0__Archaea;D_1__Euryarchaeota;D_2__Methanobacteria;D_3__Methanobacteriales;D_4__Methanobacteriaceae | D_0__Bacteria;D_1__Planctomycetes;D_2__Phycisphaerae;D_3__Phycisphaerales;__ |
| Ombrotrophic | D_0__Archaea;D_1__Euryarchaeota;D_2__Methanomicrobia;D_3__Methanocellales;D_4__Methanocellaceae | D_0__Bacteria;D_1__Proteobacteria;D_2__Alphaproteobacteria;__;__ |
| Ombrotrophic | D_0__Archaea;D_1__Euryarchaeota;D_2__Methanomicrobia;__;__ | D_0__Bacteria;D_1__Saccharibacteria;D_2__uncultured bacterium;D_3__uncultured bacterium;D_4__uncultured bacterium |
| Ombrotrophic | D_0__Archaea;D_1__Euryarchaeota;D_2__Methanomicrobia;D_3__Methanosarcinales;D_4__Methanosaetaceae | D_0__Bacteria;D_1__Verrucomicrobia;D_2__OPB35 soil group;D_3__uncultured bacterium;D_4__uncultured bacterium |
| Ombrotrophic | D_0__Archaea;D_1__Euryarchaeota;D_2__Methanomicrobia;__;__ | D_0__Bacteria;D_1__Chloroflexi;D_2__JG37-AG-4;D_3__uncultured bacterium;D_4__uncultured bacterium |
| Ombrotrophic | D_0__Archaea;D_1__Euryarchaeota;D_2__Methanomicrobia;D_3__Methanosarcinales;__ | D_0__Bacteria;D_1__Deinococcus-Thermus;D_2__Deinococci;D_3__KD3-62;D_4__uncultured bacterium |
| Ombrotrophic | D_0__Archaea;D_1__Euryarchaeota;D_2__Methanomicrobia;D_3__Methanomicrobiales;D_4__Methanomicrobiaceae | D_0__Bacteria;D_1__Proteobacteria;D_2__Deltaproteobacteria;D_3__Sva0485;D_4__uncultured bacterium |
| Ombrotrophic | D_0__Archaea;D_1__Euryarchaeota;D_2__Methanobacteria;D_3__Methanobacteriales;D_4__Methanobacteriaceae | D_0__Bacteria;D_1__Saccharibacteria;D_2__uncultured bacterium;D_3__uncultured bacterium;D_4__uncultured bacterium |
| Ombrotrophic | D_0__Archaea;D_1__Euryarchaeota;D_2__Methanomicrobia;__;__ | D_0__Bacteria;D_1__Chloroflexi;D_2__JG37-AG-4;__;__ |
| Ombrotrophic | D_0__Archaea;D_1__Euryarchaeota;D_2__Methanomicrobia;D_3__Methanosarcinales;D_4__Methanosarcinaceae | D_0__Bacteria;D_1__Acidobacteria;D_2__Subgroup 18;__;__ |
| Ombrotrophic | D_0__Archaea;D_1__Euryarchaeota;D_2__Methanomicrobia;__;__ | D_0__Bacteria;D_1__Actinobacteria;D_2__Actinobacteria;D_3__Pseudonocardiales;D_4__Pseudonocardiaceae |
| Ombrotrophic | D_0__Archaea;D_1__Euryarchaeota;D_2__Methanomicrobia;D_3__Methanosarcinales;D_4__Methanosaetaceae | D_0__Archaea;D_1__Euryarchaeota;D_2__Thermoplasmata;D_3__Thermoplasmatales;D_4__Terrestrial Miscellaneous Gp(TMEG) |
| Ombrotrophic | D_0__Archaea;D_1__Euryarchaeota;D_2__Methanomicrobia;D_3__Methanosarcinales;D_4__Methanosarcinaceae | D_0__Bacteria;D_1__Bacteroidetes;D_2__Bacteroidetes vadinHA17;D_3__uncultured bacterium;D_4__uncultured bacterium |
| Ombrotrophic | D_0__Archaea;D_1__Euryarchaeota;D_2__Methanomicrobia;D_3__Methanocellales;D_4__Methanocellaceae | D_0__Bacteria;D_1__Actinobacteria;D_2__Thermoleophilia;D_3__Solirubrobacterales;D_4__0319-6M6 |
| Ombrotrophic | D_0__Archaea;D_1__Euryarchaeota;D_2__Methanobacteria;D_3__Methanobacteriales;D_4__Methanobacteriaceae | D_0__Bacteria;D_1__Armatimonadetes;D_2__uncultured;__;__ |
| Ombrotrophic | D_0__Archaea;D_1__Euryarchaeota;D_2__Methanomicrobia;D_3__Methanomicrobiales;D_4__Methanoregulaceae | D_0__Bacteria;D_1__Planctomycetes;D_2__Phycisphaerae;__;__ |
| Ombrotrophic | D_0__Archaea;D_1__Euryarchaeota;D_2__Methanomicrobia;D_3__Methanomicrobiales;D_4__Methanomicrobiaceae | D_0__Bacteria;D_1__Proteobacteria;D_2__Alphaproteobacteria;D_3__Sphingomonadales;D_4__Sphingomonadaceae |
| Ombrotrophic | D_0__Archaea;D_1__Euryarchaeota;D_2__Methanomicrobia;D_3__Methanosarcinales;D_4__Methanosaetaceae | D_0__Bacteria;D_1__Actinobacteria;__;__;__ |
| Ombrotrophic | D_0__Archaea;D_1__Euryarchaeota;D_2__Methanomicrobia;D_3__Methanomicrobiales;D_4__Methanomicrobiaceae | D_0__Bacteria;D_1__Proteobacteria;D_2__Alphaproteobacteria;D_3__Rhizobiales;D_4__Roseiarcaceae |
| Ombrotrophic | D_0__Archaea;D_1__Bathyarchaeota;__;__;__ | D_0__Archaea;D_1__Euryarchaeota;D_2__Methanomicrobia;D_3__Methanocellales;D_4__Methanocellaceae |
| Ombrotrophic | D_0__Archaea;D_1__Bathyarchaeota;Ambiguous_taxa;Ambiguous_taxa;Ambiguous_taxa | D_0__Archaea;D_1__Euryarchaeota;D_2__Methanomicrobia;D_3__Methanocellales;D_4__Methanocellaceae |
| Ombrotrophic | D_0__Archaea;D_1__Euryarchaeota;D_2__Methanomicrobia;D_3__Methanocellales;D_4__Methanocellaceae | D_0__Bacteria;D_1__Actinobacteria;D_2__Actinobacteria;D_3__Frankiales;D_4__Frankiaceae |
| Ombrotrophic | D_0__Bacteria;D_1__Acidobacteria;D_2__Subgroup 13;D_3__uncultured bacterium;D_4__uncultured bacterium | D_0__Bacteria;D_1__Proteobacteria;D_2__Alphaproteobacteria;D_3__Rhizobiales;D_4__Methylocystaceae |
| Ombrotrophic | D_0__Archaea;D_1__Euryarchaeota;D_2__Methanomicrobia;D_3__Methanosarcinales;D_4__Methanosarcinaceae | D_0__Bacteria;D_1__BRC1;D_2__uncultured bacterium;D_3__uncultured bacterium;D_4__uncultured bacterium |
| Ombrotrophic | D_0__Archaea;D_1__Euryarchaeota;D_2__Methanomicrobia;D_3__Methanosarcinales;D_4__Methanosarcinaceae | D_0__Bacteria;D_1__Proteobacteria;D_2__Betaproteobacteria;D_3__uncultured;D_4__uncultured bacterium |
| Ombrotrophic | D_0__Archaea;D_1__Euryarchaeota;D_2__Methanomicrobia;D_3__Methanosarcinales;__ | D_0__Archaea;__;__;__;__ |
| Ombrotrophic | D_0__Archaea;D_1__Euryarchaeota;D_2__Methanomicrobia;__;__ | D_0__Bacteria;D_1__Actinobacteria;D_2__Thermoleophilia;D_3__Solirubrobacterales;D_4__Solirubrobacteraceae |
| Ombrotrophic | D_0__Archaea;D_1__Euryarchaeota;D_2__Methanomicrobia;D_3__Methanocellales;D_4__Methanocellaceae | D_0__Bacteria;D_1__Firmicutes;D_2__Negativicutes;D_3__Selenomonadales;D_4__Veillonellaceae |
| Ombrotrophic | D_0__Archaea;D_1__Euryarchaeota;D_2__Methanomicrobia;__;__ | D_0__Archaea;D_1__Woesearchaeota (DHVEG-6);__;__;__ |
| Ombrotrophic | D_0__Archaea;D_1__Euryarchaeota;D_2__Methanomicrobia;__;__ | D_0__Bacteria;D_1__Proteobacteria;D_2__Gammaproteobacteria;D_3__Xanthomonadales;D_4__Xanthomonadales Incertae Sedis |
| Ombrotrophic | D_0__Archaea;D_1__Euryarchaeota;D_2__Methanomicrobia;D_3__Methanosarcinales;__ | D_0__Bacteria;D_1__Proteobacteria;D_2__Betaproteobacteria;D_3__TRA3-20;__ |
| Ombrotrophic | D_0__Bacteria;D_1__Actinobacteria;D_2__Actinobacteria;D_3__Streptosporangiales;D_4__Thermomonosporaceae | D_0__Bacteria;D_1__Proteobacteria;D_2__Alphaproteobacteria;D_3__Rhizobiales;D_4__Methylocystaceae |
| Ombrotrophic | D_0__Bacteria;D_1__Firmicutes;D_2__Clostridia;D_3__Clostridiales;D_4__Clostridiaceae 1 | D_0__Bacteria;D_1__Proteobacteria;D_2__Alphaproteobacteria;D_3__Rhizobiales;D_4__Methylocystaceae |
| Ombrotrophic | D_0__Bacteria;D_1__Ignavibacteriae;D_2__Ignavibacteria;D_3__Ignavibacteriales;__ | D_0__Bacteria;D_1__Proteobacteria;D_2__Alphaproteobacteria;D_3__Rhizobiales;D_4__Methylocystaceae |
| Ombrotrophic | D_0__Archaea;D_1__Euryarchaeota;D_2__Methanomicrobia;D_3__Methanosarcinales;D_4__Methanosaetaceae | D_0__Bacteria;D_1__Fibrobacteres;D_2__Fibrobacteria;D_3__Fibrobacterales;D_4__Fibrobacteraceae |
| Ombrotrophic | D_0__Archaea;D_1__Euryarchaeota;D_2__Methanomicrobia;__;__ | D_0__Archaea;D_1__Parvarchaeota;D_2__uncultured archaeon;D_3__uncultured archaeon;D_4__uncultured archaeon |
| Ombrotrophic | D_0__Archaea;D_1__Bathyarchaeota;__;__;__ | D_0__Bacteria;D_1__Proteobacteria;D_2__Alphaproteobacteria;D_3__Rhizobiales;D_4__Methylocystaceae |
| Ombrotrophic | D_0__Archaea;D_1__Candidate division YNPFFA;__;__;__ | D_0__Archaea;D_1__Euryarchaeota;D_2__Methanomicrobia;D_3__Methanosarcinales;__ |
| Ombrotrophic | D_0__Archaea;D_1__Euryarchaeota;D_2__Methanobacteria;D_3__Methanobacteriales;D_4__Methanobacteriaceae | D_0__Archaea;D_1__Euryarchaeota;D_2__Methanomicrobia;D_3__Methanosarcinales;D_4__Methanosarcinaceae |
| Ombrotrophic | D_0__Archaea;D_1__Euryarchaeota;D_2__Methanomicrobia;D_3__Methanosarcinales;D_4__Methanosaetaceae | D_0__Bacteria;D_1__Actinobacteria;D_2__Actinobacteria;D_3__Corynebacteriales;D_4__Nocardiaceae |
| Ombrotrophic | D_0__Archaea;D_1__Euryarchaeota;D_2__Methanobacteria;D_3__Methanobacteriales;D_4__Methanobacteriaceae | D_0__Bacteria;D_1__Actinobacteria;D_2__Acidimicrobiia;D_3__Acidimicrobiales;__ |
| Ombrotrophic | D_0__Archaea;D_1__Euryarchaeota;D_2__Methanomicrobia;D_3__Methanosarcinales;D_4__Methanosarcinaceae | D_0__Bacteria;D_1__Acidobacteria;D_2__Holophagae;D_3__Holophagales;D_4__Holophagaceae |
| Ombrotrophic | D_0__Archaea;D_1__Euryarchaeota;D_2__Methanobacteria;D_3__Methanobacteriales;D_4__Methanobacteriaceae | D_0__Bacteria;D_1__Planctomycetes;D_2__Phycisphaerae;D_3__CPla-3 termite group;__ |
| Ombrotrophic | D_0__Archaea;D_1__Bathyarchaeota;D_2__uncultured archaeon;D_3__uncultured archaeon;D_4__uncultured archaeon | D_0__Archaea;D_1__Euryarchaeota;D_2__Methanomicrobia;D_3__Methanosarcinales;__ |
| Ombrotrophic | D_0__Archaea;D_1__Euryarchaeota;D_2__Methanobacteria;D_3__Methanobacteriales;D_4__Methanobacteriaceae | D_0__Bacteria;D_1__Verrucomicrobia;D_2__Opitutae;D_3__Opitutales;D_4__Opitutaceae |
| Ombrotrophic | D_0__Archaea;D_1__Euryarchaeota;D_2__Methanomicrobia;D_3__Methanosarcinales;D_4__Methanosarcinaceae | D_0__Bacteria;D_1__Proteobacteria;D_2__Alphaproteobacteria;D_3__Rhizobiales;D_4__A0839 |
| Ombrotrophic | D_0__Archaea;D_1__Euryarchaeota;D_2__Methanomicrobia;D_3__Methanosarcinales;D_4__Methanosaetaceae | D_0__Bacteria;D_1__Parcubacteria;D_2__uncultured bacterium;D_3__uncultured bacterium;D_4__uncultured bacterium |
| Ombrotrophic | D_0__Archaea;D_1__Euryarchaeota;D_2__Methanomicrobia;D_3__Methanomicrobiales;D_4__Methanoregulaceae | D_0__Bacteria;D_1__Bacteroidetes;D_2__Bacteroidetes vadinHA17;D_3__uncultured bacterium;D_4__uncultured bacterium |
| Ombrotrophic | D_0__Archaea;D_1__Euryarchaeota;D_2__Methanomicrobia;__;__ | D_0__Bacteria;D_1__Actinobacteria;D_2__Thermoleophilia;D_3__Solirubrobacterales;__ |
| Ombrotrophic | D_0__Archaea;D_1__Euryarchaeota;D_2__Methanobacteria;D_3__Methanobacteriales;D_4__Methanobacteriaceae | D_0__Bacteria;D_1__Actinobacteria;D_2__Actinobacteria;D_3__Corynebacteriales;D_4__Mycobacteriaceae |
| Ombrotrophic | D_0__Archaea;D_1__Euryarchaeota;D_2__Methanomicrobia;D_3__Methanosarcinales;D_4__Methanosaetaceae | D_0__Archaea;D_1__Thaumarchaeota;D_2__FHMa11 terrestrial group;D_3__uncultured archaeon;D_4__uncultured archaeon |
| Ombrotrophic | D_0__Archaea;D_1__Euryarchaeota;D_2__Methanomicrobia;__;__ | D_0__Bacteria;D_1__Actinobacteria;D_2__Thermoleophilia;D_3__Gaiellales;D_4__uncultured |
| Ombrotrophic | D_0__Bacteria;D_1__Armatimonadetes;D_2__uncultured;D_3__uncultured bacterium;D_4__uncultured bacterium | D_0__Bacteria;D_1__Proteobacteria;D_2__Alphaproteobacteria;D_3__Rhizobiales;D_4__Methylocystaceae |
| Ombrotrophic | D_0__Archaea;D_1__Euryarchaeota;D_2__Methanomicrobia;D_3__Methanosarcinales;__ | D_0__Bacteria;D_1__Proteobacteria;D_2__Betaproteobacteria;D_3__Burkholderiales;D_4__Burkholderiaceae |
| Ombrotrophic | D_0__Archaea;D_1__Euryarchaeota;D_2__Methanomicrobia;__;__ | D_0__Bacteria;D_1__Proteobacteria;D_2__Alphaproteobacteria;__;__ |
| Ombrotrophic | D_0__Archaea;D_1__Euryarchaeota;D_2__Methanomicrobia;D_3__Methanosarcinales;D_4__Methanosaetaceae | D_0__Bacteria;D_1__Proteobacteria;D_2__Alphaproteobacteria;D_3__Rhizobiales;D_4__Rhodobiaceae |
| Ombrotrophic | D_0__Archaea;D_1__Euryarchaeota;D_2__Methanomicrobia;__;__ | D_0__Bacteria;D_1__Planctomycetes;D_2__Phycisphaerae;D_3__CPla-3 termite group;__ |
| Ombrotrophic | D_0__Archaea;D_1__Euryarchaeota;D_2__Methanomicrobia;D_3__Methanomicrobiales;D_4__Methanomicrobiaceae | D_0__Archaea;D_1__Lokiarchaeota;Ambiguous_taxa;Ambiguous_taxa;Ambiguous_taxa |
| Ombrotrophic | D_0__Archaea;D_1__Euryarchaeota;D_2__Methanomicrobia;__;__ | D_0__Bacteria;D_1__Actinobacteria;D_2__Actinobacteria;D_3__Corynebacteriales;D_4__Nocardiaceae |
| Ombrotrophic | D_0__Archaea;D_1__Euryarchaeota;D_2__Methanomicrobia;D_3__Methanocellales;D_4__Methanocellaceae | D_0__Archaea;D_1__Lokiarchaeota;D_2__uncultured archaeon;D_3__uncultured archaeon;D_4__uncultured archaeon |
| Ombrotrophic | D_0__Archaea;D_1__Euryarchaeota;D_2__Methanomicrobia;D_3__Methanocellales;D_4__Methanocellaceae | D_0__Bacteria;D_1__Chloroflexi;D_2__Ktedonobacteria;D_3__Ktedonobacterales;__ |
| Ombrotrophic | D_0__Archaea;D_1__Euryarchaeota;D_2__Methanomicrobia;D_3__Methanosarcinales;D_4__Methanosaetaceae | D_0__Bacteria;D_1__Chloroflexi;D_2__Ktedonobacteria;D_3__Ktedonobacterales;__ |
| Ombrotrophic | D_0__Archaea;D_1__Euryarchaeota;D_2__Methanomicrobia;D_3__Methanomicrobiales;D_4__Methanomicrobiaceae | D_0__Bacteria;D_1__Chloroflexi;D_2__Dehalococcoidia;__;__ |
| Ombrotrophic | D_0__Archaea;D_1__Aigarchaeota;D_2__Terrestrial Hot Spring Gp(THSCG);D_3__uncultured archaeon;D_4__uncultured archaeon | D_0__Archaea;D_1__Euryarchaeota;D_2__Methanomicrobia;D_3__Methanomicrobiales;D_4__Methanomicrobiaceae |
| Ombrotrophic | D_0__Archaea;D_1__Euryarchaeota;D_2__Methanomicrobia;D_3__Methanomicrobiales;D_4__Methanomicrobiaceae | D_0__Bacteria;D_1__Chloroflexi;D_2__Dehalococcoidia;D_3__vadinBA26;D_4__uncultured bacterium |
| Ombrotrophic | D_0__Archaea;D_1__Euryarchaeota;D_2__Methanomicrobia;__;__ | D_0__Bacteria;D_1__Actinobacteria;__;__;__ |
| Ombrotrophic | D_0__Archaea;D_1__Euryarchaeota;D_2__Methanomicrobia;D_3__Methanosarcinales;D_4__Methanosaetaceae | D_0__Bacteria;D_1__Actinobacteria;D_2__Actinobacteria;D_3__Streptosporangiales;D_4__Thermomonosporaceae |
| Ombrotrophic | D_0__Archaea;D_1__Euryarchaeota;D_2__Methanomicrobia;D_3__Methanomicrobiales;D_4__Methanoregulaceae | D_0__Bacteria;D_1__Planctomycetes;D_2__Pla4 lineage;__;__ |
| Ombrotrophic | D_0__Archaea;D_1__Euryarchaeota;D_2__Methanomicrobia;__;__ | D_0__Bacteria;D_1__Proteobacteria;D_2__Alphaproteobacteria;D_3__Caulobacterales;D_4__Caulobacteraceae |
| Ombrotrophic | D_0__Archaea;D_1__Euryarchaeota;D_2__Methanomicrobia;D_3__Methanosarcinales;__ | D_0__Bacteria;D_1__Verrucomicrobia;D_2__OPB35 soil group;Ambiguous_taxa;Ambiguous_taxa |
| Ombrotrophic | D_0__Archaea;D_1__Euryarchaeota;D_2__Methanobacteria;D_3__Methanobacteriales;D_4__Methanobacteriaceae | D_0__Bacteria;D_1__Chloroflexi;D_2__JG37-AG-4;D_3__uncultured bacterium;D_4__uncultured bacterium |
| Ombrotrophic | D_0__Archaea;D_1__Euryarchaeota;D_2__Methanomicrobia;D_3__Methanocellales;D_4__Methanocellaceae | D_0__Bacteria;D_1__Proteobacteria;D_2__Alphaproteobacteria;D_3__Rhizobiales;D_4__Methylocystaceae |
| Ombrotrophic | D_0__Archaea;D_1__Euryarchaeota;D_2__Methanomicrobia;__;__ | D_0__Bacteria;D_1__Proteobacteria;D_2__Alphaproteobacteria;D_3__Rhodospirillales;D_4__DA111 |
| Ombrotrophic | D_0__Archaea;D_1__Euryarchaeota;D_2__Methanomicrobia;D_3__Methanomicrobiales;D_4__Methanoregulaceae | D_0__Bacteria;D_1__Acidobacteria;D_2__Subgroup 18;__;__ |
| Ombrotrophic | D_0__Archaea;D_1__Euryarchaeota;D_2__Methanomicrobia;D_3__Methanocellales;D_4__Methanocellaceae | D_0__Archaea;D_1__Euryarchaeota;D_2__Methanomicrobia;D_3__Methanosarcinales;D_4__Methanosaetaceae |
| Ombrotrophic | D_0__Bacteria;D_1__Proteobacteria;D_2__Alphaproteobacteria;D_3__Rhizobiales;D_4__Methylocystaceae | D_0__Bacteria;D_1__Verrucomicrobia;D_2__S-BQ2-57 soil group;D_3__uncultured bacterium;D_4__uncultured bacterium |
| Ombrotrophic | D_0__Archaea;D_1__Euryarchaeota;D_2__Methanomicrobia;__;__ | D_0__Bacteria;D_1__Elusimicrobia;D_2__Elusimicrobia;D_3__Lineage IV;__ |
| Ombrotrophic | D_0__Archaea;D_1__Euryarchaeota;D_2__Methanomicrobia;D_3__Methanosarcinales;__ | D_0__Bacteria;D_1__Chlamydiae;D_2__Chlamydiae;D_3__Chlamydiales;D_4__Simkaniaceae |
| Ombrotrophic | D_0__Archaea;D_1__Euryarchaeota;D_2__Methanomicrobia;D_3__Methanosarcinales;__ | D_0__Bacteria;D_1__Proteobacteria;D_2__Deltaproteobacteria;D_3__Deltaproteobacteria Incertae Sedis;D_4__Syntrophorhabdaceae |
| Ombrotrophic | D_0__Archaea;D_1__Euryarchaeota;D_2__Methanomicrobia;D_3__Methanomicrobiales;D_4__Methanoregulaceae | D_0__Bacteria;D_1__BRC1;D_2__uncultured bacterium;D_3__uncultured bacterium;D_4__uncultured bacterium |
| Ombrotrophic | D_0__Archaea;D_1__Euryarchaeota;D_2__Methanomicrobia;D_3__Methanomicrobiales;D_4__Methanoregulaceae | D_0__Bacteria;D_1__Actinobacteria;D_2__Actinobacteria;D_3__Catenulisporales;D_4__Actinospicaceae |
| Ombrotrophic | D_0__Archaea;D_1__Euryarchaeota;D_2__Methanobacteria;D_3__Methanobacteriales;D_4__Methanobacteriaceae | D_0__Archaea;D_1__Parvarchaeota;D_2__uncultured archaeon;D_3__uncultured archaeon;D_4__uncultured archaeon |
| Ombrotrophic | D_0__Archaea;D_1__Euryarchaeota;D_2__Methanomicrobia;D_3__Methanosarcinales;D_4__Methanosaetaceae | D_0__Bacteria;D_1__Firmicutes;D_2__Negativicutes;D_3__Selenomonadales;D_4__Veillonellaceae |
| Ombrotrophic | D_0__Archaea;D_1__Euryarchaeota;D_2__Methanomicrobia;D_3__Methanomicrobiales;D_4__Methanoregulaceae | D_0__Bacteria;D_1__Firmicutes;D_2__Clostridia;D_3__Clostridiales;D_4__Ruminococcaceae |
| Ombrotrophic | D_0__Archaea;D_1__Euryarchaeota;D_2__Methanomicrobia;D_3__Methanosarcinales;D_4__Methanosaetaceae | D_0__Archaea;D_1__Thaumarchaeota;__;__;__ |
| Ombrotrophic | D_0__Archaea;D_1__Euryarchaeota;D_2__Methanomicrobia;D_3__Methanomicrobiales;D_4__Methanoregulaceae | D_0__Bacteria;D_1__Chloroflexi;__;__;__ |
| Ombrotrophic | D_0__Archaea;D_1__Euryarchaeota;D_2__Methanomicrobia;__;__ | D_0__Bacteria;D_1__Verrucomicrobia;D_2__OPB35 soil group;__;__ |
| Ombrotrophic | D_0__Archaea;D_1__Euryarchaeota;D_2__Methanomicrobia;D_3__Methanomicrobiales;D_4__Methanomicrobiaceae | D_0__Archaea;D_1__Euryarchaeota;D_2__Methanomicrobia;D_3__Methanosarcinales;D_4__GOM Arc I |
| Ombrotrophic | D_0__Archaea;D_1__Euryarchaeota;D_2__Methanomicrobia;__;__ | D_0__Bacteria;D_1__Proteobacteria;D_2__Alphaproteobacteria;D_3__Rickettsiales;D_4__Rickettsiales Incertae Sedis |
| Ombrotrophic | D_0__Archaea;D_1__Euryarchaeota;D_2__Methanobacteria;D_3__Methanobacteriales;D_4__Methanobacteriaceae | D_0__Bacteria;D_1__Proteobacteria;D_2__Alphaproteobacteria;D_3__Rhizobiales;D_4__Rhodobiaceae |
| Ombrotrophic | D_0__Archaea;D_1__Euryarchaeota;D_2__Methanomicrobia;D_3__Methanocellales;D_4__Methanocellaceae | D_0__Bacteria;D_1__Acidobacteria;D_2__Subgroup 2;Ambiguous_taxa;Ambiguous_taxa |
| Ombrotrophic | D_0__Bacteria;D_1__Proteobacteria;D_2__Alphaproteobacteria;D_3__Rhizobiales;__ | D_0__Bacteria;D_1__Proteobacteria;D_2__Gammaproteobacteria;D_3__Methylococcales;D_4__Methylococcaceae |
| Ombrotrophic | D_0__Archaea;D_1__Euryarchaeota;D_2__Methanomicrobia;D_3__Methanosarcinales;D_4__GOM Arc I | D_0__Bacteria;D_1__Proteobacteria;D_2__Betaproteobacteria;D_3__B1-7BS;D_4__uncultured bacterium |
| Ombrotrophic | D_0__Archaea;D_1__Euryarchaeota;D_2__Methanomicrobia;D_3__Methanosarcinales;D_4__Methanosaetaceae | D_0__Bacteria;D_1__Proteobacteria;D_2__Deltaproteobacteria;D_3__Deltaproteobacteria Incertae Sedis;D_4__Syntrophorhabdaceae |
| Ombrotrophic | D_0__Archaea;D_1__Aenigmarchaeota;D_2__Deep Sea Euryarchaeotic Group(DSEG);D_3__uncultured archaeon;D_4__uncultured archaeon | D_0__Bacteria;D_1__Proteobacteria;D_2__Alphaproteobacteria;D_3__Rhizobiales;D_4__Methylocystaceae |
| Ombrotrophic | D_0__Archaea;D_1__Euryarchaeota;D_2__Methanomicrobia;D_3__Methanomicrobiales;D_4__Methanoregulaceae | D_0__Bacteria;D_1__Actinobacteria;D_2__Actinobacteria;D_3__Micrococcales;D_4__Microbacteriaceae |
| Ombrotrophic | D_0__Archaea;D_1__Euryarchaeota;D_2__Methanobacteria;D_3__Methanobacteriales;D_4__Methanobacteriaceae | D_0__Bacteria;D_1__Proteobacteria;D_2__Deltaproteobacteria;D_3__Myxococcales;D_4__Archangiaceae |
| Ombrotrophic | D_0__Archaea;D_1__Euryarchaeota;D_2__Methanobacteria;D_3__Methanobacteriales;D_4__Methanobacteriaceae | D_0__Bacteria;D_1__Fibrobacteres;D_2__Fibrobacteria;D_3__Fibrobacterales;D_4__Fibrobacteraceae |
